# Gene Expressing and sRNA Sequencing Show That Gene Differentiation Associates with a Yellow* Acer palmatum* Mutant Leaf in Different Light Conditions

**DOI:** 10.1155/2015/843470

**Published:** 2015-12-15

**Authors:** Shu-Shun Li, Qian-Zhong Li, Li-Ping Rong, Ling Tang, Bo Zhang

**Affiliations:** ^1^Institute of Horticulture, Jiangsu Academy of Agricultural Sciences, No. 50 Zhonglingjie, Nanjing 210014, China; ^2^College of Biology and The Environment, Nanjing Forestry University, No. 159 Longpan Road, Nanjing 210037, China

## Abstract

*Acer palmatum* Thunb., like other maples, is a widely ornamental-use small woody tree for leaf shapes and colors. Interestingly, we found a yellow-leaves mutant “Jingling Huangfeng” turned to green when grown in shade or low-density light condition. In order to study the potential mechanism, we performed high-throughput sequencing and obtained 1,082 DEGs in leaves grown in different light conditions that result in* A. palmatum* significant morphological and physiological changes. A total of 989 DEGs were annotated and clustered, of which many DEGs were found associating with the photosynthesis activity and pigment synthesis. The expression of CHS and FDR gene was higher while the expression of FLS gene was lower in full-sunlight condition; this may cause more colorful substance like chalcone and anthocyanin that were produced in full-light condition, thus turning the foliage to yellow. Moreover, this is the first available miRNA collection which contains 67 miRNAs of* A. palmatum*, including 46 conserved miRNAs and 21 novel miRNAs. To get better understanding of which pathways these miRNAs involved, 102 Unigenes were found to be potential targets of them. These results will provide valuable genetic resources for further study on the molecular mechanisms of* Acer palmatum* leaf coloration.

## 1. Introduction

*Acer* genus, belonging to Aceraceae family, is one group of the most important decorative plants and a main source of industrial raw sugar.* Acer palmatum* Thunb., commonly called Japanese maple or smooth Japanese maple, is a species of deciduous shrub or small woody tree originated from Japan, China, North Korea, South Korea, eastern Mongolia, and southeast Russia.* A. palmatum* can reach heights of 6–10 m, often growing as an understory plant in shady woods (The Plant Encyclopedia, http://www.theplantencyclopedia.org/wiki/Acer_palmatum). The root systems are compact and not invasive, and almost all of* A. palmatum* are adaptable and they blend well with companion plants, which are suitable characteristics for borders and ornamental layouts. The germplasm resource collection and breeding of* A. palmatum* have been widely performed in recent years. Over 1,000 cultivars have been chosen for ornamental interests like leaf shapes and colors and propagated by both sexual and asexual reproduction methods. However, molecular biology research on* A. palmatum* is still limited due to the lack of comprehensive genomic resources. Instead, physiological and biochemical approaches are the main approaches recently.

While the phenomena of coloration and changes of tree foliage draw great attentions every year, the mechanisms and reasons are still unclear and controversial [1]. Some reports showed that upregulated anthocyanins protect plant from photoinhibition by reducing excess excitation energies and avoiding oxidative damage [2, 3]. By contrast, anthocyanins in some plants were found with no function in photoprotection [4, 5]. Leaf color is decided mainly by pigments types and content in leaf. Usually, foliage of sugar maple always turned red in autumns, influenced by many factors. The timing and extent of red leaf coloration consistently correlate with foliar nitrogen (N) concentrations and starch or sugar concentrations, influenced by N status [6]. Some research found that autumn leaf redness is inducible and closely linked to photooxidative stress. Most of* A. palmatum* plants have green leaves during growth period, turning red in autumn, except for the Chinese cultivar “Jinling Huangfeng” and Dutch cultivar “Dutch-Yellow-Maple.” “Jinling Huangfeng” is an excellent colorful cultivar selected by Jiangsu Academy of Agriculture, impressing audience by its eternal bright yellow leaves during vigorous growth period. This mutant “Jinling Huangfeng” showed golden-yellow leaves throughout the whole vigorous growth period and turned orange-red in autumn, providing diversely aesthetic merit. Meanwhile, better adaptability, stronger disease resistance, and less leaf-burning possibility phenomenon guarantee “Jinling Huangfeng” more commercial advantages than green ones. In a previous study, a* de novo* transcriptome assembly has been published for* A. palmatum*, resulting in 48436 useful Unigenes, driving* A. palmatum* research into DNA level [7]. The comparison of this yellow mutant to common green-leaf cultivar found 784 upregulated and 1129 downregulated genes, which contain leaf color and stress resistance related genes. After more intense experiments, we found it interesting that this yellow-leaves mutant turned back to green when grown in shades or a low-density light condition. Unfortunately, no report of explanation for such phenomenon can be found in* A. palmatum* to date. This cultivar is perfect for studying leaf coloration mechanism. Furthermore, other physical and morphological changes also need further analysis.

High-throughput sequencing technology can yield millions of reads which code useful information of both DNA and RNA level in organisms, which is a feasible way in understanding genetic changes associated with phenotypes. DEG is widely performed to obtain large amount of transcriptome data from many organisms and tissue types in a cheap and efficient way [8, 9]. It was frequently used for gene expression profile detection with the prior knowledge of transcripts required. Moreover, DEG was usually used to detect expression profiles in different tissues and on development stage and biotic and abiotic treatment [10]. Apart from genes and mRNAs, microRNAs (miRNAs) are also an extremely hot research field. MicroRNAs are known to be fundamental and sequence-specific regulatory elements of eukaryotic genomes among small noncoding RNAs [11]. In plants, miRNAs are parts of a near-ubiquitous class of short (21–24 nt), endogenous single strand RNA molecules that mainly regulate posttranscriptional gene regulation [12].

To fully understand molecular-level in different growing conditions resulting in* A. palmatum* leaf color change, we detected differentially expressed genes (DEGs) and miRNAs of plants growing in shades and controlled conditions. These results will provide valuable genetic resources for further study on the molecular mechanisms of* Acer palmatum* Thunb. and their properties.

## 2. Materials and Methods

### 2.1. Plant Materials and cDNA Preparation

The* A. palmatum* cultivar “Jinling Huangfeng” was used and planted in Jiangsu Academy of Agricultural Sciences, Nanjing, China (E 118.877, N 32.040), for this study. Seedlings were grown in pots using tissue culture method and then were transplanted and grown in plastic pots containing soils : vermiculite mixture (3 : 1) outdoors in natural condition with an average temperature of 25°C/15°C for day/night. All saplings were arranged into three replicated blocks, each comprising two light treatments assigned at random. Half of saplings were grown in a full-sunlight condition of full incident photosynthetic photon flux density, in which an average daytime light density is 1024 *μ*mol·m^−2^ s^−1^. Another half of saplings were grown in shade using two neutral-density black-polypropylene shade cloths, in which an average daytime light density is 287 *μ*mol·m^−2^ s^−1^ (around 30% of full-light density). The former observation and preparatory experiments indicated that the leaf color differentiated to be golden-yellow and green after 30 days of treatment in such conditions. Therefore, we treated half of saplings in shade in a period of 30 days (September 15, 2013, to October 13, 2013) in this study.

For each treatment, young leaves in the same position of different saplings were sampled and then frozen in liquid nitrogen before being stored at −70°C for further experiments. Total RNA was extracted from leaves in two groups using the RNA simply total RNA Kit (Tiangen, Beijing, China) according to the manufacturer's instructions. At least 20 *μ*g of total RNA (>400 ng/*μ*L) was extracted, concentrated using oligo (dT) magnetic adsorption to cleave the fragments, and served as a template for the synthesis of first-strand cDNA. Second-strand cDNA was synthesized using DNA polymerase I and RNaseH, purified by the QiaQuick PCR extraction kit (Qiagen, Hilden, Germany), and resolved with EB buffer for end reparation and poly(A) addition. A suitable length (300 bp to 500 bp) of cDNA fragments was selected by agarose gel electrophoresis and amplified by PCR to construct the final cDNA libraries for sequencing.

A lot of physiological and phenotypic data were collected in this study. Leaf tissue (0.1 g) from each group was immersed in 10 mL of a 9 : 1 (v/v) mix of acetone: 0.1 M NH_4_OH to extract Chl a, Chl b, and carotenoid after shading using spectrophotometer as previously reported [13]. Anthocyanins were extracted and measured. Measurements for saplings from two groups were repeated at least three times, and averages and the standard deviation were calculated using SAS statistics software. The chlorophyll fluorescence was measured by a chlorophyll fluorometer and *F*_*v*_/*F*_*m*_ were calculated to test whether or not plant stress affects Photosystem II in a dark adapted state.

### 2.2. Sequencing and Digital Gene Expression Tag Profiling

The RNA libraries were sequenced with Illumina HiSeq2000. All of the raw data are publicly available at NCBI SRA database under accession SRX833686 (http://www.ncbi.nlm.nih.gov/sra/). The quality score of sequenced tags was tested by a Perl script. In order to test the sequencing confidence and bias, Sequencing Random Test and Sequencing Saturation Test were performed by analyzing mapped reads distribution on the Unigene dataset, coincidence of total tag number and total gene number, respectively. Then, the expression levels of genes of two samples were obtained using digital gene expression tag profiling methods as previously described, which were represented by tag sequence frequency after mapping to the Unigene dataset of Japanese maple [9]. All of the reads were aligned with* A. palmatum* Unigene dataset by Bowtie2 software using default parameters (http://bowtie-bio.sourceforge.net/index.shtml) [14]. To characterize the expression profiles in two samples, RPKM values representing expression values were calculated by using normalized numbers of matching clean tags [15]. The digital expression genes (DEGs) were identified using EBSeq software, and Benjamini-Hochberg Correction Method was implied to reduce false positivity [16]. To avoid the potential noise signal high-throughput sequencing, sequences were defined as DEGs only if they meet two conditions (fold change ≥2.0 and a false discovery rate <0.01). The differential expression levels of the genes in two samples were compared and visualized through scatter plots drawn by in-house Perl scripts.

### 2.3. Annotation and Classification of Tags

Unigenes were annotated by search against various nucleotide and protein databases. BLASTN algorithm was performed with Nt database with a cut-off *E*-value of 10^−5^. BLASTX was performed with Nr database, COG database, KEGG database, Swiss-Prot database, and TrEMBL database using a cut-off *E*-value of 10^−5^. The Blast2GO program was used to obtain GO annotations for each DEG by integrating various databases.

### 2.4. Small RNA Library Construction and Sequencing

Total RNA was extracted from leaves with Trizol reagent following the manufacturer's instructions according to the previously described methods. The total RNA balanced mix sample was fractionated by 15% denaturing polyacrylamide gel electrophoresis, and then the small RNA fragments between 16 and 35 nt were isolated from the gel. The small RNA molecules were ligated to a 5′ adaptor and a 3′ adaptor by T4 RNA ligase. Subsequently, the adapter-ligated small RNAs were converted to cDNA by RT-PCR following the Illumina protocol [17]. Then, the small sRNA library was sequenced with Illumina HiSeq 2500. All of the raw data are available at NCBI SRA database under accession SRX833689.

### 2.5. Identification and Annotation of miRNA

Quality trimming and adaptor removal of the Illumina reads were processed by a common-line Perl script. Firstly, low quality reads containing base quality scores below 20 were discarded. Adapters and poly A sequences were trimmed and then only 18–30 nt sequences remained. The resulting clean reads were firstly processed into nonredundant cluster sequences and aligned to the Unigenes using Bowtie2 with perfect match (no mismatch). Differential miRNAs were also detected with the parameter: absolute fold change of ≥2.0 and a false discovery rate of <0.01. A miRNA target analysis tool miRDeep2 was employed to predict the targets of putative miRNAs in Unigene sequences, with default parameters [18]. The potential target genes further performed protein annotation against GO, COG, NR, Swiss-Prot, and TrEMBL databases with an *E*-value threshold of less than 10^−5^ as the aforementioned.

## 3. Results and Discussion

### 3.1. Physiological and Morphological Index of* A. palmatum*

**Figure 1:**
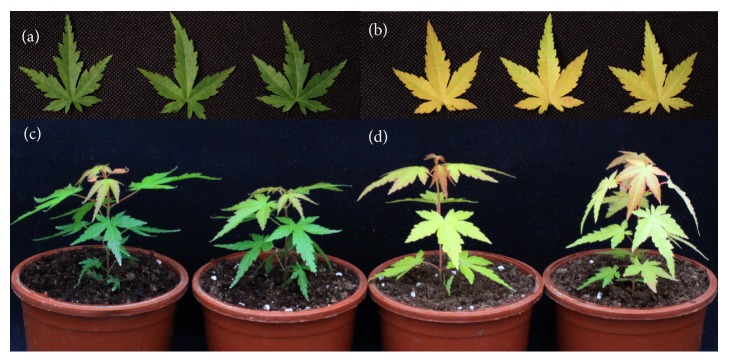
Phenotype of plants in 1-month treatment in shade (GS) and full-sunlight (YF) conditions: (a) GS leaves, (b) YF leaves, (c) GS seedlings, and (d) YF seedlings.

In order to evaluate and quantify physiological and morphological change of* A. palmatum*, we measured eleven indexes of plants. Plants under high light treatment seem to grow taller and wider, supported by stronger root as well as higher overground and underground biomass than those in shade condition. Compared with seedlings grown in full-light condition, the height and shoot diameter of seedlings grown in shade decreased 2.16 cm (13.8%) and 0.24 mm (13.1%). The root vigority and overground and underground biomass declined about 27.7%, 24.7%, and 44.2%, respectively, indicating that GS samples were less vigorous than the YF samples, especially the underground part. On the contrary, the leaf area in GS samples is 767.10 mm^2^ and 1.88 times the YF samples (408.83 mm^2^), which could be a result of the fact that seedlings grown in shade tended to have bigger leaves to trap more photon. Usually, the *F*_*v*_/*F*_*m*_ value falls in the range of 0.79 to 0.84 and tends to be approximate optimal value for many plant species, with lowered values indicating plant stress occurs [19]. In this study, the average *F*_*v*_/*F*_*m*_ value in YF sample is 0.73, which is lower than GS counterpart (0.819, normal) and normal range, indicating that this mutant may suffer from a stress in full-light condition (Table 1, Figure 1). These results showed that* A. palmatum* has not only visible color alteration, but changes in other growth characteristics as well.

The color of maples' leaves may vary in different light conditions. However, there has been no report of leaf color and pigments content information of* A. palmatum* to date. Previous research shows that, in comparison with plants grown in the low and medium light treatments,* A. platanoides* and* A. saccharum* in the high light tress treatments contain lower chlorophyll, carotenoid, and chlorophyll fluorescence parameters (*F*_*v*_/*F*_*m*_), indicating higher levels of photoinhibition existing in the seedlings exposed to high light [20]. In this study, we found that all main pigments contents related to photosynthesis pathway were extremely higher in GS samples, for instance, 11.1, 28.6, and 2.1 times in GS samples as in the YF samples. These results are consistent with the fact that chlorophyll content was higher in shade plants than in heliophytes. Abundant experiments showed that decreasing light density led to an increase in the level of chlorophyll b in the chlorophyll-bearing tissues of plants, as well as in its relative amount of chlorophyll a [21, 22].

Moreover, the content of anthocyanin reached 143.30 *μ*mol g^−1^ FW in GF samples, which was almost three times in plants that grew in shade condition (Table 1).* Acer* plants have been reported to contain five cyanidin glycosides, 3-glucoside, 3-rutinoside, 3-galloylglucoside, 3-galloylrutinoside, and 3,5-diglucoside, as well as two delphinidin glycosides, 3-glucoside and 3-rutinoside, and three unidentified anthocyanins. There were no differences in the pigment constituents in the species native to different countries [23]. Leaf color is potentially decided by the various ratio of different photosynthesis pigments as well as anthocyanins. The combination of extremely lower colorful photosynthesis pigment content and higher anthocyanin indicates a raised hypothesis that shade treatment induced gene expression differentiation in pigments biosynthesis pathway and finally resulted in differences of plant vigor and leaf color.

### 3.2. Sequencing and Expression Profiling of* A. palmatum*

To obtain a comprehensive dataset of* A. palmatum*, high-throughput sequencing was performed. A total of 18.58 million reads were yielded: 8.98 million reads and 9.59 million reads were generated for GF and YS samples, respectively. The raw data have been submitted and deposited in the NCBI Sequence Read Archive. Over 90% reads passed Q20 test and over 83% reads passed Q30 test, which showed high level of sequencing data. To imply Sequencing Random Test, the base frequency was normalized by counting bases numbers in each relative position in genes. The smooth curve of two samples proved the sequencing bias was low. Sequencing Saturation Test was carried out to test whether reads numbers were sufficient. The curve showed that when total tag number reached almost 2.5 million in each sample, the total gene number was stabilized to a constant. Almost 5 million reads in each sample provided the sequenced tags that were sufficient for further analysis. These two tests revealed that sequenced data could reasonably represent practical RNA expression level in leaf tissues (Figure S1 in Supplementary Material available online at http://dx.doi.org/10.1155/2015/843470).

Due to no available genome assembly of* A. palmatum* recently, a dataset containing 48,436 Unigenes detected in previous report was used as a reference of gene sequences [7]. Overall, 35,987 (74.30%) Unigenes were detected at least within one sample in this study. In the clean tags, 84.25% (7.57 million) and 82.11% (7.88 million) reads from GF and YS samples, respectively, could be mapped into Unigene models, and 93.63% (7.08 million) and 94.38% (7.43 million) unique reads could be mapped into Unigenes (Table 2). The high mapping ratio revealed the Unigene dataset could be a good representative of practical gene model of* A. palmatum*, and the sequencing bias indicated by mapping ratio was small and acceptable.

### 3.3. Identification, Functional Annotation, and Clustering of DEGs in* A. palmatum*

A total of 33,699 Unigenes were found to be differentially expressed among two samples. To eradicate accidental error during high-throughput sequencing, criteria of absolute fold change ≥2.0 and a false discovery rate <0.01 were adopted. After correction by Chi-squared test, 1,082 unbiased DEGs were detected, which compromised 356 upregulated Unigenes and 726 downregulated Unigenes in the YF sample.

Protein plays a key role in cell component and metabolism. To know the potential functions of the DEGs, seven canonical databases were used to annotate and classify 1,082 DEGs of* A. palmatum*, providing a comprehensive interpretation from multiple perspectives. Results showed 989 (91.4%) Unigenes were annotated by at least one database. NR database of NCBI is a nonredundant protein collection of all organisms. A total of 976 DEGs were annotated using NR database, and the most similar feature was treated as annotation to unveil the possible function of the corresponding DEG. This method was also conducted using two European protein databases below to provide more comprehensive annotation. To be specific, Swiss-Prot database is a handy annotated and corrected protein database that provides high confidence annotation while TrEMBL database adopts machine autoannotation method to increase annotation scope as a supplement to Swiss-Prot database [24]. After aligning to protein models using these two databases, 811 and 979 DEGs were, respectively, annotated using Swiss-Prot and TrEMBL databases. Finally, 857 DEGs were annotated against a nonredundant nucleotide (NT) database (Table S1).

**Figure 2:**
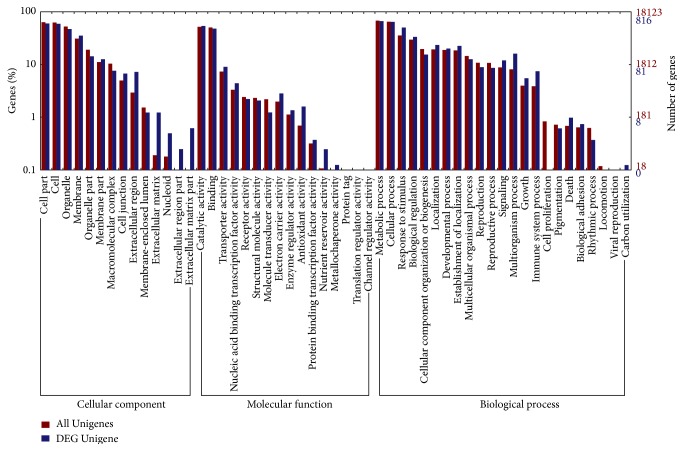
GO annotation of DEGs.

For better understanding of DEGs in pathways, the DEGs were annotated using GO, COG, and KEGG clustering systems. Gene ontology (GO) annotation system integrated protein functions across a diverse range of organisms, providing function knowledge from well-studied organisms to novel organisms by computational methods [25]. A total of 18,123 Unigenes have a homologous match with GO database and they were assigned with GO terms. Results showed these Unigenes could be categorized into 53 subgroups. To compare gene ontology between all Unigenes and DEGs, 816 DEGs existed in 47 groups, except for channel regulator activity, protein tag, translation regulator activity, cell proliferation, viral reproduction, and locomotion. In extracellular matrix, nucleoid, extracellular region part, extracellular matrix part, and nutrient reservoir activity accounted much bigger proportion in DEGs revealed that abundant genes from these groups might be associated with leaf color change. To be specific, shade condition may substantially influence the extracellular pathways and nutrient conservation. In contrast, the ratio of all Unigenes in cell proliferation and locomotion groups that overweighed DEGs showed that these Unigenes may be impossibly influenced by the light condition. Intriguingly, 41 DEGs were found associated with high light stress, compromising 38 upregulated DEGs and 3 downregulated DEGs in YF leaves (Figure 2).

**Figure 3:**
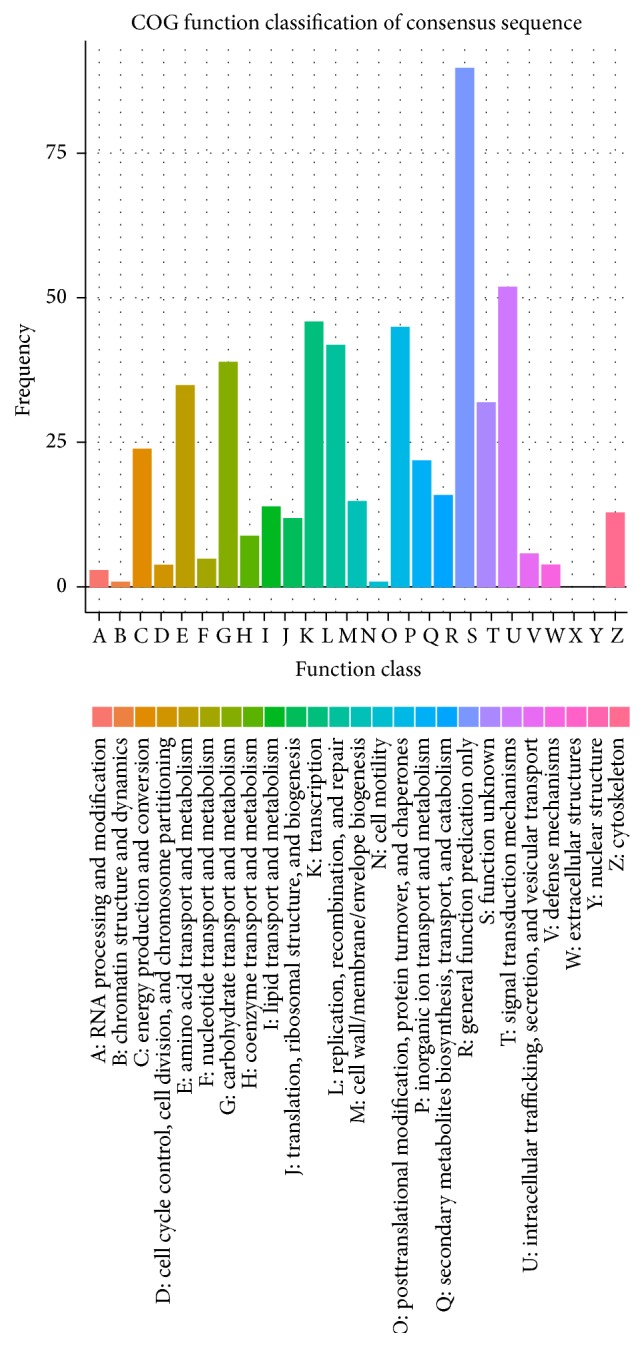
COG classification of DEGs.

The Kyoto Encyclopedia of Genes and Genomes (KEGG) resource (http://www.kegg.jp/) has been developed as a reference knowledge base for biological interpretation of genome sequences and other high-throughput data analysis [26]. To identify the biological pathways represented by the DEGs in this study, we aligned with the KEGG database. In total, 206 Unigenes could be assigned to 187 pathways. The DEGs with a potential role in photosynthesis, hormone signal transduction, and pathogen pathways were listed in Table S1. Among 134 Unigenes associated with plant hormone signal transduction (ko04075), there are abundant DEGs detected in 18 Unigenes. Moreover, 303 of the total DEGs could be classified into Clusters of Orthologous Groups (COG). Top five groups classified in COG database were general function prediction only (90), signal transduction mechanisms (52), transcription (46), posttranslational modification, protein turnover, chaperones (45), and replication, recombination, and repair (42) (Figure 3).

### 3.4. Analysis of Small RNAs* A. palmatum*

To identify conserved and novel miRNAs in* A. palmatum*, two corresponding samples were selected for small RNA sequencing and yielded 29,629,121 reads. After filtered 458 low quality reads and 3088430 (10.4%) reads which were too short or too long, a total of 23,728,039 clean reads were obtained within length of 18–30 nt, which contained 12,750,144 reads in GS sample and 10,977,895 reads in the YF sample. We obtained 9,110,216 unique small RNA sequences, most of which were only found in one sample. In order to characterize small RNAs, the length distribution and abundance of small RNAs in* A. palmatum* leaves were calculated. The majority length of reads fell in the range of 21–24 nt, with 24 nt being the most abundant group of small RNAs, in both clean reads and mapped reads, which is consistent with previous reports of other plants including* Arabidopsis* and radish.

All of the clean reads were aligned to Unigenes with a very stringent parameter, and only 1,630,672 and 1,374,966 clean reads met that standard. We tried to annotate sRNAs after being aligned against several sRNA databases, found that rRNA group was the most abundant type, and showed its necessity in basic biochemistry process (Table S2).

### 3.5. Identification of Conserved and Novel miRNAs

Usually, conserved miRNAs identification relies on information in miRBase, yet there is no information about* A. palmatum* stored in miRBase to date. Thus, we used miRDeep2 software for identification of conserved and novel miRNAs. Finally, we found 67 potential miRNAs, and 46 miRNAs were classified into conserved miRNAs for their mature and precursor sequences shared similarity with other plant miRNAs in miRBase. Other 21 miRNAs were predicted from potential precursor sequences and secondary structure and classified as novel miRNAs. To compare miRNA expression difference, statics analysis was performed between two samples. Seventeen miRNAs were found upregulated in the YF sample, and three were downregulated miRNAs (Table S3). The secondary structure of miRNAs was verified and showed in Supplemental Material, which would help to elucidate the function of these new discovered miRNAs and support phylogenetic analysis among other plant miRNAs. This is the first report of miRNAs in* A. palmatum*, providing a valuable reference resource for further study on the miRNAs regulation on gene expression and differentiation.

### 3.6. Target and Annotation of miRNAs

To understand the function and the pathway that miRNAs take part in, potential target for each miRNA was predicted using bioinformatics method and following matching roles. In total, 102 Unigenes were found to be potential target of miRNAs. Furthermore, 57 potential target Unigenes were annotated with several protein databases. According to COG annotation, most of them were associated with translation, ribosomal structure, and biogenesis. However, a total of 103 pairs of miRNA-Unigene regulation relationship were constructed, including 44 pairs functioned in* cis*-regulation way. Compared to 427 mature miRNAs of* Arabidopsis thaliana* in miRBase (version 21) [27], only a small part of miRNAs was found in* A. palmatum*, which may cause less potential* cis*-regulation relationship that was found (Table S4).

### 3.7. Analysis of DEGs Related to Photosynthesis

Abundant DEGs were detected in photosynthesis related pathways, particularly photosynthesis pathway (ko00195) and antenna proteins (ko00196) (Figure S2, Figure S3). According to KEGG analysis results, expressions of all these DEGs were slashed in the YF sample, three in Photosystem II, one in Photosystem I, one in Cytochrome b6/f complex, three in F-type ATPase, and two in light-harvesting chlorophyll protein complex (LHC). Expressions of some component showed no significant difference in photosynthetic electron transport, Allophycocyanin (AP), Phycocyanin (PC)/Phycoerythrocyanin (PEC), and Phycoerythrin (PE). That revealed that more photosynthesis pigment genes transcripted and produced more pigments to chapter electrons in shade condition. Apa-miR7486 was found significantly upregulated while its target Maple.35372 was significantly downregulated in YF leaves. That indicates when the leaves exposed to the full-sunlight condition, the expression of gene Apa-miR7486 rose and then* cis*-regulated the gene Maple.35372 in order to adjust to high-density light condition.

### 3.8. Analysis of Biosynthesis Pathway of Flavonoids and Anthocyanins

**Figure 4:**
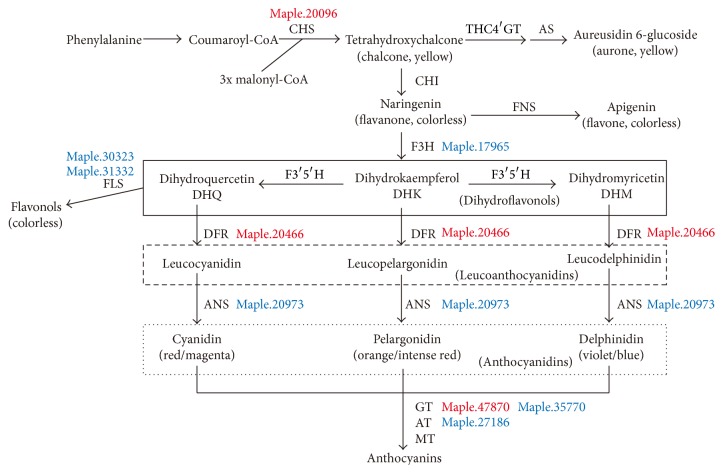
DEGs related to synthesis pathway of chalcone and anthocyanin. CHS: chalcone synthase; CHI: chalcone isomerase; F3H: flavonone 3-hydroxylase; F3′H: flavonoid 3′-hydroxylase; F3′5′H: flavonoid 3′,5′-hydroxylase; DFR: dihydroflavonol 4-reductase; ANS: anthocyanidin synthase; FLS: flavonol synthase; FNS: flavone synthase; THC4′GT: tetrahydroxychalcone 4′-glucosyltransferase; AS: aureusidin synthase; GT: glucosyltransferase; AT: acyltransferase; and MT: methyltransferase. The DEGs in red and blue indicate them being upregulated and downregulated, respectively.

Flavonoids were distributed extensively in plants as a group of representative plant secondary products, accounting for approximately 20% of the total carbon flux. Flavonoids are classified into several subfamilies including flavonol, flavone, flavanone, flavan-3-ol, isoflavone, and anthocyanidin according to the structure [28]. As previously described, anthocyanin content changes in shade condition. Such phenomenon should be a result of gene differentiation in anthocyanin synthesis pathway. In order to find the exact important gene in this process, we studied all DEGs that participate in anthocyanin pathway. In terms of* Arabidopsis*, a group of flavonoid biosynthetic genes including acetyl-CoA carboxylase (*ACC*) gene were induced upon UV-B stress [29]. However, the analogical* ACC* Unigene in* A. palmatum* was detected to be decreased in YF leaves. Chalcone synthase (CHS) is the first rate-limiting enzyme in the biosynthesis of all flavonoids, which catalyzes the formation of naringenin chalcone from p-coumaroyl-CoA and three molecules of malonyl-CoA [30]. We found that Maple.20096 which was a CHS gene increased 1.7 times in YF leaves compared to in GS leaves. It indicated flavonoids synthase activity was much higher in high-density light which produced more yellow chalcone. Flavonol synthase (FLS) contributes to the biosynthesis of flavonols branching from the main trunk route to the anthocyanin formation [31]. Dihydroflavonol reductase (DFR) is a rate-limiting enzyme leading to anthocyanin and proanthocyanidin, which catalyzes competing the substrate, dihydroflavonol with FLS [32]. The expression of DFR gene (Maple.20466) was 2.04 times in full-light condition, while several FLS genes decreased (Table 3, Figure 4). It is possible because more colorful anthocyanins, yet less colorless flavonols, were produced from chalcone, which may cause the leaves of* A. palmatum* to turn to bright yellow under full-light condition. A previous study showed that autumn leaf redness was inducible and closely linked to photooxidative stress, while anthocyanins were not found associated with antioxidant capacity enhancement in red leaves of either species, when exposed to high light [1]. This study supported that anthocyanin and flavonoids were induced by high light treatment, whereas the potential mechanism of anthocyanin and flavonoids was still unclear.

### 3.9. Analysis of Transcription Factors Related to Abiotic Stress

Transcription factors (TFs) play an important role in regulating plant development and stress responses by temporarily and spatially influencing the transcription of their target genes [33]. In this study, we identified numerous DEGs related to transcription factors, revealing a large quantity of transcription factors participated in this shade condition. To be specific, the AP2/ERF family is a large family of plant-specific transcription factors that encode a well-conserved DNA-binding domain and play an important role in abiotic stress responses [34]. In previous study, 11 Unigenes were found upregulated in yellow mutants than in green ones. However, we found among 7 DEGs that encode AP2/ERF TFs in this study 6 DEGs downregulated and 1 upregulated DEG in YF leaves. The WRKY TF family is involved in the regulation of plant developmental processes and biotic and abiotic stress responses, which can recognize and bind TTGAC(C/T) W-box* cis*-elements [35, 36]. Seven WRKY TF genes were found upregulated in GS leaves, indicating more WRKY genes were transcripted in* A. palmatum* to regulate protein network in shade condition.

The heat transcription factors (HSFs) are also important members among transcription factors, which mainly reprogram gene expression to repair protein damage through elevated synthesis of molecular chaperones and proteases [37, 38]. Five HSF genes were found in DEGs; all of them were significantly upregulated in YF leaves. After high-density light, regional heat increased in leaf cells and induced abiotic stress. Consequently, HSF genes level would dramatically increase, regulating targets such as HSPs to protect well-functioned cell structure and efficient metabolism. As another important member in heat-resistance pathway, HSP90 is a highly conserved and essential molecular chaperone involved in maturation and activation of signaling proteins in eukaryotes and is found in different cellular compartments of plants. For example, the* Arabidopsis thaliana* genome encodes four cytosolic (HSP90.1, HSP90.2, HSP90.3, and HSP90.4), one chloroplast-localized (HSP90.5), one mitochondria-localized (HSP90.6), and one endoplasmic reticulum-localized (HSP90.7) HSP90 [39]. Amazingly, a total of 23 HSP genes were found significantly upregulated in YF leaves. According to KEGG annotation, HSP90 protein was significantly upregulated. In summary, higher level of stress induced transcription factors such as AP2/ERF, MYB, and HSF proteins bring higher disease resistance yet slower growth rate.

## Supplementary Material

Fig S1. Saturation Curve of high-throughput sequencing.
Fig S2. KEGG annotation of DEGs in photosynthesis pathway.
Fig S3. KEGG annotation of DEGs related to antenna proteins.

## Figures and Tables

**Table 1:** Physiological index of *A. palmatum*.

Sample	YF	GS
Height (cm)	15.66 ± 0.81	13.50 ± 0.50
Shoot diameter (mm)	1.83 ± 0.10	1.59 ± 0.13
Root vigority	0.0645 ± 0.0017	0.0468 ± 0.0037
Overground biomass (g)	0.591 ± 0.05	0.445 ± 0.07
Underground biomass (g)	0.258 ± 0.04	0.144 ± 0.03
Leaf area (mm^2^)	408.83 ± 115.56	767.10 ± 27.77
Fv/Fm	0.730 ± 0.072	0.819 ± 0.031
Chlorophyll a (mg g^−1^ FW)	0.063 ± 0.017	0.702 ± 0.051
Chlorophyll b (mg g^−1^ FW)	0.007 ± 0.003	0.202 ± 0.019
Carotenoids (mg g^−1^ FW)	0.062 ± 0.005	0.128 ± 0.007
Anthocyanin (*μ*mol g^−1^ FW)	143.30 ± 17.64	43.04 ± 3.17

**Table 2:** Statistics of total clean tags mapping to *A. palmatum* Unigenes.

Alignment	GF reads	GF ratio	YS reads	YS ratio
Total	8984764	100.00%	9592565	100.00%
Mapped	7570105	84.25%	7876146	82.11%
Unique mapped	7088031	93.63%	7433537	94.38%
Multimapped	482074	6.37%	442609	5.62%

**Table 3:** Information of DEGs that responded to UV light or related to flavonoids and anthocyanins pathway.

DEG	Log2FC	Regulation	Annotation
Maple.33541	−3.31	Down	Electron carrier activity (GO:0009055), response to red or far red light (GO:0009639)
Maple.20973	−3.15	Down	Leucocyanidin oxygenase activity (GO:0050589)
Maple.44599	−2.77	Down	UDP-glucuronosyltransferase
Maple.31332	−2.76	Down	FLS2_ARATH LRR receptor-like serine/threonine-protein kinase FLS2
Maple.29413	−2.28	Down	Flavonol biosynthetic process (GO:0051555)
Maple.26813	−2.24	Down	Anthocyanin accumulation in tissues in response to UV light (GO:0043481)
Maple.6996	−2.20	Down	Anthocyanin accumulation in tissues in response to UV light (GO:0043481)
Maple.22770	−2.04	Down	UDP-glucose 6-dehydrogenase activity (GO:0003979)
Maple.30323	−2.04	Down	FLS2_ARATH LRR receptor-like serine/threonine-protein kinase FLS2
Maple.31217	−1.98	Down	Isoflavone 2′-hydroxylase activity (GO:0033773)
Maple.27186	−1.89	Down	Anthocyanin 5-aromatic acyltransferase
Maple.27883	−1.85	Down	F-box and wd40 domain protein
Maple.36436	−1.83	Down	FLS2_ARATH LRR receptor-like serine/threonine-protein kinase FLS2
Maple.35770	−1.79	Down	Regulation of anthocyanin catabolic process (GO:1900000)
Maple.24657	−1.75	Down	UDP-glucose:hexose-1-phosphate uridylyltransferase activity (GO:0008108)
Maple.29919	−1.73	Down	UDP-glucuronate decarboxylase activity (GO:0048040)
Maple.14258	−1.72	Down	Anthocyanin accumulation in tissues in response to UV light (GO:0043481)
Maple.17965	−1.59	Down	F3H naringenin,2-oxoglutarate 3-dioxygenase
Maple.1686	−1.57	Down	UG731_ARATH UDP-glucosyltransferase UGT73B1
Maple.34135	−1.57	Down	Anthocyanin accumulation in tissues in response to UV light (GO:0043481)
Maple.22433	−1.55	Down	Anthocyanin accumulation in tissues in response to UV light (GO:0043481)
Maple.20096	1.70	Up	Regulation of anthocyanin biosynthetic process (GO:0031540); CHSY_BETPN chalcone synthase
Maple.20466	2.04	Up	DFRA_MALDO bifunctional dihydroflavonol 4-reductase/flavanone 4-reductase
Maple.29395	2.45	Up	Response to UV-B (GO:0010224)
Maple.17973	2.63	Up	Biological process: regulation of photomorphogenesis (GO:0010099), response to UV-B (GO:0010224)
Maple.47870	3.55	Up	UDP-glucosyltransferase

## Abbreviations

COG: Clusters of Orthologous Groups of protein
DEGs: Differential Expressed Genes
GF: Green leaves sample under full-sunlight condition
GO: Gene ontology
HSP90: Heat Shock Protein 90
KEGG: Kyoto Encyclopedia of Genes and Genomes
NR: Nonredundant protein database
NT: Nonredundant nucleotide database
SSRs: Simple Sequence Repeats
TF: Transcription factor
TrEMBL: Computer-annotated supplement to Swiss-Prot database
YS: Yellow-leaves sample under shade condition.
