# Supplementary material for: Gene Expressing and sRNA Sequencing Show That Gene Differentiation Associates with a Yellow Acer palmatum Mutant Leaf in Different Light Conditions
Source: Biomed Res Int. 2015 Dec 15;2015:843470. doi: 10.1155/2015/843470 (PMC4692996; doi:10.1155/2015/843470)
Supplement: Supplementary file 1 — Fig S1. Saturation Curve of high-throughput sequencing. Fig S2. KEGG annotation of DEGs in photosynthesis pathway. Fig S3. KEGG annotation of DEGs related to antenna proteins. [file 843470.f1.zip › SupMaterial.pdf]

Provisional ID : Maple_Unigene_BMK.1046_1365
Score total : 39.4
Score for star read(s) : 3.9
Score for read counts : 33.3
Score for mfe : -0.7
Score for randfold : 0
Score for cons. seed : 3
Total read count : 77
Mature read count : 71
Loop read count : 3
Star read count : 3

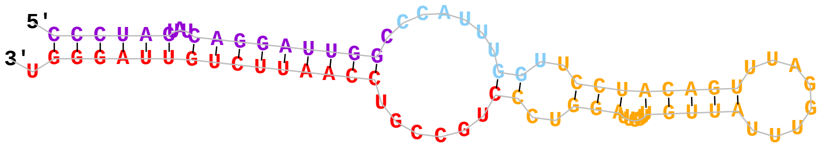

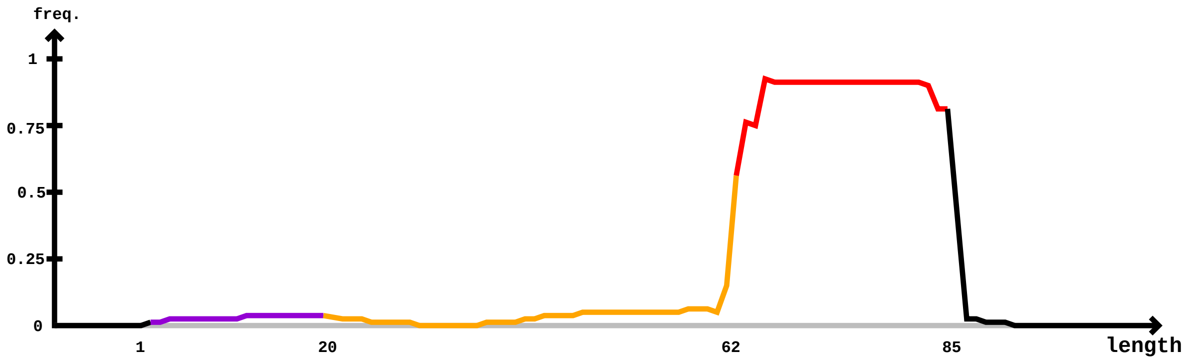

Star Mature

```
5'- uuuggucccccuaguaucaggauuggcccauuugguuccuacaguuuagguuuauuguugguuaggucccugccguccaauucuguuaggguuuuuaaaaauucuguuaaugg  -3'  obs
    uuuggucccccuaguaucaggauuggcccauuugguuccuacaguuuagguuuauuguugguuaggucccugccguccaauucuguuaggguuuuuaaaaauucuguuaaugg       exp
    (((((...((((((...(((((((((.......((..((((((((........))))).....)))..))......)))))))))))))))...)))))..............  reads  mm  sample
    ..........cuaguaucaggauuggcccaC..................................................................................  1  1  S01
    ..................aggauuggcccauuuggu.............................................................................  1  0  S01
    ...........................................guuuagguuuauuguuAguuaggu..............................................  1  1  S01
    ....................................................................ccuUccguccaauucuguuagggu.....................  2  1  S01
    ....................................................................ccuUccguccaauucuguuaggguu....................  1  1  S01
    .....................................................................cuUccguccaauucuguuagg.......................  1  1  S01
    .....................................................................cugccguccaauucuguuagggA.....................  1  1  S01
    .....................................................................cuUccguccaauucuguuagggu.....................  4  1  S01
    .....................................................................cugccgCccaauucuguuaggguu....................  1  1  S01
    .....................................................................cugccguccaauucuguuaggguu....................  4  0  S01
    .....................................................................cuUccguccaauucuguuaggguu....................  3  1  S01
    .....................................................................cuAccguccaauucuguuaggguuuu..................  1  1  S01
    ......................................................................ugcUguccaauucuguuagggu.....................  1  1  S01
    ......................................................................ugccguccaauucuguuagggC.....................  1  1  S01
    ......................................................................ugccguccaauucuguuaggguu....................  5  0  S01
    ......................................................................ugccguccaauucugGuaggguu....................  1  1  S01
    ......................................................................ugccguccaauucuguuagAguu....................  1  1  S01
    ........................................................................ccguccaauucuguuagg.......................  2  0  S01
    ........................................................................ccAuccaauucuguuagg.......................  1  1  S01
    ........................................................................ccguccaCuucuguuagggu.....................  1  1  S01
    ........................................................................ccguccaauucuguuagggu.....................  2  0  S01
    ........................................................................ccAuccaauucuguuaggguu....................  1  1  S01
    ........................................................................ccguccaauucuguuaggguu....................  1  0  S01
    ........................................................................ccguccaauucuguuagAguu....................  1  1  S01

    ........cccuaguUucaggauuggc......................................................................................  1  1  S02
    ...............................................agguuuauCguugguuaggucccu..........................................  1  1  S02
    .................................................guuuauuguugguuaAgucccugc........................................  1  1  S02
    .....................................................auuguuAguuaggucccugccguc....................................  1  1  S02
    ................................................................ggucccuAccguccaauucuguuag........................  1  1  S02
    ....................................................................ccugccguccaauucuguuagAgu.....................  1  1  S02
    ....................................................................ccugccguccaauucuguuagggG.....................  1  1  S02
    ....................................................................ccugccguccaauucuguuagggu.....................  1  0  S02
```

```
        Star                                                         Mature
uuugguccccccuaguaucaggauuggcccauuugguuccuacaguuuagguuuauuguugguuagguccccugccguccaauucuguuaggguuuuuaaaaauucuguuaaugg
....................................................................Acugccguccaauucuguuagggu.....................   2   1   S02
.....................................................................cuUccguccaauucuguuagg.......................   1   1   S02
.....................................................................cuUccguccaauucuguuagggu.....................   10  1   S02
.....................................................................cugccguccaauucuguuagAgu.....................   1   1   S02
.....................................................................cugccguccaauucuguuaggguu....................   1   0   S02
.....................................................................cugccguccaauucuguuaggguC....................   1   1   S02
.....................................................................Augccguccaauucuguuaggguu....................   2   1   S02
.....................................................................cugccguccaauucuguuagAguu....................   1   1   S02
.....................................................................cugccguccaauucuAuuaggguu....................   1   1   S02
......................................................................uUccguccaauucuguuagggu.....................   1   1   S02
......................................................................ugccguccaauucuguuaggguu....................   3   0   S02
......................................................................ugccguccaauucuAuuaggguu....................   1   1   S02
......................................................................ugccguccaauucuguuGggguu....................   1   1   S02
......................................................................ugccguccaauucuguuagAguu....................   1   1   S02
........................................................................ccguccaauucuguuagg.......................   2   0   S02
........................................................................ccguccaauucuguuagggu.....................   1   0   S02
........................................................................ccguccaauucuguuaggguu....................   1   0   S02
........................................................................ccguccaauucuguuagAguu....................   1   1   S02
.............................................................................caauucuguuaggguuuuCaa...............   1   1   S02
```

Provisional ID	:	Maple_Unigene_BMK.1632_2451
Score total	:	60.5
Score for star read(s)	:	3.9
Score for read counts	:	54.7
Score for mfe	:	2.5
Score for randfold	:	0
Score for cons. seed	:	-0.6
Total read count	:	119
Mature read count	:	69
Loop read count	:	1
Star read count	:	49

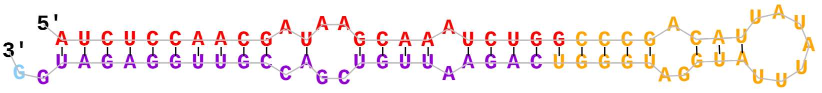

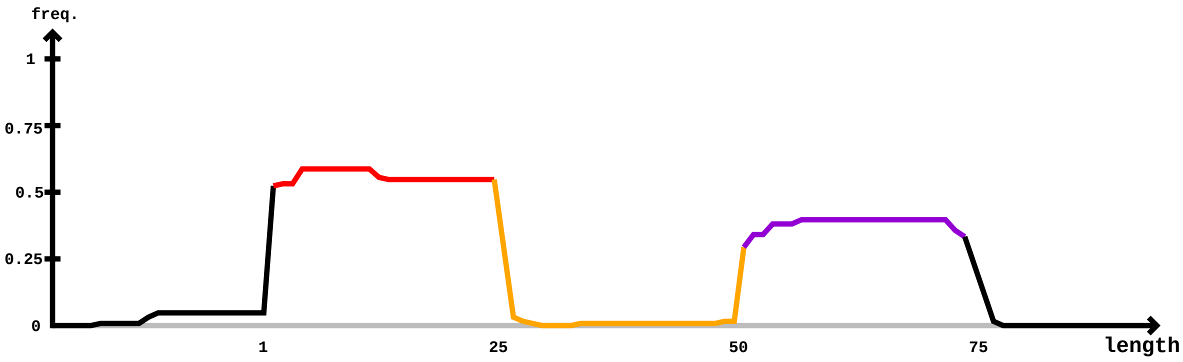


```
                                      Mature                                          Star
5'- ccgaaaaauuguaaacccucuaucuccaacgauaagcaaaucuggcccgacauuauauuuauggaugggucagaauugucgaccguuggagauggagggucuuacaauuuuua   -3'   obs
    ccgaaaaauuguaaacccucuaucuccaacgauaagcaaaucuggcccgacauuauauuuauggaugggucagaauugucgaccguuggagauggagggucuuacaauuuuua         exp
    ...((((((((((((((((((((((((((((.(..((((.(((((((((.(((.......)))..))))))))).))))..).))))))))))))))))).))))))))))).   reads   mm   sample
    ...aaaaauuguaaacccucuauc.........................................................................................   1       0    S01
    ........uuguaaacccucuaucuccaacgG.................................................................................   2       1    S01
    ........uuguaaacccucuaucuccaacgU.................................................................................   1       1    S01
    .........uguaaacccucuaucuccaacgGu................................................................................   1       1    S01
    .....................aucuccaacgGuaagcaaaucugg....................................................................   32      1    S01
    ......................ucuccaacgGuaagcaaaucuggc...................................................................   1       1    S01
    ........................uccaacgGuaagcaaaucugg....................................................................   1       1    S01
    ........................uccaacgGuaagcaaaucuggc...................................................................   1       1    S01
    ........................uccaacgGuaagcaaaucuggccc.................................................................   1       1    S01
    .....................................................uauauuuauggaCgggucaga.......................................   1       1    S01
    ....................................................................gucagaauuUucgaccguuggaga.....................   1       1    S01
    ......................................................................cagaauugucgaccguuggagau....................   2       0    S01
    ......................................................................cagaauugucgaccgAuggagaug...................   1       1    S01
    ......................................................................cagaauugucgaccguuggagaug...................   14      0    S01
    ......................................................................cagaauugucgGccguuggagaug...................   1       1    S01
    ......................................................................cagaauugucgaccguuggagaugg..................   3       0    S01
    .......................................................................agaauugucgaccgCuggagaugg..................   1       1    S01
    .......................................................................agaauugucgaccguuggagaugg..................   2       0    S01
    .........................................................................aauugucgaccguuggagaugga.................   1       0    S01
    ..........................................................................auugucgaccguuggaga.....................   1       0    S01
    ............................................................................ugucgaccguuggagaug...................   1       0    S01

    .........uguaaacccucuaucuccaacgG.................................................................................   1       1    S02
    .....................aucuccaacgGuaagcaaaucugg....................................................................   28      1    S02
    ........................uccaacgGuaagcaaaucugg....................................................................   2       1    S02
    ........................uccaacgGuaagcaaaucuggcc..................................................................   2       1    S02
    ........................uccaacgGuaagcaaaucuggcccg................................................................   1       1    S02
    ......................................................................cagaauugucgaccguuggagaug...................   8       0    S02
    ......................................................................cagaauugucgaccguuggagauC...................   1       1    S02
    ......................................................................cagGauugucgaccguuggagaug...................   1       1    S02
    ......................................................................cagaauugucgaccguuggagaCg...................   1       1    S02
    ......................................................................cagaauugucgaccguuggagaugg..................   3       0    S02
    .......................................................................agaauugucgaccguuggaga.....................   1       0    S02
```

```
                     Mature                                              Star
ccgaaaaauuguaaacccucuaucuccaacgauaagcaaaucuggcccgacauuauauuuauggauggguc agaauugucgaccguuggagauggagggucuuacaauuuuua
.......................................................................agaauugucgaccguuggagau....................   1   0   S02
.......................................................................agaauugucgaccguuggagaugg..................   1   0   S02
.........................................................................aauugucgaccguuggaga.....................   1   0   S02
.........................................................................aauugucgaccguuggagU.....................   1   1   S02
.........................................................................aauugucgaccguuggagauggag................   2   0   S02
............................................................................uguUgaccguuggagaugg..................   1   1   S02
```

Provisional ID	:	Maple_Unigene_BMK.2334_3772
Score total	:	0.1
Score for star read(s)	:	-1.3
Score for read counts	:	0
Score for mfe	:	2
Score for randfold	:	0
Score for cons. seed	:	-0.6
Total read count	:	92
Mature read count	:	86
Loop read count	:	0
Star read count	:	6

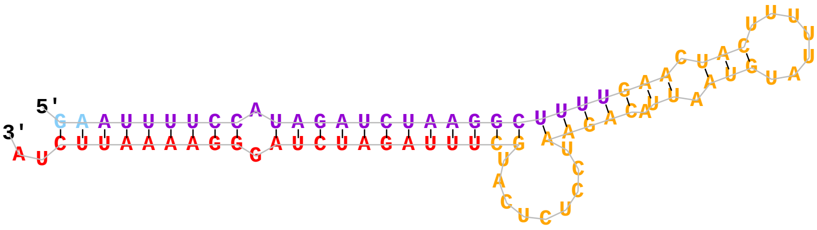

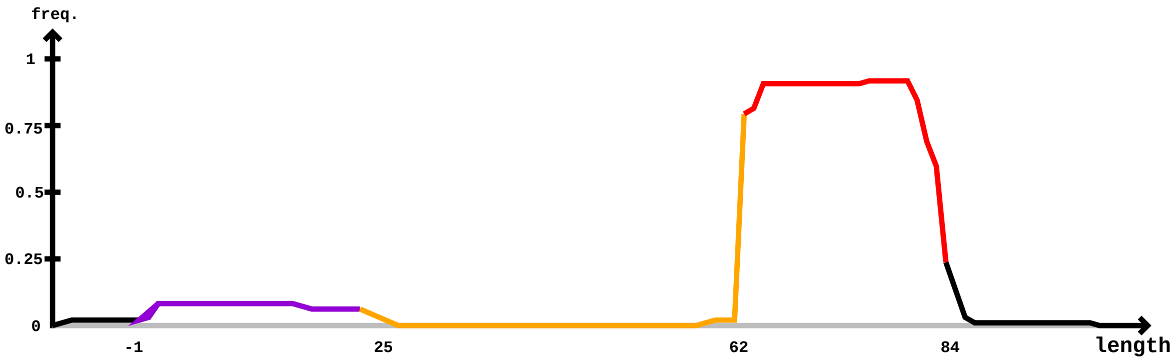


```
           Star                                          Mature
5'- uucccuagaauuuuccauagaucuaaggcuuuugaacuacuuuuuauguaauuacagaauccucucaugcuuuagaucuagggaaaauucuagggaaauucaugucuauacu  -3'   obs
    uucccuagaauuuuccauagaucuaaggcuuuugaacuacuuuuuauguaauuacagaauccucucaugcuuuagaucuagggaaaauucuagggaaauucaugucuauacu        exp
    ((((((((((((((.(((((((((((((((((((.(((.......))).)).))))).........))))))))))).)))))))))))))))...............   reads   mm   sample
    uucccuagaauuuuccauagaucua.......................................................................................   1   0   S01
    .........auuuuccauagaucuaaggcuuu................................................................................   1   0   S01
    .........auuuuccauagaucuaaggcuuuu...............................................................................   1   0   S01
    ...................................................................ugcGuuagaucuagggaaaauucu......................   1   1   S01
    ......................................................................uuuagaucuagggaaaau........................   5   0   S01
    ......................................................................uuuagaucuagggaGaauu.......................   1   1   S01
    ......................................................................uuuagaucuagggaaaUuu.......................   1   1   S01
    ......................................................................uuuagaucuagggaaaauu.......................   6   0   S01
    ......................................................................uuuagaucuagggaaaauuU......................   3   1   S01
    ......................................................................uuuagaucuagggaaaauuc......................   1   0   S01
    ......................................................................uuuagaucuagggaaaauucu.....................   5   0   S01
    ......................................................................uuuagaucuagggaaaauucC.....................   2   1   S01
    ......................................................................uuuagaucuagggaaaauuUu.....................   2   1   S01
    ......................................................................uuuagaucuagggaaaauucua....................   9   0   S01
    ......................................................................uuuagaucuagggaaaauucuag...................   1   0   S01
    .......................................................................Auagaucuagggaaaauucu.....................   1   1   S01
    .......................................................................uuagaucuagggaaaauucua....................   1   0   S01
    ........................................................................uagaucuagggaaaauucu.....................   6   0   S01
    ...................................................................................aaaauucuagggaaaCucaugucu.....   1   1   S01

    uucccuagaauuuuccauagaucu........................................................................................   1   0   S02
    ........aauuuuccauagaucuaaggcuuu................................................................................   1   0   S02
    .........auuuuccauagaucuaaggcuuuC...............................................................................   1   1   S02
    .........auuuuccauagaucuaaggcuuuu...............................................................................   2   0   S02
    ..................................................................augcuuuagaucuagggaaaauucu.....................   1   0   S02
    ......................................................................uuuagaucuagggaaaau........................   1   0   S02
    ......................................................................uuuagaucuagggaaaUu........................   1   1   S02
    ......................................................................uuuagaucuagggaaaauu.......................   6   0   S02
    ......................................................................uuuagaucuagggaaaUuu.......................   1   1   S02
    ......................................................................uuuagaucuagggaaaauuc......................   1   0   S02
    ......................................................................uuuagaucuagggaaaauuU......................   3   1   S02
    ......................................................................uuuagaucuagggaaaauucA.....................   1   1   S02
    ......................................................................uuuagaucuagggaaaauucC.....................   2   1   S02
```

Star **Mature**

uucccuagaauuuuccauagaucuaaggcuuuugaacuacuuuuuauguaauuacagaauccucucaugcuuuagaucuagggaaaauucuagggaaauucaugucuauacu			
....uuuGgaucuagggaaaauucu.....................	1	1	S02
....uuuagaucuagggaaaauuUu.....................	3	1	S02
....uuuagaucuagggaaaauucu.....................	8	0	S02
....uuuagaucuagggaaaauucua....................	5	0	S02
....uuuagaucuagggaaaauucuU....................	2	1	S02
....uuuagaucuagggaaaauucuaU...................	1	1	S02
....uuuagaucuagggaaaauucuag...................	1	0	S02
....uuuagaucuagggaaaauucuagA..................	1	1	S02
....uuuagaucuagggaaaauucuagg..................	1	0	S02
....uagaucuagggaaaauuU......................	1	1	S02
....uagaucuagggaaaauucu.....................	2	0	S02

Provisional ID	:	Maple_Unigene_BMK.3640_6661
Score total	:	792.1
Score for star read(s)	:	3.9
Score for read counts	:	788.8
Score for mfe	:	0
Score for randfold	:	0
Score for cons. seed	:	-0.6
Total read count	:	1559
Mature read count	:	1553
Loop read count	:	0
Star read count	:	6

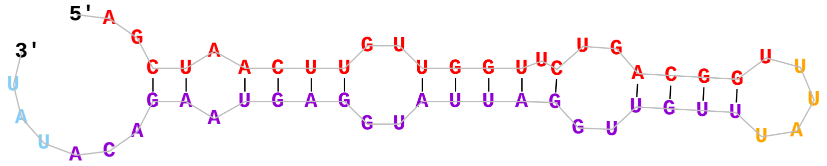

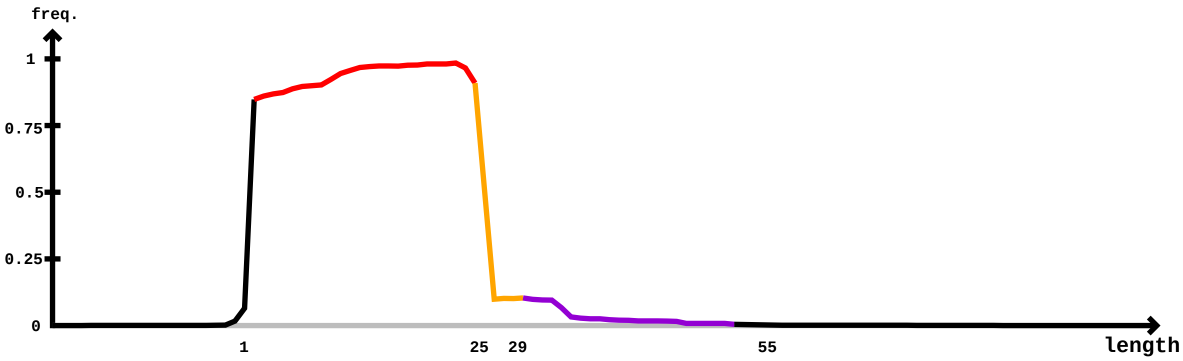

Mature **Star**

```
5'- gacugcgugacacaccuauagcuaacuuguugguucugacgguuuauuuguuggauuauggaguaagacauaugggaaaucucggcccacacccccggccacaaaaagaugca  -3'  obs
    gacugcgugacacaccuauagcuaacuuguugguucugacgguuuauuuguuggauuauggaguaagacauaugggaaaucucggcccacacccccggccacaaaaagaugca       exp
    ....((((......((((((.((.((((..((((.(..((((.....))))..))))).)))).))...)))))).......((((.........))))........)))).  reads  mm  sample
    ..cugcgugacacacUuauagc...........................................................................................  1  1  S01
    .................auagcCaacuuguugguucugac.........................................................................  1  1  S01
    .................auagcuaacuuguugguucugacA........................................................................  1  1  S01
    .................auagcCaacuuguugguucugacg........................................................................  4  1  S01
    .................auagcCaacuuguugguucugacgg.......................................................................  1  1  S01
    ..................uagcuaacuuguugguucugacgA.......................................................................  1  1  S01
    ..................uagcuaacuuguugguucugacgg.......................................................................  2  0  S01
    ..................uagcCaacuuguugguucugacgg.......................................................................  21  1  S01
    ..................uagcuaacuuguugguucugacgU.......................................................................  1  1  S01
    ..................uagcuaacuuguugguucugacggu......................................................................  2  0  S01
    ..................uagcCaacuuguugguucugacggu......................................................................  7  1  S01
    ...................agcCaacuuguugguucugacg........................................................................  2  1  S01
    ...................agcCaacuuguugguucugacgg.......................................................................  8  1  S01
    ...................agcCaacuuguugguucugacggu......................................................................  619  1  S01
    ...................agcuaacuuguuggAucugacggu......................................................................  1  1  S01
    ...................aAcuaacuuguugguucugacggu......................................................................  3  1  S01
    ...................Ggcuaacuuguugguucugacggu......................................................................  1  1  S01
    ...................agcuaacuCguugguucugacggu......................................................................  1  1  S01
    ...................agcuaacuuguugguucugacggC......................................................................  8  1  S01
    ...................agcuaacuuguuUguucugacggu......................................................................  1  1  S01
    ...................agcuaacuuguugguucugacggu......................................................................  113  0  S01
    ...................agcAaacuuguugguucugacggu......................................................................  4  1  S01
    ...................agcuaacuuguugguucugaUggu......................................................................  1  1  S01
    ...................agcuaacuugGugguucugacggu......................................................................  1  1  S01
    ...................agcuaacuuguugguucugacAgu......................................................................  15  1  S01
    ...................agcuaacuugCugguucugacggu......................................................................  2  1  S01
    ...................agcuaacuuguugguucugacggA......................................................................  1  1  S01
    ...................agcGaacuuguugguucugacggu......................................................................  1  1  S01
    ...................agcuaacuuguugguucugacggG......................................................................  2  1  S01
    ....................gcCaacuuguugguucugacggu......................................................................  4  1  S01
    ....................Acuaacuuguugguucugacgguu.....................................................................  4  1  S01
    ....................gcuaacuuguugguucugacgguCu....................................................................  1  1  S01
    .....................cuaacuuguugguucugacgg.......................................................................  1  0  S01
```

Mature Star

gacugcgugacacaccuauagcuaacuuguugguucugacgguuuauuuguuggauuauggaguaagacauaugggaaaucucggcccacacccccggccacaaaaagaugca			
.....................cCaacuuguugguucugacgg..	1	1	S01
.....................cCaacuuguugguucugacggu...	2	1	S01
.....................cuaacuuguugguucugacgguCu...	1	1	S01
......................uaacuuguugguucugacggu...	1	0	S01
......................Caacuuguugguucugacggu...	3	1	S01
.......................aacuuguugguucugacA...	1	1	S01
.......................aaUuuguugguucugacgg..	1	1	S01
.......................aacuuguugguucugacggC...	1	1	S01
.......................aaUuuguugguucugacggu...	2	1	S01
.......................aacuuguugguucugacggu...	3	0	S01
.......................aacuuguuAguucugacggu...	2	1	S01
.......................aacuuguugguucugacgguC..	1	1	S01
........................acuuguugguucugacAgu...	1	1	S01
........................acuuguugguucugacggC...	1	1	S01
........................acuuguugguucugacggu...	2	0	S01
........................acuuguugguucugacgguCuauu..	2	1	S01
..........................uuguugguucugacgguCuauuug..	1	1	S01
...........................uguugguucugacgguuuauuugu...	1	0	S01
...........................uguugguucugacgguCuauuugu...	17	1	S01
...........................uguugguucugacgguCuauuuguu..	1	1	S01
............................guugguucugacgguCuauuugu...	1	1	S01
............................guugguucugacgguCuauuuguu..	18	1	S01
.............................uugguucugacgguCuauuugu...	1	1	S01
.............................uugguucugacgguCuauuuguu..	6	1	S01
.............................uugguucugacgguCuauuuguug...	2	1	S01
..............................ugguucugacgguCuauuugu...	3	1	S01
..............................ugguucugacgguCuauuuguu..	2	1	S01
..............................ugguucugacgguCuauuuguug...	2	1	S01
..............................ugguucugacgguCuauuuguugg..	2	1	S01
...............................gguucugacgguCuauuugu...	1	1	S01
...............................gguucugacgguCuauuuguu..	1	1	S01
................................guucugacgguuuauuuAuuggau..	1	1	S01
...................................cugacgguCuauuuguuggauu...	1	1	S01
...................................cugacgguCuauuuguuggauuau...	2	1	S01
.....................................gacgguCuauuuguuggau..	1	1	S01
.....................................gacgguCuauuuguuggauu...	2	1	S01
.....................................gacgguCuauuuguuggauuaugga..	1	1	S01
....gguCuauuuguuggauuauggagu..	4	1	S01
....guCuauuuguuggauuauggagu..	1	1	S01
....auuuguuAgauuauggag...	1	1	S01
....auuuguuggauuauUgaguaagac...	3	1	S01
....uuguuggauuauUgaguaagaca..	1	1	S01
....uuguuggauuauggaguaagacaA...	1	1	S01
....auuauggaguaagacauauAggaa..................................	1	1	S01
....aucucggcUcacacccccg...............	1	1	S01
...............cCauagcuaacuuguugguucuga...	1	1	S02
................uauagUuaacuuguuggu..	1	1	S02
.................auagcuaacuuguugguucugacA...	2	1	S02
.................auaAcuaacuuguugguucugacg...	1	1	S02
.................auagcuaacuuguugguucugacg...	3	0	S02
.................auagcCaacuuguugguucugacg...	10	1	S02
.................auagcCaacuuguugguucugacgg..	1	1	S02
.................auagcuaacuuguugguucugacgU..	1	1	S02
.................auagcuaacuuguugguucugacgg..	1	0	S02
..................uagcCaacuuguugguucugacgg..	28	1	S02
..................uagcuaacuCguugguucugacgg..	1	1	S02
..................uagcuaacuuguugguucugacgg..	10	0	S02
..................uagcuaacuuguugguucugacggu...	4	0	S02
..................uagcCaacuuguugguucugacggu...	6	1	S02
...................agcCaacuuguugguucugac..	5	1	S02
...................agcuaacuuguugguucugacg...	1	0	S02
...................agcCaacuuguugguucugacg...	3	1	S02
...................agcuaacuuguugguucugacgA..	1	1	S02
...................agcuaacuuguugguucugacgg..	2	0	S02
...................agcCaacuuguugguucugacgg..	5	1	S02
...................aAcuaacuuguugguucugacgg..	1	1	S02
...................agcuaacuuguugguucugacgC..	1	1	S02
...................agcuaacuuguugguuUugacggu...	1	1	S02
...................agcuaacuuguCgguucugacggu...	1	1	S02

```
                   Mature                       Star
gacugcgugacacaccuauagcuaacuuguugguucugacgguuuauuuguuggauuauggaguaagacauaugggaaaucucggcccacacccccggccacaaaaagaugca
...................agcuaacuuguugguucugacggu......................................................................    127    0    S02
...................agcuaacuuguugguucugCcggu......................................................................    1      1    S02
...................agcuaacuGguugguucugacggu......................................................................    1      1    S02
...................agcuGacuuguugguucugacggu......................................................................    2      1    S02
...................Ngcuaacuuguugguucugacggu......................................................................    1      1    S02
...................agcuaacuuguugguucugacggC......................................................................    13     1    S02
...................aAcuaacuuguugguucugacggu......................................................................    4      1    S02
...................Ggcuaacuuguugguucugacggu......................................................................    3      1    S02
...................agGuaacuuguugguucugacggu......................................................................    1      1    S02
...................agcuaacuuguugguucugacAgu......................................................................    11     1    S02
...................agcuaacuuguugguucugacggG......................................................................    7      1    S02
...................agcuaacuuguugguucugacggA......................................................................    1      1    S02
...................agcAaacuuguugguucugacggu......................................................................    9      1    S02
...................agcCaacuuguugguucugacggu......................................................................    365    1    S02
...................agcuaacuuguugguucugGcggu......................................................................    2      1    S02
...................agcuaacuuguugguucugacgguu.....................................................................    1      0    S02
....................gcCaacuuguugguucugacg........................................................................    2      1    S02
....................gcCaacuuguugguucugacgg.......................................................................    3      1    S02
....................gcuaacuuguugguucugacAgu......................................................................    1      1    S02
....................gcCaacuuguugguucugacggu......................................................................    7      1    S02
.....................cuaacuuguugguucugacA........................................................................    1      1    S02
.....................cCaacuuguugguucugacgg.......................................................................    1      1    S02
.....................cuaacuuguugguucugacgg.......................................................................    1      0    S02
.....................cAaacuuguugguucugacgg.......................................................................    1      1    S02
.....................cCaacuuguugguucugacggu......................................................................    4      1    S02
.....................cuaacuuguugguucugacAgu......................................................................    1      1    S02
......................Caacuuguugguucugacg........................................................................    1      1    S02
......................Caacuuguugguucugacgg.......................................................................    1      1    S02
......................Caacuuguugguucugacggu......................................................................    3      1    S02
......................uaacuuguugguucugacgguCu....................................................................    1      1    S02
.......................aacuuguugguucugacg........................................................................    1      0    S02
.......................aacuuguugguucugacA........................................................................    1      1    S02
.......................aacuuguugguucugacCg.......................................................................    1      1    S02
.......................aacuuguugguucugacggu......................................................................    5      0    S02
.......................aaUuuguugguucugacggu......................................................................    1      1    S02
.......................aacuuguugguucugacggC......................................................................    1      1    S02
.......................aacuuguugguucugacAgu......................................................................    1      1    S02
.......................aacuuguugAuucugacggu......................................................................    1      1    S02
.......................aacuuguugguucugacgguCuauu.................................................................    1      1    S02
........................acuuguugguucugacgg.......................................................................    1      0    S02
........................acuuguugguucugacggu......................................................................    1      0    S02
........................aUuuguugguucugacggu......................................................................    2      1    S02
........................acuuguugAuucugacggu......................................................................    1      1    S02
........................acuuguugguucugacgguCuauu.................................................................    4      1    S02
.........................cuuguugguucugacAgu......................................................................    1      1    S02
.........................cuuguugguucugacgguCua...................................................................    1      1    S02
.........................cuuguugguucugacgguCuauu.................................................................    3      1    S02
..........................uuguugguucugacgguCuauuu................................................................    4      1    S02
...........................uguugguucugacgguCuauuugu..............................................................    16     1    S02
...........................uguugguucugacgguCuauuuguu.............................................................    1      1    S02
............................guugguucugacgguCuauuugu..............................................................    3      1    S02
............................guugguucugacgguCuauuuguu.............................................................    15     1    S02
............................guuAguucugacgguuuauuuguu.............................................................    1      1    S02
.............................uugguucugacgguCuauuugu..............................................................    1      1    S02
.............................uugguucugacgguCuauuuguu.............................................................    10     1    S02
..............................ugguucugacgguCuauuugu..............................................................    1      1    S02
..............................ugguucugacgguuuauuuguu.............................................................    1      0    S02
..............................ugguucugacgguCuauuuguu.............................................................    2      1    S02
..............................ugguucugacgguCuauuuguug............................................................    3      1    S02
..............................ugguucugacgguCuauuuguugg...........................................................    3      1    S02
...............................gguucugacgguCuauuugu..............................................................    2      1    S02
...............................gguucugacgguCuauuuguu.............................................................    1      1    S02
...............................gguucugacgguCuauuuguug............................................................    1      1    S02
................................guucugacgguCuauuugu..............................................................    2      1    S02
................................guucugacgguCuauuuguu.............................................................    1      1    S02
...................................cugacgguCuauuuguuggauua.......................................................    1      1    S02
...................................cugacgguCuauuuguuggauuau......................................................    2      1    S02
....................................ugacgguCuauuuguuggau.........................................................    1      1    S02
.....................................gacgguCuauuuguuggau.........................................................    3      1    S02
.......................................cgguCuauuuguuggauuauggag..................................................    1      1    S02
```

Mature Star

```
gacugcgugacacaccuauagcuaacuuguugguucugacgguuuauuuguuggauuauggaguaagacauaugggaaaucucggcccacacccccggccacaaaaagaugca
........................................gguCuauuuguuggauuauggagu.................................................	5	1	S02
........................................gguuuauuuguuCgauuauggagu.................................................	1	1	S02
........................................gguuuauuuguuggauuauggagu.................................................	2	0	S02
.........................................guCuauuuguuggauuauggagu.................................................	1	1	S02
.............................................auuuguuggauuauggaguaagac............................................	1	0	S02
.............................................auuuguuAgauuauggaguaagac............................................	1	1	S02
.............................................auuuguuggauuauggaguaUgaca...........................................	1	1	S02
.............................................auuuguuggauuauggaguaagaca...........................................	1	0	S02
...............................................uuguuggauuauggaguaagac............................................	1	0	S02
...............................................uuguuggauuauggaguaGgaca...........................................	1	1	S02
................................................uguuggauuauggaguaagacaGa.........................................	1	1	S02
................................................................aagacauaugggaaaucuUggccc.........................	1	1	S02
```

Provisional ID	:	Maple_Unigene_BMK.5970_10147
Score total	:	0.3
Score for star read(s)	:	-1.3
Score for read counts	:	0
Score for mfe	:	2.2
Score for randfold	:	0
Score for cons. seed	:	-0.6
Total read count	:	55
Mature read count	:	55
Loop read count	:	0
Star read count	:	0

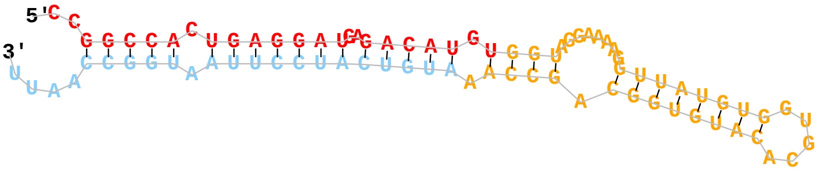

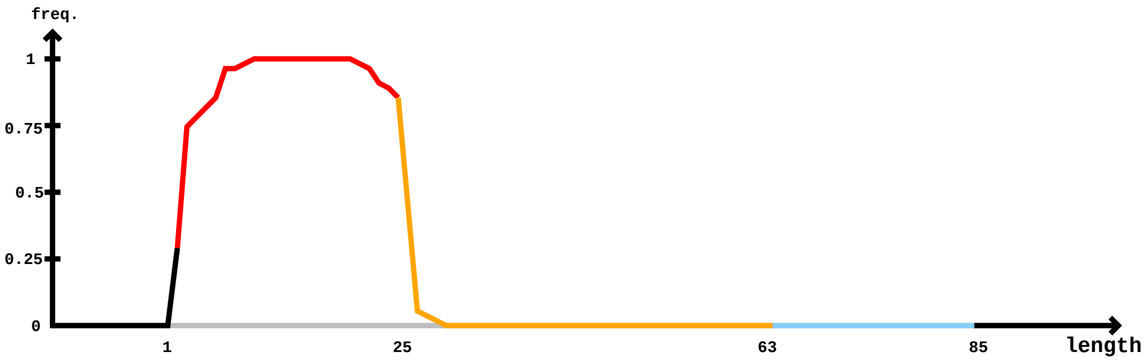

Mature Star

```
5'- gagugguguacccggccacugaggaugagacauguggguaggaaaagguuauguggugcacauguggcagccaaaugucauccuuaaauggccaauugcaccacucagcag -3'   exp
    ((((((((((...(((((.(((((((..(((((.((((........((((((((.....)))))))).)))).)))))))))))).)))))...)))))))))).....   reads   mm   sample
    ...........ccggccacugaggaugagGca..............................................................................   1   1   S01
    ...........ccggccacugaggaugagacaugu...........................................................................   1   0   S01
    ...........ccggUcacugaggaugagacaugu...........................................................................   8   1   S01
    ............cggUcacugaggaugagacaugu...........................................................................   2   1   S01
    ............cggUcacugaggaugagacaugug..........................................................................   1   1   S01
    ............Uggccacugaggaugagacaugug..........................................................................   5   1   S01
    .............ggccacugaggaugagacaugug..........................................................................   1   0   S01
    ...............ccacugaggaugagacaugu...........................................................................   1   0   S01
    ................cacugaggauAagacaugu...........................................................................   2   1   S01
    ................cacugaAgaugagacaugugg.........................................................................   1   1   S01
    ................cacugaggaugagacauguggC........................................................................   1   1   S01
    ..................cugaggauAagacaugug..........................................................................   1   1   S01
    ...................ugaggaugagacaugugguU.......................................................................   1   1   S01

    ...........ccggccacugaggaugagac...............................................................................   1   0   S02
    ...........ccggUcacugaggaugagaca..............................................................................   1   1   S02
    ...........ccggUcacugaggaugagacaugu...........................................................................   4   1   S02
    ............cggccacugaggaugagG................................................................................   1   1   S02
    ............cggUcacugaggaugagacaug............................................................................   1   1   S02
    ............cggUcacugaggaugagacaugu...........................................................................   8   1   S02
    ............Uggccacugaggaugagacaugug..........................................................................   7   1   S02
    .............ggccacugaggaugagacaugA...........................................................................   1   1   S02
    ..............gccacugaAgaugagaca..............................................................................   1   1   S02
    ..............Accacugaggaugagacaugu...........................................................................   1   1   S02
    ...............ccacugaAgaugagacau.............................................................................   1   1   S02
    ................cacugaggauAagacaug............................................................................   1   1   S02
    ................cacugaAgaugagacaugu...........................................................................   1   1   S02
```

Provisional ID	:	Maple_Unigene_BMK.10182_17248
Score total	:	0.9
Score for star read(s)	:	3.9
Score for read counts	:	-4.9
Score for mfe	:	-0.9
Score for randfold	:	0
Score for cons. seed	:	3
Total read count	:	2
Mature read count	:	1
Loop read count	:	0
Star read count	:	1

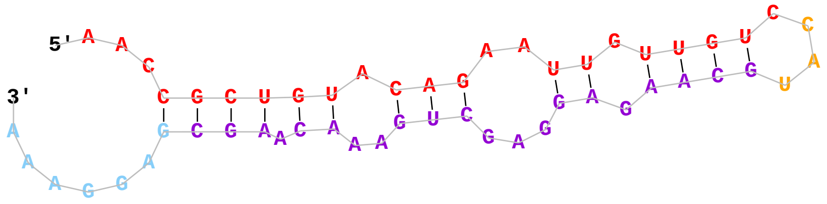

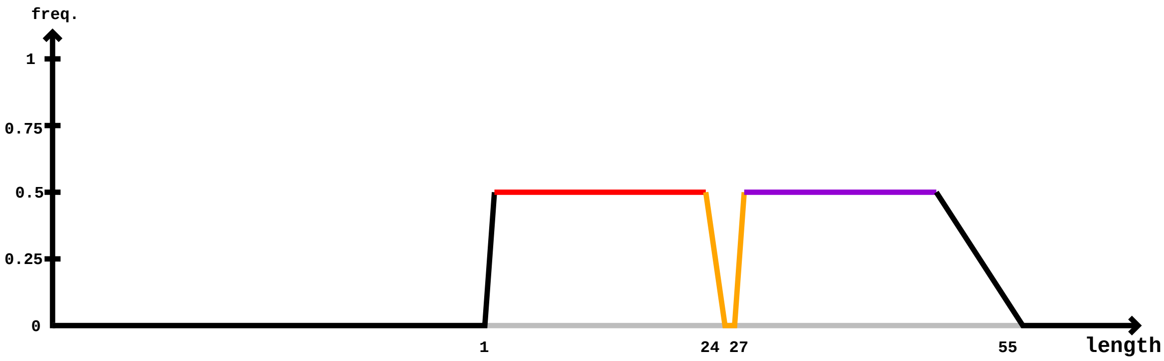


```
                                                Mature               Star
5'- uggaugauuuucuauaucaaauucguaaggacuucccaaagcuuaaccgcuguacagaauuguuguccaugcaagaggagcugaaacaagcgaggaaagcuuuugaagugg   -3'    obs
    uggaugauuuucuauaucaaauucguaaggacuucccaaagcuuaaccgcuguacagaauuguuguccaugcaagaggagcugaaacaagcgaggaaagcuuuugaagugg          exp
    ((((......))))................(((((..(((((((..(((((((.(((..((.((((....)))).))...)))..)).)))).)..))))))).)))))..    reads    mm    sample
    ............................................aaccgcuguacagaauuguuguc............................................    1        0     S01
    ......................................................................gcaagaggagcugaaacaagc....................    1        0     S01
```

Provisional ID	:	Maple_Unigene_BMK.11223_20043
Score total	:	1.1
Score for star read(s)	:	-1.3
Score for read counts	:	0
Score for mfe	:	-0.5
Score for randfold	:	0
Score for cons. seed	:	3
Total read count	:	14
Mature read count	:	14
Loop read count	:	0
Star read count	:	0

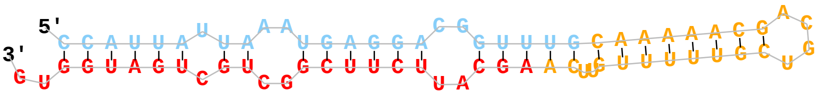

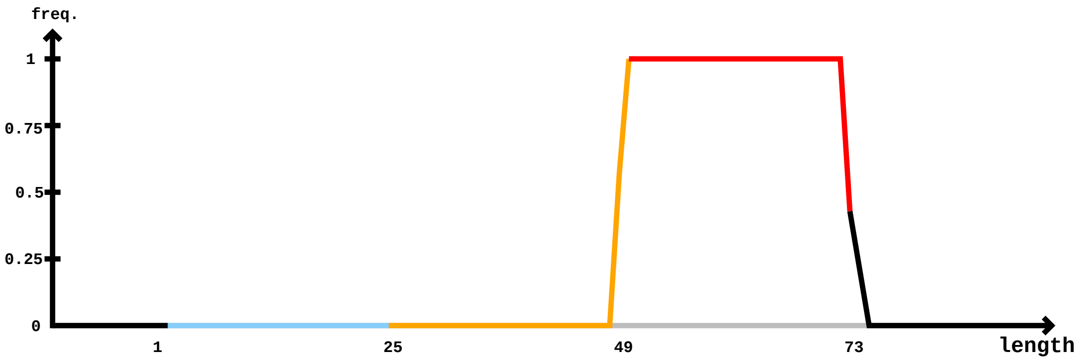


```
                    Star                                                  Mature
5'- auguauuugcccauuauuaaaugaggacgguuugcaaaaacgacgucguuuuuguucaagcauucuucggcugcugauggugaugacacgacgauuacauua  -3'   exp
    (((((.((((((((((.((..((((((..(((((((((((((....))))))))..)))))..)))))..)).))))))).........).))).))))).. reads   mm   sample
    .........................................................aagcauucuGcggcugcugauggu.....................  1       1    S01
    .........................................................aagcauucuucggcugcugauggu.....................  1       0    S01
    ..........................................................agcauucuucggcugcugauggug....................  2       0    S01

    .........................................................aagcauucuucggcugcugaAggu.....................  1       1    S02
    .........................................................aagcauucuucggcugcugauggA.....................  1       1    S02
    .........................................................aagcauucuucggcugcugauggu.....................  4       0    S02
    ..........................................................agcauucuucggcugcugauggug....................  4       0    S02
```

Provisional ID	:	Maple_Unigene_BMK.11646_21221
Score total	:	294.8
Score for star read(s)	:	3.9
Score for read counts	:	285.6
Score for mfe	:	2.3
Score for randfold	:	0
Score for cons. seed	:	3
Total read count	:	572
Mature read count	:	409
Loop read count	:	0
Star read count	:	163

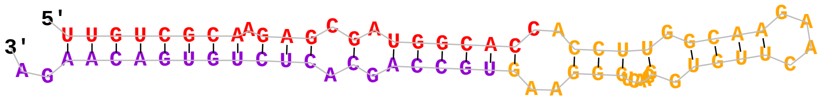

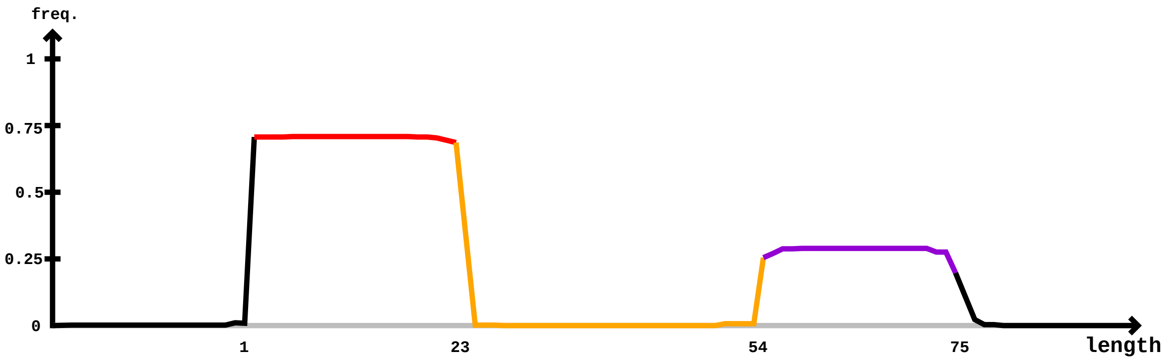


```
                         Mature                                                 Star
5'- aaaagacguucagggagucuugucgcaagagcgauggcaccaccuuggcaagaacuugugggaacugggaagugccagcacucugugacaagacacccugcaacuucuuua  -3'    obs
    aaaagacguucagggagucuugucgcaagagcgauggcaccaccuuggcaagaacuugugggaacugggaagugccagcacucugugacaagacacccugcaacuucuuua         exp
    .(((((.((((((((.(((((((((((.(((.(.((((((..((((.((((....)))).).....)))..)))))).).)))))))))))))).))))).))).))))).   reads   mm   sample
    aaCagacguucagggagu.............................................................................................   1       1    S01
    .................ucuugucgcaagagcgaug...........................................................................   1       0    S01
    .................ucuugucgcaagagcgauggca........................................................................   2       0    S01
    ...................uugucgcaagagcgauggc.........................................................................   2       0    S01
    ...................uugucgcaagagcgauggca........................................................................   1       0    S01
    ...................uugucgcaagagcgauggcac.......................................................................   4       0    S01
    ...................uuUucgcaagagcgauggcacc......................................................................   1       1    S01
    ...................Nugucgcaagagcgauggcacc......................................................................   1       1    S01
    ...................uugGcgcaagagcgauggcacc......................................................................   1       1    S01
    ...................uCgucgcaagagcgauggcacc......................................................................   1       1    S01
    ...................uugucgcaagagcgauggcaAc......................................................................   1       1    S01
    ...................uugucgcaagagcgauggcacc......................................................................   164     0    S01
    ...................uugucgcaagagcgaGggcacc......................................................................   1       1    S01
    ...................uuguUgcaagagcgauggcacc......................................................................   1       1    S01
    ...................uugucgcaagagcgauggcacG......................................................................   2       1    S01
    ...................uugucgcaagagcgauggcacA......................................................................   8       1    S01
    ...................uugucgcaGgagcgauggcacc......................................................................   2       1    S01
    ...................uugucgcaagaCcgauggcacc......................................................................   1       1    S01
    ...................uugucgcaagagcgauggcGcc......................................................................   1       1    S01
    ...................uugucgcaCgagcgauggcacc......................................................................   1       1    S01
    ...................uugucgcaagGgcgauggcacc......................................................................   3       1    S01
    ...................uugucgcaagagcgauggcacU......................................................................   35      1    S01
    ...................uugucgcaagagcgauAgcacc......................................................................   1       1    S01
    ...................uugucgcaagaUcgauggcacc......................................................................   1       1    S01
    .......................cgcaagagcgauggcacc......................................................................   1       0    S01
    ....................................................................gaagugccagcacucugugacaag...................   4       0    S01
    ........................................................................ugccagcacucugugaca.....................   6       0    S01
    ........................................................................ugccagcGcucugugacaag...................   1       1    S01
    ........................................................................ugccagUacucugugacaag...................   1       1    S01
    ........................................................................ugccagcacucugugacaag...................   28      0    S01
    ........................................................................ugccagcacucugugacaagU..................   1       1    S01
    ........................................................................ugccagcacucugugacaagG..................   1       1    S01
    ........................................................................ugccagcacucugugacaaga..................   65      0    S01
```

```
                   Mature                                                     Star
aaaagacguucagggagucuugucgcaagagcgauggcaccaccuuggcaagaacuugugggaacugggaagugccagcacucugugacaagacacccugcaacuucuuua
........................................................................ugcAagcacucugugacaaga..................  1    1   S01
........................................................................ugccagcacucugugaAaaga..................  1    1   S01
........................................................................ugccagcacAcugugacaaga..................  1    1   S01
........................................................................ugccagcacucugugacaUga..................  2    1   S01
........................................................................Cgccagcacucugugacaaga..................  1    1   S01
........................................................................ugccagcacGcugugacaaga..................  1    1   S01
........................................................................ugccagcacucugugaGaaga..................  1    1   S01
........................................................................ugccagcacucugugacaagaA.................  1    1   S01
........................................................................ugccagcacucugugacaagaUa................  1    1   S01
.........................................................................gccagcacucugugacaag...................  3    0   S01
.........................................................................Uccagcacucugugacaaga..................  1    1   S01
.........................................................................gccagcacucugugacaaga..................  3    0   S01
..........................................................................ccagcacucugugacaagac.................  1    0   S01
..........................................................................ccagcacucugugacaagacC................  1    1   S01
..........................................................................ccagcacucugugacaagaca................  4    0   S01
............................................................................agcacucugugacaagacacc..............  1    0   S01

.................ucuugucgcaagagcgauggca........................................................................  2    0   S02
...................uugucgcaagagcgauggcac.......................................................................  1    0   S02
...................uugucgcaagagcgauggcacU......................................................................  21   1   S02
...................uugucgcaagagcgauggcaUc......................................................................  1    1   S02
...................uugucgcaagagcgaCggcacc......................................................................  2    1   S02
...................uugucgcaagagcgauAgcacc......................................................................  1    1   S02
...................uugucgcaagGgcgauggcacc......................................................................  1    1   S02
...................uugGcgcaagagcgauggcacc......................................................................  1    1   S02
...................uugucgcaagagcgauggcacA......................................................................  9    1   S02
...................uugucgcaagagcgauggcacc......................................................................  118  0   S02
...................Nugucgcaagagcgauggcacc......................................................................  1    1   S02
...................uugucgcaagagcgauggcGcc......................................................................  1    1   S02
...................uugucgcaagagcgauggcacG......................................................................  3    1   S02
...................uugucgcaagaUcgauggcacc......................................................................  1    1   S02
...................Cugucgcaagagcgauggcacc......................................................................  1    1   S02
...................uugucgcaGgagcgauggcacc......................................................................  1    1   S02
...................uugCcgcaagagcgauggcacc......................................................................  1    1   S02
...................uugucgcaagagUgauggcacc......................................................................  1    1   S02
...................uugucgcaagagGgauggcacc......................................................................  1    1   S02
...................uugucgcaagagcgaGggcacc......................................................................  1    1   S02
...................uugucgUaagagcgauggcacc......................................................................  1    1   S02
...................uugucgcGagagcgauggcacc......................................................................  1    1   S02
...................uugucgcaagagcgauggcaccaccC..................................................................  1    1   S02
........................................................................ugccagcacucugugaca.....................  1    0   S02
........................................................................ugccagcacucugugacG.....................  1    1   S02
........................................................................ugccagcacucugugacaag...................  8    0   S02
........................................................................ugccagcacucugugacaaga..................  18   0   S02
........................................................................ugccagcacucugGgacaaga..................  1    1   S02
........................................................................Agccagcacucugugacaaga..................  1    1   S02
.........................................................................gccagcacucugugacaagacU................  1    1   S02
.........................................................................gccagcacucugugacaagacacU..............  1    1   S02
..........................................................................ccagcacucugugUcaagaca................  1    1   S02
..........................................................................ccagcacucugugacaagaca................  3    0   S02
```

Provisional ID	:	Maple_Unigene_BMK.12419_23059
Score total	:	538.2
Score for star read(s)	:	3.9
Score for read counts	:	533.9
Score for mfe	:	1
Score for randfold	:	0
Score for cons. seed	:	-0.6
Total read count	:	1059
Mature read count	:	1053
Loop read count	:	5
Star read count	:	1

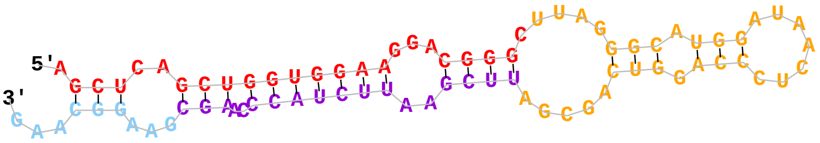

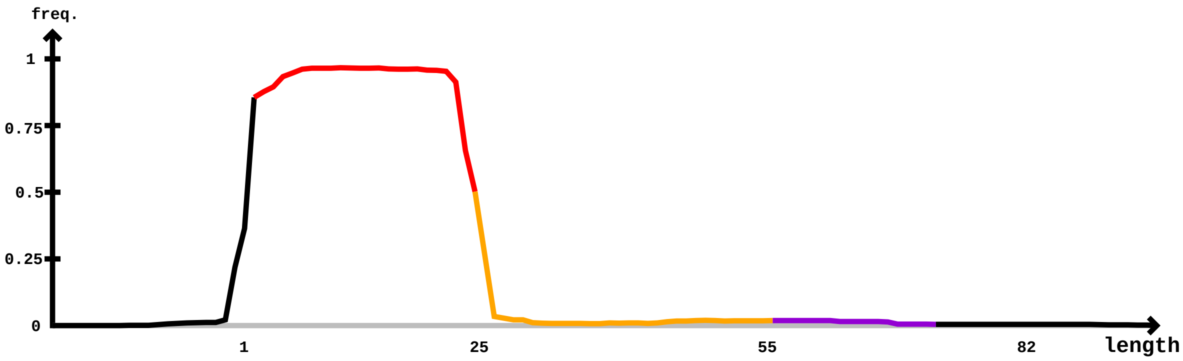

Mature **Star**

```
5'- gaccggccaucagggggcuagcucagcugguggaaggacgggcuuagggcauggauaacucccaggucagcgauucgaauucuacccaagcgaaggcaaguucugauuuaccu -3'   obs
    gaccggccaucagggggcuagcucagcugguggaaggacgggcuuagggcauggauaacucccaggucagcgauucgaauucuacccaagcgaaggcaaguucugauuuaccu         exp
    ....((..((((((..(((.(((..((((((((((...((((.....(((.(((.......))).))).....))))..)))))))..)))...))).)))))))))...)).   reads   mm   sample
    ......ccaucagggggcuagcucagcuA....................................................................................   1       1    S01
    .........ucagggCgcuagcucagcuggugg................................................................................   1       1    S01
    ..........cagggCgcuagcucagcuggugg................................................................................   1       1    S01
    ................Acuagcucagcugguggaaggacg.........................................................................   1       1    S01
    ................gUuagcucagcugguggaaggacg.........................................................................   1       1    S01
    ................Ucuagcucagcugguggaaggacg.........................................................................   1       1    S01
    .................cuagcucaAcugguggaagg............................................................................   1       1    S01
    .................cuagcucagcugguggaaggac..........................................................................   1       0    S01
    .................cuagUucagcugguggaaggac..........................................................................   1       1    S01
    .................cuagcucagcugguggaaggacg.........................................................................   2       0    S01
    .................cuagcucaAcugguggaaggacg.........................................................................   1       1    S01
    .................cuagcucagcuggAggaaggacgg........................................................................   1       1    S01
    .................cuagcuUagcugguggaaggacgg........................................................................   2       1    S01
    .................cuagUucagcugguggaaggacgg........................................................................   4       1    S01
    .................cuagcucagcuggugAaaggacgg........................................................................   1       1    S01
    .................cuagcucagcugguggaaggaAgg........................................................................   1       1    S01
    .................cuGgcucagcugguggaaggacgg........................................................................   1       1    S01
    .................cuagcucagcugguggaaAgacgg........................................................................   4       1    S01
    .................cuagcucagcuCguggaaggacgg........................................................................   1       1    S01
    .................cuagcucagcugAuggaaggacgg........................................................................   8       1    S01
    .................cuagcucagcugguggaaggacgg........................................................................   38      0    S01
    .................cuagcAcagcugguggaaggacgg........................................................................   1       1    S01
    .................cuagcucagcugguggaaggacgA........................................................................   3       1    S01
    .................cuaAcucagcugguggaaggacgg........................................................................   4       1    S01
    .................cuagcucaAcugguggaaggacgg........................................................................   12      1    S01
    .................cuagcucUgcugguggaaggacgg........................................................................   1       1    S01
    .................cuagcucagUugguggaaggacgg........................................................................   6       1    S01
    .................cuagcucagcAgguggaaggacgg........................................................................   1       1    S01
    .................Uuagcucagcugguggaaggacgg........................................................................   3       1    S01
    .................cuaUcucagcugguggaaggacgg........................................................................   2       1    S01
    .................cuagcucagcugguggaaggacggA.......................................................................   1       1    S01
    .................cuagcucagcugguggaaggacggg.......................................................................   3       0    S01
    .................cuagcucagcuggugAaaggacggg.......................................................................   1       1    S01
```

Mature Star

gaccggccaucagggggcuagcucagcugguggaaggacgggcuuagggcauggauaacucccaggucagcgauucgaauucuacccaagcgaaggcaaguucugauuuaccu			
..................uagcucagcuggugAaagg..	1	1	S01
..................uagcucagcugguggaaggacgg..	4	0	S01
..................uagcucagcugguggaaggacgU..	1	1	S01
..................uagcucaAcugguggaaggacgg..	1	1	S01
..................uagcucagcuCguggaaggacgg..	1	1	S01
..................uagcuUagcugguggaaggacgg..	1	1	S01
..................uagcucagcugguggaGggacgg..	1	1	S01
..................uagcucagcCgguggaaggacggg...	1	1	S01
..................uagcCcagcugguggaaggacggg...	1	1	S01
..................uagcucagcugguggaaggacggg...	10	0	S01
..................uagcucagcugguggaaAgacggg...	3	1	S01
..................uagcucagcugguggaaggacgAg...	1	1	S01
..................uagcucagcugguggaaggacAgg...	1	1	S01
..................uagcucaAcugguggaaggacggg...	3	1	S01
..................uagcucagcuggugAaaggacggg...	4	1	S01
..................uagcucagcuAguggaaggacggg...	5	1	S01
..................uagcucUgcugguggaaggacggg...	3	1	S01
..................uagUucagcugguggaaggacggg...	1	1	S01
..................uaAcucagcugguggaaggacggg...	1	1	S01
..................uagcucagcugguggaaggacgCg...	2	1	S01
..................uagcucagcugguggaaggacggA...	9	1	S01
..................uagcucagUugguggaaggacggg...	6	1	S01
...................agcucagcugguggaaggacA...	2	1	S01
...................aAcucagcugguggaaggacg...	1	1	S01
...................agUucagcugguggaaggacgg..	1	1	S01
...................agcucagcugguggaaggacgg..	2	0	S01
...................agcucagcuggAggaaggacgg..	1	1	S01
...................agcucagUugguggaaggacggg...	2	1	S01
...................agcucagcugguggaaggacggA...	1	1	S01
...................agcucagcuAguggaaggacggg...	1	1	S01
...................agcucagcugguggaaggacggg...	4	0	S01
...................agcucaAcugguggaaggacggg...	1	1	S01
...................agcucagcugguggaaAgacgggc..	30	1	S01
...................agUucagcugguggaaggacgggc..	5	1	S01
...................agcucUgcugguggaaggacgggc..	3	1	S01
...................agcucagcuggugAaaggacgggc..	2	1	S01
...................agcucagcugUuggaaggacgggc..	6	1	S01
...................agcucagcugguggaaggacgggG..	3	1	S01
...................agcucagcugguggaGggacgggc..	1	1	S01
...................agcucagcugAuggaaggacgggc..	2	1	S01
...................aAcucagcugguggaaggacgggc..	1	1	S01
...................agcucagcugguggaaggacAggc..	2	1	S01
...................agcucagcugguggaaggacgggc..	86	0	S01
...................agcucagcugguggaaggGcgggc..	1	1	S01
...................agcucagcugguggGaggacgggc..	1	1	S01
...................agcucagcuggAggaaggacgggc..	1	1	S01
...................agcucGgcugguggaaggacgggc..	1	1	S01
...................agcucagcugguggaaggacUggc..	1	1	S01
...................agcucagcuggCggaaggacgggc..	1	1	S01
...................agcucagcuAguggaaggacgggc..	7	1	S01
...................agcucagcugguggaaggCcgggc..	1	1	S01
...................agcucagcugguggaaggacgggU..	74	1	S01
...................agcuAagcugguggaaggacgggc..	1	1	S01
...................Ggcucagcugguggaaggacgggc..	3	1	S01
...................agcucaAcugguggaaggacgggc..	6	1	S01
...................agcucagcAgguggaaggacgggc..	1	1	S01
...................agcucagcugguggaaggacgggA..	6	1	S01
...................agcucagUugguggaaggacgggc..	6	1	S01
....................gcucagcugguAgaagga...	1	1	S01
....................gUucagcugguggaaggac..	1	1	S01
....................gcucagcuAguggaaggacg...	1	1	S01
....................gcucagcugguggaaAgacgg..	1	1	S01
....................gUucagcugguggaaggacgg..	1	1	S01
....................gcucagcuggGggaaggacgg..	1	1	S01
....................gcucagcugguggaaggacgg..	5	0	S01
....................gcucagcugguggaaggacAg..	1	1	S01
....................gcucagcuAguggaaggacgg..	2	1	S01
....................gcucagcugguggaaAgacggg...	1	1	S01
....................gcucagcugguggaaggacgggc..	1	0	S01
....................gcucagcugguggaaggacgggcC...	1	1	S01

Mature   Star

```
gaccggccaucagggggcuagcucagcugguggaaggacgggcuuagggcauggauaacucccaggucagcgauucgaauucuacccaagcgaaggcaaguucugauuuaccu
.....................cucagcuAguggaaggacg.........................................................................   1    1    S01
.....................cucagcugguggaaggacgg........................................................................   2    0    S01
.....................cucagcugguggaaAgacgg........................................................................   1    1    S01
.....................cucagcuAguggaaggacgg........................................................................   1    1    S01
.....................cucagcugAuggaaggacggg.......................................................................   1    1    S01
.....................cucagcugguggaaggacggAcuu....................................................................   1    1    S01
......................ucagUugguggaaggacg.........................................................................   1    1    S01
......................ucagcugguggaaggacgg........................................................................   2    0    S01
......................ucaAcugguggaaggacgg........................................................................   2    1    S01
......................ucagcuAguggaaggacgg........................................................................   1    1    S01
......................ucagcuAguggaaggacggg.......................................................................   1    1    S01
......................ucagcugguggaaAgacggg.......................................................................   1    1    S01
......................ucagcugguggaaggacggg.......................................................................   3    0    S01
......................ucagcugguggaaggacgggc......................................................................   2    0    S01
......................ucagcugguggaUggacgggc......................................................................   1    1    S01
......................ucagcugAuggaaggacgggc......................................................................   1    1    S01
......................ucagcuAguggaaggacgggc......................................................................   1    1    S01
......................ucagcugguggaaggacgggU......................................................................   1    1    S01
......................ucagcugguggaaggacgggcuu....................................................................   2    0    S01
......................ucaAcugguggaaggacgggcuu....................................................................   1    1    S01
.......................cagcugguggaaAgacgg........................................................................   1    1    S01
.......................cagcugguggaaUgacgg........................................................................   1    1    S01
.......................cagcugguggaaggacgg........................................................................   3    0    S01
.......................cagcugguggaaggacggU.......................................................................   1    1    S01
.......................cagcuAguggaaggacggg.......................................................................   1    1    S01
.......................cagcuggugAaaggacggg.......................................................................   1    1    S01
........................agcugguggaaggacgggU......................................................................   1    1    S01
........................agcugguggaaggacgggcuC....................................................................   1    1    S01
........................agcugguggaaggacggAcuua...................................................................   1    1    S01
........................agcugguggaaggacgggcuuG...................................................................   1    1    S01
........................agcugguggaaggacggAcuuagg.................................................................   3    1    S01
........................agcugguggaaggacgggcuuGgg.................................................................   1    1    S01
........................agcugguggaaggacgggcuuagg.................................................................   2    0    S01
............................gguggaaggacgggcuuagA.................................................................   1    1    S01
...................................ggacgggcuuagggcauggauaacu.....................................................   1    0    S01
.............................................agggcauggGuaacuccca.................................................   1    1    S01
.............................................agggcauggauaacucUcagguc.............................................   1    1    S01
........................................................aacucccaggucagcgGuucgaau.................................   1    1    S01
..........................................................cucUcaggucagcgauucgaau.................................   1    1    S01
.............................................................ccaggucagcgauucgaauucuacc...........................   1    0    S01
..............................................................caggucagcgauucgaauucuacc...........................   1    0    S01
................................................................ggucagcgGuucgaauucuacc...........................   1    1    S01
.................................................................gucaAcgauucgaauucuacc...........................   1    1    S01
...................................................................cagcgauucgaauucuacccaagc......................   1    0    S01
.........................................................................uucgaauucuaccUaagc......................   1    1    S01

.........ucagggCgcuagcucagcuggu..................................................................................   1    1    S02
.........ucagggCgcuagcucagcuggugg................................................................................   1    1    S02
..........cGggggcuagcucagcuggugg.................................................................................   1    1    S02
..........cagggUgcuagcucagcuggugga...............................................................................   1    1    S02
...........agggCgcuagcucagcugguggaa..............................................................................   1    1    S02
...........agggCgcuagcucagcugguggaaggacg.........................................................................   1    1    S02
............gggggcuagcucagcugg...................................................................................   1    0    S02
............gggggcuagcucagcuggugga...............................................................................   1    0    S02
.............ggggcuagcucagcCgguggaagg............................................................................   1    1    S02
..............gCgcuagcucagcugguggaaggacgg........................................................................   1    1    S02
................Acuagcucagcugguggaaggacg.........................................................................   1    1    S02
................gcuagUucagcugguggaaggacg.........................................................................   1    1    S02
................gUuagcucagcugguggaaggacg.........................................................................   5    1    S02
................gcuagcucaAcugguggaaggacg.........................................................................   1    1    S02
.................cuaAcucagcugguggaagg............................................................................   1    1    S02
.................cuagcucagcugguggaaAgacg.........................................................................   1    1    S02
.................cuagcucagcuCguggaaggacg.........................................................................   1    1    S02
.................cuagcucagcugguggaaggacU.........................................................................   1    1    S02
.................cuagUucagcugguggaaggacg.........................................................................   1    1    S02
.................cuagcucaAcugguggaaggacg.........................................................................   1    1    S02
.................cuagcucagUugguggaaggacg.........................................................................   1    1    S02
.................cuagcucagcugguggaaggacg.........................................................................   3    0    S02
.................cuagcucagGugguggaaggacgg........................................................................   1    1    S02
.................cuagcucaAcugguggaaggacgg........................................................................   16   1    S02
```

```
                 Mature                                       Star
gaccggccaucagggggcuagcucagcugguggaaggacgggcuuagggcauggauaacucccaggucagcgauucgaauucuacccaagcgaaggcaaguucugauuuaccu
.................cuagcucagcuggCggaaggacgg........................................................................  1   1   S02
.................cuagcucagcCgguggaaggacgg........................................................................  1   1   S02
.................cuagcucagcugAuggaaggacgg........................................................................  4   1   S02
.................Uuagcucagcugguggaaggacgg........................................................................  3   1   S02
.................cuagcucagcugguggaaggacCg........................................................................  2   1   S02
.................cuagcucagcuAguggaaggacgg........................................................................  3   1   S02
.................cuagcucagcuggugAaaggacgg........................................................................  1   1   S02
.................cuagcCcagcugguggaaggacgg........................................................................  2   1   S02
.................cuagcucagUugguggaaggacgg........................................................................  5   1   S02
.................cuagcucagcugguggaaUgacgg........................................................................  2   1   S02
.................cuagcucagcuCguggaaggacgg........................................................................  6   1   S02
.................cuaAcucagcugguggaaggacgg........................................................................  1   1   S02
.................cuagcAcagcugguggaaggacgg........................................................................  1   1   S02
.................cuagUucagcugguggaaggacgg........................................................................  9   1   S02
.................cuagcucagcugguggaaAgacgg........................................................................  1   1   S02
.................cuagcucagcugguggaaggacgA........................................................................  1   1   S02
.................cuagcucUgcugguggaaggacgg........................................................................  2   1   S02
.................cuagcucagcugguggaaggacgg........................................................................  38  0   S02
.................cuagcucagcugguggaaggacggg.......................................................................  1   0   S02
.................cuagcucagcugAuggaaggacggg.......................................................................  2   1   S02
.................cuagcucagcugguggaagAacggg.......................................................................  1   1   S02
.................cuagcucagcugguggaaggaUggg.......................................................................  1   1   S02
..................uagUucagcugguggaagg............................................................................  1   1   S02
..................uagcucagcugguggaaggacg.........................................................................  2   0   S02
..................uagUucagcugguggaaggacg.........................................................................  1   1   S02
..................uagcuUagcugguggaaggacg.........................................................................  1   1   S02
..................uagcucagcugguggaaggaUgg........................................................................  1   1   S02
..................uagcucagcuAguggaaggacgg........................................................................  1   1   S02
..................uagcucCgcugguggaaggacgg........................................................................  1   1   S02
..................uagcucagUugguggaaggacgg........................................................................  1   1   S02
..................uagcucagcugguggaaggacgg........................................................................  14  0   S02
..................uagcucagcGgguggaaggacgg........................................................................  1   1   S02
..................uagcucagcuggugAaaggacgg........................................................................  1   1   S02
..................uagcucagcugAuggaaggacgg........................................................................  2   1   S02
..................uagUucagcugguggaaggacgg........................................................................  4   1   S02
..................uagcucagcugguggaaUgacggg.......................................................................  1   1   S02
..................uagcucagUugguggaaggacggg.......................................................................  6   1   S02
..................uagcucagcugguggaaggacggA.......................................................................  5   1   S02
..................uagcucagcugguggaaggacggU.......................................................................  1   1   S02
..................uagcucagcugguggaaggacgAg.......................................................................  3   1   S02
..................uagcucagcuAguggaaggacggg.......................................................................  3   1   S02
..................uagcucaAcugguggaaggacggg.......................................................................  6   1   S02
..................uagcucagcugCuggaaggacggg.......................................................................  1   1   S02
..................uagcucUgcugguggaaggacggg.......................................................................  2   1   S02
..................uagcucagcuggugAaaggacggg.......................................................................  1   1   S02
..................uagcucagcugguggaaAgacggg.......................................................................  11  1   S02
..................uagcucagcugguggaaggacggg.......................................................................  27  0   S02
..................uagcucagcCgguggaaggacggg.......................................................................  1   1   S02
..................uagcucagcugguggaaggacggC.......................................................................  1   1   S02
..................uagcucagcugAuggaaggacgggc......................................................................  1   1   S02
...................agcucagcugguggaagg............................................................................  1   0   S02
...................agcucagcugguggaaggaU..........................................................................  1   1   S02
...................agcucagcugguggaaggacA.........................................................................  2   1   S02
...................agcucagcuAguggaaggacg.........................................................................  1   1   S02
...................agcucagcugguggaaAgacg.........................................................................  1   1   S02
...................agcucagcugguggaaggacgg........................................................................  4   0   S02
...................aAcucagcugguggaaggacgg........................................................................  1   1   S02
...................agcucagcugguggaaggacgA........................................................................  1   1   S02
...................agUucagcugguggaaggacgg........................................................................  1   1   S02
...................agcucagcugguggaaggaUgg........................................................................  1   1   S02
...................agcucagUugguggaaggacgg........................................................................  1   1   S02
...................agcucaAcugguggaaggacgg........................................................................  1   1   S02
...................agcucagcugguggaaAgacgg........................................................................  3   1   S02
...................agcucagcugguggaaggacggg.......................................................................  7   0   S02
...................agcucagUugguggaaggacggg.......................................................................  1   1   S02
...................agcucagcugguggaaggacggA.......................................................................  2   1   S02
...................agcucagcugguggaaAgacggg.......................................................................  1   1   S02
...................agcucaAcugguggaaggacggg.......................................................................  2   1   S02
...................agcucagcugguggaaggacAgg.......................................................................  1   1   S02
...................agcucagcugguggaaAgacgggc......................................................................  12  1   S02
```

Mature Star

gaccggccaucagggggcuagcucagcugguggaaggacgggcuuagggcauggauaacucccaggucagcgauucgaauucuacccaagcgaaggcaaguucugauuuaccu			
...................agcucGgcugguggaaggacgggc..	1	1	S02
...................agcucagcugguggaaggacgggU..	46	1	S02
...................agcucagcugguggaagAacgggc..	1	1	S02
...................agcucagcugguggaaggacgggc..	110	0	S02
...................agcucagcugguggaaggacggAc..	3	1	S02
...................agcucagcugAuggaaggacgggc..	2	1	S02
...................agcucagcuAguggaaggacgggc..	12	1	S02
...................agcucagcugguggaaggacAggc..	2	1	S02
...................agcucagUugguggaaggacgggc..	12	1	S02
...................agcucagcugguggaaggacgggA..	6	1	S02
...................agcucagcugguggaaggacgAgc..	1	1	S02
...................agcucagcuggCggaaggacgggc..	1	1	S02
...................aAcucagcugguggaaggacgggc..	5	1	S02
...................agcucagcugguggGaggacgggc..	1	1	S02
...................agUucagcugguggaaggacgggc..	6	1	S02
...................agcucaAcugguggaaggacgggc..	7	1	S02
...................agcucagcugguggaGggacgggc..	1	1	S02
...................Ggcucagcugguggaaggacgggc..	1	1	S02
...................agcucUgcugguggaaggacgggc..	3	1	S02
...................agcucagcugguggaaggaAgggc..	1	1	S02
...................agcucagcugguAgaaggacgggc..	1	1	S02
...................agcucagcugUuggaaggacgggc..	12	1	S02
....................gcucagGugguggaaggacg...	1	1	S02
....................Acucagcugguggaaggacgg..	1	1	S02
....................gcucagcugguggaaAgacgg..	1	1	S02
....................gcucagcugguggaaggacgg..	2	0	S02
....................gcucagcugguggaaggacgggU..	1	1	S02
....................gcucagcugguggaaggacgggc..	1	0	S02
.....................cucaAcugguggaaggacgg..	2	1	S02
.....................cucagcugguggaaggacgg..	1	0	S02
.....................cucagcugAuggaaggacgg..	1	1	S02
.....................cucagcugguggaaggacggg...	1	0	S02
.....................cucagcugguggaaggacggA...	1	1	S02
.....................cucagcugguggaaggacgggc..	1	0	S02
.....................cucagcugguggaaggacgggcu...	3	0	S02
.....................cucagcuAguggaaggacgggcuu..	1	1	S02
.....................cucagcugguggaaggacgggUuu..	1	1	S02
.....................cucagcugguggaaggaUgggcuu..	1	1	S02
......................ucagcugguggaaggacA...	1	1	S02
......................ucagcugguggaaggacg...	1	0	S02
......................ucagcugguggaaggacC...	2	1	S02
......................ucagcugAuggaaggacg...	2	1	S02
......................ucagcugguggaaggacgU..	1	1	S02
......................ucagcugguggaaggacgg..	2	0	S02
......................ucUgcugguggaaggacggg...	1	1	S02
......................ucagcugguggaaAgacggg...	1	1	S02
......................ucagcugguggaaggacgUg...	1	1	S02
......................ucagcuAguggaaggacgggc..	1	1	S02
......................ucagcugguggaaggacgggc..	1	0	S02
......................Acagcugguggaaggacgggc..	1	1	S02
......................ucUgcugguggaaggacgggc..	1	1	S02
......................ucagcuAguggaaggacgggcuu..	1	1	S02
......................ucagcugguggaaggaUgggcuu..	1	1	S02
......................ucagcugguggaaggacgggcuuU...	1	1	S02
......................ucagcugguggaaggacgggcuuG...	1	1	S02
......................ucagcugguggaaggacgggcuua...	1	0	S02
......................ucagcugguggaaggacgggcuuC...	2	1	S02
.......................cagcCgguggaaggacgg..	1	1	S02
.......................cagcugguggaaAgacgg..	1	1	S02
.......................cagcuAguggaaggacgg..	1	1	S02
.......................cagcugguggaaggacgUg...	1	1	S02
.......................cagcugguggaaggacggA...	1	1	S02
.......................cagcugAuggaaggacggg...	1	1	S02
.......................cagcuAguggaaggacgggc..	1	1	S02
........................agcugguggaaggacggA...	1	1	S02
........................agcuAguggaaggacgggc..	1	1	S02
........................agcugguggaaggacggAcuuagg...	1	1	S02
........................agUugguggaaggacgggcuuagg...	1	1	S02
........................agcugguggaaggacgggcuuGgg...	2	1	S02
.........................gcugguggaaggacgggc..	1	0	S02

```
                    Mature                                                  Star
gaccggccaucagggggcuagcucagcugguggaaggacgggcuuagggcauggauaacucccaggucagcgauucgaauucuacccaagcgaaggcaaguucugauuuaccu
.........................Ccugguggaaggacgggc......................................................................    1    1    S02
.........................gcugguggaaggacgggU......................................................................    1    1    S02
.........................gcugguggaaggacgggcuuGggg................................................................    1    1    S02
............................gguggaaggacggAcuuagg.................................................................    1    1    S02
...............................ggaaggacgggcuuaggg................................................................    1    0    S02
................................gaaggacgggcuuagggc...............................................................    1    0    S02
..................................aggacgggcuuGgggcauggaua........................................................    1    1    S02
....................................gacgggcuuagggcaugg...........................................................    1    0    S02
.....................................acgggcuuGgggcauggauaacu.....................................................    1    1    S02
...........................................uuagggcauggauaacucccaggu..............................................    2    0    S02
.............................................agggcauggauaacucccagguc.............................................    1    0    S02
........................................................aacucccaggucagcgGuucgaau.................................    2    1    S02
.............................................................ccaggucagcgauucgaauucuac............................    1    0    S02
..............................................................caggucagcgauucgaauucu..............................    1    0    S02
..............................................................caggucagcgaCucgaauucuac............................    1    1    S02
..............................................................caggucagcgaCucgaauucuacc...........................    2    1    S02
...............................................................aggucaAcgauucgaauucuacc...........................    2    1    S02
...............................................................aggucagcgauucgaauucuaccc..........................    1    0    S02
.................................................................gucagcgauucgaauucuacc...........................    1    0    S02
..................................................................ucagcgauucgaauucuacccaag.......................    1    0    S02
.....................................................................gcgauucgaauucuaAcc..........................    1    1    S02
...................................................................................acccaUgcgaaggcaaguucugau......    1    1    S02
.......................................................................................aagcgaaggcaaAuucugauu.....    1    1    S02
.......................................................................................aGgcgaaggcaaguucugauuuac..    1    1    S02
............................................................................................aaggcaaguucugauuuacUu    1    1    S02
............................................................................................aaggcGaguucugauuuaccu    1    1    S02
```

Provisional ID	:	Maple_Unigene_BMK.12710_23799
Score total	:	0.5
Score for star read(s)	:	-1.3
Score for read counts	:	0
Score for mfe	:	2.4
Score for randfold	:	0
Score for cons. seed	:	-0.6
Total read count	:	568
Mature read count	:	568
Loop read count	:	0
Star read count	:	0

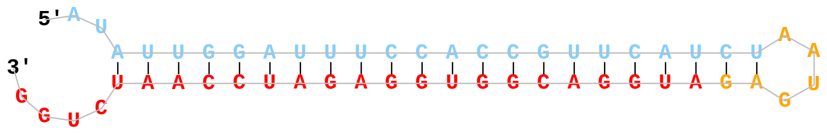

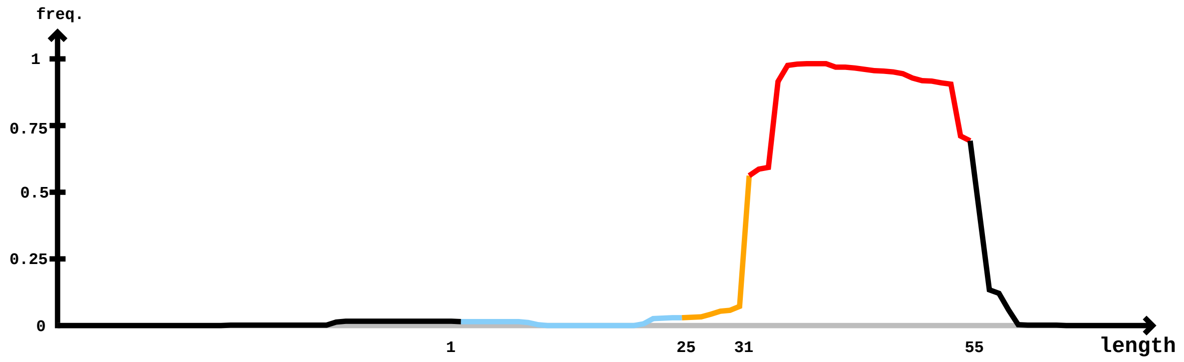

Star Mature

```
5'- uuugauuuuuuuuauaaagagauagauuugguggaaguccauauuggauuuccaccguucaucuaaugagauggacgguggagauccaaucuggaucuccaacaaauccaaa   -3'   exp
    ....(((((((.....))))))).((((((.((((..((((.((((((((((((((((((((((....)))))))))))))))))))))).)))).)))).))))))....   reads   mm   sample
    ................aagagauGgauuugguggaagucc........................................................................   1   1   S01
    ...........................uugguggaaguccaGauugga................................................................   3   1   S01
    ............................ugguggaaguccaGauuggau...............................................................   1   1   S01
    ...........................................................caucuCaugagauggacggu.................................   2   1   S01
    ...........................................................caucuCaugagauggacgguggag.............................   1   1   S01
    ............................................................aucuUaugagauggacggu.................................   1   1   S01
    ............................................................aucuCaugagauggacggu.................................   3   1   S01
    ............................................................aucuCaugagauggacggugg...............................   1   1   S01
    ............................................................aucuCaugagauggacggugga..............................   1   1   S01
    ............................................................aucuCaugagauggacgguggag.............................   1   1   S01
    ............................................................aucuaaugUgauggacgguggaga............................   1   1   S01
    ..............................................................cuCaugagauggacgguggagau...........................   1   1   S01
    ................................................................Caugagauggacggugga..............................   1   1   S01
    ..................................................................ugagauAgacgguggagau...........................   1   1   S01
    ..................................................................ugagauggacgguggagauccaau......................   1   0   S01
    ...................................................................gagauggacgguggagauA..........................   1   1   S01
    ...................................................................gagauggacgguggUgauc..........................   1   1   S01
    ...................................................................gagauggacgguAgagaucc.........................   1   1   S01
    ...................................................................gagauggaAgguggagaucc.........................   1   1   S01
    ...................................................................gagauggacgguggagauccaauc.....................   1   0   S01
    ....................................................................agauggacgguggagauA..........................   1   1   S01
    .....................................................................gauggacgguggagaucc.........................   2   0   S01
    .....................................................................gauggacgguggUgaucca........................   1   1   S01
    ......................................................................auggacgguggagaucca........................   1   0   S01
    ......................................................................auggacgguggagauccaaucC....................   2   1   S01
    ......................................................................auggacgguggagaucUaaucu....................   2   1   S01
    ......................................................................auggacgguggagauccaaucu....................   19  0   S01
    ......................................................................auggacggugAagauccaaucu....................   1   1   S01
    ......................................................................auggacggugCagauccaaucu....................   1   1   S01
    ......................................................................auggacgguggagauccaaucug...................   1   0   S01
    ......................................................................auggacgguggagauccaauAugg..................   5   1   S01
    ......................................................................auggacgguggagauccaaucugg..................   27  0   S01
    ......................................................................auggacgguggagaucUaaucugg..................   1   1   S01
    ......................................................................auggacggugCagauccaaucugg..................   2   1   S01
```

```
                                                        Star                          Mature
uuugauuuuuuuuauaaagagauagauuugguggaagucc auauuggauuuccaccguucaucu aaugag auggacgguggagauccaaucugg aucuccaacaaauccaaa
......................................................................auggacgguggagauccaauUugg..................	1	1	S01
......................................................................auggacgguggaUauccaaucugg..................	1	1	S01
......................................................................augAacgguggagauccaaucugg..................	2	1	S01
......................................................................auggacgAuggagauccaaucugg..................	1	1	S01
......................................................................auggacgguggagauccaaucugU..................	1	1	S01
......................................................................auggacgguggagauccaaucugA..................	4	1	S01
......................................................................auAgacgguggagauccaaucugg..................	1	1	S01
......................................................................auggacgguggagauccaaucuggU.................	1	1	S01
......................................................................auggacgguggagauccaaucuggG.................	2	1	S01
.......................................................................uggacggugUagauccaau......................	1	1	S01
.......................................................................uggacgguggagauccaauUugg..................	1	1	S01
.......................................................................uggacggugUagauccaaucugg..................	1	1	S01
.......................................................................ugAacgguggagauccaaucugga.................	1	1	S01
.......................................................................uggacgguggagauccaaucugga.................	2	0	S01
........................................................................Agacgguggagauccaaucugg..................	2	1	S01
........................................................................Agacgguggagauccaaucuggau................	1	1	S01
.........................................................................gacgguggagauccaUucu....................	5	1	S01
.........................................................................gacgguggagauccaaucu....................	6	0	S01
.........................................................................Aacgguggagauccaaucu....................	3	1	S01
.........................................................................gacgguggagaucUaaucu....................	1	1	S01
.........................................................................gacgguggagauccaaucC....................	1	1	S01
.........................................................................gacggugAagauccaaucu....................	1	1	S01
.........................................................................gacAguggagauccaaucugg..................	1	1	S01
.........................................................................Aacgguggagauccaaucugg..................	2	1	S01
.........................................................................gacgguggagauccaauAugg..................	2	1	S01
.........................................................................gacgguggagauccaUucugg..................	14	1	S01
.........................................................................gacggugAagauccaaucugg..................	2	1	S01
.........................................................................gacgguggagauccaaucugU..................	1	1	S01
.........................................................................gacgguggagauccaaucugg..................	5	0	S01
.........................................................................gacgguggagauccaaucugA..................	1	1	S01
.........................................................................gacgguggagaCccaaucugg..................	1	1	S01
.........................................................................gacgguggagauccaaucuggG.................	1	1	S01
.........................................................................gacgguggagauccaaucuggau................	1	0	S01
.........................................................................gacgguggagauccaaucuggaG................	1	1	S01
.........................................................................gacggugUagauccaaucuggauc...............	1	1	S01
.........................................................................gacgguggGgauccaaucuggauc...............	1	1	S01
.........................................................................gacgguggagaucAaaucuggauc...............	2	1	S01
.........................................................................gacgguggagauccaaucuggauU...............	1	1	S01
.........................................................................gacgguggagauccaGucuggauc...............	1	1	S01
.........................................................................gacgguggagauccaaucuggauc...............	9	0	S01
.........................................................................gacgguggagauccGaucuggauc...............	1	1	S01
.........................................................................gacgguggagauUcaaucuggauc...............	5	1	S01
.........................................................................gacgguggagauccaaCcuggauc...............	1	1	S01
.........................................................................gacgguggagauUcaaucuggaucu..............	2	1	S01
..........................................................................acgguggagauccaaucuggauA...............	1	1	S01
..........................................................................acgguggagauUcaaucuggaucu..............	12	1	S01
..........................................................................acgguggagaucAaaucuggaucu..............	1	1	S01
..........................................................................acgguggagauccaaucuggaucu..............	2	0	S01
..........................................................................acgguggagauccaaucuggaucC..............	3	1	S01

...........................uugguggaaguccaGauugg.................................................................	2	1	S02
...........................uugguggaaguccaGauugga................................................................	2	1	S02
............................ugguggaaguccaGauuggau...............................................................	1	1	S02
...........................................................caucuCaugagauggacgguggag.............................	1	1	S02
............................................................aucuCaugagauggacggu.................................	2	1	S02
............................................................aucuCaugagauggacggugg...............................	1	1	S02
............................................................aucuCaugagauggacgguggaga............................	1	1	S02
.............................................................ucuCaugagauggacggugga..............................	1	1	S02
.................................................................Uugagauggacgguggagaucca........................	1	1	S02
..................................................................ugagauggacgguAgagauc..........................	1	1	S02
..................................................................ugagauggacgguggagaucca........................	1	0	S02
..................................................................ugagauUgacgguggagaucca........................	1	1	S02
..................................................................ugagauggacgguggagauccaauU.....................	1	1	S02
...................................................................gagauggaUgguggagaucc.........................	1	1	S02
...................................................................gagauggacgguggagauccaau......................	1	0	S02
....................................................................agauggacgguggaCaucc.........................	1	1	S02
.....................................................................gauggacgCuggagaucc.........................	1	1	S02
.....................................................................gauggGcgguggagaucc.........................	1	1	S02
.....................................................................gauggacAguggagaucc.........................	2	1	S02
.....................................................................gauggacAguggagauccaaucug...................	1	1	S02
```

```
                                        Star                          Mature
uuugauuuuuuuuauaaagagauagauuugguggaaguccauauuggauuuccaccguucaucuaaugagauggacgguggagauccaaucuggaucuccaacaaauccaaa
.....................................................................gauggacggugAagauccaaucug...................   1   1   S02
......................................................................auggacgguggagaucca........................   1   0   S02
......................................................................auggacgguggagauccaUu......................   1   1   S02
......................................................................auggacgguggagauccaauc.....................   1   0   S02
......................................................................auggacgguggagauccaaucu....................   38  0   S02
......................................................................Guggacgguggagauccaaucu....................   1   1   S02
......................................................................auggacggugAagauccaaucu....................   1   1   S02
......................................................................auggacgguggagauccaauAu....................   3   1   S02
......................................................................auggacgguggagauGcaaucu....................   1   1   S02
......................................................................auggacgguggagauccaUucu....................   1   1   S02
......................................................................auggacgguggagauccaaucA....................   1   1   S02
......................................................................auggacgguggagGuccaaucu....................   1   1   S02
......................................................................augUacgguggagauccaaucu....................   1   1   S02
......................................................................auggacgguggagauccaaucC....................   2   1   S02
......................................................................auggacggugCagauccaaucu....................   1   1   S02
......................................................................auggacgguggagauccaaucG....................   1   1   S02
......................................................................auggGcgguggagauccaaucu....................   1   1   S02
......................................................................auggacgguggagauccaaucug...................   1   0   S02
......................................................................auggacgguggagauccaauAug...................   1   1   S02
......................................................................auggacggugUagauccaaucugg..................   1   1   S02
......................................................................auggaGgguggagauccaaucugg..................   1   1   S02
......................................................................auggacgguggagaucGaaucugg..................   1   1   S02
......................................................................augUacgguggagauccaaucugg..................   1   1   S02
......................................................................auggacgguggagauccaCucugg..................   1   1   S02
......................................................................auggacgguggagauAcaaucugg..................   1   1   S02
......................................................................auggacgguggagauccaaucugA..................   10  1   S02
......................................................................auggacggugAagauccaaucugg..................   1   1   S02
......................................................................augAacgguggagauccaaucugg..................   4   1   S02
......................................................................auggacgguggagauUcaaucugg..................   1   1   S02
......................................................................auggacgguggagauccaUucugg..................   20  1   S02
......................................................................auggacgguggagauccaauAugg..................   22  1   S02
......................................................................auggacAguggagauccaaucugg..................   2   1   S02
......................................................................auggacggugCagauccaaucugg..................   1   1   S02
......................................................................auggacgguggagauccaaucugg..................   91  0   S02
......................................................................auggacgguggagauccaaucugU..................   1   1   S02
......................................................................auggacgguggaAauccaaucugg..................   1   1   S02
......................................................................auggacgguggagauccaaucuggC.................   1   1   S02
......................................................................auggacgguggagauccaaucuggU.................   3   1   S02
......................................................................auggacgguggagauccaaucuggUu................   1   1   S02
......................................................................auggacgguggagauccaaucuggaA................   1   1   S02
.......................................................................uggacgguggagauccaC.......................   1   1   S02
.......................................................................uggacggugAagauccaaucu....................   1   1   S02
.......................................................................uggacgguggagauccGaucu....................   1   1   S02
.......................................................................ugAacgguggagauccaaucu....................   1   1   S02
.......................................................................uggacgguggagauccaaucu....................   1   0   S02
.......................................................................uggacgguggagauccaaucuU...................   1   1   S02
.......................................................................uggacgguggagauccaaucug...................   1   0   S02
.......................................................................uggacgguggagauccaaucugg..................   1   0   S02
.......................................................................uggacgguggagauccaUucugg..................   1   1   S02
........................................................................Agacgguggagauccaaucuggau................   1   1   S02
.........................................................................Aacgguggagauccaaucu....................   3   1   S02
.........................................................................gacggugUagauccaaucu....................   1   1   S02
.........................................................................gacgguggagauccaUucu....................   12  1   S02
.........................................................................gacgguggagauccaaucu....................   1   0   S02
.........................................................................gacgguggagauccaauAu....................   1   1   S02
.........................................................................gacgguggagauccaaucA....................   1   1   S02
.........................................................................gacggugAagauccaaucu....................   1   1   S02
.........................................................................gacgguggagauccaauAug...................   1   1   S02
.........................................................................gacgguggagauccaUucug...................   1   1   S02
.........................................................................Aacgguggagauccaaucug...................   2   1   S02
.........................................................................gacgguggagauccaUucugg..................   61  1   S02
.........................................................................gacgguggagauccaauUugg..................   1   1   S02
.........................................................................gacggugAagauccaaucugg..................   1   1   S02
.........................................................................Aacgguggagauccaaucugg..................   8   1   S02
.........................................................................gacgguggagaCccaaucugg..................   1   1   S02
.........................................................................gacgguggagauccaauAugg..................   1   1   S02
.........................................................................gacgguggaUauccaaucugg..................   1   1   S02
.........................................................................gacgguggagauccaaucugg..................   12  0   S02
.........................................................................gacgguggagauccaaucuggauU...............   3   1   S02
.........................................................................gacgguggagauccaaucuggauc...............   8   0   S02
```

```
                                        Star                        Mature
uuugauuuuuuuuauaaagagauagauuugguggaaguccauauuggauuuccaccguucaucuaaugagauggacgguggagauccaaucuggaucuccaacaaauccaaa
.........................................................................gacgguggagauccGaucuggauc...............     1     1     S02
.........................................................................gacgguggagauccaaucuggaCc...............     1     1     S02
.........................................................................gacggugUagauccaaucuggauc...............     1     1     S02
..........................................................................acgguggagauccaUucugg..................     1     1     S02
..........................................................................acgguggagauccaaucugg..................     1     0     S02
..........................................................................acgguggagauccaauAugga.................     1     1     S02
..........................................................................acgguggagauccaaucuggauc...............     1     0     S02
..........................................................................acgguggagaucAaaucuggaucu..............     2     1     S02
..........................................................................acgguggagauUcaaucuggaucu..............     8     1     S02
..........................................................................acgguggagauccaaucuggaucu..............     1     0     S02
..........................................................................acgguggagauccaaucuggaucC..............     2     1     S02
..........................................................................acgguggagauccaaucuggauAuc.............     1     1     S02
...........................................................................cgguggagaucAaaucugg..................     1     1     S02
...........................................................................cgguggagauccaaucuggaG................     1     1     S02
...........................................................................cggugUagauccaaucuggaucu..............     1     1     S02
............................................................................gguggagauccaaucuggaG................     1     1     S02
....................................................................................uccaaucuggaucuccaCc.........     1     1     S02
```

Provisional ID	:	Maple_Unigene_BMK.14174_28324
Score total	:	0.6
Score for star read(s)	:	-1.3
Score for read counts	:	0
Score for mfe	:	2.5
Score for randfold	:	0
Score for cons. seed	:	-0.6
Total read count	:	204
Mature read count	:	188
Loop read count	:	0
Star read count	:	16

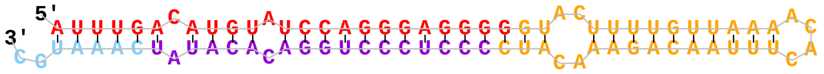

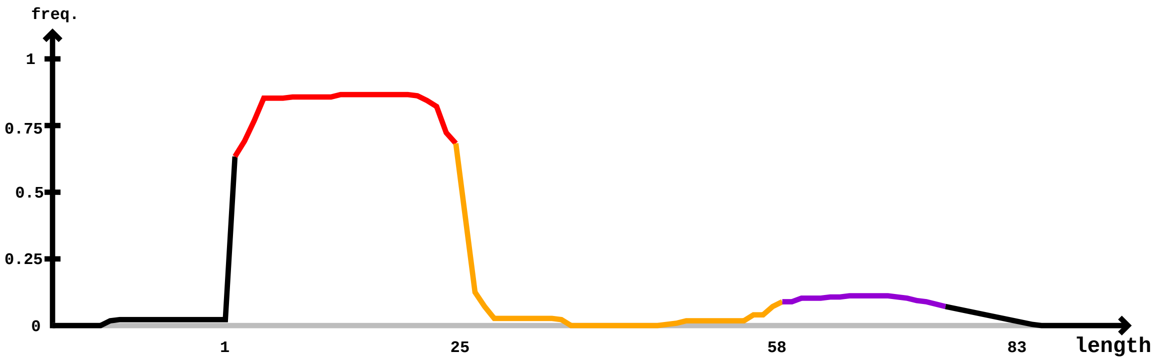


```
                Mature                                             Star
5'- uuaccuuccuccccaacauuugacauguauccagggaggggguacuuuuguuaaaacacuuuaacagaaacauccccucccuggacacauaucaaaugcuaggaagggag  -3'   obs
    uuaccuuccuccccaacauuugacauguauccagggaggggguacuuuuguuaaaacacuuuaacagaaacaucccucccuggacacauaucaaaugcuaggaagggag         exp
    ...(((((((........(((((((.((((.(((((((((((((..((((((((((....))))))))))..))))))))))))).)))).)))))))..)))))))...   reads   mm   sample
    ....cuuccuccccaacaCuugacaugu..................................................................................   2       1    S01
    .....Cuccuccccaacauuugac......................................................................................   1       1    S01
    .................aCuugacauguauccaggg..........................................................................   1       1    S01
    .................aCuugacauguauccaggga.........................................................................   1       1    S01
    .................aCuugacauguauccagggag........................................................................   3       1    S01
    .................aCuugacauguauccagggagg.......................................................................   6       1    S01
    .................aCuugacauguauccagggaggg......................................................................   6       1    S01
    .................aCuugacauguauccagggagggg.....................................................................   75      1    S01
    .................auuugacauguGuccagggagggg.....................................................................   1       1    S01
    ..................Cuugacauguauccaggga.........................................................................   1       1    S01
    ..................Cuugacauguauccagggagg.......................................................................   5       1    S01
    ...................uugacauguGuccaggga.........................................................................   1       1    S01
    ...................uugacauguGuccagggag........................................................................   1       1    S01
    ...................uugacauguauccagggag........................................................................   1       0    S01
    ...................uugacauguauccagggagg.......................................................................   1       0    S01
    ...................uugacauguGuccagggaggg......................................................................   1       1    S01
    ...................uugacauguauccagggagggg.....................................................................   1       0    S01
    ...................uugacauguauccagggaggggg....................................................................   1       0    S01
    ...................uugacauguGuccagggagggggu...................................................................   4       1    S01
    ....................ugacauguauccagggagggg.....................................................................   2       0    S01
    ....................ugacauguGuccagggagggggu...................................................................   1       1    S01
    ....................ugacauguauccagggagggggua..................................................................   1       0    S01
    ....................ugacauguauccagggaggggguG..................................................................   8       1    S01
    .......................cauguauccagggaggggguG..................................................................   1       1    S01
    ............................auccagggaggggguGcuuuuguu..........................................................   4       1    S01
    .......................................................................aCccccucccuggacacau....................   1       1    S01
    .......................................................................auccccucccuggacacauGu..................   1       1    S01
    .........................................................................ccccucccuggacacauG...................   1       1    S01
    .........................................................................ccccucUcuggacacaua...................   1       1    S01
    .........................................................................ccccucccuggacacauGu..................   2       1    S01
    .........................................................................ccccucccuggacacauGuca................   1       1    S01
    .........................................................................ccccucccuggacacauaucaaGu.............   1       1    S01
    ..........................................................................cccucccuggacacauGuca................   1       1    S01
```

Mature Star

```
uuaccuuccuccccaacauuugacauguauccagggaggggguacuuuuguuaaaacacuuuaacagaaacauccccucccuggacacauaucaaaugcuaggaagggag

....cuuccuccccaacaCuugacaug...................................................................................  1   1   S02
....cuuccuccccaacaCuugacaugu..................................................................................  1   1   S02
.................aCuugacauguauccaggga.........................................................................  1   1   S02
.................aCuugacauguauccagggagg.......................................................................  2   1   S02
.................aCuugacauguauccagggaggg......................................................................  2   1   S02
.................aCuugacauguauccagggagggg.....................................................................  38  1   S02
.................aCuugacauguauccagggaggggg....................................................................  1   1   S02
..................Cuugacauguauccagggagg.......................................................................  7   1   S02
...................uugacauguGuccagggagg.......................................................................  1   1   S02
...................uugacauguGuccagggagggggu...................................................................  4   1   S02
...................uugacauguauccagggagggggu...................................................................  1   0   S02
....................ugacauguauccagggagggA.....................................................................  1   1   S02
....................ugacauguauccagggaggCg.....................................................................  1   1   S02
....................ugacauguauccagggagggg.....................................................................  2   0   S02
....................ugacauguauccagggaggggg....................................................................  1   0   S02
....................ugacauguaucUagggagggggu...................................................................  2   1   S02
........................auguGuccagggaggggg....................................................................  1   1   S02
...........................uauccaggAaggggguacuuuugu...........................................................  1   1   S02
............................auccagggaggggguGcuuuuguu..........................................................  1   1   S02
..............................................................aacagaaUcauccccucccuggaca.......................  1   1   S02
...............................................................acagaaacauccccucccuggac........................  1   0   S02
................................................................cagaaUcauccccucccuggacac......................  1   1   S02
................................................................cagaaacaCccccucccuggacac......................  1   1   S02
.......................................................................aCccccucccuggacaca.....................  1   1   S02
.......................................................................auccccucccuggacacUu....................  1   1   S02
.......................................................................auccccucUcuggacacauauca................  1   1   S02
.........................................................................ccccucccuggacacauaucaaGu.............  1   1   S02
..........................................................................cccucccuggacacauGu..................  3   1   S02
............................................................................cucUcuggacacauauca................  1   1   S02
............................................................................cucccuggacacauGucaa...............  1   1   S02
............................................................................cucccuggacacauGucaaaugc...........  1   1   S02
...............................................................................ccuggacacauaucaaauAcu..........  1   1   S02
.................................................................................uggacacauaucaaaugcuG.........  1   1   S02
```

Provisional ID	:	Maple_Unigene_BMK.14279_29227
Score total	:	0.5
Score for star read(s)	:	3.9
Score for read counts	:	-4.9
Score for mfe	:	-1.3
Score for randfold	:	0
Score for cons. seed	:	3
Total read count	:	2
Mature read count	:	1
Loop read count	:	0
Star read count	:	1

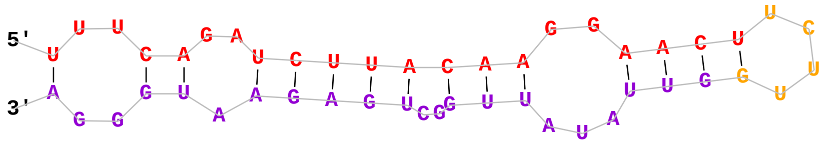

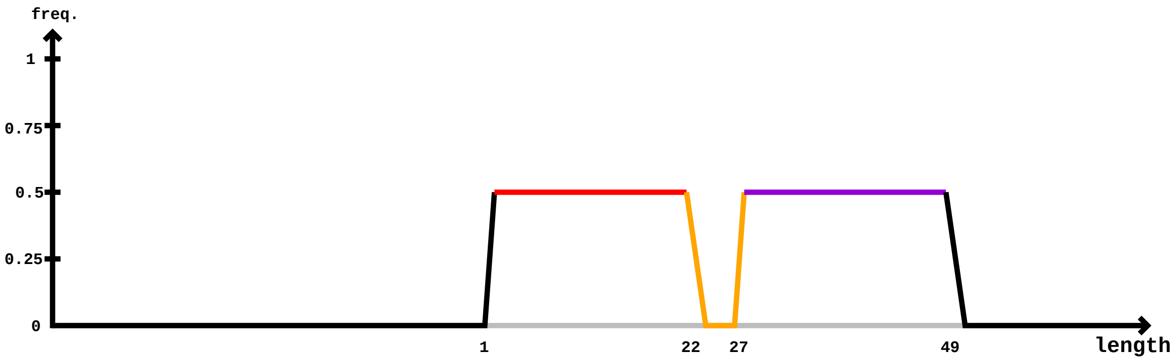

Mature Star

```
5'- uacaagaguuaaggccaucaaugaaguuugugcucaagccaaauuuucagaucuuacaaggaacuucuugguuauauuggcugagaaugggagguugagacacauagaccuc   -3'   obs
    uacaagaguuaaggccaucaaugaaguuugugcucaagccaaauuuucagaucuuacaaggaacuucugguuauauuggcugagaauggggagguugagacacauagaccuc         exp
    ......................((.((((((((((((.((..((((((((......(((..((((....))))...))).))))))))..).).))))).))))..))).))   reads   mm   sample
    ............................................uuucagaucuuacaaggaacu...............................................   1       0    S01

    ..................................................................guuauauuggcugagaauggga..........................   1       0    S02
```

Provisional ID	:	Maple_Unigene_BMK.14564_31162
Score total	:	0
Score for star read(s)	:	3.9
Score for read counts	:	-4.9
Score for mfe	:	-2
Score for randfold	:	0
Score for cons. seed	:	3
Total read count	:	2
Mature read count	:	1
Loop read count	:	0
Star read count	:	1

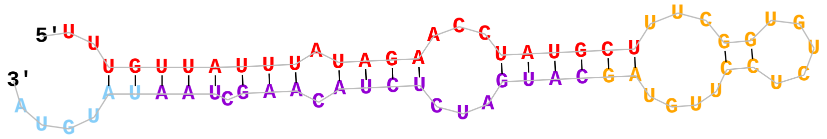

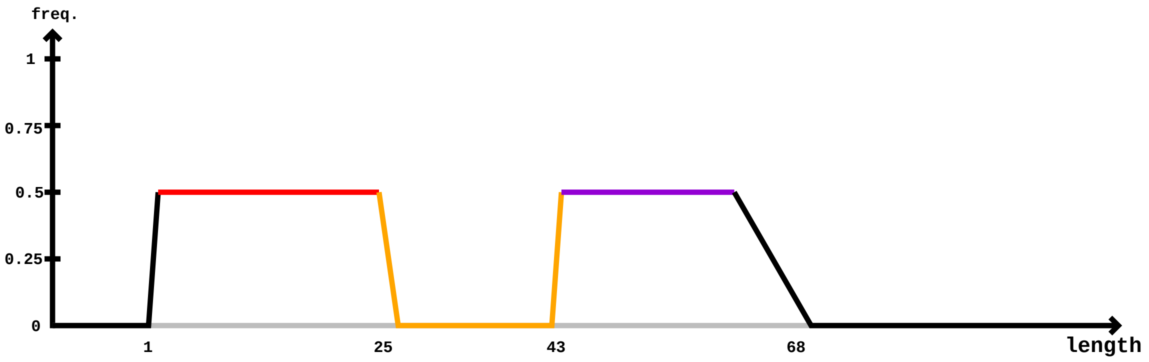

Mature **Star**

```
5'- aucaugugauuuguuauuuauagaaccuaugcuuucggugucuccuuguagcaugaucucuacaagcuaauauguaacuuuuggugagggguuauagaugucucaccuc   -3'    obs
    aucaugugauuuguuauuuauagaaccuaugcuuucggugucuccuuguagcaugaucucuacaagcuaauauguaacuuuuggugagggguuauagaugucucaccuc          exp
    .....((....((((((((.((((...((((((...((.....))....))))))...)))).))).)))))....))....(((((((.(((...))).)))))))..   reads      mm        sample
    ...................................................caugaucucuacaagcuaa.......................................   1          0         S01

    .........uuuguuauuuauagaaccuaugcu............................................................................   1          0         S02
```

Provisional ID	:	Maple_Unigene_BMK.14656_31955
Score total	:	0.5
Score for star read(s)	:	-1.3
Score for read counts	:	-0.3
Score for mfe	:	-0.7
Score for randfold	:	0
Score for cons. seed	:	3
Total read count	:	11
Mature read count	:	11
Loop read count	:	0
Star read count	:	0

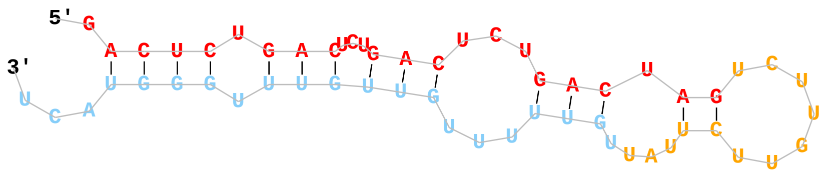

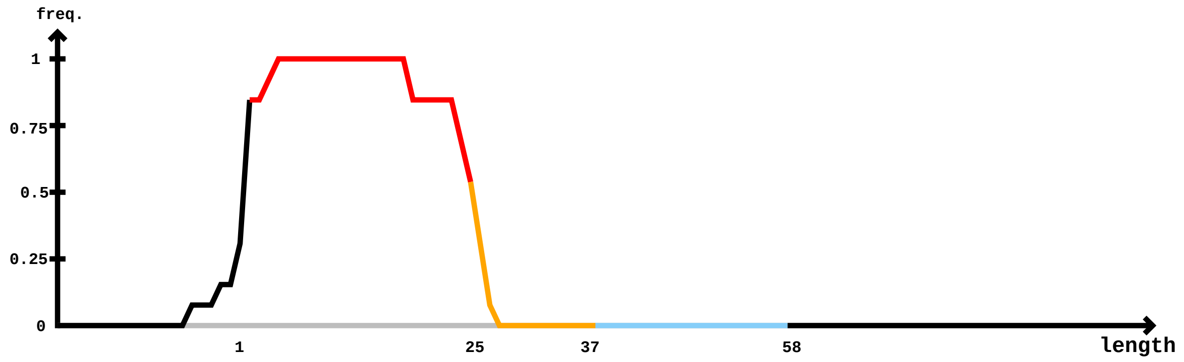

Mature Star

```
5'- caugcucuaccacacucugacucugacucugacucugacuagucuuguucuuauuguuuuuguuguuuggguacuggugguggccgugguccuuugaugacaaugaggacau  -3'   exp
    ((((..(((((((......((((.(((...(((...(((.((.......))....)))...)))))).))))....))))))).))))((((((..........))))))..   reads   mm   sample
    ............Gacucugacucugacucugacuc.............................................................................   1       1    S01
    .................Cgacucugacucugacucugacu........................................................................   2       1    S01
    ..................gacucugacucugacucugacuC.......................................................................   1       1    S01
    ..................gacucugacucugacucugacuGg......................................................................   5       1    S01
    ....................cucugacucugacucugacuaguc....................................................................   1       0    S01

    ...............ucugacucugacucugacuG.............................................................................   2       1    S02
    ..................gacucugacucugacucugacuGg......................................................................   1       1    S02
    .....................ucugacucugacucugacuG.......................................................................   2       1    S02
```

Provisional ID	:	Maple_Unigene_BMK.14893_32649
Score total	:	17411.7
Score for star read(s)	:	3.9
Score for read counts	:	17403
Score for mfe	:	1.8
Score for randfold	:	0
Score for cons. seed	:	3
Total read count	:	34147
Mature read count	:	27761
Loop read count	:	0
Star read count	:	6386

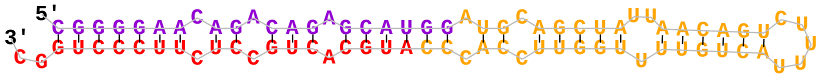

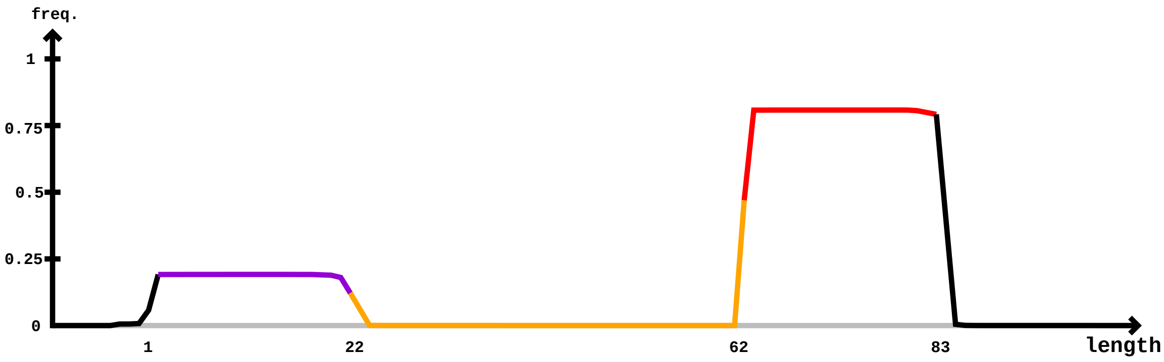

Star Mature

```
5'- agacaaagacggggaacagacagagcauggaugcagcuauuaacagucuuuuacuguuuugguuccacccaugcacugccucuucccuggcucugacuccuuucucuccuc   -3'   obs
    agacaaagacggggaacagacagagcauggaugcagcuauuaacagucuuuuacuguuuugguuccacccaugcacugccucuucccuggcucugacuccuuucucuccuc         exp
    ...((.((.(((((((.((.(((.((((((.((.(((((..((((((.....)))))).))))).)).)))))).))).)).))))))).)).))................   reads   mm   sample
    .....aagacggggaacagacag........................................................................................   2       0    S01
    .....aagacggggaacagacaga.......................................................................................   2       0    S01
    .....aagacggggaacagacagagA.....................................................................................   1       1    S01
    .....aagacggggaacagacagagU.....................................................................................   1       1    S01
    .....aagacggggaacagacagagc.....................................................................................   6       0    S01
    .....aagacggggaacagacagagca....................................................................................   2       0    S01
    .....aagacggggaacagacagagcau...................................................................................   1       0    S01
    .....aagacggggaacagacGgagcaug..................................................................................   1       1    S01
    .....aagacggggaacagGcagagcaug..................................................................................   1       1    S01
    .....aagacggggaacUgacagagcaug..................................................................................   1       1    S01
    .....aagacgggAaacagacagagcaug..................................................................................   1       1    S01
    .....aagacgggUaacagacagagcaug..................................................................................   2       1    S01
    .....aagacggggaacagacagagcGug..................................................................................   1       1    S01
    .....aagacggAgaacagacagagcaug..................................................................................   2       1    S01
    .....aagacggggaacagacagagcauA..................................................................................   4       1    S01
    .....aagacggggaacagacagagcaug..................................................................................   103     0    S01
    .....Nagacggggaacagacagagcaug..................................................................................   1       1    S01
    .....aUgacggggaacagacagagcaug..................................................................................   1       1    S01
    ......agacggggaacagacagagcau...................................................................................   1       0    S01
    ......agacggggaacagacagagcaugga................................................................................   1       0    S01
    .......gacggggaacagacagagc.....................................................................................   1       0    S01
    .......gacggggaacagacagagca....................................................................................   2       0    S01
    .......gacggggaacagacagagcau...................................................................................   2       0    S01
    .......gacggggaacagacagagcaug..................................................................................   31      0    S01
    .......gacggggaacagacaAagcaug..................................................................................   1       1    S01
    .......gacAgggaacagacagagcaug..................................................................................   1       1    S01
    .......gacggggaacagacagagcauA..................................................................................   3       1    S01
    .......gacggggaacagacagagcauU..................................................................................   1       1    S01
    .......gacggggGacagacagagcaug..................................................................................   1       1    S01
    ........acggggaacagacUgagc.....................................................................................   1       1    S01
    ........acggggaacagacagagU.....................................................................................   4       1    S01
    ........acggggaacagacagagc.....................................................................................   19      0    S01
    ........acggggGacagacagagc.....................................................................................   1       1    S01
```

```
         Star                                         Mature
agacaaagacggggaacagacagagcauggaugcagcuauuaacagucuuuuacuguuuugguuccacccaugcacugccucuucccuggcucugacuccuuucucuccuc
........acggggaacagacagagca....................................................................................   15    0  S01
........acggggaacagacagagcaC...................................................................................   1     1  S01
........acggggaacagacGgagcau...................................................................................   1     1  S01
........acggggaacagacagagcau...................................................................................   11    0  S01
........acAgggaacagacagagcaug..................................................................................   3     1  S01
........acggggaacagacagagcGug..................................................................................   7     1  S01
........acggggaacagacagaUcaug..................................................................................   2     1  S01
........acggggaacagacagagcCug..................................................................................   1     1  S01
........acggggaacagacagagGaug..................................................................................   2     1  S01
........acggggaacagacagagcauU..................................................................................   7     1  S01
........acgggAaacagacagagcaug..................................................................................   1     1  S01
........acggggaacagacagagcauA..................................................................................   53    1  S01
........acggggaaAagacagagcaug..................................................................................   1     1  S01
........acggggCacagacagagcaug..................................................................................   3     1  S01
........acggggaacagacagCgcaug..................................................................................   2     1  S01
........acggggaacagaGagagcaug..................................................................................   1     1  S01
........aUggggaacagacagagcaug..................................................................................   5     1  S01
........acgCggaacagacagagcaug..................................................................................   1     1  S01
........acggggaacGgacagagcaug..................................................................................   5     1  S01
........Ucggggaacagacagagcaug..................................................................................   1     1  S01
........acggggaacagCcagagcaug..................................................................................   1     1  S01
........aAggggaacagacagagcaug..................................................................................   1     1  S01
........acggggaaGagacagagcaug..................................................................................   1     1  S01
........acggggaacagGcagagcaug..................................................................................   4     1  S01
........Gcggggaacagacagagcaug..................................................................................   5     1  S01
........acggggaacagaAagagcaug..................................................................................   2     1  S01
........acggggaacagacaAagcaug..................................................................................   4     1  S01
........acggggaGcagacagagcaug..................................................................................   8     1  S01
........acggggaacagacagagcaug..................................................................................   1046  0  S01
........acggggaacagacaUagcaug..................................................................................   1     1  S01
........acggggaacagacagUgcaug..................................................................................   1     1  S01
........acggggaacagacagGgcaug..................................................................................   5     1  S01
........acggggaacagacagagUaug..................................................................................   2     1  S01
........acggggaacaCacagagcaug..................................................................................   1     1  S01
........acggggaacaAacagagcaug..................................................................................   2     1  S01
........acggggaacagaUagagcaug..................................................................................   1     1  S01
........acggggGacagacagagcaug..................................................................................   1     1  S01
........Ncggggaacagacagagcaug..................................................................................   1     1  S01
........acgUggaacagacagagcaug..................................................................................   5     1  S01
........acggggaacagacagaAcaug..................................................................................   1     1  S01
........acgggCaacagacagagcaug..................................................................................   2     1  S01
........acggggaacagacGgagcaug..................................................................................   4     1  S01
........acggggaacagacagagcaGg..................................................................................   1     1  S01
........acgAggaacagacagagcaug..................................................................................   2     1  S01
........acggggaacagacCgagcaug..................................................................................   1     1  S01
........acggggaacagacagagcaCg..................................................................................   11    1  S01
........acggggaacagacagagcaugg.................................................................................   5     0  S01
........Ccggggaacagacagagcaugg.................................................................................   1     1  S01
........acggggaacagacagagcaugU.................................................................................   4     1  S01
........acggggaacagacagagcaugA.................................................................................   3     1  S01
........acggggaacagacagagcaugC.................................................................................   1     1  S01
........acggggaacagacagagcaugUa................................................................................   1     1  S01
........acggggaacagacagagcauggUu...............................................................................   1     1  S01
........acggggaacagacagagcauggaug..............................................................................   1     0  S01
........acggggaacagacagagcauggaugU.............................................................................   1     1  S01
.........cggggCacagacagagca....................................................................................   1     1  S01
.........cggggaacGgacagagca....................................................................................   1     1  S01
.........cggggaacagacagagca....................................................................................   16    0  S01
.........cggggaacagacagagcG....................................................................................   1     1  S01
.........cggggaacagacagaUcau...................................................................................   1     1  S01
.........cggggaacagacagagcaG...................................................................................   7     1  S01
.........cggggCacagacagagcau...................................................................................   1     1  S01
.........cggggaacagacagagAau...................................................................................   1     1  S01
.........cggggaacagGcagagcau...................................................................................   1     1  S01
.........cggggaacagacagagcaC...................................................................................   15    1  S01
.........cggUgaacagacagagcau...................................................................................   2     1  S01
.........cggggaGcagacagagcau...................................................................................   1     1  S01
.........cggggaacagacagagcau...................................................................................   169   0  S01
.........cggggaaGagacagagcau...................................................................................   1     1  S01
.........cggggaacagacUgagcau...................................................................................   1     1  S01
```

Star **Mature**

```
agacaaagacggggaacagacagagcauggaugcagcuauuaacagucuuuuacuguuuugguuccacccaugcacugccucuucccuggcucugacuccuuucucuccuc
.........cggggaacagacagagcaA...................................................................................  5    1  S01
.........cggggaacagacGgagcau...................................................................................  1    1  S01
.........cggggaacagacGgagcaug..................................................................................  2    1  S01
.........cggggaacagacagagcGug..................................................................................  2    1  S01
.........cggggaacagacUgagcaug..................................................................................  1    1  S01
.........cggAgaacagacagagcaug..................................................................................  1    1  S01
.........cggggaacagacagGgcaug..................................................................................  2    1  S01
.........cggggaacagacagagcaGg..................................................................................  1    1  S01
.........cggggaacagacagagcaCg..................................................................................  1    1  S01
.........cggggaacagacagagcauU..................................................................................  5    1  S01
.........Aggggaacagacagagcaug..................................................................................  1    1  S01
.........cggggCacagacagagcaug..................................................................................  1    1  S01
.........cggggGacagacagagcaug..................................................................................  1    1  S01
.........cggggaacagacagagcaug..................................................................................  106  0  S01
.........cggggaacagacagagcauA..................................................................................  6    1  S01
.........cggggaGcagacagagcaug..................................................................................  3    1  S01
.........cggggaacagacUgagcaugg.................................................................................  4    1  S01
.........cggggaacagUcagagcaugg.................................................................................  4    1  S01
.........cggggaacagaAagagcaugg.................................................................................  1    1  S01
.........cggCgaacagacagagcaugg.................................................................................  2    1  S01
.........cgAggaacagacagagcaugg.................................................................................  3    1  S01
.........cggggaacagacagagcaugA.................................................................................  190  1  S01
.........cggggaacagaGagagcaugg.................................................................................  1    1  S01
.........Aggggaacagacagagcaugg.................................................................................  2    1  S01
.........cggggaacagacGgagcaugg.................................................................................  12   1  S01
.........cggggaacagaUagagcaugg.................................................................................  4    1  S01
.........cggggaacagacagagcaugU.................................................................................  35   1  S01
.........cggggaacUgacagagcaugg.................................................................................  5    1  S01
.........cggggaaAagacagagcaugg.................................................................................  9    1  S01
.........cgggAaacagacagagcaugg.................................................................................  2    1  S01
.........cggggaacaUacagagcaugg.................................................................................  2    1  S01
.........cggggaacagacagagcaGgg.................................................................................  5    1  S01
.........cggggaacagGcagagcaugg.................................................................................  10   1  S01
.........cggggaaGagacagagcaugg.................................................................................  2    1  S01
.........cggggaacagacagagUaugg.................................................................................  2    1  S01
.........cggggaaUagacagagcaugg.................................................................................  5    1  S01
.........cggggaacagacagagcGugg.................................................................................  10   1  S01
.........cgUggaacagacagagcaugg.................................................................................  1    1  S01
.........cCgggaacagacagagcaugg.................................................................................  5    1  S01
.........cggggaacagacagagcCugg.................................................................................  3    1  S01
.........cgggUaacagacagagcaugg.................................................................................  1    1  S01
.........cggggUacagacagagcaugg.................................................................................  4    1  S01
.........cggggaacagacagagcauAg.................................................................................  2    1  S01
.........cggggaacagacagagcaugC.................................................................................  16   1  S01
.........cggggaacagacagUgcaugg.................................................................................  4    1  S01
.........cggggaacagacaAagcaugg.................................................................................  3    1  S01
.........cggggaacagacagagcaAgg.................................................................................  4    1  S01
.........Nggggaacagacagagcaugg.................................................................................  1    1  S01
.........cggggaacagacagaCcaugg.................................................................................  1    1  S01
.........cUgggaacagacagagcaugg.................................................................................  1    1  S01
.........cggggaacaAacagagcaugg.................................................................................  2    1  S01
.........cggUgaacagacagagcaugg.................................................................................  17   1  S01
.........cggggaacagacaUagcaugg.................................................................................  1    1  S01
.........cggggaacagacagagcauUg.................................................................................  1    1  S01
.........Gggggaacagacagagcaugg.................................................................................  2    1  S01
.........cggggaacagacagagcUugg.................................................................................  1    1  S01
.........cggggaacagacagaUcaugg.................................................................................  2    1  S01
.........cggggCacagacagagcaugg.................................................................................  2    1  S01
.........cggggaacagacagagGaugg.................................................................................  5    1  S01
.........cggggaacagacagagcauCg.................................................................................  8    1  S01
.........cgggCaacagacagagcaugg.................................................................................  1    1  S01
.........cAgggaacagacagagcaugg.................................................................................  2    1  S01
.........cggggGacagacagagcaugg.................................................................................  17   1  S01
.........Uggggaacagacagagcaugg.................................................................................  4    1  S01
.........cggggaGcagacagagcaugg.................................................................................  7    1  S01
.........cggggaacagacagaAcaugg.................................................................................  7    1  S01
.........cggggaacagacagGgcaugg.................................................................................  14   1  S01
.........cggAgaacagacagagcaugg.................................................................................  5    1  S01
.........cggggaUcagacagagcaugg.................................................................................  1    1  S01
.........cggggaacGgacagagcaugg.................................................................................  10   1  S01
```

Star **Mature**

```
agacaaagacggggaacagacagagcauggaugcagcuauuaacagucuuuuacuguuuugguuccacccaugcacugccucuucccuggcucugacuccuuucucuccuc
.........cggggaacagacCgagcaugg.................................................................................  3     1  S01
.........cggggaacagacagagcaCgg.................................................................................  6     1  S01
.........cggggaacagacagagcaugg.................................................................................  2752  0  S01
.........cggAgaacagacagagcaugga................................................................................  1     1  S01
.........cggggaacagacagagcauggG................................................................................  2     1  S01
.........cggggaaUagacagagcaugga................................................................................  1     1  S01
.........cggggaacagacagagcauggU................................................................................  9     1  S01
.........cggggaacagacagagcaugga................................................................................  5     0  S01
.........cggggaacagacagagcauggC................................................................................  2     1  S01
.........cggggaacagacagagcauggUu...............................................................................  2     1  S01
.........cggggaacagacagagcauggau...............................................................................  2     0  S01
.........cggggaacagacagagcauggaug..............................................................................  1     0  S01
..........Cgggaacagacagagcaug..................................................................................  1     1  S01
..........Cgggaacagacagagcaugg.................................................................................  1     1  S01
............ggaacagacagagcaugA.................................................................................  1     1  S01
............ggaacagacagagcaugg.................................................................................  1     0  S01
............ggaacagacagagcauggaug..............................................................................  1     0  S01
................cagacagagcauggaugcagc..........................................................................  1     0  S01
....................cagagcauggaugcagcuauuaaca..................................................................  1     0  S01
...........................uggaugcagcuauuaaca..................................................................  1     0  S01
....................................................................cAaugcacugccucuuccc........................  1     1  S01
.....................................................................caugcacugccucuucccuggc....................  3     0  S01
......................................................................augcacugccucuucccA.......................  1     1  S01
......................................................................augcacuUccucuucccu.......................  1     1  S01
......................................................................augUacugccucuucccu.......................  1     1  S01
......................................................................augcacugccucuCcccu.......................  1     1  S01
......................................................................augcacugccucuucccu.......................  55    0  S01
......................................................................augcacugUcucuucccu.......................  1     1  S01
......................................................................augcacugccucuucccC.......................  6     1  S01
......................................................................augcacugccucuucccG.......................  2     1  S01
......................................................................augcacCgccucuucccu.......................  1     1  S01
......................................................................augcacugccucuucccuU......................  3     1  S01
......................................................................augcacugccucuucccCg......................  1     1  S01
......................................................................augcacugccucuucccug......................  9     0  S01
......................................................................augcacuUccucuucccug......................  1     1  S01
......................................................................augcacugccucuucccuA......................  2     1  S01
......................................................................augcacugccucuucccGgg.....................  1     1  S01
......................................................................augcacugccucuucccugU.....................  3     1  S01
......................................................................augcacugccucuucccugC.....................  4     1  S01
......................................................................augcacugccucuucccugg.....................  19    0  S01
......................................................................Gugcacugccucuucccuggc....................  35    1  S01
......................................................................augcacugccucuucccCggc....................  36    1  S01
......................................................................augcacugccucuAcccuggc....................  10    1  S01
......................................................................augcaUugccucuucccuggc....................  10    1  S01
......................................................................augcacugccucGucccuggc....................  3     1  S01
......................................................................augcacugccucuuGccuggc....................  6     1  S01
......................................................................aGgcacugccucuucccuggc....................  6     1  S01
......................................................................augcacuUccucuucccuggc....................  14    1  S01
......................................................................augcacugccuUuucccuggc....................  6     1  S01
......................................................................augcacugccucuucGcuggc....................  13    1  S01
......................................................................augGacugccucuucccuggc....................  21    1  S01
......................................................................augcacugccucuucccuggU....................  1680  1  S01
......................................................................augcacugccucuucccuggA....................  386   1  S01
......................................................................Nugcacugccucuucccuggc....................  6     1  S01
......................................................................augcacugccucuucccGggc....................  8     1  S01
......................................................................augcGcugccucuucccuggc....................  36    1  S01
......................................................................augcacugccucCucccuggc....................  51    1  S01
......................................................................augcacugccucuucAcuggc....................  10    1  S01
......................................................................augcaGugccucuucccuggc....................  18    1  S01
......................................................................augcCcugccucuucccuggc....................  12    1  S01
......................................................................augcacugccucuuccGuggc....................  10    1  S01
......................................................................augcacugccucuuccAuggc....................  10    1  S01
......................................................................Cugcacugccucuucccuggc....................  5     1  S01
......................................................................augcacGgccucuucccuggc....................  9     1  S01
......................................................................augcacCgccucuucccuggc....................  37    1  S01
......................................................................auAcacugccucuucccuggc....................  22    1  S01
......................................................................augcacugccucuuAccuggc....................  6     1  S01
......................................................................augcacugcGucuucccuggc....................  19    1  S01
......................................................................augcacugcUucuucccuggc....................  23    1  S01
......................................................................augcacugccucuucccuCgc....................  7     1  S01
```

```
         Star                                                           Mature
agacaaagacggggaacagacagagcaugguaugcagcuauuaacagucuuuuacuguuuugguuccacccaugcacugccucuucccuggcucugacuccuuucucuccuc
......................................................................augcacugcAucuucccuggc....................    15      1    S01
......................................................................aCgcacugccucuucccuggc....................    40      1    S01
......................................................................aAgcacugccucuucccuggc....................    11      1    S01
......................................................................augcacugccucuucccAggc....................    14      1    S01
......................................................................augcacugccGcuucccuggc....................    10      1    S01
......................................................................augcacugccucuucccugCc....................    8       1    S01
......................................................................augcacAgccucuucccuggc....................    15      1    S01
......................................................................Uugcacugccucuucccuggc....................    7       1    S01
......................................................................augcacugccucuucccuAgc....................    20      1    S01
......................................................................augcacugccucuucccugUc....................    9       1    S01
......................................................................augcaAugccucuucccuggc....................    10      1    S01
......................................................................augcacugccuAuucccuggc....................    12      1    S01
......................................................................augcacugAcucuucccuggc....................    19      1    S01
......................................................................augcacugccucuucccuggc....................    10460   0    S01
......................................................................auUcacugccucuucccuggc....................    3       1    S01
......................................................................augcacugccucuucccuggG....................    39      1    S01
......................................................................augcacugccucuuUccuggc....................    10      1    S01
......................................................................augcacugccucuuccUuggc....................    9       1    S01
......................................................................augcacugccucuGcccuggc....................    9       1    S01
......................................................................augcacugccucuucccugAc....................    25      1    S01
......................................................................augcacugccucuucccuUgc....................    7       1    S01
......................................................................augcacugccCcuucccuggc....................    38      1    S01
......................................................................augAacugccucuucccuggc....................    7       1    S01
......................................................................augcacugccAcuucccuggc....................    9       1    S01
......................................................................augcacugccucuCcccuggc....................    29      1    S01
......................................................................augcacugGcucuucccuggc....................    13      1    S01
......................................................................auCcacugccucuucccuggc....................    4       1    S01
......................................................................augcacugccucAucccuggc....................    7       1    S01
......................................................................augcacugccucuucUcuggc....................    15      1    S01
......................................................................augUacugccucuucccuggc....................    21      1    S01
......................................................................augcacugUcucuucccuggc....................    20      1    S01
......................................................................augcacuAccucuucccuggc....................    25      1    S01
......................................................................augcacuCccucuucccuggc....................    18      1    S01
......................................................................augcUcugccucuucccuggc....................    6       1    S01
......................................................................augcacugccuGuucccuggc....................    10      1    S01
......................................................................augcacugccucuucccuggcG...................    2       1    S01
......................................................................augcacugccucuucccuggcA...................    1       1    S01
......................................................................augcacugccucuucccuggcu...................    2       0    S01
......................................................................augcacugccucuucccuggcCcug................    1       1    S01
.......................................................................ugcacugccucuucccuU......................    26      1    S01
.......................................................................ugcaUugccucuucccug......................    2       1    S01
.......................................................................ugcacugAcucuucccug......................    1       1    S01
.......................................................................ugcacAgccucuucccug......................    1       1    S01
.......................................................................ugcacugcUucuucccug......................    1       1    S01
.......................................................................ugcacuAccucuucccug......................    1       1    S01
.......................................................................uAcacugccucuucccug......................    1       1    S01
.......................................................................Ggcacugccucuucccug......................    1       1    S01
.......................................................................ugcacuUccucuucccug......................    1       1    S01
.......................................................................ugcacugcAucuucccug......................    1       1    S01
.......................................................................ugcacugccucuucccug......................    122     0    S01
.......................................................................ugcacCgccucuucccug......................    3       1    S01
.......................................................................ugcacugccucuucccCg......................    1       1    S01
.......................................................................ugcacugccucCucccug......................    1       1    S01
.......................................................................Agcacugccucuucccug......................    1       1    S01
.......................................................................ugcacugccucuucccuA......................    8       1    S01
.......................................................................ugcacugccucuucccuC......................    7       1    S01
.......................................................................ugcacugccucuuccGug......................    1       1    S01
.......................................................................uAcacugccucuucccugg.....................    1       1    S01
.......................................................................ugcacugccucuucccuAg.....................    2       1    S01
.......................................................................ugcacugccucuucccugA.....................    9       1    S01
.......................................................................ugcacugccucuucccugU.....................    50      1    S01
.......................................................................ugcacAgccucuucccugg.....................    1       1    S01
.......................................................................ugcacugccucuCcccugg.....................    1       1    S01
.......................................................................ugcacugccAcuucccugg.....................    1       1    S01
.......................................................................ugcUcugccucuucccugg.....................    1       1    S01
.......................................................................ugcacugccucuucccugg.....................    92      0    S01
.......................................................................Ggcacugccucuucccugg.....................    1       1    S01
.......................................................................ugcacugccuGuucccugg.....................    1       1    S01
.......................................................................ugcacugccucuucccugC.....................    9       1    S01
.......................................................................ugcGcugccucuucccugg.....................    1       1    S01
```

```
         Star                                                       Mature
agacaaagacggggaacagacagagcaugg augcagcuauuaacagucuuuuacuguuuugguuccacccaugcacugccucuucccuggcucugacuccuuucucuccuc
.......................................................................ugcacugcUucuucccugg.....................   1     1   S01
.......................................................................ugcacugccucuAcccuggc....................   1     1   S01
.......................................................................ugUacugccucuucccuggc....................   10    1   S01
.......................................................................ugcacugccucuucccugAc....................   17    1   S01
.......................................................................ugcacugccucuuUccuggc....................   6     1   S01
.......................................................................ugcacugccucuucccGggc....................   4     1   S01
.......................................................................ugcacugccucuucccuUgc....................   4     1   S01
.......................................................................ugcacugccuAuucccuggc....................   5     1   S01
.......................................................................uUcacugccucuucccuggc....................   5     1   S01
.......................................................................ugcacugccucCucccuggc....................   18    1   S01
.......................................................................ugcacugccucGucccuggc....................   8     1   S01
.......................................................................ugGacugccucuucccuggc....................   5     1   S01
.......................................................................ugcacugccucuCcccuggc....................   16    1   S01
.......................................................................ugcGcugccucuucccuggc....................   32    1   S01
.......................................................................ugcacugcUucuucccuggc....................   5     1   S01
.......................................................................ugcacugccuGuucccuggc....................   3     1   S01
.......................................................................ugcUcugccucuucccuggc....................   7     1   S01
.......................................................................ugcacugccucuucGcuggc....................   3     1   S01
.......................................................................ugcacugcAucuucccuggc....................   7     1   S01
.......................................................................ugcaAugccucuucccuggc....................   2     1   S01
.......................................................................ugcacugccucuuGccuggc....................   7     1   S01
.......................................................................ugcacugccGcuucccuggc....................   6     1   S01
.......................................................................ugcacCgccucuucccuggc....................   17    1   S01
.......................................................................ugcacugccucuuAccuggc....................   9     1   S01
.......................................................................Cgcacugccucuucccuggc....................   18    1   S01
.......................................................................ugcCcugccucuucccuggc....................   8     1   S01
.......................................................................ugcacugccucuucccuggG....................   23    1   S01
.......................................................................ugAacugccucuucccuggc....................   7     1   S01
.......................................................................ugcacugccucuucccuggA....................   194   1   S01
.......................................................................ugcacugAcucuucccuggc....................   19    1   S01
.......................................................................ugcaUugccucuucccuggc....................   3     1   S01
.......................................................................ugcacugccucuucUcuggc....................   12    1   S01
.......................................................................uCcacugccucuucccuggc....................   4     1   S01
.......................................................................ugcacGgccucuucccuggc....................   3     1   S01
.......................................................................ugcacugccAcuucccuggc....................   8     1   S01
.......................................................................ugcacugccucuuccGuggc....................   10    1   S01
.......................................................................ugcacugccucuucccugCc....................   9     1   S01
.......................................................................ugcacuAccucuucccuggc....................   18    1   S01
.......................................................................ugcacAgccucuucccuggc....................   10    1   S01
.......................................................................ugcacugccucuucccuggU....................   890   1   S01
.......................................................................ugcacugccucuucccAggc....................   11    1   S01
.......................................................................ugcacugccCcuucccuggc....................   16    1   S01
.......................................................................ugcacugccucuGcccuggc....................   6     1   S01
.......................................................................ugcacugccucuucAcuggc....................   4     1   S01
.......................................................................ugcacugccucAucccuggc....................   6     1   S01
.......................................................................ugcacugcGucuucccuggc....................   4     1   S01
.......................................................................ugcacugccuUuucccuggc....................   9     1   S01
.......................................................................ugcacuUccucuucccuggc....................   9     1   S01
.......................................................................ugcacugccucuucccugUc....................   10    1   S01
.......................................................................ugcacugccucuucccCggc....................   20    1   S01
.......................................................................ugcacugGcucuucccuggc....................   8     1   S01
.......................................................................Ggcacugccucuucccuggc....................   5     1   S01
.......................................................................ugcacugccucuucccuCgc....................   2     1   S01
.......................................................................ugcacuCccucuucccuggc....................   5     1   S01
.......................................................................uAcacugccucuucccuggc....................   16    1   S01
.......................................................................ugcacugccucuuccAuggc....................   5     1   S01
.......................................................................ugcacugccucuucccuggc....................   5391  0   S01
.......................................................................ugcacugUcucuucccuggc....................   8     1   S01
.......................................................................ugcacugccucuucccuAgc....................   9     1   S01
.......................................................................Ngcacugccucuucccuggc....................   5     1   S01
.......................................................................Agcacugccucuucccuggc....................   12    1   S01
.......................................................................ugcacugccucuucccuAgcu...................   2     1   S01
.......................................................................ugcacugccCcuucccuggcu...................   4     1   S01
.......................................................................ugcacugccucuucAcuggcu...................   1     1   S01
.......................................................................ugcacugccuAuucccuggcu...................   2     1   S01
.......................................................................ugcacCgccucuucccuggcu...................   4     1   S01
.......................................................................ugcacugUcucuucccuggcu...................   4     1   S01
.......................................................................ugcacugccuGuucccuggcu...................   1     1   S01
.......................................................................Ggcacugccucuucccuggcu...................   3     1   S01
.......................................................................ugcacuUccucuucccuggcu...................   6     1   S01
```

```
         Star                                                     Mature
agacaaagacggggaacagacagagcauggaugcagcuauuaacagucuuuuacuguuuugguuccacccaugcacugccucuucccuggcucugacuccuuucucuccuc
.......................................................................ugcacugccucuuccGuggcu...................    3       1    S01
.......................................................................ugcacugccucuucccAggcu...................    4       1    S01
.......................................................................ugcacugccucuuUccuggcu...................    1       1    S01
.......................................................................ugcacugccGcuucccuggcu...................    1       1    S01
.......................................................................ugcacugccucuucUcuggcu...................    4       1    S01
.......................................................................ugcacugcGucuucccuggcu...................    3       1    S01
.......................................................................ugcacugcUucuucccuggcu...................    3       1    S01
.......................................................................ugcacugccucuucccugCcu...................    1       1    S01
.......................................................................ugcacugccucuAcccuggcu...................    3       1    S01
.......................................................................ugUacugccucuucccuggcu...................    3       1    S01
.......................................................................Ngcacugccucuucccuggcu...................    1       1    S01
.......................................................................uAcacugccucuucccuggcu...................    3       1    S01
.......................................................................ugcacugccucuucccuggcu...................    1479    0    S01
.......................................................................ugcGcugccucuucccuggcu...................    9       1    S01
.......................................................................ugcacugccucuucccugAcu...................    4       1    S01
.......................................................................ugcacugccucuuccAuggcu...................    3       1    S01
.......................................................................ugcacugAcucuucccuggcu...................    6       1    S01
.......................................................................ugcacugccuUuucccuggcu...................    1       1    S01
.......................................................................ugcacugccucuucccuggUu...................    9       1    S01
.......................................................................Agcacugccucuucccuggcu...................    4       1    S01
.......................................................................ugcaUugccucuucccuggcu...................    4       1    S01
.......................................................................uUcacugccucuucccuggcu...................    1       1    S01
.......................................................................ugcacugccucuuGccuggcu...................    1       1    S01
.......................................................................ugcacugccucuucccuggcG...................    47      1    S01
.......................................................................ugcCcugccucuucccuggcu...................    1       1    S01
.......................................................................ugcacugccucGucccuggcu...................    3       1    S01
.......................................................................ugcacugccucuucccuggcC...................    190     1    S01
.......................................................................ugcacugccucuCcccuggcu...................    1       1    S01
.......................................................................ugcacugccucuGcccuggcu...................    1       1    S01
.......................................................................ugcacuCccucuucccuggcu...................    3       1    S01
.......................................................................ugcacAgccucuucccuggcu...................    3       1    S01
.......................................................................ugcacugccucCucccuggcu...................    6       1    S01
.......................................................................ugcacuAccucuucccuggcu...................    7       1    S01
.......................................................................ugcacugccucuucccGggcu...................    2       1    S01
.......................................................................ugcacugccucuucccuggcA...................    72      1    S01
.......................................................................uCcacugccucuucccuggcu...................    1       1    S01
.......................................................................ugcacugccucuuccUuggcu...................    1       1    S01
.......................................................................ugcacugGcucuucccuggcu...................    1       1    S01
.......................................................................Cgcacugccucuucccuggcu...................    5       1    S01
.......................................................................ugcacugccucuuAccuggcu...................    3       1    S01
.......................................................................ugcacugccucuucccCggcu...................    3       1    S01
.......................................................................ugcacugccucuucccugUcu...................    5       1    S01
.......................................................................ugcacugccAcuucccuggcu...................    2       1    S01
.......................................................................ugcacugccucuucccuggcuG..................    2       1    S01
.......................................................................ugcacugccucuucccuggcAc..................    1       1    S01
.......................................................................ugcacugccucuucccuggcuc..................    54      0    S01
.......................................................................ugcacugccucuucccuggcuA..................    7       1    S01
.......................................................................ugcacugccucuucccuggcuU..................    46      1    S01
.......................................................................ugcacugccuAuucccuggcuc..................    1       1    S01
.......................................................................ugcacugccucuucccuggcuAu.................    1       1    S01
.......................................................................ugcacugccucuucccuggcucC.................    2       1    S01
.......................................................................ugcacugccucuucccuggcuGu.................    1       1    S01
.......................................................................ugcacugccucuucccuggcuUu.................    8       1    S01
.......................................................................ugcacugccucuucccuggcucugac..............    1       0    S01
........................................................................gcacugccucuucccuggc....................    1       0    S01
........................................................................gcacugccucuucccuggU....................    1       1    S01
.........................................................................cacugccucuucccuggc....................    4       0    S01
.........................................................................cacugccucuucccuggU....................    1       1    S01
.....................................................................................ccuggcucugacuccuuucu......    1       0    S01

...caaagacggggaacagacaga.......................................................................................    1       0    S02
.....aagacggggaacagacag........................................................................................    1       0    S02
.....aagacggggaacagacagagU.....................................................................................    1       1    S02
.....aagacggggaacagacagagc.....................................................................................    5       0    S02
.....aagacggggaacagacagagca....................................................................................    1       0    S02
.....aagacggggaAagacagagca....................................................................................    1       1    S02
.....aagacggggaacagacagagcauA..................................................................................    1       1    S02
.....aagacggggaacagacGgagcaug..................................................................................    1       1    S02
.....aagacggggaacagacagagcaug..................................................................................    48      0    S02
.....aagacggggaacagacagagcaCg..................................................................................    3       1    S02
```

Star **Mature**

```
agacaaagacggggaacagacagagcauggaugcagcuauuaacagucuuuuacuguuuugguuccacccaugcacugccucuucccuggcucugacuccuuucucuccuc
.....Uagacggggaacagacagagcaug..................................................................................  1    1  S02
.....aagacggggaacagacagagcaugC.................................................................................  1    1  S02
......agacggggaacagacagagcaugg.................................................................................  1    0  S02
.......gacggggaacagacagagcau...................................................................................  2    0  S02
.......gacggggaacagacagagcaug..................................................................................  10   0  S02
.......gacggggaacagacagagcaCg..................................................................................  1    1  S02
.......gacggggaacagacagagcauA..................................................................................  2    1  S02
.......Cacggggaacagacagagcaug..................................................................................  1    1  S02
.......gacggggaacagacagagcaugU.................................................................................  2    1  S02
........acggggaacagacagagc.....................................................................................  5    0  S02
........acggggaacagacagagU.....................................................................................  1    1  S02
........acggggaacagacagagA.....................................................................................  1    1  S02
........acggggaacUgacagagca....................................................................................  1    1  S02
........acggggaacagacagagca....................................................................................  7    0  S02
........acggggaacagacagagcau...................................................................................  8    0  S02
........acggggaacagacagagcaC...................................................................................  1    1  S02
........acggggaacagacCgagcaug..................................................................................  1    1  S02
........acggggaacagacagCgcaug..................................................................................  1    1  S02
........acggggaaGagacagagcaug..................................................................................  1    1  S02
........acggggaaAagacagagcaug..................................................................................  1    1  S02
........acggggaacagacagagcaug..................................................................................  341  0  S02
........acggggaacagacagagcCug..................................................................................  1    1  S02
........acgggAaacagacagagcaug..................................................................................  1    1  S02
........acggggGacagacagagcaug..................................................................................  2    1  S02
........acAgggaacagacagagcaug..................................................................................  1    1  S02
........acggggaacagacGgagcaug..................................................................................  2    1  S02
........acggggaCcagacagagcaug..................................................................................  1    1  S02
........acggggaacagacagagcaCg..................................................................................  24   1  S02
........Gcggggaacagacagagcaug..................................................................................  1    1  S02
........acggggaGcagacagagcaug..................................................................................  1    1  S02
........acggggaacCgacagagcaug..................................................................................  1    1  S02
........acggggaacagGcagagcaug..................................................................................  3    1  S02
........acgUggaacagacagagcaug..................................................................................  1    1  S02
........aAggggaacagacagagcaug..................................................................................  1    1  S02
........acggAgaacagacagagcaug..................................................................................  1    1  S02
........acggggaacagacagaAcaug..................................................................................  2    1  S02
........acgAggaacagacagagcaug..................................................................................  1    1  S02
........acggggaacagacagagcauA..................................................................................  9    1  S02
........acggggaacagacagagcauU..................................................................................  1    1  S02
........acggggaacagacagagcaGg..................................................................................  3    1  S02
........acgggCaacagacagagcaug..................................................................................  2    1  S02
........acggggaacGgacagagcaug..................................................................................  3    1  S02
........acggggaacagacagagcaugU.................................................................................  1    1  S02
........acggggaacagacagagcaugg.................................................................................  3    0  S02
........acggggaacagacagagcaugA.................................................................................  1    1  S02
........acggggaacagacagagcaugC.................................................................................  1    1  S02
........acggggaacagacagagcauggauA..............................................................................  1    1  S02
........acggggaacagacagagcauggauU..............................................................................  1    1  S02
.........cggggaacagacagagca....................................................................................  3    0  S02
.........cggggaacagacagagcU....................................................................................  2    1  S02
.........cggUgaacagacagagcau...................................................................................  1    1  S02
.........cggggGacagacagagcau...................................................................................  1    1  S02
.........cggggaacagacagagcaA...................................................................................  1    1  S02
.........cggggaacagacagagcaC...................................................................................  2    1  S02
.........cggggaacagaAagagcau...................................................................................  1    1  S02
.........cggggaacagacagagcau...................................................................................  47   0  S02
.........cggggaacagacagagcaGg..................................................................................  1    1  S02
.........cUgggaacagacagagcaug..................................................................................  1    1  S02
.........cggggaacagacagagcauA..................................................................................  5    1  S02
.........cggggaacagacagagcaug..................................................................................  29   0  S02
.........cggggaacagacagagcaCg..................................................................................  2    1  S02
.........cggUgaacagacagagcaug..................................................................................  1    1  S02
.........cggggaacagacCgagcaug..................................................................................  1    1  S02
.........cggggaAagacagagcaugg..................................................................................  3    1  S02
.........Gggggaacagacagagcaugg.................................................................................  2    1  S02
.........cggggaacagacagagcaugA.................................................................................  48   1  S02
.........cCgggaacagacagagcaugg.................................................................................  1    1  S02
.........cggggaacagacagGgcaugg.................................................................................  2    1  S02
.........cggggaacagacagagcGugg.................................................................................  3    1  S02
.........cggggaacagacaAagcaugg.................................................................................  1    1  S02
```

Star **Mature**

```
agacaaagacggggaacagacagagcauggaugcagcuauuaacagucuuuuacuguuuugguuccacccaugcacugccucuucccuggcucugacuccuuucucuccuc
.........cggggaacagaUagagcaugg.................................................................................  2    1  S02
.........cggggUacagacagagcaugg.................................................................................  1    1  S02
.........cggggaacagacagagcaGgg.................................................................................  1    1  S02
.........cggggaacagaGagagcaugg.................................................................................  1    1  S02
.........cgggAaacagacagagcaugg.................................................................................  1    1  S02
.........cggggaacagacagagcaugU.................................................................................  7    1  S02
.........cggggaacagacagagcaugC.................................................................................  3    1  S02
.........cggggaacagacGgagcaugg.................................................................................  1    1  S02
.........cggggaacagacagCgcaugg.................................................................................  2    1  S02
.........cggggaacagGcagagcaugg.................................................................................  2    1  S02
.........cggggaGcagacagagcaugg.................................................................................  2    1  S02
.........cggggGacagacagagcaugg.................................................................................  2    1  S02
.........cggggaacCgacagagcaugg.................................................................................  1    1  S02
.........cAgggaacagacagagcaugg.................................................................................  2    1  S02
.........cggAgaacagacagagcaugg.................................................................................  1    1  S02
.........cggggaacagacagagcaCgg.................................................................................  1    1  S02
.........cggggaacagacagagcauCg.................................................................................  5    1  S02
.........cggUgaacagacagagcaugg.................................................................................  8    1  S02
.........cggggaacagacagagcauAg.................................................................................  1    1  S02
.........cggggaacagacagagGaugg.................................................................................  1    1  S02
.........cggggaCcagacagagcaugg.................................................................................  1    1  S02
.........cggggaacagacagagcaugg.................................................................................  761  0  S02
.........cggggaaUagacagagcaugg.................................................................................  4    1  S02
.........cggggaacagacaCagcaugg.................................................................................  1    1  S02
.........Uggggaacagacagagcaugg.................................................................................  2    1  S02
.........cggggaacagacagaAcaugg.................................................................................  1    1  S02
.........cggggaacagacagagAaugg.................................................................................  1    1  S02
.........cggggaacGgacagagcaugg.................................................................................  4    1  S02
.........cggggaacagacagagcaAgg.................................................................................  2    1  S02
.........cggggCacagacagagcaugg.................................................................................  1    1  S02
.........cggggaacagacagagcauggC................................................................................  1    1  S02
.........cggggaacagacagagcaugga................................................................................  5    0  S02
.........cggggaacagacagagcauggU................................................................................  1    1  S02
.........cggggaacagGcagagcaugga................................................................................  1    1  S02
..........Cgggaacagacagagcaugg.................................................................................  1    1  S02
..........ggggaacagacagagcaugg.................................................................................  1    0  S02
...........gggaacagacagagcaug..................................................................................  1    0  S02
...........................uggaugcagcuauuaacU..................................................................  1    1  S02
.....................................................................caugcacugccucuucccuggc....................  1    0  S02
......................................................................augcacugccucuucccG.......................  1    1  S02
......................................................................augcacugccucuucccu.......................  9    0  S02
......................................................................augcacugccucuucccC.......................  1    1  S02
......................................................................augcacugccucuucccug......................  1    0  S02
......................................................................augcacugccucuucccuU......................  1    1  S02
......................................................................augcacugccucuucccugg.....................  7    0  S02
......................................................................augcacuUccucuucccuggc....................  4    1  S02
......................................................................augcacugccucuucUcuggc....................  5    1  S02
......................................................................augcacuCccucuucccuggc....................  3    1  S02
......................................................................augcacugccucuuccGuggc....................  1    1  S02
......................................................................augAacugccucuucccuggc....................  3    1  S02
......................................................................aAgcacugccucuucccuggc....................  2    1  S02
......................................................................augcacugccucuucccAggc....................  1    1  S02
......................................................................augcacugccuGuucccuggc....................  1    1  S02
......................................................................augcacugccAcuucccuggc....................  1    1  S02
......................................................................Uugcacugccucuucccuggc....................  1    1  S02
......................................................................augcacugccucCucccuggc....................  5    1  S02
......................................................................augcacugcGucuucccuggc....................  4    1  S02
......................................................................augcacugccucuucAcuggc....................  2    1  S02
......................................................................augcacugccucuucccGggc....................  2    1  S02
......................................................................augcacugccucuucccugAc....................  4    1  S02
......................................................................augcacugccucuuUccuggc....................  3    1  S02
......................................................................aCgcacugccucuucccuggc....................  9    1  S02
......................................................................aGgcacugccucuucccuggc....................  1    1  S02
......................................................................augcacugcAucuucccuggc....................  4    1  S02
......................................................................augcacugccucuCcccuggc....................  9    1  S02
......................................................................augcacugccucuucGcuggc....................  4    1  S02
......................................................................augcaGugccucuucccuggc....................  2    1  S02
......................................................................augcacugccucuucccuggU...................  271  1  S02
......................................................................augcacugccucuuccAuggc....................  1    1  S02
......................................................................augGacugccucuucccuggc....................  6    1  S02
```

```
         Star                                                          Mature
agacaaagacggggaacagacagagcauggaugcagcuauuaacagucuuuuacuguuuugguuccacccaugcacugccucuucccuggcucugacuccuuucucuccuc
......................................................................augcacuAccucuucccuggc....................   12     1   S02
......................................................................augUacugccucuucccuggc....................   3      1   S02
......................................................................Gugcacugccucuucccuggc....................   6      1   S02
......................................................................augcacugAcucuucccuggc....................   3      1   S02
......................................................................auAcacugccucuucccuggc....................   7      1   S02
......................................................................augcUcugccucuucccuggc....................   2      1   S02
......................................................................augcacugccucuucccuggc....................   1975   0   S02
......................................................................auCcacugccucuucccuggc....................   1      1   S02
......................................................................augcacugccucuucccuggG....................   12     1   S02
......................................................................augcGcugccucuucccuggc....................   10     1   S02
......................................................................augcacugccucuucccCggc....................   10     1   S02
......................................................................augcacugccucAucccuggc....................   3      1   S02
......................................................................augcacugccucuucccuUgc....................   4      1   S02
......................................................................augcacugccucGucccuggc....................   3      1   S02
......................................................................augcacugccucuuGccuggc....................   1      1   S02
......................................................................augcacugccucuucccuggA....................   60     1   S02
......................................................................augcacugccCcuucccuggc....................   10     1   S02
......................................................................augcacugccucuuAccuggc....................   2      1   S02
......................................................................Nugcacugccucuucccuggc....................   4      1   S02
......................................................................augcacugccuAuucccuggc....................   1      1   S02
......................................................................augcaUugccucuucccuggc....................   2      1   S02
......................................................................augcacugccucuAcccuggc....................   2      1   S02
......................................................................augcacugccucuucccugCc....................   1      1   S02
......................................................................Cugcacugccucuucccuggc....................   1      1   S02
......................................................................auUcacugccucuucccuggc....................   1      1   S02
......................................................................augcacugccucuucccuAgc....................   9      1   S02
......................................................................augcacugccucuuccUuggc....................   1      1   S02
......................................................................augcacugUcucuucccuggc....................   3      1   S02
......................................................................augcCcugccucuucccuggc....................   4      1   S02
......................................................................augcacCgccucuucccuggc....................   4      1   S02
......................................................................augcaAugccucuucccuggc....................   3      1   S02
......................................................................augcacugGcucuucccuggc....................   2      1   S02
......................................................................augcacugccucuucccuggcC...................   1      1   S02
......................................................................augcacugccucuucccuggcG...................   1      1   S02
.......................................................................Cgcacugccucuucccug......................   1      1   S02
.......................................................................ugcacugccucuucccuA......................   2      1   S02
.......................................................................ugcacugccucuucccug......................   30     0   S02
.......................................................................ugcacugccucuucccuU......................   4      1   S02
.......................................................................ugcacugccucuucccCg......................   2      1   S02
.......................................................................ugcacugccucuucccugC.....................   1      1   S02
.......................................................................ugcacugccucuucccugU.....................   5      1   S02
.......................................................................ugcacugccucuucccugg.....................   18     0   S02
.......................................................................uAcacugccucuucccugg.....................   1      1   S02
.......................................................................ugcacugccucuucccugA.....................   1      1   S02
.......................................................................ugcacugccucuucccuUg.....................   1      1   S02
.......................................................................Agcacugccucuucccuggc....................   1      1   S02
.......................................................................ugcacugccucuucUcuggc....................   3      1   S02
.......................................................................ugcacugccucuucccuggA...................   39     1   S02
.......................................................................ugcacugccucuAcccuggc....................   1      1   S02
.......................................................................ugcacugccucuuAccuggc....................   2      1   S02
.......................................................................ugcacugccucuucccuggc....................   1136   0   S02
.......................................................................uUcacugccucuucccuggc....................   1      1   S02
.......................................................................ugcacugccucuGcccuggc....................   1      1   S02
.......................................................................ugcacuAccucuucccuggc....................   3      1   S02
.......................................................................ugcacAgccucuucccuggc....................   2      1   S02
.......................................................................ugcacugccucuucccCggc....................   6      1   S02
.......................................................................ugcacugccucuucccuUgc....................   2      1   S02
.......................................................................ugcacugAcucuucccuggc....................   1      1   S02
.......................................................................ugcacCgccucuucccuggc....................   1      1   S02
.......................................................................ugcacugccucuucccuggU...................   180    1   S02
.......................................................................ugcacuCccucuucccuggc....................   2      1   S02
.......................................................................ugcacugccucuucccuggG...................   7      1   S02
.......................................................................ugcacugccucAucccuggc....................   1      1   S02
.......................................................................ugcacugccucuuccAuggc....................   3      1   S02
.......................................................................ugcacugccucuucccuAgc....................   3      1   S02
.......................................................................ugcacugccGcuucccuggc....................   1      1   S02
.......................................................................ugcacugccuAuucccuggc....................   2      1   S02
.......................................................................ugcacugccucCucccuggc....................   5      1   S02
.......................................................................ugcacGgccucuucccuggc....................   1      1   S02
.......................................................................ugcacugccucuucGcuggc....................   2      1   S02
```

Star Mature

```
agacaaagacggggaacagacagagcauggaugcagcuauuaacagucuuuuacuguuuugguuccacccaugcacugccucuucccuggcucugacuccuuucucuccuc
.......................................................................ugcacugccuUuucccuggc....................   5     1   S02
.......................................................................ugUacugccucuucccuggc....................   1     1   S02
.......................................................................ugcacugccuGuucccuggc....................   1     1   S02
.......................................................................ugcacugccucuucccugAc....................   4     1   S02
.......................................................................ugcacugUcucuucccuggc....................   4     1   S02
.......................................................................ugcacugcGucuucccuggc....................   1     1   S02
.......................................................................ugcacuUccucuucccuggc....................   5     1   S02
.......................................................................ugcacugccucuucAcuggc....................   1     1   S02
.......................................................................ugcacugccucuCcccuggc....................   6     1   S02
.......................................................................ugcacugccucuuUccuggc....................   4     1   S02
.......................................................................ugcacugccucuucccugUc....................   2     1   S02
.......................................................................uAcacugccucuucccuggc....................   2     1   S02
.......................................................................ugcacugccAcuucccuggc....................   1     1   S02
.......................................................................ugcacugcUucuucccuggc....................   2     1   S02
.......................................................................ugcacugccCcuucccuggc....................   3     1   S02
.......................................................................Cgcacugccucuucccuggc....................   4     1   S02
.......................................................................ugcacugGcucuucccuggc....................   1     1   S02
.......................................................................ugcUcugccucuucccuggc....................   1     1   S02
.......................................................................ugcGcugccucuucccuggc....................   10    1   S02
.......................................................................ugcacugccucuucccAggc....................   3     1   S02
.......................................................................ugcacugccucuuccUuggc....................   1     1   S02
.......................................................................Ggcacugccucuucccuggc....................   2     1   S02
.......................................................................ugcacugccucuuGccuggc....................   1     1   S02
.......................................................................Cgcacugccucuucccuggcu...................   3     1   S02
.......................................................................ugcacugccucuucccuggUu...................   1     1   S02
.......................................................................ugcacugccucuucccuggcG...................   61    1   S02
.......................................................................ugcacugccucuucccuggcA...................   28    1   S02
.......................................................................uUcacugccucuucccuggcu...................   1     1   S02
.......................................................................ugcacugccucuuAccuggcu...................   1     1   S02
.......................................................................ugcacAgccucuucccuggcu...................   1     1   S02
.......................................................................ugcacuUccucuucccuggcu...................   3     1   S02
.......................................................................ugcacugccucAucccuggcu...................   1     1   S02
.......................................................................Ggcacugccucuucccuggcu...................   1     1   S02
.......................................................................ugcacugccuGuucccuggcu...................   1     1   S02
.......................................................................ugcacugcUucuucccuggcu...................   1     1   S02
.......................................................................ugcUcugccucuucccuggcu...................   1     1   S02
.......................................................................ugcGcugccucuucccuggcu...................   3     1   S02
.......................................................................ugcacugccucuucccugAcu...................   1     1   S02
.......................................................................ugcacugccucuucccuUgcu...................   2     1   S02
.......................................................................Agcacugccucuucccuggcu...................   2     1   S02
.......................................................................ugcacugccucuucccuggcC...................   56    1   S02
.......................................................................ugcacugccucuucccugUcu...................   1     1   S02
.......................................................................ugcacugAcucuucccuggcu...................   1     1   S02
.......................................................................ugcacugccucuucccuggcu...................   444   0   S02
.......................................................................ugcacCgccucuucccuggcu...................   1     1   S02
.......................................................................ugcacugccucuucccCggcu...................   1     1   S02
.......................................................................ugcacugcAucuucccuggcu...................   2     1   S02
.......................................................................ugcacugccCcuucccuggcu...................   3     1   S02
.......................................................................ugcacugccGcuucccuggcu...................   1     1   S02
.......................................................................ugcacuAccucuucccuggcu...................   3     1   S02
.......................................................................ugcacugccucuuccAuggcu...................   1     1   S02
.......................................................................ugcacugccucuuGccuggcu...................   2     1   S02
.......................................................................ugcacugccucuuUccuggcu...................   1     1   S02
.......................................................................ugGacugccucuucccuggcu...................   1     1   S02
.......................................................................uCcacugccucuucccuggcu...................   1     1   S02
.......................................................................ugcacuCccucuucccuggcu...................   2     1   S02
.......................................................................ugcacugccucuucccuggcuG..................   1     1   S02
.......................................................................ugcacuCccucuucccuggcuc..................   1     1   S02
.......................................................................ugcacugccucuucccuggcuA..................   3     1   S02
.......................................................................ugcacugccucuucccuggcuc..................   15    0   S02
.......................................................................ugcacugccucuucccuggcuU..................   14    1   S02
.......................................................................ugcacugccucuucccuggcucG.................   1     1   S02
.......................................................................ugcacugccucuucccuggcucug................   1     0   S02
.....................................................................................ccuggcucugacuccuuucucu....   2     0   S02
```

Provisional ID	:	Maple_Unigene_BMK.15759_34885
Score total	:	0.3
Score for star read(s)	:	-1.3
Score for read counts	:	0
Score for mfe	:	2.2
Score for randfold	:	0
Score for cons. seed	:	-0.6
Total read count	:	593
Mature read count	:	593
Loop read count	:	0
Star read count	:	0

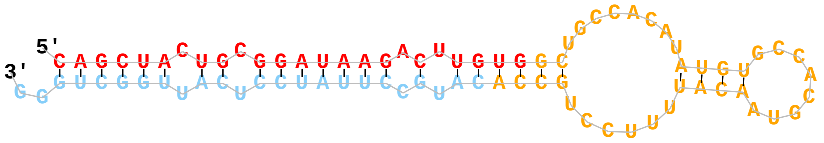

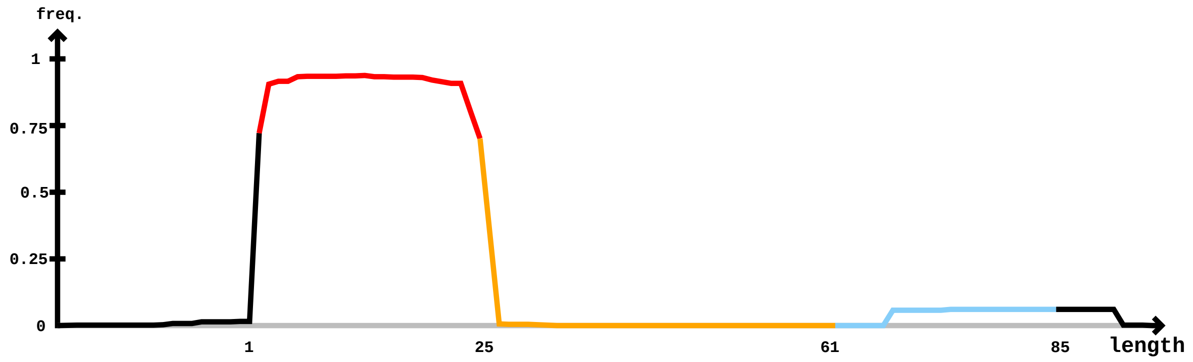

Mature **Star**

```
5'- cauugcuggguugugcucccagcuacugcggauaagacuuguggcugccacauaugugccacguaacauuuuccugccacaugccuuauccucauuggcugggugcaccacuc -3'   exp
    .......((((.((((.((((((((.((.(((((((.(.((((((........((((........))))......)))))).).))))))).)).)))))))).)))).))))   reads   mm   sample
    cauugcuggguugugcuccU.............................................................................................   1   1   S01
    ..........uugugcucccagcuacugcgg..................................................................................   1   0   S01
    ..........uugugcuccUagcuacugcgg..................................................................................   1   1   S01
    ............ugcuccUagcuacugcggauaag..............................................................................   1   1   S01
    ............ugcuccUagcuacugcggauaaga.............................................................................   2   1   S01
    ............ugcucccagcuacugcggauaagac............................................................................   1   0   S01
    ...................Uagcuacugcggauaaga............................................................................   3   1   S01
    ...................cagcuacugcggauaaga............................................................................   1   0   S01
    ...................cagcuacugcggauaagacu..........................................................................   1   0   S01
    ...................cagcuacugcggauaagacuug........................................................................   12   0   S01
    ...................cagcuacugcggaCaagacuug........................................................................   1   1   S01
    ...................cagcuacugcggauaagacuuU........................................................................   1   1   S01
    ...................cagcuacugcggauaagacuugC.......................................................................   5   1   S01
    ...................cagcuacugcggaGaagacuugu.......................................................................   1   1   S01
    ...................cagcuacugcggauaagacuugu.......................................................................   11   0   S01
    ...................cagcuacugcggauaagacCugug......................................................................   1   1   S01
    ...................cagcuacugcggauaagaGuugug......................................................................   1   1   S01
    ...................cagcuacugcggauaagacuuguA......................................................................   7   1   S01
    ...................cagcuacugcUgauaagacuugug......................................................................   1   1   S01
    ...................cagcuacugcggauaaUacuugug......................................................................   1   1   S01
    ...................cagcuacugcggauaagacAugug......................................................................   2   1   S01
    ...................cagcuacugcggauaagacuugug......................................................................   87   0   S01
    ...................cagcuacugcggauaagacuugCg......................................................................   1   1   S01
    ...................cagcuacugcggauaagacuuguC......................................................................   1   1   S01
    ...................cagcuacugAggauaagacuugug......................................................................   1   1   S01
    ...................cagcuGcugcggauaagacuugug......................................................................   2   1   S01
    ...................cagcuacugcggauaagacuCgug......................................................................   1   1   S01
    ...................cagcuacugcggaCaagacuugug......................................................................   1   1   S01
    ...................cagcuacAgcggauaagacuugug......................................................................   2   1   S01
    ...................cGgcuacugcggauaagacuugug......................................................................   1   1   S01
    ...................cagcuacugcggauaagacGugug......................................................................   1   1   S01
    ...................Uagcuacugcggauaagacuugug......................................................................   1   1   S01
    ...................cagcuacugcggauaagacuuUug......................................................................   1   1   S01
    ...................cagcuacugcggaGaagacuugug......................................................................   1   1   S01
```

```
                   Mature                                                     Star
cauugcuggguugugcucccagcuacugcggauaagacuuguggcugccacauaugugccacguaacauuuuccugccacaugccuuauccucauuggcugggugcaccacuc
....................agcuacugcggauaagacAug........................................................................   5    1   S01
....................agcuacugcggauaagacuAg........................................................................   1    1   S01
....................agcuacugcggauaagacuug........................................................................   2    0   S01
....................agcuaUugcggauaagacuugu.......................................................................   1    1   S01
....................agcuacugcggauaagacuugu.......................................................................   3    0   S01
....................agcuacugcggauaagacAugu.......................................................................   3    1   S01
....................agcuacugcggauaagacuugug......................................................................   1    0   S01
....................agcuacugcggauaagacAugug......................................................................   13   1   S01
....................agcuacugcggauaagacGugugg.....................................................................   1    1   S01
....................agcuacugcggauaagacuugugA.....................................................................   3    1   S01
....................agcuacugcggauaagacuugugg.....................................................................   15   0   S01
....................agcuacugcggauaagacAugugg.....................................................................   2    1   S01
.....................gcuacugcggauaagacuugu.......................................................................   1    0   S01
.....................gcuacugcggauaagacAugu.......................................................................   1    1   S01
.....................gcuacugcggauaagacAugug......................................................................   1    1   S01
.....................gcuacugcggauaagacuuguggc....................................................................   1    0   S01
.......................uacugcggauaagacAug........................................................................   1    1   S01
.......................uacugcggauaagacuuguU......................................................................   1    1   S01
.......................uacugcggauaagacAugug......................................................................   2    1   S01
..............................gauaagacuuguggcugcca...............................................................   1    0   S01
.....................................................................................uuauccucauuggcugggugcacA....   1    1   S01
.....................................................................................uuauccucauuggcugggugcacU....   3    1   S01
.....................................................................................uuauccAcauuggcugggugcacc....   1    1   S01
.....................................................................................uuauccucauuggcugggugcacc....   21   0   S01
...........................................................................................ucauuggcugggugcacc....   1    0   S01

.........guugugcucccagcuacugUggau................................................................................   1    1   S02
..........uugugcuccUagcuacugcgg..................................................................................   1    1   S02
.................cccagcuacugcggauaagacuug........................................................................   1    0   S02
...................Uagcuacugcggauaagac...........................................................................   3    1   S02
...................cagcuacugcggauaagacu..........................................................................   3    0   S02
...................cagcuacugcggauaagacuuU........................................................................   1    1   S02
...................cagcuacugcggauaagacuuA........................................................................   1    1   S02
...................cagcuacugcggauaagacuug........................................................................   7    0   S02
...................cagcuacugcggauaaAacuugu.......................................................................   1    1   S02
...................cagcuacugcggauaagacuugu.......................................................................   27   0   S02
...................cagcuacugcggauaagacuugA.......................................................................   2    1   S02
...................cagcuacugcggauaagacuuAu.......................................................................   1    1   S02
...................cagcuacugcggauaagacuCgu.......................................................................   1    1   S02
...................cagcuacugUggauaagacuugu.......................................................................   1    1   S02
...................cagUuacugcggauaagacuugug......................................................................   1    1   S02
...................cagcuacugcggauaagacuugug......................................................................   201  0   S02
...................cagcuacugcggauaagacuuguU......................................................................   6    1   S02
...................cagcuacugUggauaagacuugug......................................................................   1    1   S02
...................cGgcuacugcggauaagacuugug......................................................................   2    1   S02
...................cagcuacugcggauaagacuuguA......................................................................   17   1   S02
...................cagcuacugcggauaagaGuugug......................................................................   1    1   S02
...................cagcuacugcggauaagacuCgug......................................................................   1    1   S02
...................cagcuacugcggauaagacAugug......................................................................   4    1   S02
...................cagcuacugcggauaagacuuguC......................................................................   1    1   S02
...................cagcuacugcggauGagacuugug......................................................................   1    1   S02
...................cagcuacugcCgauaagacuugug......................................................................   1    1   S02
...................cagcuUcugcggauaagacuugug......................................................................   1    1   S02
...................cagcuacugAggauaagacuugug......................................................................   2    1   S02
...................cagcuacugcggauaagacuugCg......................................................................   10   1   S02
...................cagcuacugGggauaagacuugug......................................................................   1    1   S02
...................Uagcuacugcggauaagacuugug......................................................................   3    1   S02
...................cagcuacugcggauaagGcuugug......................................................................   1    1   S02
...................cagcuacugcggauaagaAuugug......................................................................   1    1   S02
....................agcuacugcggauaagacuug........................................................................   7    0   S02
....................agcuacugcggauaagacAug........................................................................   24   1   S02
....................agcuacAgcggauaagacuug........................................................................   1    1   S02
....................agcuacugcggauaagacuugu.......................................................................   4    0   S02
....................agcuacugcggauaagacAugu.......................................................................   2    1   S02
....................agcuacugcggauaagacuugug......................................................................   5    0   S02
....................agcuacugcggauaagacAugug......................................................................   9    1   S02
....................agcuacugcggauaagacAugugg.....................................................................   1    1   S02
....................agcuacugcggauaagacuugCgg.....................................................................   1    1   S02
....................agcuacugcggauaagacuugugg.....................................................................   15   0   S02
.....................gcuacugcggaCaagacuug........................................................................   1    1   S02
```

```
                   Mature                                                      Star
cauugcuggguugugcuccCAGCUACUGCGGAUAAGACUUGUGgcugccacauaugugccacguaacauuuuccugccaCAUGCCUUAUCCUCAUUGGCUGGGugcaccacuc
.....................gcuacugcggauaagacuug........................................................................    1    0    S02
.....................gcuacugcggauaagacuugu.......................................................................    1    0    S02
.......................uacugcggauaagacuuguU......................................................................    1    1    S02
.......................uacugcggauaagacAugug......................................................................    2    1    S02
.......................uacugcggauaagacuugug......................................................................    3    0    S02
.......................uacugcggauaagacuugugg.....................................................................    1    0    S02
........................acugcggauaagacuuguggcugc.................................................................    1    0    S02
............................cggauaagacAuguggcugcc................................................................    1    1    S02
.....................................................................................uuauccucauuggcugggugcacc....    8    0    S02
.....................................................................................uuauccucauuggcugggugcacA....    1    1    S02
.....................................................................................uuauccucauuggcugggugcacU....    2    1    S02
...........................................................................................ucauuggcugggugcaccacu.    1    0    S02
```

Provisional ID	:	Maple_Unigene_BMK.16052_35749
Score total	:	4446.2
Score for star read(s)	:	3.9
Score for read counts	:	4437.1
Score for mfe	:	2.1
Score for randfold	:	0
Score for cons. seed	:	3
Total read count	:	8715
Mature read count	:	8102
Loop read count	:	0
Star read count	:	613

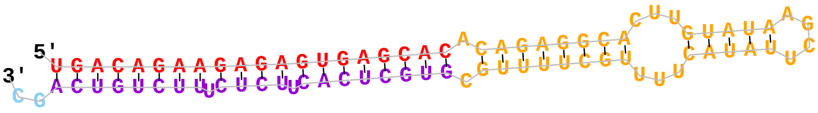

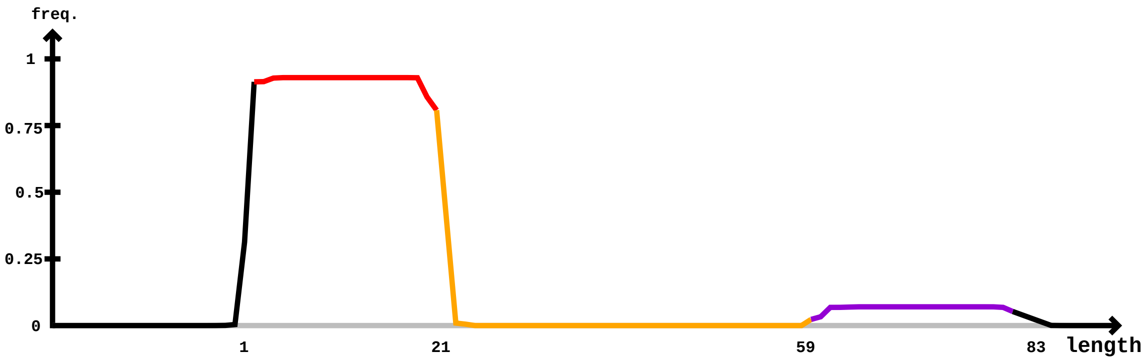

Mature **Star**

```
5'- auucuugcgacauagaaauugacagaagagagugagcacacagaggcacuuguauaagcuuauacuuuugcuuuugcgugcucacuucucuuucugucagcuuccagug  -3'   obs
    auucuugcgacauagaaauugacagaagagagugagcacacagaggcacuuguauaagcuuauacuuuugcuuuugcgugcucacuucucuuucugucagcuuccagug         exp
    ..........(((.(((.(((((((((((((((((((((.((((((((...(((((....)))))...)))))))).)))))))).)))).))))))))).)))..)))   reads   mm   sample
    ...............aaauugacagaagagagugagU........................................................................   1       1    S01
    ................aauugacagaagagagGgagc........................................................................   1       1    S01
    .................auugacagaagagGgugagc........................................................................   1       1    S01
    .................auugacagaagagagugagc........................................................................   12      0    S01
    .................auugacagaagagagugagU........................................................................   2       1    S01
    .................auugacagaagagagugagcU.......................................................................   1       1    S01
    .................auugacagaagagagugagca.......................................................................   1       0    S01
    .................Cuugacagaagagagugagcac......................................................................   3       1    S01
    .................auugacagaagagagugagcac......................................................................   1       0    S01
    ..................uugacagaagagagugaU.........................................................................   1       1    S01
    ..................uugacagaagagagugagU........................................................................   6       1    S01
    ..................uugacagaagagagGgagc........................................................................   1       1    S01
    ..................uugacagaagagCgugagc........................................................................   1       1    S01
    ..................uugacagaagagGgugagc........................................................................   1       1    S01
    ..................uugacagaagagagugagc........................................................................   57      0    S01
    ..................uugacagaagagagugagA........................................................................   1       1    S01
    ..................uugUcagaagagagugagc........................................................................   1       1    S01
    ..................uugGcagaagagagugagc........................................................................   1       1    S01
    ..................uugacCgaagagagugagc........................................................................   1       1    S01
    ..................uugacagaagGgagugagca.......................................................................   1       1    S01
    ..................uugacagaagagagugagcG.......................................................................   1       1    S01
    ..................uugacagGagagagugagca.......................................................................   1       1    S01
    ..................uugacagaagagagugagca.......................................................................   48      0    S01
    ..................uugacagaagagagugagcU.......................................................................   1       1    S01
    ..................uugacagaGgagagugagcac......................................................................   4       1    S01
    ..................uugacagaagagagugagcaA......................................................................   12      1    S01
    ..................uugacagaagagagugagcaG......................................................................   1       1    S01
    ..................uugacGgaagagagugagcac......................................................................   2       1    S01
    ..................Nugacagaagagagugagcac......................................................................   1       1    S01
    ..................uugacagGagagagugagcac......................................................................   5       1    S01
    ..................uugaGagaagagagugagcac......................................................................   1       1    S01
    ..................uugacaUaagagagugagcac......................................................................   1       1    S01
    ..................uugacagaagagaAugagcac......................................................................   2       1    S01
```

Mature Star

```
auucuugcgacauagaaauugacagaagagagugagcacacagaggcacuuguauaagcuuauacuuuugcuuuugcgugcucacuucucuucugucagcuuccagug
..................uugacagaagagaguAagcac..................................................................... 1     1    S01
..................uugacUgaagagagugagcac..................................................................... 1     1    S01
..................uugacagaagagagugUgcac..................................................................... 2     1    S01
..................uugCcagaagagagugagcac..................................................................... 2     1    S01
..................uugacagaagagagGgagcac..................................................................... 5     1    S01
..................uugacagaagagagugagcGc..................................................................... 2     1    S01
..................uugacagaagGgagugagcac..................................................................... 3     1    S01
..................uugacagaagagagugagcaU..................................................................... 83    1    S01
..................uugacagaagagagugGgcac..................................................................... 9     1    S01
..................uCgacagaagagagugagcac..................................................................... 2     1    S01
..................uugGcagaagagagugagcac..................................................................... 5     1    S01
..................uugacagaagagagugagAac..................................................................... 1     1    S01
..................Augacagaagagagugagcac..................................................................... 4     1    S01
..................uugacagCagagagugagcac..................................................................... 1     1    S01
..................uugUcagaagagagugagcac..................................................................... 2     1    S01
..................uugacagaagagagCgagcac..................................................................... 6     1    S01
..................uugacagaagagagugagUac..................................................................... 1     1    S01
..................uugacaAaagagagugagcac..................................................................... 1     1    S01
..................uugacagaagaUagugagcac..................................................................... 1     1    S01
..................uugacagaagagagugagcac..................................................................... 754   0    S01
..................uugacagaagagagugagcaca.................................................................... 1     0    S01
..................uugacagaagagagugagcacC.................................................................... 10    1    S01
..................uugacagaagagagugagcacG.................................................................... 2     1    S01
..................uugacagaagagagugagcacU.................................................................... 13    1    S01
..................uugacagaagagagugagcacacC.................................................................. 1     1    S01
...................ugacagaagagagugagU....................................................................... 21    1    S01
...................Ggacagaagagagugagc....................................................................... 1     1    S01
...................ugacagaaAagagugagc....................................................................... 2     1    S01
...................ugGcagaagagagugagc....................................................................... 1     1    S01
...................uCacagaagagagugagc....................................................................... 1     1    S01
...................ugacagaagagagugagA....................................................................... 3     1    S01
...................ugacagaagagGgugagc....................................................................... 2     1    S01
...................ugacagaGgagagugagc....................................................................... 1     1    S01
...................ugacagaagagagugGgc....................................................................... 1     1    S01
...................ugacGgaagagagugagc....................................................................... 2     1    S01
...................ugacagaagagagugagc....................................................................... 152   0    S01
...................Agacagaagagagugagc....................................................................... 1     1    S01
...................ugUcagaagagagugagc....................................................................... 1     1    S01
...................ugaGagaagagagugagc....................................................................... 1     1    S01
...................ugacUgaagagagugagc....................................................................... 1     1    S01
...................ugacagaagagagCgagc....................................................................... 3     1    S01
...................ugacagaagagagCgagca...................................................................... 1     1    S01
...................Ggacagaagagagugagca...................................................................... 2     1    S01
...................Cgacagaagagagugagca...................................................................... 2     1    S01
...................ugacagaagagagugagcG...................................................................... 6     1    S01
...................ugacagaagCgagugagca...................................................................... 1     1    S01
...................ugacagaagagGgugagca...................................................................... 1     1    S01
...................ugacagaagagagugagcU...................................................................... 5     1    S01
...................ugacagaagagagugagca...................................................................... 164   0    S01
...................ugacagaagGgagugagca...................................................................... 1     1    S01
...................ugacagaagagagugGgca...................................................................... 2     1    S01
...................ugaGagaagagagugagca...................................................................... 1     1    S01
...................ugaUagaagagagugagcac..................................................................... 2     1    S01
...................ugacagaagagagugagcaU..................................................................... 228   1    S01
...................ugacagaagagaguAagcac..................................................................... 1     1    S01
...................ugacagaagagagugagcaG..................................................................... 5     1    S01
...................ugacagaUgagagugagcac..................................................................... 1     1    S01
...................Cgacagaagagagugagcac..................................................................... 9     1    S01
...................ugacagaagagCgugagcac..................................................................... 2     1    S01
...................ugacagaagagagugagUac..................................................................... 4     1    S01
...................ugacagaagaUagugagcac..................................................................... 3     1    S01
...................ugacagaCgagagugagcac..................................................................... 1     1    S01
...................ugacagaagagagugagcac..................................................................... 1840  0    S01
...................Agacagaagagagugagcac..................................................................... 6     1    S01
...................ugacagaagagaUugagcac..................................................................... 3     1    S01
...................ugacagCagagagugagcac..................................................................... 2     1    S01
...................ugacagaagagaguUagcac..................................................................... 1     1    S01
...................ugacagaagagagugagGac..................................................................... 1     1    S01
...................ugacUgaagagagugagcac..................................................................... 2     1    S01
...................ugacagaagagUgugagcac..................................................................... 1     1    S01
```

```
                   Mature                                                        Star
auucuugcgacauagaaauugacagaagagagugagcacacagaggcacuuguauaagcuuauacuuuugcuuuugcgugcucacuucucuuucugucagcuuccagug
...................ugacagaagagagugUgcac......................................................................    3    1    S01
...................ugacagaagagagCgagcac......................................................................    14   1    S01
...................ugacagaagagagugGgcac......................................................................    6    1    S01
...................ugacagaagagagugagAac......................................................................    1    1    S01
...................ugacagaagagagGgagcac......................................................................    17   1    S01
...................uAacagaagagagugagcac......................................................................    1    1    S01
...................ugacagaagagGgugagcac......................................................................    12   1    S01
...................ugacagaagagagugagNac......................................................................    1    1    S01
...................ugacagaagagagugaAcac......................................................................    4    1    S01
...................ugacagaagGgagugagcac......................................................................    6    1    S01
...................ugacagaGgagagugagcac......................................................................    9    1    S01
...................ugacGgaagagagugagcac......................................................................    8    1    S01
...................Ngacagaagagagugagcac......................................................................    3    1    S01
...................ugacagaagUgagugagcac......................................................................    1    1    S01
...................ugacagaagagagugaUcac......................................................................    1    1    S01
...................ugacagaaAagagugagcac......................................................................    2    1    S01
...................ugacagaagagagugagcGc......................................................................    12   1    S01
...................ugaAagaagagagugagcac......................................................................    3    1    S01
...................ugacagaagaAagugagcac......................................................................    2    1    S01
...................ugacagaagaCagugagcac......................................................................    1    1    S01
...................ugacagaagagaCugagcac......................................................................    1    1    S01
...................Ggacagaagagagugagcac......................................................................    2    1    S01
...................ugaGagaagagagugagcac......................................................................    1    1    S01
...................ugacagaagagagugagcaA......................................................................    18   1    S01
...................ugacagUagagagugagcac......................................................................    3    1    S01
...................ugacaAaagagagugagcac......................................................................    1    1    S01
...................ugGcagaagagagugagcac......................................................................    10   1    S01
...................ugUcagaagagagugagcac......................................................................    1    1    S01
...................uCacagaagagagugagcac......................................................................    1    1    S01
...................ugacGgaagagagugagcaca.....................................................................    1    1    S01
...................ugacagaagagagugagcacC.....................................................................    42   1    S01
...................ugacagCagagagugagcaca.....................................................................    1    1    S01
...................ugacagaagagagugagcacG.....................................................................    2    1    S01
...................ugacagaagagagugagcacU.....................................................................    31   1    S01
...................ugacagaagagagugagcaca.....................................................................    16   0    S01
...................ugacagaagagagugagcacac....................................................................    1    0    S01
...................ugacagaagagagugagcacCc....................................................................    1    1    S01
....................gacagaagagagugagca.......................................................................    2    0    S01
....................gacagaagagagugagcac......................................................................    1    0    S01
....................gacagaagagagugagcaca.....................................................................    2    0    S01
.....................acagaagagagugagcac......................................................................    6    0    S01
.....................acagaagagagugagcaca.....................................................................    25   0    S01
.....................acagaagagagugagcacU.....................................................................    3    1    S01
.....................acagaagagagugagcacC.....................................................................    1    1    S01
.....................acagaagagagugagcacG.....................................................................    2    1    S01
.....................acagaagGgagugagcaca.....................................................................    1    1    S01
.....................acagaagagagugagcacac....................................................................    14   0    S01
.....................acagaagagagugagcacacC...................................................................    12   1    S01
.....................acagaagagagugagcacacU...................................................................    12   1    S01
......................cagaagagagugagcaca.....................................................................    1    0    S01
......................cagaagagagugagcacU.....................................................................    2    1    S01
......................cagaagaAagugagcaca.....................................................................    1    1    S01
......................cagaagagagugagcacac....................................................................    3    0    S01
......................cagaagagagugagcacCc....................................................................    1    1    S01
......................cagaagagagugagcacacU...................................................................    1    1    S01
.............................................................................gugcucacuucucuuucug.............    1    0    S01
.............................................................................gugcucacuucucuuucugG............    1    1    S01
.............................................................................gugcucaAuucucuuucuguc...........    1    1    S01
.............................................................................gugcucacuucucuuuUuguc...........    1    1    S01
.............................................................................gugcucacuucucuuucCguc...........    1    1    S01
.............................................................................gugcucacuucucuuucuguU...........    4    1    S01
.............................................................................gugcucacuucucuuucugCc...........    1    1    S01
.............................................................................gugcucacuucucuuucuguA...........    1    1    S01
.............................................................................gugcucacuCcucuuucuguc...........    1    1    S01
.............................................................................gugcucacuucucuuucuguc...........    32   0    S01
.............................................................................gugcucacuucucuuuUuguca..........    1    1    S01
.............................................................................gugcucacuucucuuucugucG..........    3    1    S01
.............................................................................gugcucacuucucuuucugucC..........    4    1    S01
.............................................................................gugcucacuucucuuucugucU..........    2    1    S01
.............................................................................gugcucacuucCcuuucuguca..........    1    1    S01
```

Mature **Star**

auucuugcgacauagaaauugacagaagagagugagcacacagaggcacuuguauaagcuuauacuuuugcuuuugcgugcucacuucucuuucugucagcuuccagug			
....gugcucacuucucuuucuUuca..........	3	1	S01
....gugcucacuucucuuucuguca..........	67	0	S01
....Nugcucacuucucuuucuguca..........	1	1	S01
....gugcucCcuucucuuucuguca..........	2	1	S01
....gCgcucacuucucuuucuguca..........	1	1	S01
....gugcucacuuUucuuucuguca..........	1	1	S01
....gugcucacuucucuuGcuguca..........	1	1	S01
....gugcucacuucucuuucuAuca..........	1	1	S01
....Augcucacuucucuuucuguca..........	1	1	S01
....gugcucacuucucuuucugucag.........	2	0	S01
....gugcucacuucucuuucugucaC.........	1	1	S01
....ugcucacCucucuuucugu............	1	1	S01
....ugcucacuucucuuuAuguc...........	1	1	S01
....ugcucacuucucuuucuguc...........	16	0	S01
....ugcucacuucucuuucuguU...........	1	1	S01
....ugcucacuucucuuucuguca..........	4	0	S01
....ugcucacuucucuuucugucag.........	25	0	S01
....ugcucacuucucuuucugucaA.........	3	1	S01
....ugcucacuAcucuuucugucag.........	1	1	S01
....ugcucacuucucuuucugucagU........	2	1	S01
....ugcucacuucucuuucugucagA........	1	1	S01
....ugcucacuucucuuucugucagc........	3	0	S01
....ugcucacuucucuuucugucagcA.......	1	1	S01
....gcucacuucucuuucugC............	2	1	S01
....gcucacuucucuuucugu............	6	0	S01
....gcucacuucGcuuucuguc...........	1	1	S01
....gcucacuucucuuucuguc...........	35	0	S01
....gcucacuucucuuucuguU...........	2	1	S01
....gcucCcuucucuuucuguc...........	1	1	S01
....gcucacuucucuuucuguA...........	1	1	S01
....gcucacuucucuuucuguca..........	5	0	S01
....gcucacuucucuuucugucG..........	1	1	S01
....gcucacuucucuuucugucU..........	1	1	S01
....Acucacuucucuuucugucag.........	1	1	S01
....gcucacuuUucuuucugucag.........	2	1	S01
....gcucacuucGcuuucugucag.........	1	1	S01
....gcucacuucucuuucugucaU.........	1	1	S01
....gcucacCucucuuucugucag.........	2	1	S01
....gcucacuuAucuuucugucag.........	1	1	S01
....Ucucacuucucuuucugucag.........	1	1	S01
....gcucacuucucuuucCgucag.........	3	1	S01
....gcucacuucucuuuUugucag.........	1	1	S01
....gcucacuucucuuucugucag.........	70	0	S01
....gcucacuucucuuucugucaA.........	4	1	S01
....gcucacuucucuuucugucGg.........	2	1	S01
....gcucacuucucuCucugucag.........	1	1	S01
....Acucacuucucuuucugucagc........	1	1	S01
....gcucacuucucuuCcugucagc........	1	1	S01
....gcucacuucucuuucugGcagc........	1	1	S01
....gcucacuucucuuucugucUgc........	1	1	S01
....gcucacuucucuuucugucagc........	40	0	S01
....gcucacuucucuuucugucagA........	2	1	S01
....gcucCcuucucuuucugucagc........	1	1	S01
....gcucacuucucuuuUugucagc........	1	1	S01
....gcCcacuucucuuucugucagc........	1	1	S01
....gcucacuucucuuucugucagU........	5	1	S01
....gcucacuGcucuuucugucagc........	1	1	S01
....gcucacuucucuuucuAucagc........	1	1	S01
....gcucacuucucuuucugucagcu.......	16	0	S01
....gcucacuucucuuucugucagcA.......	1	1	S01
....gcuUacuucucuuucugucagcu.......	1	1	S01
....gcucacuucucuuucugucagUu.......	1	1	S01
....gcucacuucucuuucugucagcuu......	1	0	S01
....cucacuucucuuucugucag.........	1	0	S01
....ucacuucucuuucugucGgc........	1	1	S01
....ucacuucucuuucugucagc........	1	0	S01
....ucacuucucuuucugucagcu.......	1	0	S01
....ucacuucucuuucugucagcA.......	1	1	S01
....ucacuucucuuucugucagcC.......	1	1	S01
....ucacuucucuuucugucagcuu......	2	0	S01

Mature **Star**

```
auucuugcgacauagaaauugacagaagagagugagcacacagaggcacuuguauaagcuuauacuuuugcuuuugcgugcucacuucucuucugucagcuuccagug
.................................................................................ucacuucucuucugucagcuuc.....   1    0    S01
..................................................................................cacuucucuucugucag.........   3    0    S01
..................................................................................cacuucucuucugucagc........   4    0    S01

................aauugacagaagagagAgagc.......................................................................   1    1    S02
................aauugacagaagagagugagc.......................................................................   2    0    S02
.................auugacagaagagagugagc.......................................................................   6    0    S02
.................auugacagaagagagAgagc.......................................................................   1    1    S02
.................auugacagaagagagugagcac.....................................................................   1    0    S02
..................uugacagaagagagugaA........................................................................   1    1    S02
..................uugacagaagagagugag........................................................................   2    0    S02
..................uugacagaagagagugaCc.......................................................................   1    1    S02
..................uugacagaagagagugagU.......................................................................   9    1    S02
..................uugacagaagagagCgagc.......................................................................   1    1    S02
..................uugacCgaagagagugagc.......................................................................   1    1    S02
..................uugacagaagagGgugagc.......................................................................   1    1    S02
..................uugacagaGgagagugagc.......................................................................   2    1    S02
..................uugacagaagagaguAagc.......................................................................   1    1    S02
..................uugacagaagagagugagA.......................................................................   2    1    S02
..................uugacagaagagagugagc.......................................................................   100  0    S02
..................uugacagaagagagugagG.......................................................................   1    1    S02
..................uugacagaagagagGgagca......................................................................   1    1    S02
..................uugacagaagagagugagAa......................................................................   1    1    S02
..................uugacGgaagagagugagca......................................................................   1    1    S02
..................uugacagaagagagugagcG......................................................................   5    1    S02
..................uugacagGagagagugagca......................................................................   1    1    S02
..................uugGcagaagagagugagca......................................................................   1    1    S02
..................uugacagaagagagugagcU......................................................................   1    1    S02
..................uugacagaaUagagugagca......................................................................   1    1    S02
..................uugacagaagagagugagca......................................................................   32   0    S02
..................uugacagaagagagugaAcac.....................................................................   2    1    S02
..................uAgacagaagagagugagcac.....................................................................   1    1    S02
..................uugacagaagagagugagUac.....................................................................   4    1    S02
..................Gugacagaagagagugagcac.....................................................................   1    1    S02
..................uugacagaagagaguUagcac.....................................................................   1    1    S02
..................uugacagaagGgagugagcac.....................................................................   9    1    S02
..................uugaGagaagagagugagcac.....................................................................   1    1    S02
..................uugacagaagagagugagcGc.....................................................................   10   1    S02
..................uugacagaagagagGgagcac.....................................................................   13   1    S02
..................uuUacagaagagagugagcac.....................................................................   2    1    S02
..................uugacagaGgagagugagcac.....................................................................   1    1    S02
..................uugacaAaagagagugagcac.....................................................................   1    1    S02
..................uugacagaagagagugagGac.....................................................................   3    1    S02
..................uugacagaagagagugagcUc.....................................................................   1    1    S02
..................uugacagaagagagugGgcac.....................................................................   9    1    S02
..................uugacCgaagagagugagcac.....................................................................   1    1    S02
..................uuCacagaagagagugagcac.....................................................................   1    1    S02
..................uCgacagaagagagugagcac.....................................................................   4    1    S02
..................uugacagaCgagagugagcac.....................................................................   1    1    S02
..................uugacaCaagagagugagcac.....................................................................   1    1    S02
..................uugacagaagCgagugagcac.....................................................................   2    1    S02
..................uugacagaagagagCgagcac.....................................................................   5    1    S02
..................Cugacagaagagagugagcac.....................................................................   3    1    S02
..................uugacagaagagagugagcaA.....................................................................   15   1    S02
..................uugacagaagagagugUgcac.....................................................................   2    1    S02
..................uugaUagaagagagugagcac.....................................................................   1    1    S02
..................uugacagaagagagugagcaG.....................................................................   4    1    S02
..................uugacagaagagaguAagcac.....................................................................   1    1    S02
..................uugacGgaagagagugagcac.....................................................................   5    1    S02
..................uugacagaagagagugagcaU.....................................................................   119  1    S02
..................uugacagaagagagugaCcac.....................................................................   1    1    S02
..................uugaAagaagagagugagcac.....................................................................   1    1    S02
..................uugGcagaagagagugagcac.....................................................................   10   1    S02
..................uugacagaagagCgugagcac.....................................................................   1    1    S02
..................uugacagaagagagugaUcac.....................................................................   2    1    S02
..................uugacagaagagagugagcCc.....................................................................   2    1    S02
..................uugacUgaagagagugagcac.....................................................................   2    1    S02
..................uugacagGagagagugagcac.....................................................................   4    1    S02
..................uugacagaagagGgugagcac.....................................................................   7    1    S02
..................uugacagaaAagagugagcac.....................................................................   3    1    S02
```

```
                    Mature                                                        Star
auucuugcgacauagaaauugacagaagagagugagcacacagaggcacuuguauaagcuuauacuuuugcuuuugcgugcucacuucucuucugucagcuuccagug
.................uugCcagaagagagugagcac......................................................................   1      1   S02
.................uugacagaagagagugagAac......................................................................   4      1   S02
.................uugacagaagagagugagcac......................................................................   1150   0   S02
.................uugacagUagagagugagcac......................................................................   2      1   S02
.................uugacagaagagagugagcacC.....................................................................   26     1   S02
.................uugacagaagagagugagcacU.....................................................................   16     1   S02
.................uugacagaagagagugagcaca.....................................................................   4      0   S02
.................uugacagaagagagAgagcaca.....................................................................   1      1   S02
.................uugacagaagagagugagcacG.....................................................................   2      1   S02
.................uugacagaagagagugagcacaU....................................................................   1      1   S02
.................uugacagaagagagugagcacUc....................................................................   1      1   S02
..................ugacagaagagagugagU........................................................................   29     1   S02
..................ugacagaaCagagugagc........................................................................   1      1   S02
..................ugacagaagagagugGgc........................................................................   1      1   S02
..................ugacagaagagagugagA........................................................................   1      1   S02
..................ugacagaagagaguAagc........................................................................   1      1   S02
..................ugacCgaagagagugagc........................................................................   1      1   S02
..................ugacagaagagagugagc........................................................................   179    0   S02
..................ugacagaCgagagugagc........................................................................   2      1   S02
..................ugacagaagGgagugagc........................................................................   1      1   S02
..................ugacagaagagGgugagc........................................................................   2      1   S02
..................Cgacagaagagagugagca.......................................................................   1      1   S02
..................ugacagaagagagugagcG.......................................................................   19     1   S02
..................ugacagaagagagugagca.......................................................................   120    0   S02
..................ugacagaGgagagugagca.......................................................................   2      1   S02
..................ugacagaagagagCgagca.......................................................................   1      1   S02
..................ugacagaagagagugagcU.......................................................................   4      1   S02
..................ugacagaagagagugUgcac......................................................................   3      1   S02
..................ugacagaagaAagugagcac......................................................................   3      1   S02
..................ugacagaagagagugagUac......................................................................   2      1   S02
..................ugacagaagagagugagAac......................................................................   1      1   S02
..................ugacagaagagagugaAcac......................................................................   3      1   S02
..................Ngacagaagagagugagcac......................................................................   2      1   S02
..................uAacagaagagagugagcac......................................................................   3      1   S02
..................ugacagaagagaAugagcac......................................................................   2      1   S02
..................Agacagaagagagugagcac......................................................................   1      1   S02
..................ugaUagaagagagugagcac......................................................................   1      1   S02
..................ugacagaGgagagugagcac......................................................................   3      1   S02
..................ugacagaagagaUugagcac......................................................................   1      1   S02
..................ugacagaagagagugagcac......................................................................   1689   0   S02
..................ugacaAaagagagugagcac......................................................................   2      1   S02
..................ugacagaagCgagugagcac......................................................................   1      1   S02
..................ugGcagaagagagugagcac......................................................................   10     1   S02
..................Cgacagaagagagugagcac......................................................................   2      1   S02
..................ugacGgaagagagugagcac......................................................................   10     1   S02
..................ugacagaagagaCugagcac......................................................................   1      1   S02
..................ugacagaagagagCgagcac......................................................................   13     1   S02
..................ugacagaagUgagugagcac......................................................................   4      1   S02
..................ugacagaUgagagugagcac......................................................................   1      1   S02
..................ugacagaagGgagugagcac......................................................................   14     1   S02
..................ugacagaagagagugagcGc......................................................................   5      1   S02
..................ugacagaagagagugagcUc......................................................................   1      1   S02
..................ugacagaagagaguUagcac......................................................................   2      1   S02
..................Ggacagaagagagugagcac......................................................................   3      1   S02
..................ugacagaagagagugagcaA......................................................................   15     1   S02
..................ugaGagaagagagugagcac......................................................................   2      1   S02
..................ugacagaaAagagugagcac......................................................................   2      1   S02
..................ugacagaagagagugGgcac......................................................................   12     1   S02
..................ugCcagaagagagugagcac......................................................................   1      1   S02
..................ugacagaagagagGgagcac......................................................................   9      1   S02
..................ugacagaagagGgugagcac......................................................................   9      1   S02
..................ugacagaCgagagugagcac......................................................................   1      1   S02
..................ugacagaagagagugagcaU......................................................................   183    1   S02
..................ugacagaagagagugagcaG......................................................................   9      1   S02
..................ugacagCagagagugagcac......................................................................   3      1   S02
..................ugacagaagagCgugagcac......................................................................   2      1   S02
..................ugaAagaagagagugagcac......................................................................   3      1   S02
..................ugacUgaagagagugagcac......................................................................   2      1   S02
..................ugacagaagagagugagcacU.....................................................................   24     1   S02
..................ugGcagaagagagugagcaca.....................................................................   1      1   S02
```

Mature / Star			
auucuugcgacauagaaauugacagaagagagugagcacacagaggcacuuguauaagcuuauacuuuugcuuuugcgugcucacuucucuuucugucagcuuccagug			
...................ugacagaagagagugagcacG...	8	1	S02
...................ugacagaagagagugagcacC...	44	1	S02
...................ugacagaagagagugagcaca...	28	0	S02
...................ugacagaagagagugagcGca...	1	1	S02
....................gacagaagagagugagcG...	1	1	S02
....................gacagaagagagugagcac..	1	0	S02
.....................acagaagagagugagcac..	2	0	S02
.....................acagaagagagugagcaca...	7	0	S02
.....................acagaagagagugagcacC...	2	1	S02
.....................acagaagagagugagcacac..	10	0	S02
.....................acagaagagagugagcacaU..	1	1	S02
.....................acagaagagagugagcacacU...	5	1	S02
.....................acagaagagagugagcacacG...	1	1	S02
.....................acagaagagagugagcacacC...	12	1	S02
......................cagaagagagugagcaca...	1	0	S02
......................cagaagagagugagcacU...	1	1	S02
......................cagaagagagugaAcacac..	1	1	S02
......................cagaagagagugagcacac..	1	0	S02
......................cagaagagagugagcacacU...	1	1	S02
......................cagaagagagugagcacacC...	1	1	S02
....gugcucacuucucuuucugu............	2	0	S02
....gugcucacuucucuuucuguU...........	1	1	S02
....gugcucacuucucuuucuguc...........	14	0	S02
....gugcCcacuucucuuucuguc...........	1	1	S02
....gugcucacuucucCuucuguca..........	1	1	S02
....gugcucCcuucucuuucuguca..........	1	1	S02
....gugcucacuucucuuucuguca..........	34	0	S02
....gugcucacuucucuuucugucG..........	4	1	S02
....guCcucacuucucuuucuguca..........	1	1	S02
....gugcucacuucucuuucugucC..........	2	1	S02
....gugcucacuucucuuucugucU..........	2	1	S02
....Uugcucacuucucuuucugucag.........	1	1	S02
....gugcucacuucucuuucugucag.........	1	0	S02
....ugcucacuucucuuucugu............	1	0	S02
....ugcucacuucucuuucuguc...........	6	0	S02
....ugcucacuucucuuucuguca..........	3	0	S02
....ugcucacuucucuuucugucG..........	1	1	S02
....ugcucacuucucuuucugucaA.........	1	1	S02
....ugcucacuucucuuucugucag.........	14	0	S02
....ugcucacuucucuuucugucagc........	2	0	S02
....gcucacuucucuuucugC............	1	1	S02
....gcucacuucucuuucugu............	3	0	S02
....gcCcacuucucuuucuguc...........	1	1	S02
....gcucacuucucuuucuguU...........	1	1	S02
....gcucacuucucuuucuguc...........	11	0	S02
....gcucacuucucuuucuguca..........	1	0	S02
....gcucacuucucuuucugucG..........	1	1	S02
....gcucacuucucuuucuguUag.........	1	1	S02
....gcucacuucucuuucugucaA.........	2	1	S02
....gcCcacuucucuuucugucag.........	1	1	S02
....gcucacuucucuuucugucag.........	25	0	S02
....gcucacuucucuuucugucagU........	3	1	S02
....gcucacuucucGuucugucagc........	1	1	S02
....gcucacuucucuuucugucagA........	1	1	S02
....gcucacuucucuuucugucagc........	18	0	S02
....gcucaAuucucuuucugucagc........	1	1	S02
....gcucacuucucuuucugucagcu.......	10	0	S02
....gcucCcuucucuuucugucagcu.......	1	1	S02
....gcucacuucucuuucugucagcC.......	2	1	S02
....gcucacuucucuuucugucagcA.......	1	1	S02
....ucacuucucuuucugucag.........	1	0	S02
....cacuucucAuucugucag.........	1	1	S02

Provisional ID	:	Maple_Unigene_BMK.17161_38786
Score total	:	1032.5
Score for star read(s)	:	3.9
Score for read counts	:	1023.9
Score for mfe	:	1.8
Score for randfold	:	0
Score for cons. seed	:	3
Total read count	:	2020
Mature read count	:	1620
Loop read count	:	4
Star read count	:	396

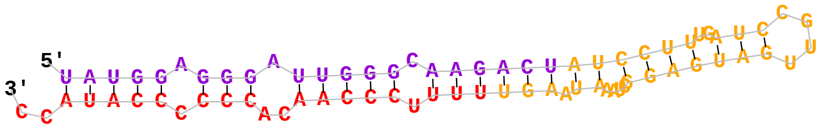

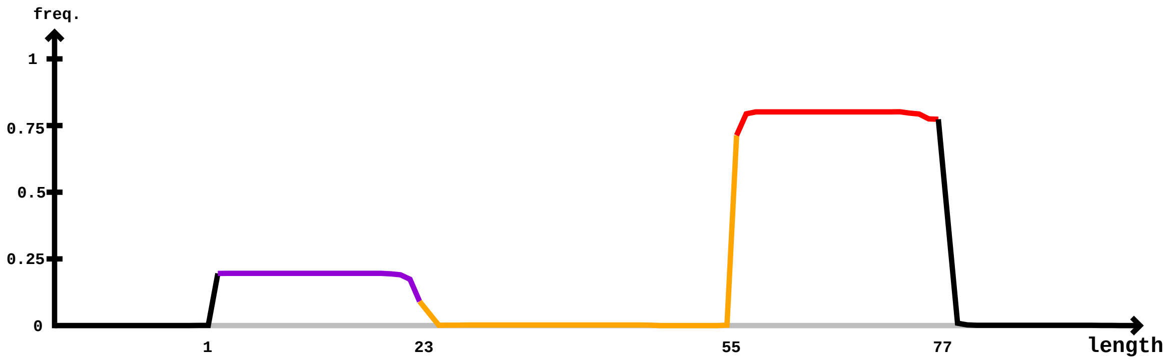

Star Mature

```
5'- ggugaaguuuuugguauggagggauugggcaagacuauccuuugauccguugaugaggcuaaauaaguuuuucccaacaccccccauaccguugauuucccucuacuccu   -3'   obs
    ggugaaguuuuugguauggagggauugggcaagacuauccuuugauccguugaugaggcuaaauaaguuuuucccaacaccccccauaccguugauuucccucuacuccu         exp
    ((.(((((((..((((((((.(((.(((((.(((((((((((((..(((....)))))))....)).)))))).)))))..))).))))))))..))))))).)).......   reads   mm   sample
    ..............uauggagggauugggcaa..............................................................................   1   0   S01
    ..............uauggagggauuUggcaa..............................................................................   1   1   S01
    ..............uauggagggauugggcaU..............................................................................   1   1   S01
    ..............uauggagggauugggcaaA.............................................................................   1   1   S01
    ..............uauggagggauugggcaag.............................................................................   3   0   S01
    ..............uauggagggauugggcaaga............................................................................   17   0   S01
    ..............uauggagggauugggcaagC............................................................................   1   1   S01
    ..............uauAgagggauugggcaaga............................................................................   1   1   S01
    ..............uauggagggauugggcaagU............................................................................   6   1   S01
    ..............uaCggagggauugggcaaga............................................................................   1   1   S01
    ..............uauggagggauugggcUaga............................................................................   1   1   S01
    ..............uauggagggauugggcaagac...........................................................................   86   0   S01
    ..............uauggagggauugggcaagaU...........................................................................   14   1   S01
    ..............uauggagggauuggAcaagac...........................................................................   1   1   S01
    ..............uauggagggauugggcaagaA...........................................................................   1   1   S01
    ..............uauggagggauugggcaGgac...........................................................................   2   1   S01
    ..............uauggagggauugggcGagac...........................................................................   1   1   S01
    ..............uauggagggaAugggcaagac...........................................................................   1   1   S01
    ..............uauggagggauugggcaaAac...........................................................................   1   1   S01
    ..............uauggagggauuUggcaagac...........................................................................   1   1   S01
    ..............uauUgagggauugggcaagac...........................................................................   1   1   S01
    ..............uauggagggauugggcaagGc...........................................................................   1   1   S01
    ..............uauggagggaCugggcaagacu..........................................................................   1   1   S01
    ..............uauggagggauugggcaagacA..........................................................................   2   1   S01
    ..............uauggagggauugggcaagaUu..........................................................................   14   1   S01
    ..............uauggagggGuugggcaagacu..........................................................................   1   1   S01
    ..............uauggagggauugggcaagacG..........................................................................   1   1   S01
    ..............uaCggagggauugggcaagacu..........................................................................   1   1   S01
    ..............uauggagggauugggcaagGcu..........................................................................   1   1   S01
    ..............uauggagggauugggcaagacC..........................................................................   15   1   S01
    ..............uaugAagggauugggcaagacu..........................................................................   1   1   S01
    ..............uauggCgggauugggcaagacu..........................................................................   1   1   S01
    ..............uauggagggauugggcGagacu..........................................................................   1   1   S01
```

Star **Mature**

gguggaaguuuuugguauggagggauugggcaagacuauccuuugauccguugaugaggcuaaauaaguuuuucccaacaccccccauaccguugauuucccucuacuccu			
...............uauggagggauugggcaaCacu..	1	1	S01
...............uauggagggauugggcaagacu..	69	0	S01
...............uauggagggauugggcaagacuG...	1	1	S01
...............uauggagggauugggcaagacuU...	1	1	S01
...............uauggagggauugggcaagacuCucc..	1	1	S01
................auggagggauugggcaagac...	1	0	S01
....................................uauccuuugauccguugCugaggcu..	1	1	S01
....ccuuugauccguugaugaggcu..	1	0	S01
....uuuuCcccaacaccccccauacc....................	1	1	S01
....uuuuucccaacaccccccauacc....................	1	0	S01
....uuuucccaacacccccca........................	10	0	S01
....uuuucccaacaAcccccau.......................	1	1	S01
....uuuucccaacaccccccau.......................	4	0	S01
....uuuucccaacaccccccaua......................	27	0	S01
....uuuucUcaacaccccccaua......................	1	1	S01
....uuuucccaaGaccccccauac.....................	1	1	S01
....uuuucccaacaccccccauac.....................	2	0	S01
....uuuucccGacaccccccauacc....................	5	1	S01
....uuuucccaacaccccAcauacc....................	2	1	S01
....uuGucccaacaccccccauacc....................	1	1	S01
....uuuucccaacaccccccaCacc....................	1	1	S01
....uuuuUccaacaccccccauacc....................	1	1	S01
....Nuuucccaacaccccccauacc....................	1	1	S01
....uuuucccaacaccccccCuacc....................	1	1	S01
....uuuucccaacacccAccauacc....................	2	1	S01
....uuuucccaacacGccccauacc....................	1	1	S01
....uuuucccaacaccccccGuacc....................	6	1	S01
....uuuucccaacacUccccauacc....................	1	1	S01
....uuuucccaacaccccUcauacc....................	1	1	S01
....uuuucccaacacccccAauacc....................	1	1	S01
....uuuucccaacGccccccauacc....................	2	1	S01
....uuuucccCacaccccccauacc....................	1	1	S01
....uuuucccaacaccccccauCcc....................	2	1	S01
....uuuucccaacaccccccaAacc....................	2	1	S01
....uuuucUcaacaccccccauacc....................	1	1	S01
....uuuucccaacaccccccauacU....................	175	1	S01
....Cuuucccaacaccccccauacc....................	3	1	S01
....uuuCcccaacaccccccauacc....................	3	1	S01
....uuuucccaacaccccccauUcc....................	1	1	S01
....uuuucccaaGaccccccauacc....................	1	1	S01
....uCuucccaacaccccccauacc....................	1	1	S01
....uuuucccaCcaccccccauacc....................	1	1	S01
....uuuucAcaacaccccccauacc....................	5	1	S01
....uuuGcccaacaccccccauacc....................	4	1	S01
....uuuucccaaAaccccccauacc....................	4	1	S01
....uuuuAccaacaccccccauacc....................	4	1	S01
....uuuucccaacacAccccauacc....................	1	1	S01
....uuuucGcaacaccccccauacc....................	3	1	S01
....uuuucccaacCccccccauacc....................	1	1	S01
....uuuucccaacaccccccauacG....................	4	1	S01
....uuuucccaacaccccccauacA....................	60	1	S01
....uuuucccaacacccccUauacc....................	3	1	S01
....uuuucccaacaccccccauacc....................	795	0	S01
....uuuucccaacacccccGauacc....................	1	1	S01
....uuuuGccaacaccccccauacc....................	1	1	S01
....uuuucccaacaccccccauaccU...................	2	1	S01
....uuuucccaacaccccccauaccgG..................	1	1	S01
....uuuucccaacaccccccauaccgu..................	1	0	S01
....uuuucccaacaccccccauaccguC.................	1	1	S01
....uuuucccaacaccccccauaccguu.................	1	0	S01
....uuucccaacaccccccau.......................	2	0	S01
....uuucccaacaccccccaua......................	1	0	S01
....uuAcccaacaccccccauacc....................	1	1	S01
....uuucccaacacUccccauacc....................	1	1	S01
....uuuUccaacaccccccauacc....................	3	1	S01
....uuuAccaacaccccccauacc....................	1	1	S01
....uuucccaacaccccccauGcc....................	1	1	S01
....uuucccaacaAcccccauacc....................	1	1	S01
....uuucccaacaccccccauacc....................	87	0	S01
....uuucccaaUaccccccauacc....................	1	1	S01

```
               Star                                                   Mature
gguggaaguuuuugguauggagggauugggcaagacuauccuuugauccguugaugaggcuaaauaaguuuuucccaacaccccccauaccguugauuucccucuacuccu
......................................................................uuucccGacaccccccauacc....................    3    1    S01
......................................................................uuucccaacGccccccauacc....................    1    1    S01
......................................................................uuucccaacaccccccauacA....................    3    1    S01
......................................................................Guucccaacaccccccauacc....................    2    1    S01
......................................................................uuucccaacaccccccauacU....................    22   1    S01
......................................................................uuucccaacacAccccauacc....................    1    1    S01
......................................................................uuucccaacaccccccaGacc....................    1    1    S01
......................................................................uuucAcaacaccccccauacc....................    1    1    S01
......................................................................uuucccaacaccccccauaccg...................    2    0    S01
.......................................................................uucccaacaccUcccaua......................    1    1    S01
.......................................................................uucccaacaccccccaua......................    1    0    S01
.......................................................................uuUccaacaccccccauaccgu..................    4    1    S01
.......................................................................uucccaacaccUcccauaccgu..................    3    1    S01
.......................................................................uuUccaacaccccccauaccguu.................    1    1    S01

.............gguauggagggauugggcaag.............................................................................    1    0    S02
...............uauggagggauugggcaU..............................................................................    1    1    S02
...............uauggagggauugggcaag.............................................................................    2    0    S02
...............uauggagggauugggcaaga............................................................................    7    0    S02
...............uauggagggauugggcaagac...........................................................................    46   0    S02
...............uauAgagggauugggcaagac...........................................................................    1    1    S02
...............uauUgagggauugggcaagac...........................................................................    1    1    S02
...............uauggagggauugggcaagaU...........................................................................    8    1    S02
...............uauggagggauGgggcaagac...........................................................................    1    1    S02
...............uauggagggauugggGaagac...........................................................................    1    1    S02
...............uauggagggauugggcaagacC..........................................................................    5    1    S02
...............uauggagggauugggcaagacu..........................................................................    48   0    S02
...............uauggagggauugggcaaAacu..........................................................................    1    1    S02
...............uauggagggauugggcaagacG..........................................................................    6    1    S02
...............uauggagggauugggcaagGcu..........................................................................    1    1    S02
...............uauggagggUuugggcaagacu..........................................................................    1    1    S02
...............uauggagggauugggcaagaUu..........................................................................    3    1    S02
...............uauggagggauugggcaagacA..........................................................................    3    1    S02
...............uauggagggauugggcaagacuC.........................................................................    1    1    S02
...............uauggagggauugggcaagacuU.........................................................................    1    1    S02
.........................................uuugauccguugaugaggA...................................................    1    1    S02
.........................................uuugauccguugaugaggcu..................................................    1    0    S02
....................................................................uuuuucccaacaccccccauacG....................    1    1    S02
.....................................................................uuuucccaacaccccccaua......................    5    0    S02
.....................................................................uuuucccaacaccccccauU......................    1    1    S02
.....................................................................uuuucccaaAaccccccauacc....................    1    1    S02
.....................................................................uuuucccaacaGcccccauacc....................    1    1    S02
.....................................................................uuGucccaacaccccccauacc....................    1    1    S02
.....................................................................Cuuucccaacaccccccauacc....................    2    1    S02
.....................................................................uuuucccaacGccccccauacc....................    1    1    S02
.....................................................................uuuucccaacaccccccauacG....................    12   1    S02
.....................................................................uuuucccaacaccccccauacA....................    12   1    S02
.....................................................................uuuucAcaacaccccccauacc....................    1    1    S02
.....................................................................uuuucccaacaccccccaCacc....................    1    1    S02
.....................................................................uuuucccaaGaccccccauacc....................    1    1    S02
.....................................................................uuuucccaacaccccccauaAc....................    1    1    S02
.....................................................................uAuucccaacaccccccauacc....................    1    1    S02
.....................................................................uuuucccGacaccccccauacc....................    2    1    S02
.....................................................................uuuucccaacaUcccccauacc....................    1    1    S02
.....................................................................uuuucccaacacccUccauacc....................    1    1    S02
.....................................................................uuuucccaacaccccccauacc....................    197  0    S02
.....................................................................uuuucccaacaccccccGuacc....................    2    1    S02
.....................................................................uuuucccaacaccccccauacU....................    35   1    S02
.....................................................................uuuucccaacaccccccauGcc....................    1    1    S02
.....................................................................uuuucccaGcaccccccauacc....................    1    1    S02
.....................................................................uuuuUccaacaccccccauacc....................    1    1    S02
.....................................................................uuuuccGaacaccccccauacc....................    1    1    S02
.....................................................................uuuucccaacaccccccauaccUu..................    1    1    S02
......................................................................uuucccaacaccccccaua......................    1    0    S02
......................................................................uuucccaacaccccccauacc....................    14   0    S02
......................................................................uuucccaacaccccccauacU....................    5    1    S02
......................................................................uuucccaacaccccccauacG....................    2    1    S02
......................................................................uuucccaacaccccccauacA....................    3    1    S02
......................................................................uuucccaacaccccAcauacc....................    1    1    S02
......................................................................uuucccaGcaccccccauacc....................    1    1    S02
```

```
               Star                                                   Mature
gguggaaguuuuugguauggagggauugggcaagacuauccuuugauccguugaugaggcuaaauaaguuuuucccaacaccccccauaccguugauuucccucuacuccu
......................................................................uuCcccaacaccccccauacc....................     1     1     S02
.......................................................................uucccaacaccccccauacc....................     1     0     S02
.......................................................................uucccaacaccUcccauacc....................     1     1     S02
.......................................................................uucccaacaccUcccauaccgu..................     2     1     S02
.......................................................................uucccaacaccccccauaccgu..................     1     0     S02
......................................................................................auaccguugauuucccucuac....     1     0     S02
.........................................................................................ccguugauuucccucuacuc..     1     0     S02
```

Provisional ID : Maple_Unigene_BMK.17554_40142
Score total : 3.4
Score for star read(s) : -1.3
Score for read counts : 0
Score for mfe : 1.7
Score for randfold : 0
Score for cons. seed : 3
Total read count : 505
Mature read count : 467
Loop read count : 0
Star read count : 38

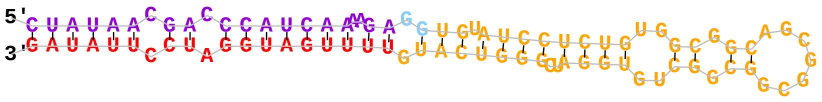

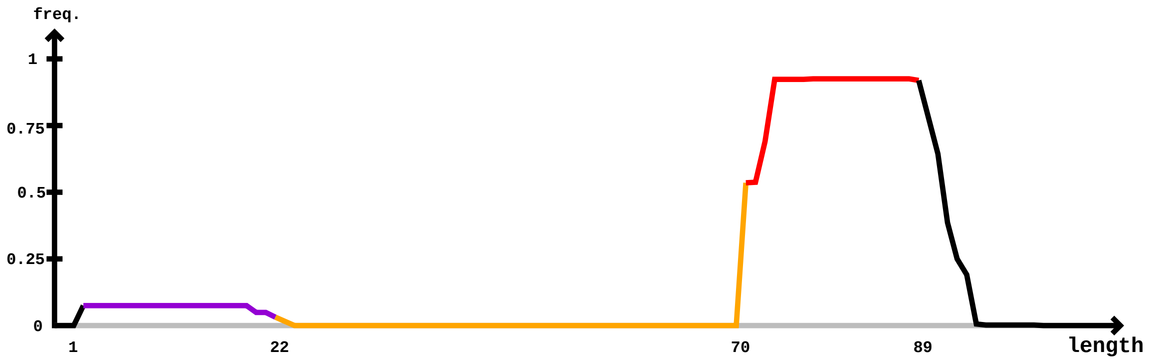

Star   Mature

```
5'- ccuauaacgacccaucaaaagagguguauccucuguggcggcagcggcggcggcuguggaucgggucauguuuugauggauccuuauaggguaguuggugguagccuug  -3'  obs
    ccuauaacgacccaucaaaagagguguauccucuguggcggcagcggcggcggcuguggaucgggucauguuuugauggauccuuauaggguaguuggugguagccuug        exp
    ((((((.((.(((((((..((.(((.((((((((..((.((.......)).))..))))..))))))).)))))))))).)).)))))))......(((....)))...  reads  mm  sample
    .cuauaacgacccaucaaa..........................................................................................  5  0  S01
    .cuauaacgacccaGcaaa..........................................................................................  1  1  S01
    .cuauaacgacccaucaaaGg........................................................................................  1  1  S01
    .cuauaacgacccaucaaaag........................................................................................  4  0  S01
    .cuauaacgacccaucaaaaA........................................................................................  1  1  S01
    .cuauaacgacccaucaaaaga.......................................................................................  9  0  S01
    .cuauaacgacccaucaaaagaU......................................................................................  1  1  S01
    ......................................................................uuuugauggauccuuaua.....................  2  0  S01
    ......................................................................uuuuCauggauccuuauag....................  1  1  S01
    ......................................................................uuCugauggauccuuauag....................  1  1  S01
    ......................................................................uuuugauggauccCuauag....................  1  1  S01
    ......................................................................uuuuUauggauccuuauag....................  1  1  S01
    ......................................................................uuuugauggauccuuauag....................  85  0  S01
    ......................................................................uuuugauggauccuAauag....................  1  1  S01
    ......................................................................uuuugauggCuccuuauag....................  1  1  S01
    ......................................................................uuuugauggauccuuauGg....................  1  1  S01
    ......................................................................uuuugauggauccuuauaA....................  4  1  S01
    ......................................................................uuuugauggauccuuauagg...................  1  0  S01
    ......................................................................Auuugauggauccuuauaggg..................  1  1  S01
    ......................................................................uuuugauggauccuuauaggC..................  1  1  S01
    ......................................................................uuuugauggauccuuaGaggg..................  1  1  S01
    ......................................................................uuuugauggGuccuuauaggg..................  1  1  S01
    ......................................................................uuuugauggauccuuauaCgg..................  1  1  S01
    ......................................................................uuuugauggauccuuauaggg..................  78  0  S01
    ......................................................................uuuugauggauccuuauaggU..................  1  1  S01
    ......................................................................uuuugauggauccuuauaggA..................  3  1  S01
    ......................................................................uuuugauAgauccuuauaggg..................  1  1  S01
    ......................................................................uuAugauggauccuuauaggg..................  1  1  S01
    ......................................................................uuuugauggauccuuauaggUu.................  1  1  S01
    .......................................................................uuugauggauccuuauagggu.................  1  0  S01
    ........................................................................uugauggauccuuauagg...................  1  0  S01
    ........................................................................uugauggauccuuauagggC.................  4  1  S01
    ........................................................................uugauggaCccuuauagggu.................  1  1  S01
```

```
 Star                                                                      Mature
ccuauaacgacccaucaaaagaggguguaucccucuguggcggcagcggcggcggcuguggaucgggucauguuuugauggauccuuauaggguaguuggugguagccuug
.........................................................................uugauggauccuuauagggA.................    1    1    S01
.........................................................................uugauggGuccuuauagggu.................    1    1    S01
.........................................................................uugauggauccuuauagggu.................    22   0    S01
.........................................................................uugauggauccuuauagggG.................    1    1    S01
.........................................................................uugauggauccuuauagggua................    18   0    S01
..........................................................................ugauggauccuuauagggu.................    11   0    S01
..........................................................................ugauggauccuuauaggAu.................    1    1    S01
..........................................................................ugauggauccuuauaggguG................    1    1    S01
..........................................................................ugauggauccuuauaAgguag...............    1    1    S01
..........................................................................ugauggGuccuuauaggguag...............    1    1    S01
..........................................................................ugauggauccuuauaggAuag...............    1    1    S01
..........................................................................ugauggauccuuauaggguaA...............    2    1    S01
..........................................................................ugauggauccuuauaggguag...............    43   0    S01
..........................................................................ugauggauccuCauaggguag...............    1    1    S01
..........................................................................ugauggauccuuauagCguag...............    1    1    S01
..........................................................................ugauggauccuGauaggguag...............    1    1    S01
..........................................................................ugauggauccuuauaggguagu..............    2    0    S01

.cuauaacgacccaucaaG...........................................................................................    2    1    S02
.cuauaacgacccaucaaa...........................................................................................    5    0    S02
.cuauaacgacccaucaaaag.........................................................................................    3    0    S02
.cuauaGcgacccaucaaaaga........................................................................................    1    1    S02
.cuauaacgacccaucaaaaga........................................................................................    5    0    S02
.......................................................................uuuugauggauccuuaua.....................    1    0    S02
.......................................................................uuuugauggauAcuuauag....................    1    1    S02
.......................................................................uuuugauggauccuuauag....................    35   0    S02
.......................................................................uuuugauggauccuuauaA....................    2    1    S02
.......................................................................uuuugauggauccuuCuag....................    1    1    S02
.......................................................................uuuugauggauccuuauagg...................    1    0    S02
.......................................................................uuuugauggauccuuauaggA..................    4    1    S02
.......................................................................uuuugauggauccuuauaggU..................    1    1    S02
.......................................................................uuuugauggauccuuauagCg..................    2    1    S02
.......................................................................uuuugauggauccuuauaggg..................    35   0    S02
.........................................................................uugauggauccuuauagg...................    2    0    S02
.........................................................................uugauggauGcuuauagggu.................    1    1    S02
.........................................................................uugauggauccuuauagggu.................    11   0    S02
.........................................................................uugauggauccuuauagggG.................    2    1    S02
.........................................................................uugauggauccuuauagggC.................    2    1    S02
.........................................................................uugauggauccuuauaggguU................    1    1    S02
.........................................................................uugauggauccuuauagggua................    10   0    S02
..........................................................................ugauggauccuuauagggu.................    4    0    S02
..........................................................................ugauggauccuuauagggG.................    2    1    S02
..........................................................................ugauggauccuuauagggC.................    2    1    S02
..........................................................................ugauggauccuuauagggA.................    1    1    S02
..........................................................................ugauggauccuuauaggguag...............    39   0    S02
..........................................................................uAauggauccuuauaggguag...............    1    1    S02
..........................................................................ugauggauccuuauaggguGg...............    1    1    S02
..........................................................................ugaGggauccuuauaggguag...............    1    1    S02
..........................................................................ugGuggauccuuauaggguag...............    1    1    S02
..............................................................................ggauccuuauaggguaguuggugg........    1    0    S02
```

Provisional ID	:	Maple_Unigene_BMK.17826_41302
Score total	:	0
Score for star read(s)	:	3.9
Score for read counts	:	-4.4
Score for mfe	:	-2.4
Score for randfold	:	0
Score for cons. seed	:	3
Total read count	:	3
Mature read count	:	1
Loop read count	:	1
Star read count	:	1

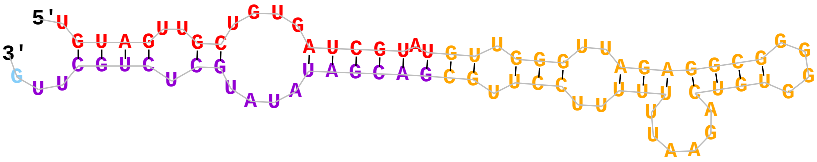

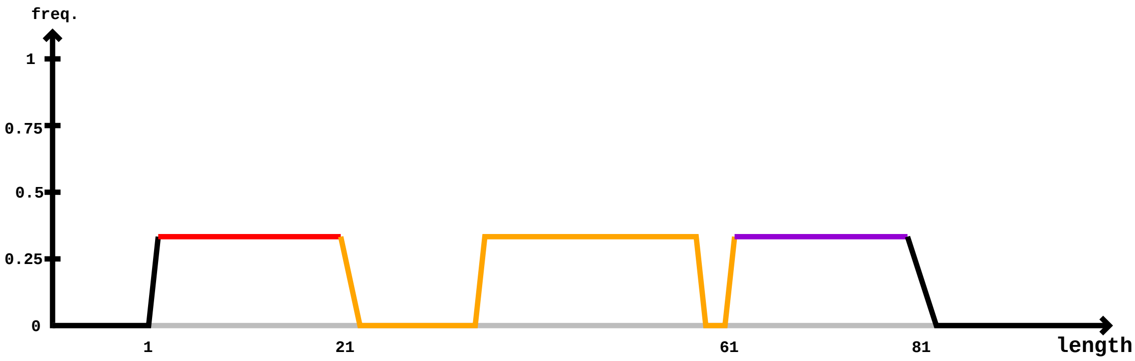

Mature **Star**

```
5'- uguauucaauguaguugcugugaucguauguuggguuagaggcgggggugucagaauuuuuuuuccuugcgacgauauaugcucugcuuggauguccacuuucauaugu   -3'    obs
    uguauucaauguaguugcugugaucguauguuggguuagaggcgggggugucagaauuuuuuuuccuugcgacgauauaugcucugcuuggauguccacuuucauaugu          exp
    ..(((((((.((((..((....(((((.(((.(((..(((((((....))))......)))..))).))))))))....)).)))))))))))................   reads   mm   sample
    .........uguaguugcugugaucguau................................................................................   1       0    S01
    ......................................................................gacgauauaugcucugcuC....................   1       1    S01

    ...........................................gggggugucagaauuuuuuuuccu..........................................   1       0    S02
```

Provisional ID	:	Maple_Unigene_BMK.18089_42434
Score total	:	1.3
Score for star read(s)	:	3.9
Score for read counts	:	-4.4
Score for mfe	:	-1
Score for randfold	:	0
Score for cons. seed	:	3
Total read count	:	3
Mature read count	:	2
Loop read count	:	0
Star read count	:	1

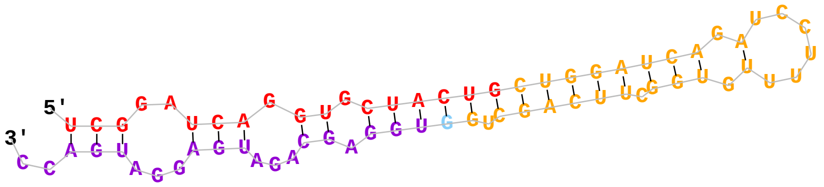

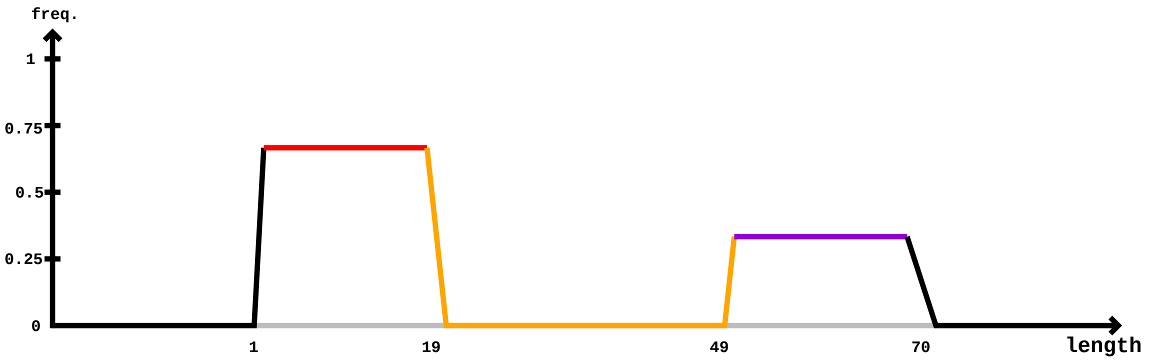


```
                      Mature                                   Star
5'- cgaauucagguuuucggaagucggaucaggugcuacugcuggaucagauccuuuuguggcuucagcugguggagcagaugaggaugaccuuuacaguuaaauuauaugu   -3'   obs
    cgaauucagguuuucggaagucggaucaggugcuacugcuggaucagauccuuuuguggcuucagcugguggagcagaugaggaugaccuuuacaguuaaauuauaugu         exp
    ((((........))))...((((..(((.((.((((((((((((((.(......).))).)))))).))))).))...)))...)))).....................   reads   mm   sample
    .....................................................................uggagcagaugaggaugacU....................   1       1    S01

    ....................ucggaucaggugcuacug.......................................................................   1       0    S02
    ....................ucggaucaggugcuacuU.......................................................................   1       1    S02
```

Provisional ID	:	Maple_Unigene_BMK.18641_45510
Score total	:	0.3
Score for star read(s)	:	-1.3
Score for read counts	:	-1.8
Score for mfe	:	0.5
Score for randfold	:	0
Score for cons. seed	:	3
Total read count	:	8
Mature read count	:	8
Loop read count	:	0
Star read count	:	0

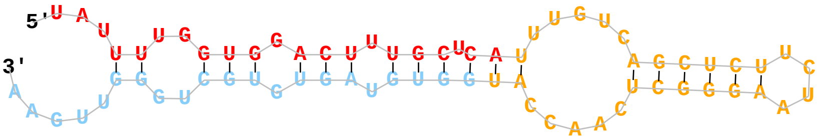

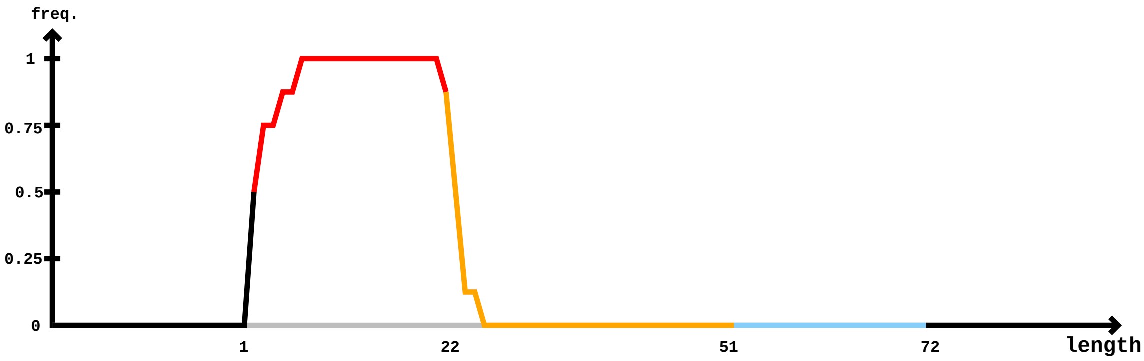

Mature **Star**

```
5'- uccccaguuguaugcuuuguauuuugguggacuuugcucauuugucagcucuucuaagggcucaaccauggguguagugugcuggguugaagaaguauggaauugaguac   -3'    exp
    ....(((((.((((((((....((..(((.(((.(((.(((.....((((((....)))))).....)))))).))).)))..)).....)))))))).))))).....     reads      mm          sample
    ...................Gauuuugguggacuuugcuca......................................................................     2          1           S01
    ....................auuuugguggacuuugcuc.......................................................................     1          0           S01

    ...................Gauuuugguggacuuugcuca......................................................................     2          1           S02
    ....................auuuugguggacuuugcuca......................................................................     1          0           S02
    .......................uugguggacuuugcuca......................................................................     1          0           S02
    ........................ugguggacuuugcucUuuu...................................................................     1          1           S02
```

Provisional ID	:	Maple_Unigene_BMK.20179_53584
Score total	:	2.8
Score for star read(s)	:	-1.3
Score for read counts	:	0
Score for mfe	:	1.1
Score for randfold	:	0
Score for cons. seed	:	3
Total read count	:	39
Mature read count	:	31
Loop read count	:	1
Star read count	:	7

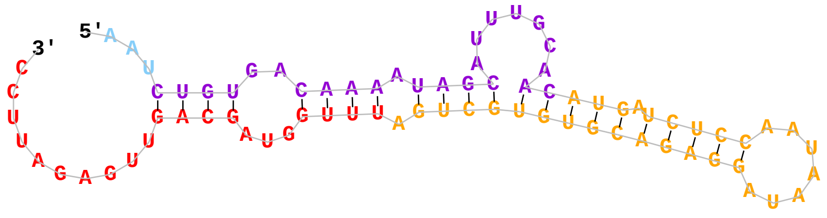

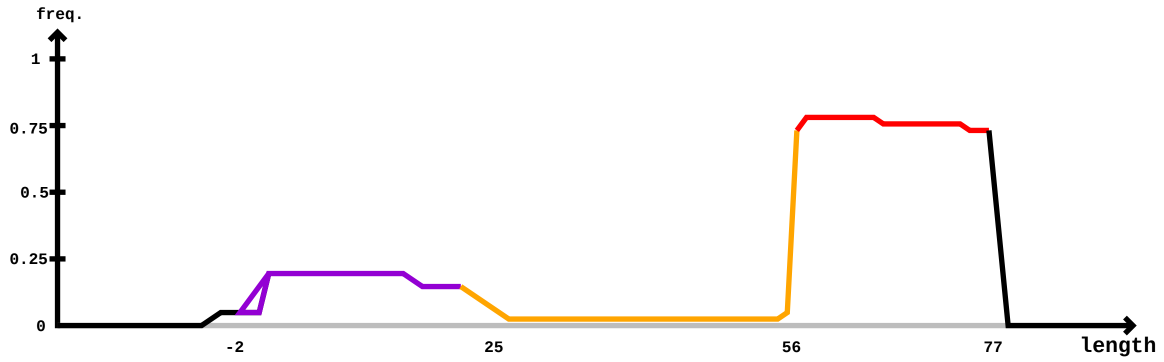


```
                        Star                                 Mature
5'- gauuccaacugagguugaaucugugacaaaauagcauuugcaacaugaucuccaauaauaggagacgugugcugauuugguagcaguugagauuccaacugagguugaau  -3'   obs
    gauuccaacugagguugaaucugugacaaaauagcauuugcaacaugaucuccaauaauaggagacgugugcugauuugguagcaguugagauuccaacugagguugaau        exp
    .....(((((..((((((((((.((((....((.((...(((.((((.(((((.......))))))))))))).....)).))...))))))))).))))).)))))...   reads   mm   sample
    ..............uugaaucugugacaaaauagc...........................................................................   1       0    S01
    ....................cugugacaaaauagcauuugcGac..................................................................   1       1    S01
    ....................cugugacaaaauagcauuugcaac..................................................................   4       0    S01
    ....................cugugacaaaauagcGuuugcaac..................................................................   1       1    S01
    ...........................................caugaucuccaauaauagg................................................   1       0    S01
    ...........................................................................uuugguagUaguugagauucc..............   2       1    S01
    ...........................................................................uuugguagcagCugagauucc..............   6       1    S01
    ............................................................................uugguagcagCugagauucca.............   1       1    S01

    ...............ugaaucugugacaaaauagca..........................................................................   1       0    S02
    ..............................................................agacgugugcugauuugguagc..........................   1       0    S02
    ..........................................................................auuugguagcagCugagau.................   1       1    S02
    ...........................................................................uuugguagUaguugagauucc..............   8       1    S02
    ...........................................................................uuugguagcagCugagauucc..............   12      1    S02
    ............................................................................uugguagcagCugagauucca.............   1       1    S02
```

Provisional ID	:	Maple_Unigene_BMK.20301_53976
Score total	:	3.5
Score for star read(s)	:	-1.3
Score for read counts	:	0
Score for mfe	:	1.8
Score for randfold	:	0
Score for cons. seed	:	3
Total read count	:	71
Mature read count	:	70
Loop read count	:	1
Star read count	:	0

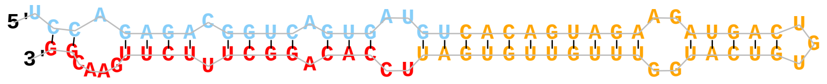

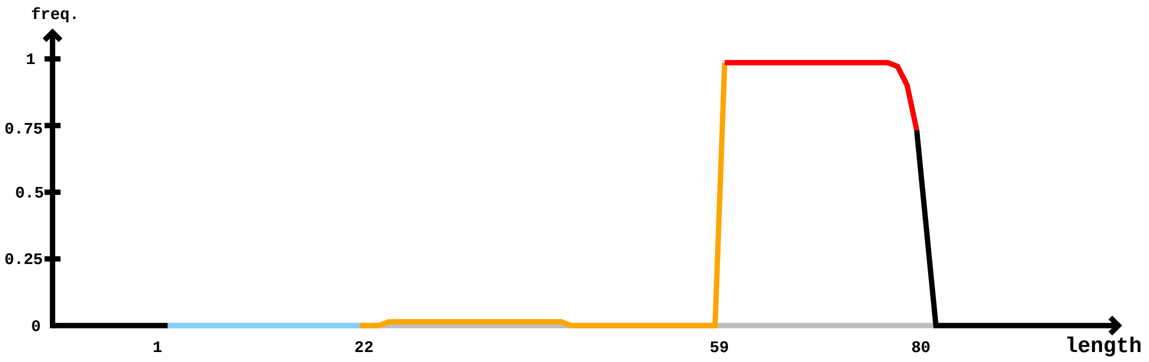


```
                Star                                                 Mature
5'- ugcugcugcuuccagagacggucagugaugucacaguagaagaugacugugucaugguuuguugugauuccacaggcuuucuugaacgguugcggccgcggugcaugug   -3'    exp
    .((.(((((..((.((((.((((.(((..((((((((((..(((((...)))))..))))))))))..))).)))).)))).))))....))..))))).))............    reads      mm         sample
    ....................................................................uccacaggcuuucuugaaU......................    1          1          S01
    ....................................................................uccacaggcuuucuugaacC.....................    1          1          S01
    ....................................................................uccacaggcuuucuugaacU.....................    2          1          S01
    ....................................................................uccacaggcuuucuugaacUg....................    10         1          S01

    .................................caguagaagaugacugugu.........................................................    1          0          S02
    ....................................................................uccacaggcuuucuugaa.......................    1          0          S02
    ....................................................................uccacaggcuuucuugaac......................    4          0          S02
    ....................................................................uccacaggcuuucuugaacA.....................    1          1          S02
    ....................................................................uccacaggcuuucuugaacC.....................    2          1          S02
    ....................................................................uccacaggcuuucuugaacg.....................    2          0          S02
    ....................................................................uccacaggcuuucuugaacU.....................    4          1          S02
    ....................................................................uccacaggcuuucuugaacUg....................    38         1          S02
    ....................................................................uccacaggcuuucuugaacCg....................    4          1          S02
```

Provisional ID	:	Maple_Unigene_BMK.21056_57975
Score total	:	1.8
Score for star read(s)	:	-1.3
Score for read counts	:	0
Score for mfe	:	0.1
Score for randfold	:	0
Score for cons. seed	:	3
Total read count	:	20
Mature read count	:	9
Loop read count	:	5
Star read count	:	6

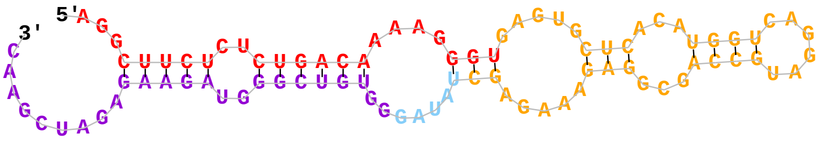

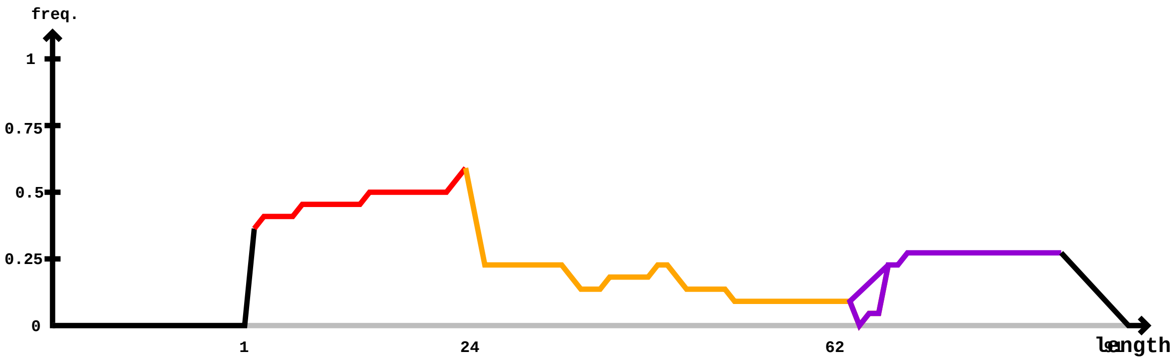


```
                      Mature                                                          Star
5'- agaaagacgaagaaggaagaggcuucucucugacaaaaggguagagugcucacauggucaggaugccagcggagaaagagcuauagggugucggguagaagagaucgaacgaa  -3'   obs
    agaaagacgaagaaggaagaggcuucucucugacaaaaggguagagugcucacauggucaggaugccagcggagaaagagcuauagggugucggguagaagagaucgaacgaa        exp
    .............(...((..(((((..((((((....(((.....(((...((((......))))...))).....))).....))))))..)))))...))...)...    reads   mm    sample
    ...................aggcuucucucugacaaaagggu......................................................................    2       0     S01
    ...................aggcuucucucugacaaaagAgu......................................................................    1       1     S01
    ...................aggcuucucGcugacaaaagggu......................................................................    1       1     S01
    ...................aggcuucCcucugacaaaagggu......................................................................    1       1     S01
    ...................aggcuucucucugacaaaaggguA.....................................................................    1       1     S01
    ....................ggcuucucucugacaaaagggu......................................................................    1       0     S01
    ........................ucucucugacaaaagggugagugcucacC...........................................................    1       1     S01
    ........................................gugagugcucacauggucaggaC.................................................    1       1     S01
    .........................................ugagugcucacauggucaggaugccaAc...........................................    1       1     S01
    ..........................................gagugcucacauggucaggauA................................................    1       1     S01
    .............................................................augccagcgAagaaagagcua..............................    1       1     S01
    ...................................................................................agggugucggguagaagagaucg......    1       0     S01
    .....................................................................................ggugucggguagaagagauc.......    1       0     S01
    .....................................................................................ggugucggguagaagagaucgaCc...    1       1     S01
    .......................................................................................ugucggguagaagagaucgaC....    1       1     S01

    ...................aggcuucucucugacaaaagggu......................................................................    2       0     S02
    ...............................gacaaaaggAugagugcucac............................................................    1       1     S02
    ........................................................ucaggaugccagcgAagaaagagcua..............................    1       1     S02
    .....................................................................................ggugucggguagaagagaucgaCc...    2       1     S02
```

Provisional ID	:	Maple_Unigene_BMK.21549_60752
Score total	:	0
Score for star read(s)	:	3.9
Score for read counts	:	-4.9
Score for mfe	:	1.7
Score for randfold	:	0
Score for cons. seed	:	-0.6
Total read count	:	2
Mature read count	:	1
Loop read count	:	0
Star read count	:	1

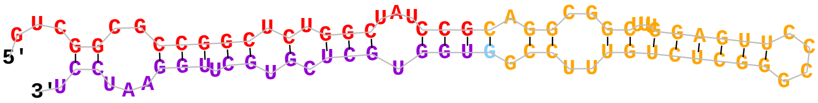

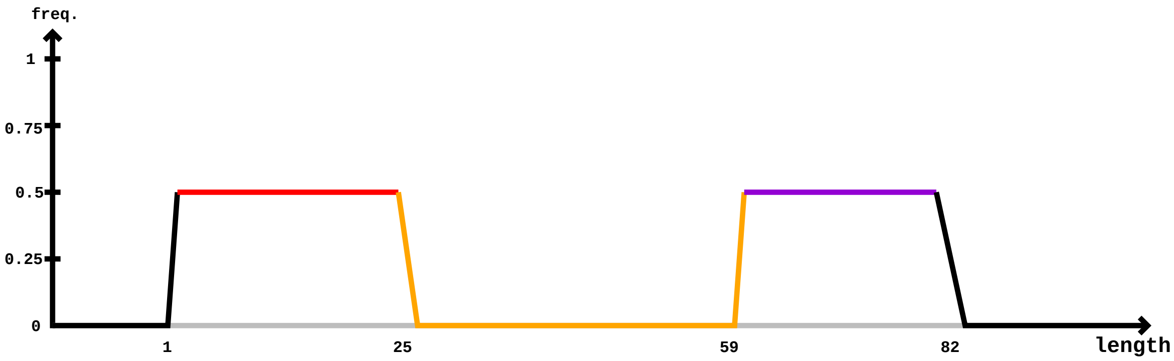

Mature Star

```
5'- ggcaagggaucgucggcgccggcucuggcuauccgcaggcggcuuuggaguucccgggcucuguuuccgguggugcucgugcuuggaauccuuuugcccuuucucuuccuua   -3'   obs
    ggcaagggaucgucggcgccggcucuggcuauccgcaggcggcuuuggaguucccgggcucuguuuccgguggugcucgugcuuggaauccuuuugcccuuucucuuccuua         exp
    ((((((((((..(.(((((.(((....((((.(((.((((((((..((....))..))))..))))).)))))))))).))))).)..)))).))))))..............   reads   mm   sample
    ...........gucggcgccggcucuggcuauccg.............................................................................   1       0    S01

    ......................................................................uggugcucgugcuuggaauccu....................   1       0    S02
```

Provisional ID : Maple_Unigene_BMK.22350_65602
Score total : 0.3
Score for star read(s) : -1.3
Score for read counts : 0
Score for mfe : -1.3
Score for randfold : 0
Score for cons. seed : 3
Total read count : 689
Mature read count : 688
Loop read count : 1
Star read count : 0

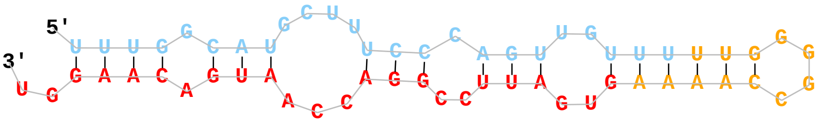

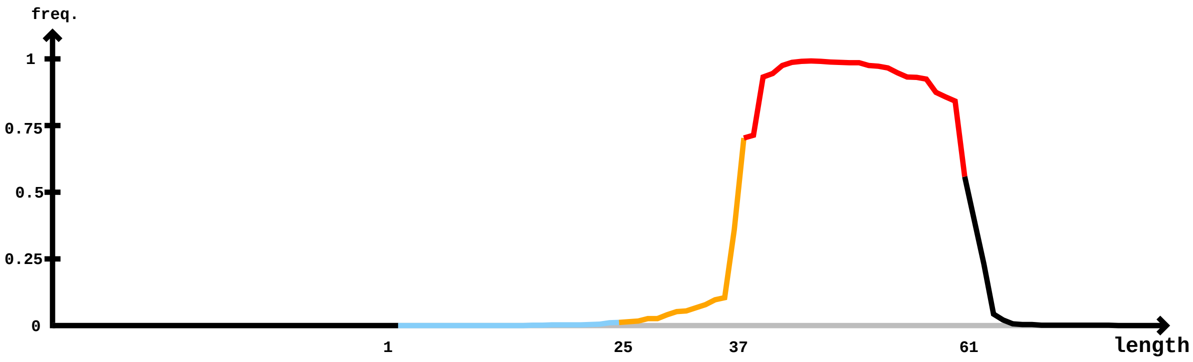

Star Mature

```
5'- uacagauugucuucccucuuaaaauuaaggcccaguuuggcaugcuuucccaguuguuuuuggggccaaaagugauuccggaccaaugacaagguguuugguaagauuguagaga   -3'   exp
    ...............((((...((((...(((((.((((.(((....(((.(((..((((((....))))))..)))..)))...))).)))).))...)))..))))..)))).   reads   mm   sample
    .................................................ccaguugCuuuuggggccaaaagu..........................................   1    1    S01
    .......................................................guuuuuggggccaaaagugauucc....................................   1    0    S01
    ..........................................................uuuggggcUaaaagugauu......................................   1    1    S01
    ............................................................uggggcUaaaagugauuccggacc...............................   1    1    S01
    .............................................................gggAccaaaagugauuccggacc...............................   1    1    S01
    .............................................................ggggccaaaagugauuccggacc...............................   1    0    S01
    .............................................................gggAccaaaagugauuccggacca..............................   1    1    S01
    ...............................................................gAccaaaagugauuccggaccaau............................   4    1    S01
    ................................................................Accaaaagugauuccggaccaau............................   1    1    S01
    ................................................................Accaaaagugauuccggaccaaug...........................   3    1    S01
    .................................................................ccaaaagugauuccggaccaauga..........................   1    0    S01
    ..................................................................caaaagugauuccggaccaC.............................   3    1    S01
    ..................................................................caaaagugauuccggaccaaA............................   1    1    S01
    ...................................................................aaaagugauuccgAaccaa.............................   1    1    S01
    ...................................................................aaaagugauuccggaUcaaugac.........................   1    1    S01
    ...................................................................aaaagugauuccggaccaaAgaca........................   1    1    S01
    ....................................................................aaagugauuccggaccaaugacC........................   1    1    S01
    ....................................................................aaagugauuccggaccaaugacG........................   1    1    S01
    ....................................................................aaagugauuccggaccaaugaca........................   3    0    S01
    ....................................................................aaagugauuccggaccaaugacaa.......................   1    0    S01
    .....................................................................aagugauuccggaccaaA............................   1    1    S01
    .....................................................................aagugauCccggaccaaugaca........................   1    1    S01
    ......................................................................agugauCccggaccaaugaca........................   1    1    S01
    ......................................................................agugauuccggaccaaugacaG.......................   1    1    S01
    ......................................................................agugauuccggaccaaugacaagA.....................   4    1    S01
    ......................................................................agugauCccggaccaaugacaagg.....................   7    1    S01
    ......................................................................agugauuccggaccaaAgacaagg.....................   1    1    S01
    ......................................................................agugauuccggaccaaugacaagU.....................   1    1    S01
    ......................................................................agugauuccggaccGaugacaagg.....................   1    1    S01
    ......................................................................agugauuccggaccaaugacaagg.....................   59   0    S01
    ......................................................................agugauuccggaccaGugacaagg.....................   1    1    S01
    ......................................................................aguUauuccggaccaaugacaagg.....................   1    1    S01
    ......................................................................agugGuuccggaccaaugacaagg.....................   1    1    S01
    ......................................................................agugauuccggaccaaugacaaCg.....................   1    1    S01
```

Star Mature

uacagauugucuuccucuuaaaauuaaggcccaguuuggcaugcuuucccaguuguuuuuggggccaaaagugauuccggaccaaugacaagguguuugguaagauuguagaga			
....agugauuccggaccaaugacaGgg.....................	9	1	S01
....Ggugauuccggaccaaugacaagg.....................	1	1	S01
....agugauuccggaccaaugacaaggu....................	1	0	S01
....gugauuccggaccaaugaca........................	8	0	S01
....gugauuccggaccaaugacaa.......................	3	0	S01
....gugauuccggaccaaugacaaA......................	1	1	S01
....gugauuccggaccaaugacaGgg.....................	2	1	S01
....gugauuccggaccaaugacaagg.....................	7	0	S01
....gugauCccggaccaaugacaaggu....................	1	1	S01
....gugauuccggaccaaugacaGggu....................	3	1	S01
....gugauuccggaccaaugacaaggu....................	79	0	S01
....gugauuccggaccaaugacaaggA....................	2	1	S01
....gugauuccUgaccaaugacaaggu....................	1	1	S01
....gGgauuccggaccaaugacaaggu....................	1	1	S01
....gugauuccggaccaaugacaaggG....................	3	1	S01
....gugauuccggaccaaAgacaaggu....................	1	1	S01
....gCgauuccggaccaaugacaaggu....................	1	1	S01
....gugauuccggaccaaugacaaggC....................	11	1	S01
....gugaCuccggaccaaugacaaggu....................	1	1	S01
....gugauuccggaccaauAacaaggu....................	1	1	S01
....ugauuccAgaccaaugacaaggu....................	1	1	S01
....ugauuccggaccaaugacaaggug...................	1	0	S01
....gauuccggaccaaugacaG.......................	1	1	S01
....gauuccggaccaaugacaGg......................	1	1	S01
....gauuccggaccaaugacaagg.....................	3	0	S01
....gauCccggaccaaugacaaggu....................	1	1	S01
....gauuccggaccaaugacaGggu....................	1	1	S01
....gauuccggaccaaugacaaggu....................	1	0	S01
....gauuccggaccaaugacaaggug...................	4	0	S01
....gauCccggaccaaugacaaggug...................	1	1	S01
....gauCccggaccaaugacaaggugu..................	4	1	S01
....gauuccggaccaaugacaaggugC..................	2	1	S01
....gauuccggaccaaugaAaaggugu..................	1	1	S01
....gauuccggaccaGugacaaggugu..................	1	1	S01
....gauuccggaccaaugacaGggugu..................	2	1	S01
....gauuccggaccaaugacaaggugu..................	26	0	S01
....gauuccggaccaaAgacaaggugu..................	1	1	S01
....gauCccggaccaaugacaagguguu.................	1	1	S01
....gauuccggaccaaugacaGgguguu.................	1	1	S01
....auuccggaccaaugacaaggu....................	1	0	S01
....auuccggaccaaugacaaggugu..................	1	0	S01
....auuccggaccaaugacaGgguguu.................	1	1	S01
....auCccggaccaaugacaagguguu.................	1	1	S01
....uuccggaccaaugacaaggug...................	1	0	S01
....uCccggaccaaugacaaggug...................	1	1	S01
....uuccggaccaaugacaagguguuu................	3	0	S01
....uuccggaccaaugacaGgguguuu................	2	1	S01
....Cccggaccaaugacaagg.....................	2	1	S01
....uccggaccaaugacaaggu....................	1	0	S01
....Cccggaccaaugacaagguguuug...............	1	1	S01
....uccggaccaaugacaagguguuug...............	1	0	S01
....ccggaccaaugacaaggu....................	1	0	S01
....ccggaccaaugacaGggug...................	1	1	S01
....ccggaUcaaugacaaggug...................	1	1	S01
....ugacaagguguuugguaagauugC.....	1	1	S01

....aguuguuuuuggggccaaaaguga..	1	0	S02
....Cuuuuggggccaaaagugauuccg...................................	1	1	S02
....uuuuggggcAaaaagugau.......................................	1	1	S02
....uuuugggAccaaaagugauu......................................	1	1	S02
....uuuuggggccaaaagugauuccA...................................	1	1	S02
....uuuuAgggccaaaagugauuccgg..................................	1	1	S02
....uggggccaaaagugauuccggacc...............................	1	0	S02
....Gggggccaaaagugauuccggacc...............................	2	1	S02
....ggggccaaaagugauuccgga.................................	1	0	S02
....ggggccaaaagugauuccgAacc...............................	1	1	S02
....gggAccaaaagugauuccggacca..............................	1	1	S02
....gggAccaaaagugauuccggaccaaug...........................	1	1	S02
....gAccaaaagugauuccggacc...............................	1	1	S02
....gAccaaaagugauuccggaccaau............................	6	1	S02

Star **Mature**

```
uacagauugucuuccucuuaaaauuaaggcccaguuuggcaugcuuucccaguuguuuuuggggccaaaagugauuccggaccaaugacaagguguuugguaagauuguagaga
...............................................................Accaaaagugauuccggaccaaug...........................  5   1  S02
................................................................ccaaaagugauuccggaccaaAg...........................  1   1  S02
.................................................................caaaagugauUcggaccaa..............................  1   1  S02
.................................................................caaaagugauuccggaccaaAg...........................  2   1  S02
.................................................................caaaagugauuccggaccaaugac.........................  1   0  S02
.................................................................caaaagugauCccggaccaaugaca........................  1   1  S02
..................................................................aaaagugauuccggaccaaAgac.........................  1   1  S02
..................................................................aaaagugauuccggaccaaugaca........................  1   0  S02
..................................................................aaaagugauCccggaccaaugaca........................  1   1  S02
..................................................................aaaagugauuccggaccaaAgaca........................  3   1  S02
...................................................................aaagugauuccggaccaC.............................  1   1  S02
...................................................................aaagugauuccggaccaaA............................  1   1  S02
...................................................................aaagugauuccggaccaaAgac.........................  1   1  S02
...................................................................aaagugauuccggaccaaugac.........................  1   0  S02
...................................................................aaagugauuccggaccaaugaca........................  3   0  S02
...................................................................aaagugauuccggaccaaugacaCg......................  1   1  S02
....................................................................aagugauCccggaccaaugaca........................  1   1  S02
....................................................................aagugauuccggaccaaugacaGg......................  1   1  S02
....................................................................aagugauuccggaccaaugacaag......................  2   0  S02
.....................................................................agugauuccggaccaaugaca........................  1   0  S02
.....................................................................agugauuccggaccaaugacaG.......................  3   1  S02
.....................................................................agugauuccggaccaaugacaCg......................  1   1  S02
.....................................................................agugauuccggaccaaugacaGg......................  1   1  S02
.....................................................................Ggugauuccggaccaaugacaagg.....................  2   1  S02
.....................................................................agugauCccggaccaaugacaagg.....................  12  1  S02
.....................................................................aguAauuccggaccaaugacaagg.....................  1   1  S02
.....................................................................agugauuccggaccaaugacaagg.....................  60  0  S02
.....................................................................agugauuccggaccaaugacaagA.....................  5   1  S02
.....................................................................agugauuccggaccaGugacaagg.....................  1   1  S02
.....................................................................agugauuccggaccaaugacaGgg.....................  12  1  S02
.....................................................................agugUuuccggaccaaugacaagg.....................  1   1  S02
.....................................................................agugauuccggaccaaugacaaCg.....................  2   1  S02
.....................................................................agugauuccggaccaaAgacaagg.....................  3   1  S02
......................................................................gugauuccggaccaaugaca........................  7   0  S02
......................................................................gugauuccggaccaaugaGa........................  1   1  S02
......................................................................gugauuccggaccaaAgaca........................  1   1  S02
......................................................................gugauuccggaUcaaugaca........................  1   1  S02
......................................................................gugauuccggaccaaugacG........................  1   1  S02
......................................................................gugauuccggaccaaugacaa.......................  4   0  S02
......................................................................gugauuccggaccaaugacaag......................  2   0  S02
......................................................................gugauuccggaccaaugacaGg......................  1   1  S02
......................................................................gugauuccggaccaaAgacaagg.....................  2   1  S02
......................................................................gugauuccggaccaaugacaagg.....................  5   0  S02
......................................................................gugauuccggaccaaugacaaggC....................  10  1  S02
......................................................................gugauuccggaccaaugacaaggu....................  80  0  S02
......................................................................gugauuccggaccaaugacaaggA....................  2   1  S02
......................................................................gugauuccgAaccaaugacaaggu....................  1   1  S02
......................................................................gugauuccggGccaaugacaaggu....................  1   1  S02
......................................................................gugauuccggaccaaugacaGggu....................  3   1  S02
......................................................................gugauuccggaccaaAgacaaggu....................  5   1  S02
......................................................................gugauuccCgaccaaugacaaggu....................  1   1  S02
......................................................................gugauuccggaccaaugacaaggG....................  6   1  S02
......................................................................gugauuccggaccaaugGcaaggu....................  1   1  S02
......................................................................gugauuccggaccaaugacaagguA...................  1   1  S02
......................................................................gugauuccggaccaaugacaagguguu.................  1   0  S02
.......................................................................ugauuccggaccaaugacaagg.....................  1   0  S02
.......................................................................ugauuccggaccaaugacaaggC....................  1   1  S02
.......................................................................ugauuccggaccaaugacaaggu....................  4   0  S02
........................................................................gauuccggaccaaugacaGg......................  1   1  S02
........................................................................gauuccggaccaaugacaGgg.....................  1   1  S02
........................................................................gauuccggaccaaAgacaagg.....................  1   1  S02
........................................................................gauuccggaccaaugacaagg.....................  4   0  S02
........................................................................gauuccggaccaaugacaGggu....................  1   1  S02
........................................................................gauuccggaccaaugacaaggu....................  2   0  S02
........................................................................gauuccggaccaaugacaaggC....................  1   1  S02
........................................................................gauuccggaccaaugacaaggug...................  3   0  S02
........................................................................gauuccggaccaaugacaaggGgu..................  1   1  S02
........................................................................Aauuccggaccaaugacaaggugu..................  2   1  S02
........................................................................gauuccggaccaaugacaaggugG..................  3   1  S02
........................................................................gauuccggaccaaugacaaggugu..................  70  0  S02
```

```
                                  Star                                Mature
uacagauugucuuccucuuaaaauuaaggcccaguuuggcaugcuuucccaguuguuuuuggggccaaaagugauuccggaccaaugacaagguguuugguaagauuguagaga
........................................................................gauuccggacUaaugacaaggugu..................   1    1   S02
........................................................................gauuccggaccaGugacaaggugu..................   1    1   S02
........................................................................gauuccggaccaaugacaaggugC..................   4    1   S02
........................................................................gauuccggaccaaugacaGggugu..................   14   1   S02
........................................................................gauuccggaccaUugacaaggugu..................   1    1   S02
........................................................................gauuccggaccaaAgacaaggugu..................   2    1   S02
........................................................................gauuccggaccaaugacaCggugu..................   1    1   S02
........................................................................Uauuccggaccaaugacaaggugu..................   1    1   S02
........................................................................gauuccggaccaaugacaagguguu.................   1    0   S02
.........................................................................auuccggaccaaugacaGggugu...................   1    1   S02
.........................................................................auuccggaccaaugacaagguguC.................   1    1   S02
.........................................................................auuccggaccaaugacaagguguu.................   3    0   S02
.........................................................................auuccggaccaaAgacaagguguu.................   1    1   S02
..........................................................................uuccggaccaaugacaagg.....................   2    0   S02
..........................................................................uuccggaccaaugacaaggug...................   2    0   S02
..........................................................................uuccggaccaaugacaaggugu..................   2    0   S02
..........................................................................uuccggaccaaugacaagguguu.................   1    0   S02
..........................................................................uuccggaccaaugacaGgguguu.................   2    1   S02
..........................................................................uCccggaccaaugacaagguguu.................   3    1   S02
..........................................................................uuccggaccaaugacaGgguguuu................   2    1   S02
..........................................................................uuccggaccaaugacaaggAguuu................   1    1   S02
..........................................................................uuccggaccaaugacaagguguuu................   2    0   S02
...........................................................................uccggaccaaugacaagg.....................   1    0   S02
...........................................................................uccggaccaaugacaaggu....................   1    0   S02
...........................................................................uccggaccaaugacaaggug...................   1    0   S02
...........................................................................uccggaccaaugacaaggugu..................   1    0   S02
...........................................................................Cccggaccaaugacaagguguuu................   1    1   S02
............................................................................ccggaccaaugacaaggug...................   1    0   S02
............................................................................ccggaccaaugacaagguguuuggu.............   1    0   S02
.............................................................................cggaccaaugacaagguguuuggu.............   1    0   S02
```

Provisional ID	:	Maple_Unigene_BMK.23022_69686
Score total	:	2.5
Score for star read(s)	:	3.9
Score for read counts	:	-4.9
Score for mfe	:	0.5
Score for randfold	:	0
Score for cons. seed	:	3
Total read count	:	2
Mature read count	:	1
Loop read count	:	0
Star read count	:	1

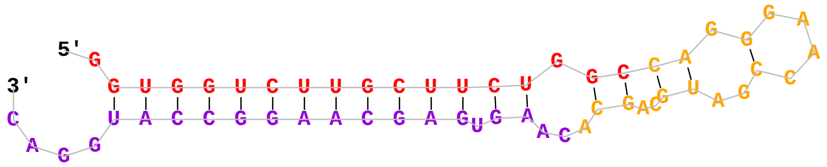

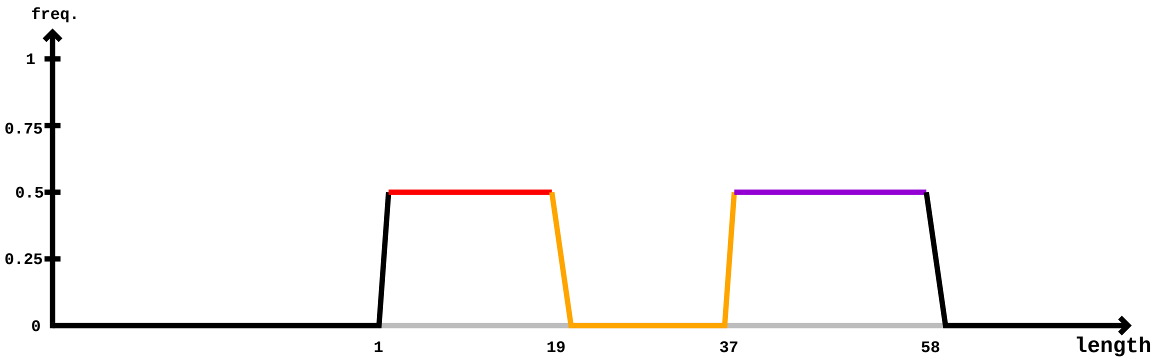

Mature   Star

```
5'- gagccucucaaagggagaugugguaaagggaaaggugguucuugcuucuggccagggaaccgaugcagcacaagugagcaaggccauggacuuggcgacagcauuugcguc  -3'   obs
    gagccucucaaagggagaugugguaaagggaaaggugguucuugcuucuggccagggaaccgaugcagcacaagugagcaaggccauggacuuggcgacagcauuugcguc        exp
    ....((((.....))))..((.((.(((......(((((((((((((.((((.(....)..))..))...)).)))))))))))...))).)).)).((....))...   reads   mm   sample
    .................................ggugguucuugcuucuggc...........................................................   1       0    S02
    .....................................................................caagugagcaaggccauggaU....................   1       1    S02
```

Provisional ID	:	Maple_Unigene_BMK.24035_76508
Score total	:	2.4
Score for star read(s)	:	-1.3
Score for read counts	:	0
Score for mfe	:	0.7
Score for randfold	:	0
Score for cons. seed	:	3
Total read count	:	17
Mature read count	:	16
Loop read count	:	1
Star read count	:	0

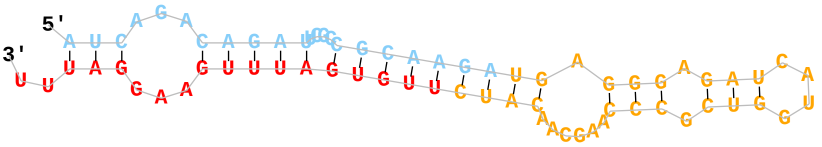

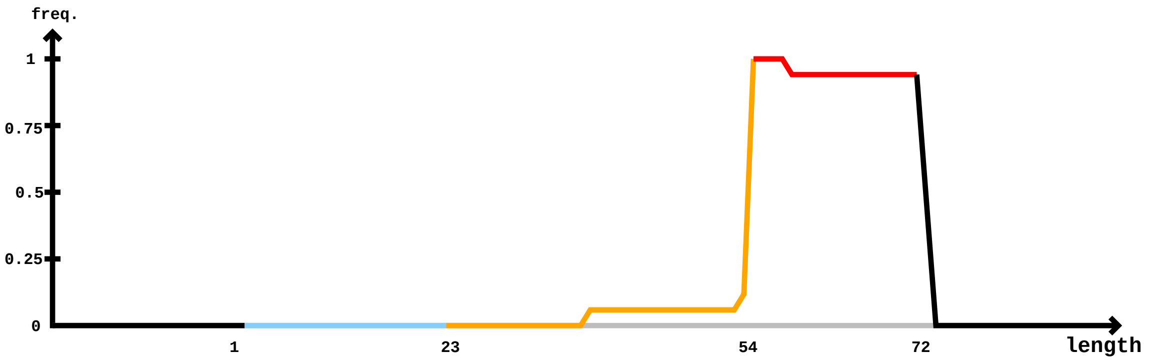

Star Mature

```
5'- uaugcgcaauccagccagaucagacagauucgccgcaagaugagggagaucauggucgcccaagcaacaucuugugauuugaaggauuugguccuaaaguucauuccug   -3'    exp
    .(((.((......((((((((...(((((....(((((((((.(((.(((....))).)))......))))))))))))))...))))))))......)).))).....     reads      mm        sample
    ......................................................gucgcccaagcaacaucuugu..................................     1          0         S01
    .......................................................................uugugauuuAaaggauuu....................     8          1         S01

    ......................................................................cuugugauuugaaggauuu....................     1          0         S02
    .......................................................................uugugauuuAaaggauuu....................     7          1         S02
```

Provisional ID	:	Maple_Unigene_BMK.24178_77414
Score total	:	0.5
Score for star read(s)	:	-1.3
Score for read counts	:	0
Score for mfe	:	-1.1
Score for randfold	:	0
Score for cons. seed	:	3
Total read count	:	15
Mature read count	:	15
Loop read count	:	0
Star read count	:	0

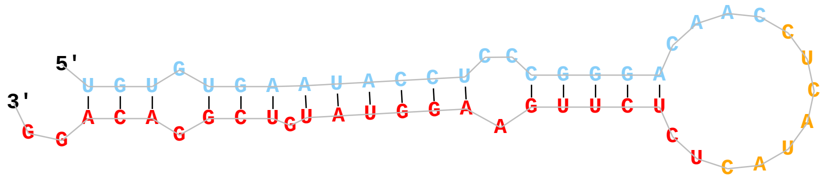

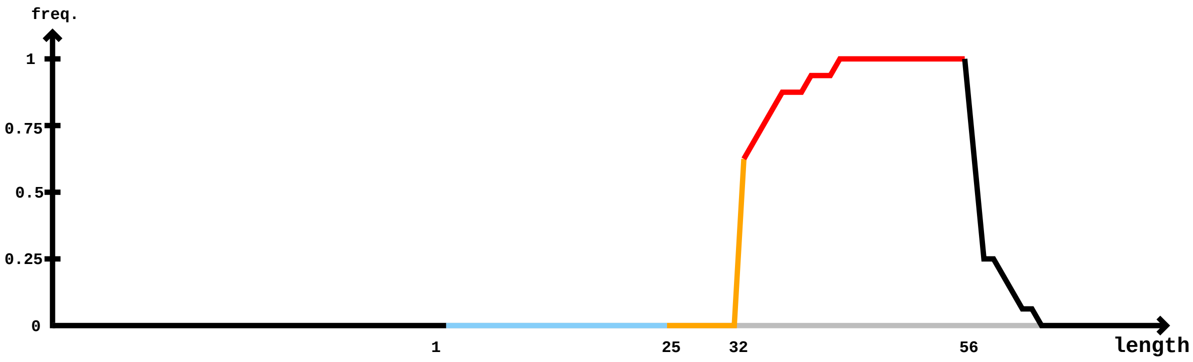

Star Mature

```
5'- auccuugaucacaacuuuuuuccuucccugaaaguaccuugugugaauaccucccgggacaaccucauacucucuugaagguaugucggacaggcggccaaauuucaggcccgu   -3'    exp
    ............((((((..........))))))..(((((.(((((((((..(((((............))))).)))))).))).))))).((((........))))...    reads     mm     sample
    ......................................................................ucucuugaagguaugucggacagg....................    6         0      S01
    .......................................................................cucuugaagguauguAggacagg....................    1         1      S01
    .............................................................................aagguaugucggacaggcggcA...............    1         1      S01

    ......................................................................ucucuugaagguaugucggacagg....................    4         0      S02
    ........................................................................ucuugaagguaugucggacaggc...................    1         0      S02
    .........................................................................cuugaagguaugucggacaggcgg.................    1         0      S02
    ..........................................................................uugaagguaugucggacaggcggU................    1         1      S02
    ................................................................................guaugucggacaggcgAccaa.............    1         1      S02
```

Provisional ID	:	Maple_Unigene_BMK.24259_77884
Score total	:	0.3
Score for star read(s)	:	-1.3
Score for read counts	:	0
Score for mfe	:	2.2
Score for randfold	:	0
Score for cons. seed	:	-0.6
Total read count	:	4903
Mature read count	:	4848
Loop read count	:	0
Star read count	:	55

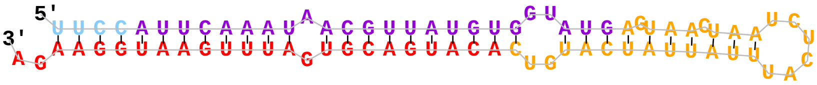

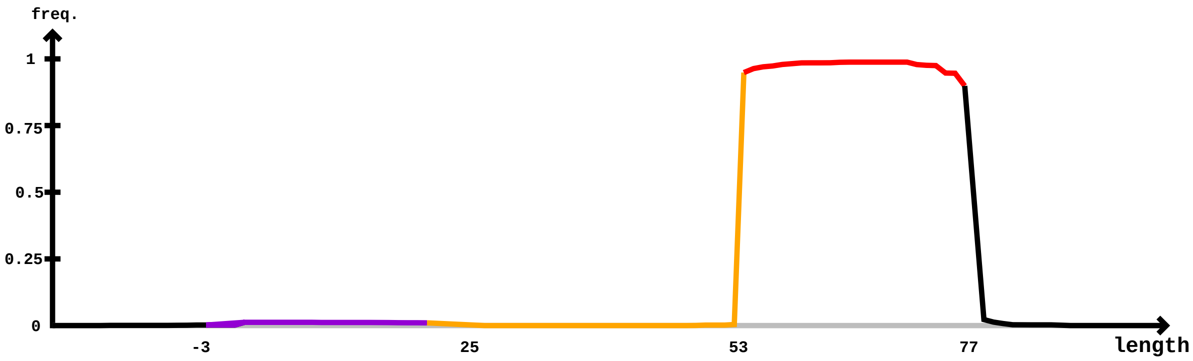

Star **Mature**

```
5'- auauacuguguauuuuccauucaaauaacguuaugugguaugaguaacuaaucucauuuauuaucaugucacaugacgugauuugaauggaagauacacgaugcaacguaugca   -3'   obs
    auauacuguguauuuuccauucaaauaacguuaugugguaugaguaacuaaucucauuuauuaucaugucacaugacgugauuugaauggaagauacacgaugcaacguaugca         exp
    .......((((((((((((((((((.((((((((((..((((.(((.(((......))))))))))..)))))))))).))))))))))))))))))..((((.....))))    reads   mm    sample
    ....acCguguauuuuccauucaaau........................................................................................    1       1     S01
    ...........auuuuccauucaaauaacguuGu................................................................................    1       1     S01
    ............uuuuccauucaaauaacguuG.................................................................................    1       1     S01
    .............uuuccauucaaauaacUuu..................................................................................    1       1     S01
    .............uuuccauucaaauaacguuGugug.............................................................................    1       1     S01
    ..................auucaaauaacguuauguggua..........................................................................    1       0     S01
    ..................auucaaauaacguuaugugguaug........................................................................    9       0     S01
    ..................auucaaauaacguuGugugguaug........................................................................    16      1     S01
    .................................................................augCcacaugacgugauuugaau..........................    1       1     S01
    ..................................................................ugCcacaugacgugauuugaau..........................    3       1     S01
    ..................................................................ugCcacaugacgugauuugaaugg........................    1       1     S01
    .....................................................................cacaugacgugauuuUaaugga.......................    1       1     S01
    .....................................................................cacaugacAugauuugaaugga.......................    1       1     S01
    .....................................................................cacaugacgugauuugaauggaag.....................    1       0     S01
    ......................................................................acaugacgugauuugaaC..........................    1       1     S01
    ......................................................................acaugacgugauuugaau..........................    20      0     S01
    ......................................................................acGugacgugauuugaau..........................    1       1     S01
    ......................................................................acaugacgugauuugaaug.........................    10      0     S01
    ......................................................................aUaugacgugauuugaaugg........................    1       1     S01
    ......................................................................acaugacgugauuugaaugg........................    1       0     S01
    ......................................................................acaugacgugauuugaaugga.......................    58      0     S01
    ......................................................................acaugacgGgauuugaaugga.......................    1       1     S01
    ......................................................................acaugacgugauuugaauggG.......................    1       1     S01
    ......................................................................acaugacguAauuugaaugga.......................    1       1     S01
    ......................................................................acaugacgGgauuugaauggaa......................    1       1     S01
    ......................................................................acaugacgugauuugaauggaa......................    2       0     S01
    ......................................................................acaugacgugauuugaauAgaag.....................    1       1     S01
    ......................................................................acaugacgugauuugaauggaGg.....................    1       1     S01
    ......................................................................acaugaAgugauuugaauggaag.....................    1       1     S01
    ......................................................................acaugacgugauuugaGuggaag.....................    1       1     S01
    ......................................................................acaugacgugauuugaauggaaA.....................    4       1     S01
    ......................................................................acaugacgugauuugCauggaag.....................    1       1     S01
    ......................................................................acaugacguAauuugaauggaag.....................    1       1     S01
```

Star **Mature**

```
auauacuguguauuuuccauucaaauaacguuaugugguaugaguaacuaaucucauuuauuaucaugucacaugacgugauuugaauggaagauacacgaugcaacguaugca
......................................................................acaugacgugCuuugaauggaag.....................	1	1	S01
......................................................................acaugacgugauuugaauggaag.....................	80	0	S01
......................................................................acauUacgugauuugaauggaag.....................	1	1	S01
......................................................................acaCgacgugauuugaauggaag.....................	1	1	S01
......................................................................acaugacgugGuuugaauggaag.....................	1	1	S01
......................................................................acaugacgugGuuugaauggaaga....................	8	1	S01
......................................................................Ccaugacgugauuugaauggaaga....................	1	1	S01
......................................................................acaugacgugauuugaGuggaaga....................	7	1	S01
......................................................................acaugacgugCuuugaauggaaga....................	4	1	S01
......................................................................acaugacgugauuugaaGggaaga....................	2	1	S01
......................................................................acaugacgugauuugaauggaagU....................	9	1	S01
......................................................................acaugacgugauuugaauggaaAa....................	4	1	S01
......................................................................acaugacgugauuCgaauggaaga....................	7	1	S01
......................................................................acaugaAgugauuugaauggaaga....................	1	1	S01
......................................................................acaugacguUauuugaauggaaga....................	2	1	S01
......................................................................Gcaugacgugauuugaauggaaga....................	6	1	S01
......................................................................acaugacgugauuugaaugCaaga....................	1	1	S01
......................................................................acaugacgugaCuugaauggaaga....................	3	1	S01
......................................................................acUugacgugauuugaauggaaga....................	1	1	S01
......................................................................acaugacgugauuugaauggaagG....................	24	1	S01
......................................................................acaugacgugauuuUaauggaaga....................	3	1	S01
......................................................................acaugCcgugauuugaauggaaga....................	1	1	S01
......................................................................acaugacgugauuuAaauggaaga....................	3	1	S01
......................................................................acaugaGgugauuugaauggaaga....................	1	1	S01
......................................................................acaugaUgugauuugaauggaaga....................	2	1	S01
......................................................................aAaugacgugauuugaauggaaga....................	1	1	S01
......................................................................aGaugacgugauuugaauggaaga....................	1	1	S01
......................................................................acaugacgugauuugaaCggaaga....................	7	1	S01
......................................................................acaugacgugauuugaauggaGga....................	8	1	S01
......................................................................acaugacgugauuugaauggaaUa....................	1	1	S01
......................................................................Ucaugacgugauuugaauggaaga....................	1	1	S01
......................................................................acaAgacgugauuugaauggaaga....................	1	1	S01
......................................................................acaCgacgugauuugaauggaaga....................	2	1	S01
......................................................................acaugacgGgauuugaauggaaga....................	3	1	S01
......................................................................acaugacgugaAuugaauggaaga....................	3	1	S01
......................................................................acaugacgugauCugaauggaaga....................	2	1	S01
......................................................................acaugacgugauuugaauggGaga....................	12	1	S01
......................................................................acaugacgugauuGgaauggaaga....................	2	1	S01
......................................................................acaugacgugauuugaauCgaaga....................	1	1	S01
......................................................................acaugacguCauuugaauggaaga....................	1	1	S01
......................................................................acaugacgugauuugaCuggaaga....................	2	1	S01
......................................................................acaugacgugUuuugaauggaaga....................	2	1	S01
......................................................................acaugacgugauuugaauggaaCa....................	3	1	S01
......................................................................acaugacgugauuugaauAgaaga....................	2	1	S01
......................................................................acaugGcgugauuugaauggaaga....................	5	1	S01
......................................................................acaugacgCgauuugaauggaaga....................	8	1	S01
......................................................................acaugacAugauuugaauggaaga....................	2	1	S01
......................................................................acaugacgugauuugaauggCaga....................	2	1	S01
......................................................................acaGgacgugauuugaauggaaga....................	4	1	S01
......................................................................acGugacgugauuugaauggaaga....................	4	1	S01
......................................................................acauAacgugauuugaauggaaga....................	2	1	S01
......................................................................acaugacgAgauuugaauggaaga....................	1	1	S01
......................................................................acaugacgugauuugaauggaaga....................	1774	0	S01
......................................................................acCugacgugauuugaauggaaga....................	1	1	S01
......................................................................acaugacgugauAugaauggaaga....................	3	1	S01
......................................................................acaugacCugauuugaauggaaga....................	1	1	S01
......................................................................acaugacgugauuugGauggaaga....................	7	1	S01
......................................................................acaugacgugauuugaauUgaaga....................	1	1	S01
......................................................................acaugacgugauuAgaauggaaga....................	3	1	S01
......................................................................Ncaugacgugauuugaauggaaga....................	2	1	S01
......................................................................acaugUcgugauuugaauggaaga....................	1	1	S01
......................................................................acaugacgugauuugaauggaagaA...................	3	1	S01
......................................................................acaugacgugauuugaauggaagau...................	18	0	S01
......................................................................acaugacgugauuugaauggaagaC...................	2	1	S01
......................................................................acaugacgugauuugaauggaagauU..................	6	1	S01
.......................................................................caugacgugauuugaaugga.......................	1	0	S01
.......................................................................caugacgugauuugaauggaag.....................	2	0	S01
.......................................................................caugacgugauuugaauggaaga....................	9	0	S01
.......................................................................cGugacgugauuugaauggaagau...................	1	1	S01
.......................................................................caugacgugauuugaauggaagau...................	28	0	S01
```

Star **Mature**

```
auauacuguguauuuuccauucaaauaacguuaugugguaugaguaacuaaucucauuuauuaucaugucacaugacgugauuugaauggaagauacacgaugcaacguaugca
.......................................................................caugacgugauuugaauggaagaG...................  1    1    S01
.......................................................................caugacgugauuugaauggaagaC...................  6    1    S01
.......................................................................caugacAugauuugaauggaagau...................  1    1    S01
.......................................................................caugacgugauuugaauggaagauU..................  2    1    S01
.......................................................................caugacgugauuugaauggaagauC..................  1    1    S01
.......................................................................caugacgugauuugaauggaagauG..................  1    1    S01
........................................................................augacAugauuugaaugga.......................  1    1    S01
........................................................................augacAugauuugaauggaa......................  1    1    S01
........................................................................augacgugauuugaauggaaga....................  2    0    S01
........................................................................augacgugauuugaauggaagau...................  2    0    S01
........................................................................augacgugauuugaauggaagauU..................  2    1    S01
........................................................................augacgugauuugaauggaagaua..................  6    0    S01
.........................................................................ugacgugauuugaauggaaga....................  3    0    S01
.........................................................................ugacgugauuugaauggaagauU..................  1    1    S01
.........................................................................ugacgugauuugaauggaagauac.................  3    0    S01
.........................................................................ugacgugauuugaauggaagauacC................  1    1    S01
..........................................................................gacgugauuugaaGggaaga....................  1    1    S01
..........................................................................gaUgugauuugaauggaagauac.................  1    1    S01
..........................................................................gacgugauuugaauggaagauaU.................  1    1    S01
..........................................................................gacgugauuugaauggaagauac.................  1    0    S01
..........................................................................gaGgugauuugaauggaagauac.................  1    1    S01
..........................................................................gacgugauuugaauggaGgauac.................  1    1    S01
..........................................................................gaUgugauuugaauggaagauaca................  1    1    S01
..........................................................................gacgugauuugaauggaagauaca................  8    0    S01
..........................................................................gacgugauuugaauggaagauacaA...............  1    1    S01
...........................................................................acgugauuugaauggaaga....................  1    0    S01
...........................................................................acgugauuugaauggaagaua..................  1    0    S01
...........................................................................acgugauuugaauggaagauaca................  1    0    S01
............................................................................cgugauuugaauggaaga....................  2    0    S01
............................................................................cguAauuugaauggaagauac.................  1    1    S01
............................................................................cgugauuugaauggaagauac.................  6    0    S01
...............................................................................gauuugaauggaagauacacgaug...........  1    0    S01
................................................................................auuugaauggaagauacacgaug...........  1    0    S01
................................................................................auuugaauggaagauacacgaugc..........  2    0    S01
................................................................................auuugaauggaagauacacgaugU..........  1    1    S01
..................................................................................uugaauggaagauacacgaug...........  2    0    S01

....acCguguauuuuccauucaaau........................................................................................  2    1    S02
...........auuuuccauucaaauaacguuGug...............................................................................  1    1    S02
.............uuccauucaaauaacguuGu.................................................................................  1    1    S02
.............uuccauucaaauaacguuGugug..............................................................................  1    1    S02
..................auucaaauaacguuGugugguaug........................................................................  14   1    S02
..................auucaaauaacguuaugugguaCg........................................................................  2    1    S02
..................auucaaauaacguuaugugguaug........................................................................  8    0    S02
.................................................................augCcacaugacgugauuugaaug.........................  1    1    S02
..................................................................ugCcacaugacgugauuugaau..........................  3    1    S02
.....................................................................cacaugacgugauuugaaugga.......................  2    0    S02
.....................................................................cacaugacgugauuugaauggaag.....................  5    0    S02
......................................................................acGugacgugauuugaau..........................  1    1    S02
......................................................................acaugacgugauuugaaC..........................  1    1    S02
......................................................................acaugacgugauuugaau..........................  14   0    S02
......................................................................acaugacgugGuuugaaug.........................  1    1    S02
......................................................................acaugacgugauuugaaug.........................  2    0    S02
......................................................................acaugacgugauuugaaugg........................  2    0    S02
......................................................................acaugacgugGuuugaaugga.......................  2    1    S02
......................................................................Ucaugacgugauuugaaugga.......................  1    1    S02
......................................................................acaugacgugauuugaauCga.......................  1    1    S02
......................................................................acaugacgugaGuugaaugga.......................  1    1    S02
......................................................................acaugacgugauuugaauggG.......................  2    1    S02
......................................................................acaugacgugauuugaaugga.......................  60   0    S02
......................................................................acauAacgugauuugaaugga.......................  1    1    S02
......................................................................acaCgacgugauuugaaugga.......................  1    1    S02
......................................................................acaugGcgugauuugaauggaag.....................  1    1    S02
......................................................................acaugacgugauuugaCuggaag.....................  1    1    S02
......................................................................acaugacgugauuugaauggaag.....................  110  0    S02
......................................................................acaugacgugauuugaauggaGg.....................  2    1    S02
......................................................................acaugacgugauuuCaauggaag.....................  1    1    S02
......................................................................acaugacgugauCugaauggaag.....................  1    1    S02
......................................................................acaugacgugauuugaauggUag.....................  1    1    S02
......................................................................acaugacgGgauuugaauggaag.....................  1    1    S02
```

Star Mature

```
auauacuguguauuuuccauucaaauaacguuaugugguaugaguaacuaaucucauuuauuaucaugucacaugacgugauuugaauggaagauacacgaugcaacguaugca
......................................................................acaugacgAgauuugaauggaag.....................  1     1  S02
......................................................................aAaugacgugauuugaauggaag.....................  1     1  S02
......................................................................acaugacgugauuugaauAgaag.....................  1     1  S02
......................................................................acaugacgugauuugaauggaaA.....................  5     1  S02
......................................................................acaCgacgugauuugaauggaag.....................  1     1  S02
......................................................................acaugacgugauuugaaugAaag.....................  1     1  S02
......................................................................acaugacgugauuugaauggaagG....................  89    1  S02
......................................................................acaugacgugauuugaauUgaaga....................  1     1  S02
......................................................................acaugacgugauuGgaauggaaga....................  3     1  S02
......................................................................acaugacgugauuugaauggaGga....................  8     1  S02
......................................................................acaugacgugauuugaauggaaUa....................  1     1  S02
......................................................................acaAgacgugauuugaauggaaga....................  1     1  S02
......................................................................acaugacgugauuugCauggaaga....................  1     1  S02
......................................................................acaugacgGgauuugaauggaaga....................  1     1  S02
......................................................................acaugacgugauuugaaugAaaga....................  6     1  S02
......................................................................acaugacgugauuugaCuggaaga....................  1     1  S02
......................................................................acaugacguUauuugaauggaaga....................  1     1  S02
......................................................................acaugacgugauuugaauggaagU....................  16    1  S02
......................................................................Ucaugacgugauuugaauggaaga....................  1     1  S02
......................................................................acaugacgugauuugaaAggaaga....................  2     1  S02
......................................................................Ncaugacgugauuugaauggaaga....................  1     1  S02
......................................................................acaugacgugauCugaauggaaga....................  4     1  S02
......................................................................aGaugacgugauuugaauggaaga....................  1     1  S02
......................................................................acGugacgugauuugaauggaaga....................  7     1  S02
......................................................................acaugacgugaGuugaauggaaga....................  1     1  S02
......................................................................acaugCcgugauuugaauggaaga....................  1     1  S02
......................................................................acaugacgugauuCgaauggaaga....................  1     1  S02
......................................................................acaugacgugauuugaaugCaaga....................  1     1  S02
......................................................................acaugacguCauuugaauggaaga....................  2     1  S02
......................................................................acaugacgugauuugaauggaaga....................  1930  0  S02
......................................................................acaugacAugauuugaauggaaga....................  5     1  S02
......................................................................acaugacgugUuuugaauggaaga....................  1     1  S02
......................................................................acaugacgugauuugaauggUaga....................  1     1  S02
......................................................................acaugacgugCuuugaauggaaga....................  3     1  S02
......................................................................acaugacgugauuugaauggaUga....................  1     1  S02
......................................................................acaugacgugauuugaaGggaaga....................  2     1  S02
......................................................................acaugaAgugauuugaauggaaga....................  2     1  S02
......................................................................aUaugacgugauuugaauggaaga....................  4     1  S02
......................................................................Gcaugacgugauuugaauggaaga....................  4     1  S02
......................................................................acaugacgugauuugaauggGaga....................  7     1  S02
......................................................................acaugacCugauuugaauggaaga....................  1     1  S02
......................................................................acaugacguAauuugaauggaaga....................  1     1  S02
......................................................................acaugacgugaCuugaauggaaga....................  5     1  S02
......................................................................acaugacgugauuugaaCggaaga....................  5     1  S02
......................................................................acaugaUgugauuugaauggaaga....................  1     1  S02
......................................................................acaugacgugauuugaauggaaAa....................  9     1  S02
......................................................................acaugacUugauuugaauggaaga....................  1     1  S02
......................................................................acaugacgugauGugaauggaaga....................  2     1  S02
......................................................................acaugacgugauuugGauggaaga....................  7     1  S02
......................................................................acaugacgugauuugaGuggaaga....................  9     1  S02
......................................................................acaugacgugauuugaauggaagC....................  3     1  S02
......................................................................acaugacgugauuugaauAgaaga....................  3     1  S02
......................................................................acaugGcgugauuugaauggaaga....................  7     1  S02
......................................................................acaugacgugauuuUaauggaaga....................  6     1  S02
......................................................................acaugacgugGuuugaauggaaga....................  9     1  S02
......................................................................acaugUcgugauuugaauggaaga....................  1     1  S02
......................................................................acaugacgugauuuCaauggaaga....................  1     1  S02
......................................................................acaCgacgugauuugaauggaaga....................  3     1  S02
......................................................................acaugacgCgauuugaauggaaga....................  3     1  S02
......................................................................acaGgacgugauuugaauggaaga....................  7     1  S02
......................................................................acaugacgugauuAgaauggaaga....................  1     1  S02
......................................................................acaugacgugaAuugaauggaaga....................  3     1  S02
......................................................................acauAacgugauuugaauggaaga....................  2     1  S02
......................................................................acaugacgugauuugUauggaaga....................  4     1  S02
......................................................................acaugacgAgauuugaauggaaga....................  8     1  S02
......................................................................acaugGcgugauuugaauggaagau...................  1     1  S02
......................................................................acaugacguAauuugaauggaagau...................  1     1  S02
......................................................................acaugacgugauuCgaauggaagau...................  1     1  S02
......................................................................acaugacgugauuugaauggaagaG...................  3     1  S02
......................................................................acaugacgugauuugaauggaagaA...................  9     1  S02
```

Star **Mature**

auauacuguguauuuuccauucaaauaacguuaugugguaugaguaacuaaucucauuuauuaucaugucacaugacgugauuugaauggaagauacacgaugcaacguaugca			
....acaugacgugauAugaauggaagau...................	1	1	S02
....acaugacgugauuugaauggaagau...................	19	0	S02
....acaugacgugauuugaauggaagaC...................	3	1	S02
....acaugacgGgauuugaauggaagau...................	1	1	S02
....acaugacgugauuugaauggaagauU..................	1	1	S02
....acaugacgugauuugaauggaagauC..................	1	1	S02
....caugacgugauuugaaugga.......................	1	0	S02
....caugacgugauuugaauggaag.....................	2	0	S02
....caugacgGgauuugaauggaag.....................	1	1	S02
....caugacgugauuugaauggaaga....................	4	0	S02
....caugacgugauuugaauggaagau...................	8	0	S02
....caugacgugauuugaauggaagaA...................	1	1	S02
....caugacgugauuugaauggaagauU..................	3	1	S02
....augacgugauuugaaugga.......................	1	0	S02
....augacgugauuugaauggaagau...................	3	0	S02
....augacgugauuugaauggaagauU..................	2	1	S02
....augacgugauuugaaCggaagaua..................	1	1	S02
....augGcgugauuugaauggaagaua..................	1	1	S02
....augacgCgauuugaauggaagaua..................	1	1	S02
....augacgugauuugaauggaagaua..................	10	0	S02
....ugacgugauuugaauggaaga....................	2	0	S02
....ugacgugauuugaauggaagauU..................	2	1	S02
....ugacgugauuugaauggaagauaU.................	1	1	S02
....ugacgugauuugaauggaagauac.................	3	0	S02
....gacgugauuugaauggGagauac.................	1	1	S02
....gacgugauuugaauggaagauac.................	2	0	S02
....gacgugauuugaauggaagauacG................	2	1	S02
....gacgugauuugaauggaaAauaca................	1	1	S02
....gaUgugauuugaauggaagauaca................	1	1	S02
....gacgugauuugaauggaagauacC................	2	1	S02
....gacgugauuugaauggaagauaca................	3	0	S02
....acgugauuugaauggaag.....................	1	0	S02
....acgugauuugaauggaaga....................	2	0	S02
....acgugauuugaauggaagau...................	2	0	S02
....acgugaGuugaauggaagaua..................	1	1	S02
....acgugauuugaauggaagaua..................	3	0	S02
....acgugauuugaauggaagauaca................	1	0	S02
....acgugauuugaauggaagauacacU..............	1	1	S02
....cgugauuugaauggaagaua..................	1	0	S02
....cgugauuugaauUgaagauac.................	1	1	S02
....cgugauuugaauggaagauUc.................	1	1	S02
....cgugauuugaauggaagauac.................	1	0	S02
....cgugauuugaauggaagGuac.................	1	1	S02
....cgugauuugaauggGagauac.................	1	1	S02
....gugauuugaauggaagauacU................	1	1	S02
....auuugaauggaagauacacgaug...........	2	0	S02
....auuugaauggaagauacacgaugc..........	3	0	S02
....uuugaauggaagauacacgaugc..........	1	0	S02

Provisional ID	:	Maple_Unigene_BMK.26307_92749
Score total	:	4.4
Score for star read(s)	:	3.9
Score for read counts	:	-4.4
Score for mfe	:	1.9
Score for randfold	:	0
Score for cons. seed	:	3
Total read count	:	3
Mature read count	:	2
Loop read count	:	0
Star read count	:	1

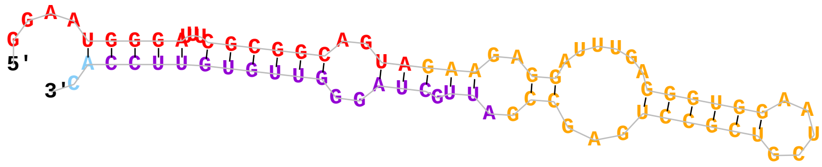

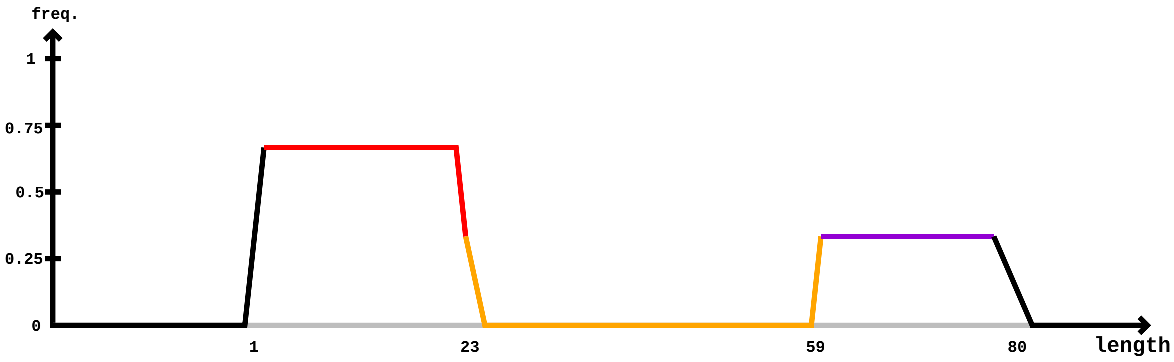

Mature Star

```
5'- caaaaggcgucgugggaggcggaaugggauuucgcggcaguagaagaggauuugaggguggaaucgucgccugagccgauugcuaggguuguguuccacgucccgccgacgc  -3'   obs
    caaaaggcgucgugggaggcggaaugggauuucgcggcaguagaagaggauuugaggguggaaucgucgccugagccgauugcuaggguuguguuccacgucccgccgacgc         exp
    ......((((((((((.(.((((((.(((((...((((((......((......((((((.....))))))...)).)))))).))))).)))))).).))))).))))))  reads   mm   sample
    ...................cggaaugggauuucgcggcagu.......................................................................  1       0    S02
    ....................ggaaugggauuucgcggcagua......................................................................  1       0    S02
    ..............................................................................auugcuaggguuguguucc...............  1       0    S02
```

Provisional ID	:	Maple_Unigene_BMK.27880_105384
Score total	:	2.2
Score for star read(s)	:	-1.3
Score for read counts	:	-0.3
Score for mfe	:	0.8
Score for randfold	:	0
Score for cons. seed	:	3
Total read count	:	11
Mature read count	:	11
Loop read count	:	0
Star read count	:	0

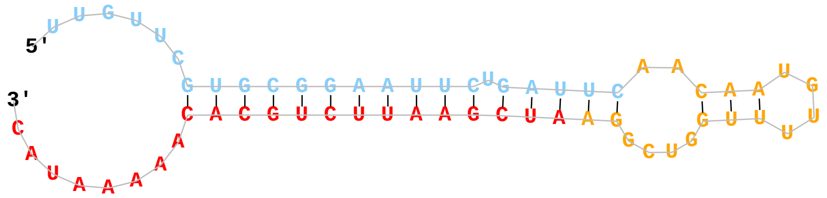

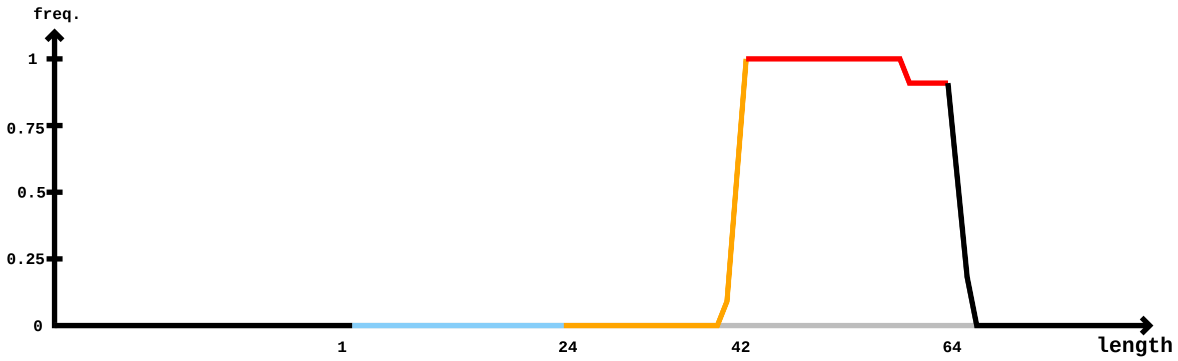

Star Mature

```
5'- uucaaugcuacuagcuauauauaccucaguuguucgugcggaauucugauucaacaauguuuuggucggaaucgaauucugcacaaaaauacagguucggucggaacccgca   -3'   exp
    .............((....................((((((((((.(((((..(((....)))....))))))))))))))))).........(((((.....))))).)).   reads   mm   sample
    ....................................................................gaaucgaauucugcacaaa.........................   1       0    S01
    .....................................................................aaucgaauucugcacaaaaaAac....................   2       1    S01
    .....................................................................aaucgaauucugcacaaaaaAaca...................   1       1    S01
    ......................................................................aucgaauucugcacaaaaauac....................   2       0    S01

    .....................................................................aaucgaauucugcacaaaaaAac....................   1       1    S02
    .....................................................................aaucgaauucugcacaaaaaAaca...................   1       1    S02
    ......................................................................aucgaauucugcacaaaaauac....................   1       0    S02
    ......................................................................aucgaauucugcacaaaaaAacag..................   1       1    S02
    ......................................................................aucgaauucugcacaaaaauacag..................   1       0    S02
```

Provisional ID	:	Maple_Unigene_BMK.28529_110396
Score total	:	3.6
Score for star read(s)	:	3.9
Score for read counts	:	-4.9
Score for mfe	:	1.7
Score for randfold	:	0
Score for cons. seed	:	3
Total read count	:	2
Mature read count	:	1
Loop read count	:	0
Star read count	:	1

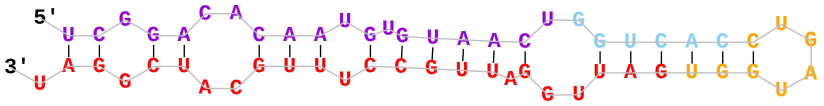

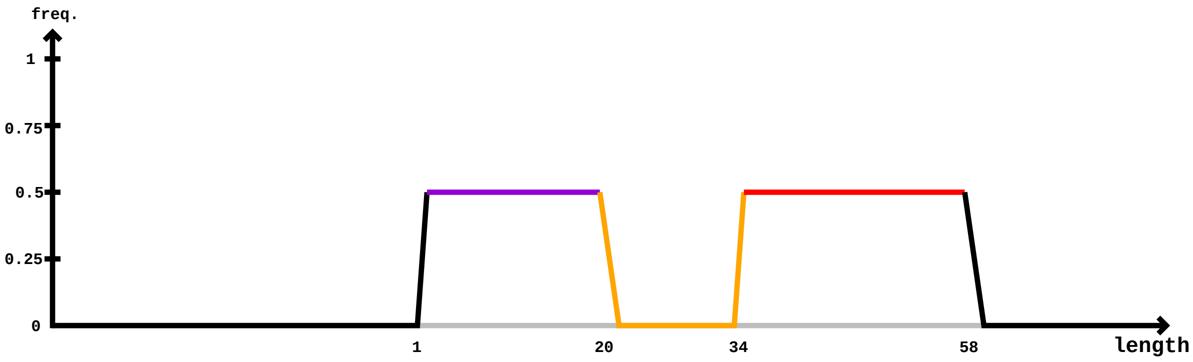

Star Mature

```
5'- gaaagcugggcuccaaagauuaacggaggguccuuggucggacacaauguguaacuggucaccugaugguugauuggauugccuuugcaucggaucgacaugauccggguucggg   -3'    obs
    gaaagcugggcuccaaagauuaacggaggguccuuggucggacacaauguguaacuggucaccugauggugauuggauugccuuugcaucggaucgacaugauccggguucggg          exp
    .....((((((((..............(((((.((((((.((..(((.(.(((((..((((((....))))))..).))))).)))..)).))))))...))))))))))))).   reads      mm          sample
    .....................................ucggacacaauguguaacG..........................................................   1          1           S01
    ......................................................................gauuggauugccuuugcaucggau....................   1          0           S01
```

Provisional ID	:	Maple_Unigene_BMK.29814_121210
Score total	:	2.7
Score for star read(s)	:	3.9
Score for read counts	:	-4.9
Score for mfe	:	0.8
Score for randfold	:	0
Score for cons. seed	:	3
Total read count	:	2
Mature read count	:	1
Loop read count	:	0
Star read count	:	1

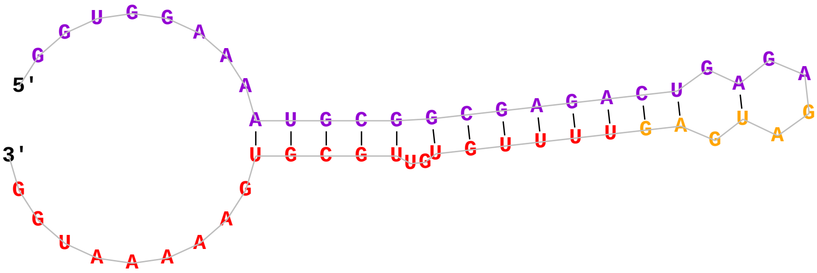

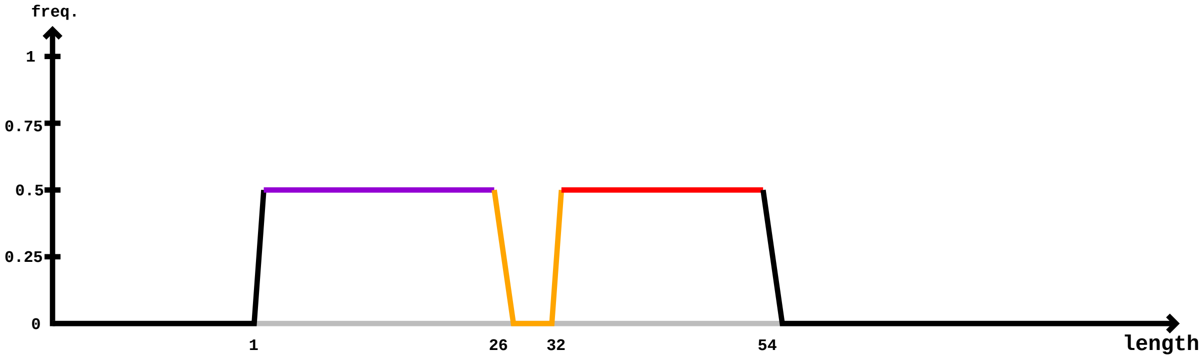

Star Mature

```
5'- gauuugguuucguuuucggcgguggaaaaugcggcgagacugagagaugaguuuguguugcgugaaaaauggaugugguguuucgggagagacgcgucggcgaagcagguaggaa   -3'   obs
    gauuugguuucguuuucggcgguggaaaaugcggcgagacugagagaugaguuuguguugcgugaaaaauggaugugguguuucgggagagacgcgucggcgaagcagguaggaa         exp
    ......(((((((......(.((.....((((((((((((.(....).))))))))..))))).....)).)(((((((.(((....)))))))))).)))))))........   reads   mm   sample
    ....................gguggaaaaugcggcgagacugaga...................................................................   1       0    S01

    ...................................................uuuguguugcgugaaaaaugg.........................................   1       0    S02
```

Provisional ID	:	Maple_Unigene_BMK.30577_128567
Score total	:	1.2
Score for star read(s)	:	-1.3
Score for read counts	:	0
Score for mfe	:	-0.4
Score for randfold	:	0
Score for cons. seed	:	3
Total read count	:	18
Mature read count	:	18
Loop read count	:	0
Star read count	:	0

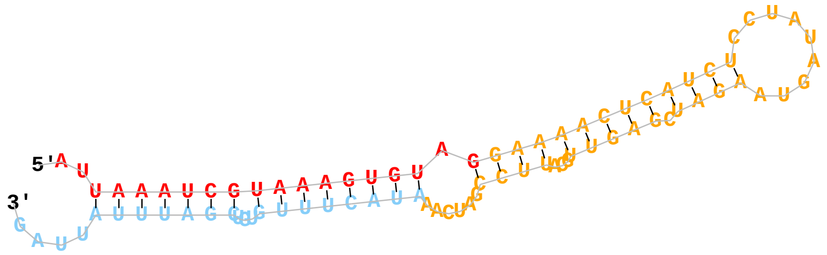

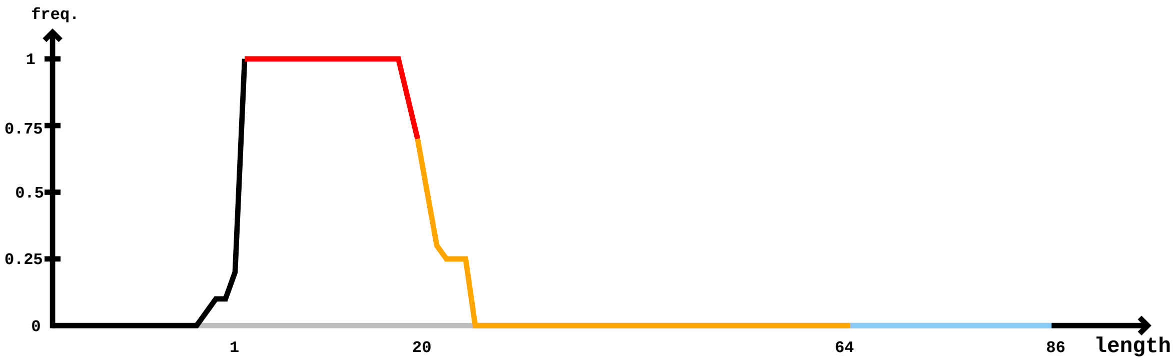

Mature Star

```
5'- ucucacauuugaccguuaauuaaaaucguaaaguguaggaaaacucaucuccuauaguaagaucgaguugcauuccgaucaaauacuuugugucgauuuauuaggucguugua   -3'    exp
    ....(((..(((((......((((((((((((((.((((((((((((.........)))).)))))...))))......))))))))...)))))))...))))).))).   reads   mm   sample
    ...............uuUauuaaaaucguaaagugu.............................................................................   1   1   S01
    .................Cauuaaaaucguaaagugu.............................................................................   1   1   S01
    .................aauuaaaaucguaaaguAuagga.........................................................................   1   1   S01
    ..................auuaaaaucguaaaguguG............................................................................   2   1   S01
    ..................auuaaaaucguaaaguguGg...........................................................................   1   1   S01
    ..................auuaaaaucguaaaguguUg...........................................................................   3   1   S01
    ..................auuaaaaucguaaagugAag...........................................................................   1   1   S01
    ..................auuaaaaucguaaaguguaggaaGa......................................................................   2   1   S01
    ..................auuaaaaucguaaaguguGggaaaa......................................................................   1   1   S01

    ..............guCaauuaaaaucguaaagugu.............................................................................   1   1   S02
    ..................auuaaaaucguaaaguguG............................................................................   1   1   S02
    ..................auuaaaaucguaaaguguGg...........................................................................   1   1   S02
    ..................auuaaaaucguaaaguguGgg..........................................................................   2   1   S02
    ..................auuaaaaucguaaaguguaggaaGa......................................................................   2   1   S02
```

Provisional ID	:	Maple_Unigene_BMK.32446_145133
Score total	:	19
Score for star read(s)	:	3.9
Score for read counts	:	18
Score for mfe	:	-2.1
Score for randfold	:	0
Score for cons. seed	:	-0.6
Total read count	:	47
Mature read count	:	46
Loop read count	:	0
Star read count	:	1

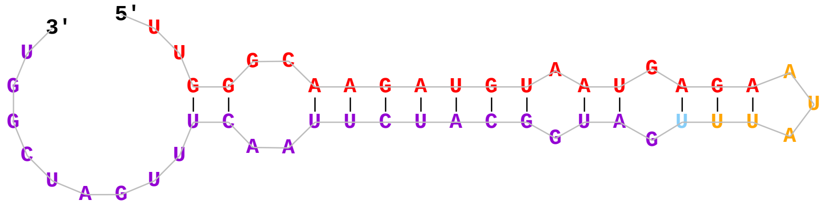

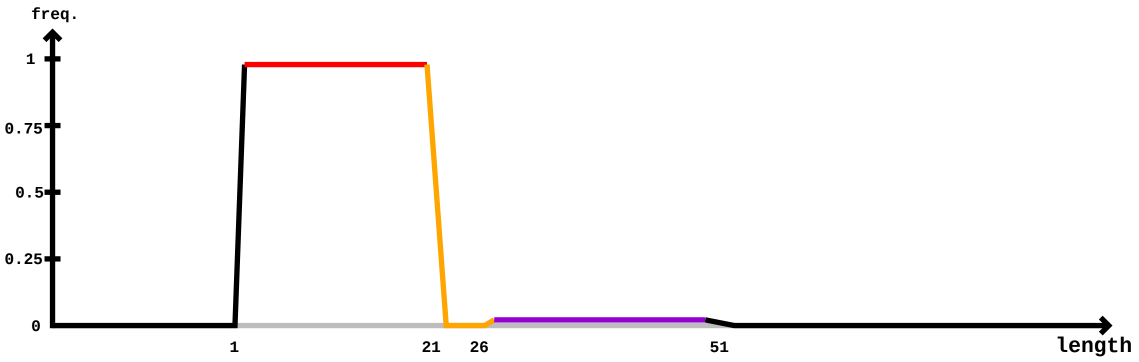

Mature **Star**

```
5'- aacugaggcaugauacaauugggcaagauguaaugagaauauuugauggcaucuuaacuuugaucggucauguuacggguaugaucagaagaacuaccaugagcucua  -3'   obs
    aacugaggcaugauacaauugggcaagauguaaugagaauauuugauggcaucuuaacuuugaucgguucauguuacggguaugaucagaagaacuaccaugagcucua        exp
    ..(((..(((((((......((..(((((((.((.(((...))).)).)))))))..))......)))))))..)))..........(((.((......)).))).   reads   mm   sample
    ..................uugggcaagauguaaugagU......................................................................   1       1    S01
    ..................uugggcaagauguaaugagC......................................................................   1       1    S01
    ..................uugAgcaagauguaaugaga......................................................................   7       1    S01
    ..................uugggcaagauguaaugagCa.....................................................................   1       1    S01

    ..................uugggcaagauguaaugagC......................................................................   3       1    S02
    ..................uugAgcaagauguaaugaga......................................................................   23      1    S02
    ..................uugAgcaagauguaaugagaa.....................................................................   1       1    S02
    ..................uugggcaagauguaaugagCa.....................................................................   9       1    S02
    ............................................gauggcaucuuaacuuugaucggG........................................   1       1    S02
```

Provisional ID	:	Maple_Unigene_BMK.32533_145937
Score total	:	0.5
Score for star read(s)	:	3.9
Score for read counts	:	-2.8
Score for mfe	:	0.1
Score for randfold	:	0
Score for cons. seed	:	-0.6
Total read count	:	6
Mature read count	:	4
Loop read count	:	1
Star read count	:	1

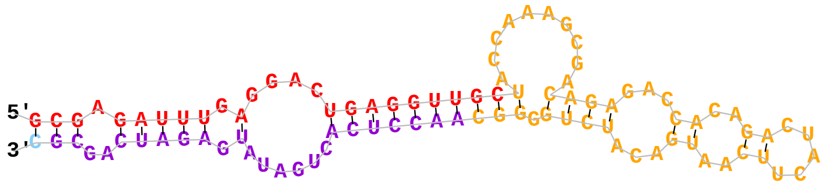

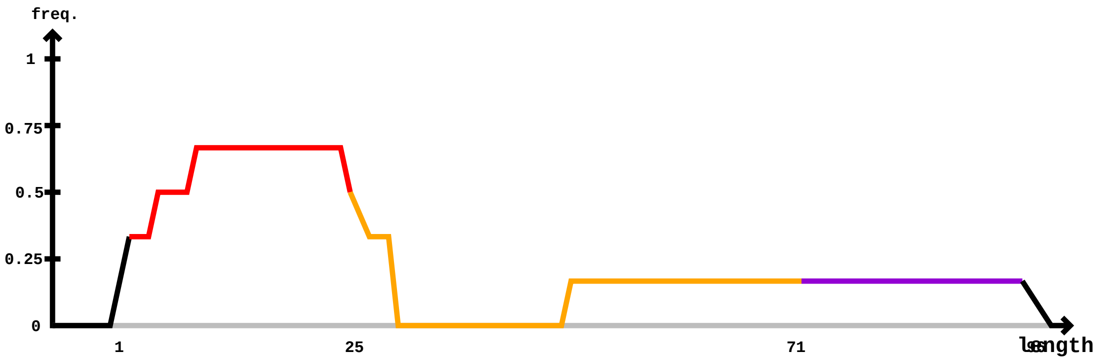

Mature **Star**

```
5'- ggggaugcgagauuugaggacugagguugcuaccaaagcgacagagaccacagacuacuucaaugacaucuggggcaaccucacugauaugagaucagcgcuca  -3'  obs
    ggggaugcgagauuugaggacugagguugcuaccaaagcgacagagaccacagacuacuucaaugacaucuggggcaaccucacugauaugagaucagcgcuca        exp
    .(((.(((..(((((.(....(((((((((..........((((...((..((.....))..))...)))).)))))))))......).))))).)))))).  reads   mm   sample
    ......gcgGgauuugaggacugagguugc..........................................................................  1       1    S01
    ............................................................................aaccucacGgauaugagaucagcg....  1       1    S01

    .....ugcgGgauuugaggacugagguug...........................................................................  1       1    S02
    .........agauuugaggacugagguugcuacc......................................................................  1       0    S02
    ............uugaggacugagguugcuacc.......................................................................  1       0    S02
    ....................................................gacuacuucaaugacaucuggAgc............................  1       1    S02
```

Provisional ID	:	Maple_Unigene_BMK.32845_148680
Score total	:	0.9
Score for star read(s)	:	-1.3
Score for read counts	:	0
Score for mfe	:	-0.7
Score for randfold	:	0
Score for cons. seed	:	3
Total read count	:	44
Mature read count	:	44
Loop read count	:	0
Star read count	:	0

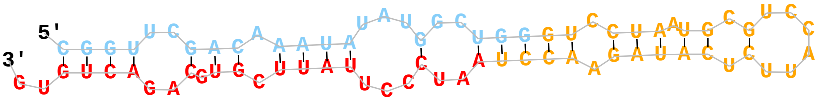

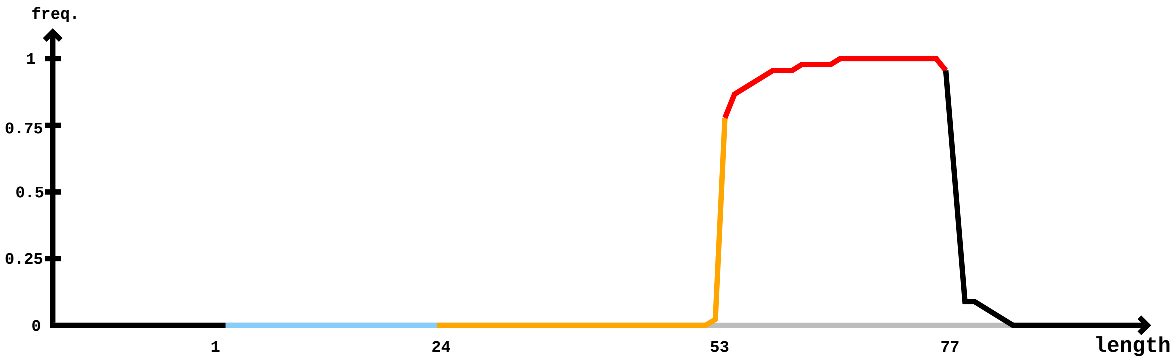

Star Mature

```
5'- ugauucugggguguuuacgguucgacaaauauauggcugguccuaaugcguccauucucauagaaccuaaucccuuauucgugcagacugugaagguggaagaguuaaauga  -3'   exp
    ((((((...(.((((((((..(((.((((...(..(((((.(((.((.(......).))))).)))))..)...)))).)).)..))))))))).)....))))))).....   reads   mm   sample
    ....................................................................uaaucccuuauucgugcagacugu.....................   1       0    S01
    .....................................................................aaucccuuauuAgugcagacugug....................   1       1    S01
    .....................................................................aaucccuuauucgugcagacuguA...................   1       1    S01
    .....................................................................aauGccuuauucgugcagacugug....................   1       1    S01
    .....................................................................aaucccuuauucgugcagacugug....................   10      0    S01
    ......................................................................aucccuuauucgugcagacuguA....................   1       1    S01
    ......................................................................aucccuuauucgugcagacugug....................   1       0    S01
    ......................................................................aucccuuauucgugcagacugugU...................   1       1    S01
    .........................................................................ccuuauucgugcagacugugaagg................   1       0    S01
    ..........................................................................cuuauucgugcagacugu.....................   1       0    S01
    .............................................................................auucgugcagacugugaaggu...............   1       0    S01

    .....................................................................aaucccuuauucgugcagacugCg....................   5       1    S02
    .....................................................................aaucccuuauucgugcagacCgug....................   1       1    S02
    .....................................................................aaucccuuauucgugcagacugug....................   15      0    S02
    ......................................................................aucccuuauucgugcagacuguga...................   1       0    S02
    .......................................................................ucccuuauucgugcagacugug....................   1       0    S02
    ........................................................................cccuuauucgugcagacugugaag.................   1       0    S02
    .................................................................................gugcagacugugaaggug..............   1       0    S02
```

Provisional ID	:	Maple_Unigene_BMK.33498_155140
Score total	:	3.8
Score for star read(s)	:	3.9
Score for read counts	:	-4.4
Score for mfe	:	1.4
Score for randfold	:	0
Score for cons. seed	:	3
Total read count	:	3
Mature read count	:	2
Loop read count	:	0
Star read count	:	1

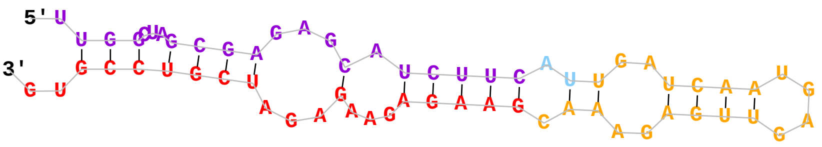

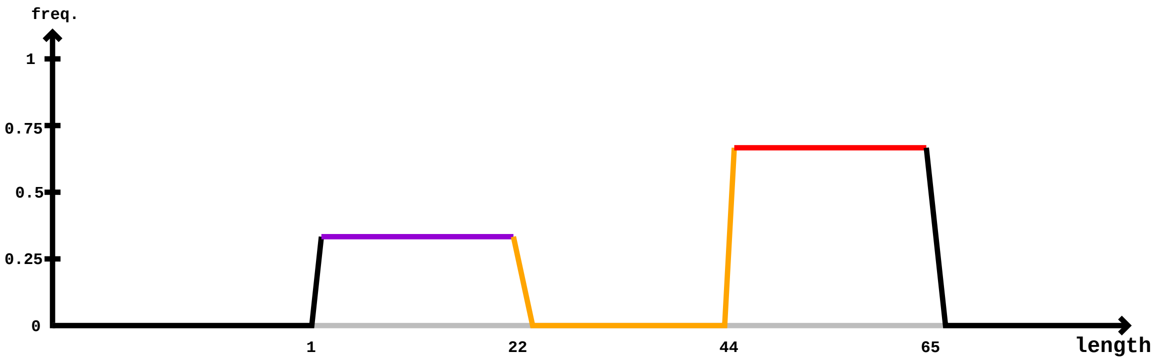

Star **Mature**

```
5'- ccagcugauggcauuaaauggcugugauuggcuagcgagagcaucuucauugaucaaugaguugagaaacgaagagaagagaucguccgugguacagcaguugaugucaac  -3'   obs
    ccagcugauggcauuaaauggcugugauuggcuagcgagagcaucuucauugaucaaugaguugagaaacgaagagaagagaucguccgugguacagcaguugaugucaac         exp
    ........((((((((((.((((((..(((...((((...(.(((((.((..((((....))))..)).)))))...)...)))))))...)))))).))))))))))..    reads       mm        sample
    ...........................uuggcuagcgagagcaucuuc...............................................................    1           0         S01
    ......................................................................gaagagaagagaucguccguA....................    1           1         S01

    ......................................................................gaagagaagagaucguccgug....................    1           0         S02
```

Provisional ID	:	Maple_Unigene_BMK.33522_155330
Score total	:	7763.3
Score for star read(s)	:	3.9
Score for read counts	:	7754.6
Score for mfe	:	1.8
Score for randfold	:	0
Score for cons. seed	:	3
Total read count	:	15222
Mature read count	:	14844
Loop read count	:	0
Star read count	:	378

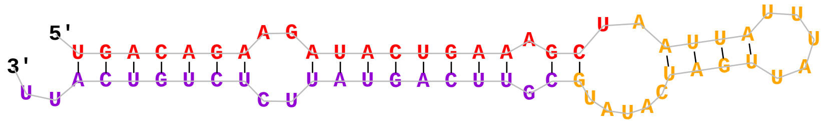

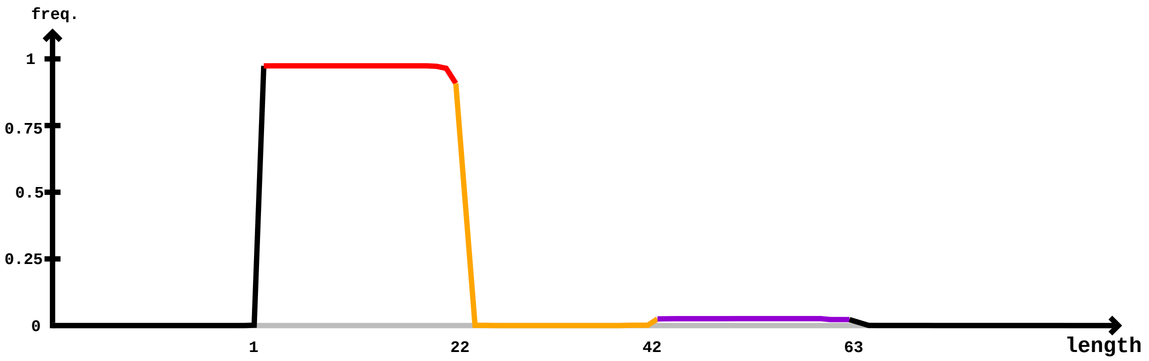

Mature **Star**

```
5'- aaaugucuagauuuuguuaaugacagaagauacugaaagcuaauuauuuauugaucauaugcguucaguauucucugucauuaaugaauucuaagacaaauaucguuga  -3'   obs
    aaaugucuagauuuuguuaaugacagaagauacugaaagcuaauuauuuauugaucauaugcguucaguauucucugucauuaaugaauucuaagacaaauaucguuga        exp
    ...((((((((.((..(((((((((((..((((((((.((..((((.....)))).....)).))))))))..)))))))))))..)).))).)))))...........   reads   mm   sample
    ...................augacagaagauacugaaagc.....................................................................   3       0    S01
    ...................Cugacagaagauacugaaagcu....................................................................   2       1    S01
    ...................augacagaagauacugaaagcu....................................................................   2       0    S01
    ....................ugacagaagauacugaaa.......................................................................   12      0    S01
    ....................ugacagaagauacugaaUg......................................................................   1       1    S01
    ....................ugacagaagauacugaaGg......................................................................   1       1    S01
    ....................ugacagaagauacugaaag......................................................................   39      0    S01
    ....................ugacagaagauacugaaaA......................................................................   1       1    S01
    ....................Agacagaagauacugaaag......................................................................   1       1    S01
    ....................ugacagaagauacugaaaU......................................................................   13      1    S01
    ....................ugacagaagauacugaaaC......................................................................   2       1    S01
    ....................ugacagaagauacugGaag......................................................................   1       1    S01
    ....................ugacagaagauacugaaagA.....................................................................   15      1    S01
    ....................uAacagaagauacugaaagc.....................................................................   1       1    S01
    ....................ugacagaagauaUugaaagc.....................................................................   1       1    S01
    ....................ugacagaagauaGugaaagc.....................................................................   1       1    S01
    ....................ugacagaagauacugaaaAc.....................................................................   2       1    S01
    ....................ugacagaagauacugaaagc.....................................................................   359     0    S01
    ....................ugacagaagauacugaaagU.....................................................................   94      1    S01
    ....................ugacagaagCuacugaaagc.....................................................................   1       1    S01
    ....................Agacagaagauacugaaagc.....................................................................   1       1    S01
    ....................ugacagaagauacugaaaUc.....................................................................   5       1    S01
    ....................ugacagaagauacugaaGgc.....................................................................   1       1    S01
    ....................ugacagaagauacugCaagc.....................................................................   3       1    S01
    ....................ugacagaagauacugaaagG.....................................................................   1       1    S01
    ....................ugacagGagauacugaaagc.....................................................................   1       1    S01
    ....................ugacagaagauacugaGagc.....................................................................   2       1    S01
    ....................Cgacagaagauacugaaagc.....................................................................   1       1    S01
    ....................ugacGgaagauacugaaagc.....................................................................   2       1    S01
    ....................ugacagaagGuacugaaagc.....................................................................   3       1    S01
    ....................uCacagaagauacugaaagc.....................................................................   1       1    S01
    ....................ugacUgaagauacugaaagc.....................................................................   1       1    S01
    ....................ugacaAaagauacugaaagc.....................................................................   1       1    S01
```

```
                    Mature                                      Star
aaaugucuagauuuuguuaaugacagaagauacugaaagcuaauuauuuauugaucauaugcguucaguauucucugucauuaaugaauucuaagacaaauaucguuga
....................ugacagaagUuacugaaagc.....................................................................   1     1   S01
....................ugacagaagauacugGaagc.....................................................................   1     1   S01
....................ugacagaagaGacugaaagcu....................................................................   6     1   S01
....................ugacagaagauUcugaaagcu....................................................................   2     1   S01
....................ugacagaagauacugaaaAcu....................................................................   12    1   S01
....................Agacagaagauacugaaagcu....................................................................   9     1   S01
....................ugacagaagauaUugaaagcu....................................................................   4     1   S01
....................ugacagaagauCcugaaagcu....................................................................   2     1   S01
....................ugacagaCgauacugaaagcu....................................................................   5     1   S01
....................ugaAagaagauacugaaagcu....................................................................   11    1   S01
....................ugCcagaagauacugaaagcu....................................................................   3     1   S01
....................ugacagaaCauacugaaagcu....................................................................   1     1   S01
....................ugacagaaAauacugaaagcu....................................................................   3     1   S01
....................ugacagaagauacGgaaagcu....................................................................   4     1   S01
....................ugacagaagauaAugaaagcu....................................................................   6     1   S01
....................uAacagaagauacugaaagcu....................................................................   12    1   S01
....................ugacGgaagauacugaaagcu....................................................................   26    1   S01
....................ugacCgaagauacugaaagcu....................................................................   1     1   S01
....................ugaGagaagauacugaaagcu....................................................................   22    1   S01
....................Cgacagaagauacugaaagcu....................................................................   19    1   S01
....................ugacagaagaCacugaaagcu....................................................................   15    1   S01
....................ugacagaagauacuAaaagcu....................................................................   11    1   S01
....................ugacagaagauacugaaagUu....................................................................   49    1   S01
....................ugaUagaagauacugaaagcu....................................................................   6     1   S01
....................ugacagaagCuacugaaagcu....................................................................   2     1   S01
....................ugacagaGgauacugaaagcu....................................................................   28    1   S01
....................ugacagaagauacugaaGgcu....................................................................   34    1   S01
....................ugacagaagauacugaUagcu....................................................................   1     1   S01
....................ugacagaagaAacugaaagcu....................................................................   3     1   S01
....................ugacagaagGuacugaaagcu....................................................................   29    1   S01
....................ugacUgaagauacugaaagcu....................................................................   5     1   S01
....................ugacagaUgauacugaaagcu....................................................................   2     1   S01
....................ugacagaagauacAgaaagcu....................................................................   7     1   S01
....................ugacaCaagauacugaaagcu....................................................................   5     1   S01
....................ugacagaagauacugaaagcG....................................................................   198   1   S01
....................ugGcagaagauacugaaagcu....................................................................   35    1   S01
....................ugacaUaagauacugaaagcu....................................................................   2     1   S01
....................ugacagaagauacugUaagcu....................................................................   3     1   S01
....................ugacagaagauacugaCagcu....................................................................   1     1   S01
....................Ngacagaagauacugaaagcu....................................................................   5     1   S01
....................ugacagaagauaGugaaagcu....................................................................   3     1   S01
....................ugacagaagauacugaaCgcu....................................................................   1     1   S01
....................ugacagaagauacugaaUgcu....................................................................   2     1   S01
....................uCacagaagauacugaaagcu....................................................................   4     1   S01
....................ugacagaagauacugaaaUcu....................................................................   3     1   S01
....................uUacagaagauacugaaagcu....................................................................   5     1   S01
....................ugacagaagauacugGaagcu....................................................................   32    1   S01
....................ugacagGagauacugaaagcu....................................................................   13    1   S01
....................ugacagaagauacugaaagGu....................................................................   1     1   S01
....................ugacagaagauacugaGagcu....................................................................   19    1   S01
....................ugacagaaUauacugaaagcu....................................................................   2     1   S01
....................ugacagaagauacuCaaagcu....................................................................   2     1   S01
....................ugacagaagauacugaaaCcu....................................................................   5     1   S01
....................ugacagaagUuacugaaagcu....................................................................   5     1   S01
....................ugacagaagauacugaaagAu....................................................................   3     1   S01
....................ugacagaagauGcugaaagcu....................................................................   15    1   S01
....................Ggacagaagauacugaaagcu....................................................................   8     1   S01
....................ugacagUagauacugaaagcu....................................................................   2     1   S01
....................ugacagaagauacugCaagcu....................................................................   10    1   S01
....................ugacagaagauacCgaaagcu....................................................................   17    1   S01
....................ugacagaagauacugaaagcA....................................................................   272   1   S01
....................ugacaAaagauacugaaagcu....................................................................   10    1   S01
....................ugacagaagauacugaaagcC....................................................................   818   1   S01
....................ugUcagaagauacugaaagcu....................................................................   4     1   S01
....................ugacagaagauacugaaagcu....................................................................   6281  0   S01
....................ugacagaagauacugaaagcCa...................................................................   1     1   S01
....................ugacagaagauacugaaagcuU...................................................................   108   1   S01
....................ugGcagaagauacugaaagcua...................................................................   1     1   S01
....................ugacagaagauacugaaagcua...................................................................   8     0   S01
....................ugacagaagGuacugaaagcua...................................................................   1     1   S01
```

```
                    Mature                                  Star
aaaugucuagauuuuguuaaugacagaagauacugaaagcuaauuauuuauugaucauaugcguucaguauucucugucauuaaugaauucuaagacaaauaucguuga
....................ugacagaagauacugaaagcuG...................................................................	4	1	S01
....................ugacagaagauacugaaagcuC...................................................................	14	1	S01
....................ugacagaagauacugaaagUua...................................................................	3	1	S01
....................ugacagaagauacCgaaagcua...................................................................	1	1	S01
....................ugacagaagauacugaaagcuaC..................................................................	1	1	S01
....................ugacagaagauacugaaagcuaau.................................................................	1	0	S01
....................ugacagaagauacugaaagcuaUu.................................................................	2	1	S01
.....................gacagaagauacugaaagc.....................................................................	1	0	S01
..........................................................augcguucaguauucGcugucauu...........................	1	1	S01
..........................................................augcguucaguauucucugucauu...........................	5	0	S01
..........................................................augcAuucaguauucucugucauu...........................	1	1	S01
...........................................................ugcguucaguauucucugucauC...........................	1	1	S01
...........................................................ugcguucaguauucucugucauu...........................	1	0	S01
.............................................................cguucaguauucucuguc..............................	29	0	S01
.............................................................cguucaguauucucuguU..............................	2	1	S01
.............................................................cAuucaguauucucuguc..............................	6	1	S01
.............................................................cguucaguauucuUuguc..............................	1	1	S01
.............................................................cguucaguauuUucuguc..............................	1	1	S01
.............................................................cguucagCauucucuguc..............................	1	1	S01
.............................................................cguucaguauucuGugucauu...........................	1	1	S01
.............................................................cguucaguauucucugucauC...........................	9	1	S01
.............................................................cguucaguauGcucugucauu...........................	1	1	S01
.............................................................cguucagAauucucugucauu...........................	1	1	S01
.............................................................cguucaguauucucugucauu...........................	88	0	S01
.............................................................cguucaguaAucucugucauu...........................	1	1	S01
.............................................................cguuGaguauucucugucauu...........................	1	1	S01
.............................................................cguucaguauucucugucGuu...........................	1	1	S01
.............................................................cAuucaguauucucugucauu...........................	80	1	S01
.............................................................cguucaguauucucugucauA...........................	1	1	S01
.............................................................cguucaguauucucugucauuG..........................	1	1	S01
..............................................................Auucaguauucucugucauu...........................	4	1	S01
...............................................................uucaguauucucugucauu...........................	1	0	S01
...............................................................uucaguauucucugucauuC..........................	1	1	S01
.....................................................................aucucugucauuaaugGauu....................	1	1	S01
.....................................................................aucucugucauuaaugaauu....................	1	0	S01
.....................................................................aucucugucauuaaugaauucua.................	1	0	S01

..................aaugacagaagauacCgaaag......................................................................	1	1	S02
..................aCugacagaagauacugaaagcu....................................................................	1	1	S02
...................augacagaagauacugaaagc.....................................................................	5	0	S02
...................augacagaagauacugaaagU.....................................................................	1	1	S02
...................augacagaagauacugaaagcC....................................................................	1	1	S02
...................augacagaagauacugaaagcu....................................................................	2	0	S02
...................augacagaagauacuCaaagcu....................................................................	1	1	S02
...................augacCgaagauacugaaagcu....................................................................	1	1	S02
....................ugacagaagauacugaaU.......................................................................	3	1	S02
....................ugacaUaagauacugaaa.......................................................................	1	1	S02
....................ugacagaagauacugaaa.......................................................................	11	0	S02
....................ugacagaaAauacugaaag......................................................................	1	1	S02
....................ugacagaagauacugaGag......................................................................	1	1	S02
....................ugacagaGgauacugaaag......................................................................	1	1	S02
....................ugacagaagauacGgaaag......................................................................	1	1	S02
....................ugacagaagauacugaaag......................................................................	44	0	S02
....................ugacagaagauacugaaaA......................................................................	1	1	S02
....................ugacagaagauacugaaaU......................................................................	6	1	S02
....................ugacagaagauacugaaGgc.....................................................................	1	1	S02
....................ugacagaagauacugaaagG.....................................................................	2	1	S02
....................ugacagaagauacugGaagc.....................................................................	1	1	S02
....................ugacagaagauacugaGagc.....................................................................	1	1	S02
....................ugacagaagGuacugaaagc.....................................................................	2	1	S02
....................ugacagUagauacugaaagc.....................................................................	1	1	S02
....................ugacagaagauacuCaaagc.....................................................................	1	1	S02
....................ugacagGagauacugaaagc.....................................................................	2	1	S02
....................ugacagaagauacugaaaUc.....................................................................	4	1	S02
....................Ngacagaagauacugaaagc.....................................................................	1	1	S02
....................ugacagaagauacugaaagc.....................................................................	256	0	S02
....................ugaAagaagauacugaaagc.....................................................................	1	1	S02
....................ugacagaCgauacugaaagc.....................................................................	1	1	S02
....................ugacagaagauacugaaagA.....................................................................	7	1	S02
....................ugacagaagauacCgaaagc.....................................................................	1	1	S02
```

```
                    Mature                  Star
aaaugucuagauuuuguuaaugacagaagauacugaaagcuaauuauuuauugaucauaugcguucaguauucucugucauuaaugaauucuaagacaaauaucguuga
....................uAacagaagauacugaaagc.....................................................................  2     1   S02
....................ugacagaagauacugaaagU.....................................................................  65    1   S02
....................ugacGgaagauacugaaagc.....................................................................  3     1   S02
....................ugacagaagauacugUaagc.....................................................................  1     1   S02
....................ugacagaagauGcugaaagc.....................................................................  1     1   S02
....................ugacagaagauacCgaaagcu....................................................................  3     1   S02
....................ugacagaGgauacugaaagcu....................................................................  14    1   S02
....................ugacagaaCauacugaaagcu....................................................................  2     1   S02
....................ugacagaagauacAgaaagcu....................................................................  6     1   S02
....................ugacagaagauacuUaaagcu....................................................................  2     1   S02
....................ugacagaagauacugaaCgcu....................................................................  2     1   S02
....................ugaGagaagauacugaaagcu....................................................................  2     1   S02
....................ugacagaagauCcugaaagcu....................................................................  6     1   S02
....................ugacagaagauUcugaaagcu....................................................................  1     1   S02
....................ugacagaagCuacugaaagcu....................................................................  3     1   S02
....................ugacagGagauacugaaagcu....................................................................  17    1   S02
....................ugacagaagaGacugaaagcu....................................................................  5     1   S02
....................ugacagaagauacugaaagcC....................................................................  479   1   S02
....................ugacagaagauaUugaaagcu....................................................................  9     1   S02
....................ugacagCagauacugaaagcu....................................................................  3     1   S02
....................ugacagaagauacugaaaCcu....................................................................  1     1   S02
....................ugacagaaUauacugaaagcu....................................................................  1     1   S02
....................ugacagaagauacugaaaAcu....................................................................  9     1   S02
....................ugacagaagauacugaaagGu....................................................................  2     1   S02
....................Agacagaagauacugaaagcu....................................................................  6     1   S02
....................ugacCgaagauacugaaagcu....................................................................  3     1   S02
....................ugacagaagauacugaGagcu....................................................................  13    1   S02
....................ugacagaagauacugaCagcu....................................................................  2     1   S02
....................ugacUgaagauacugaaagcu....................................................................  3     1   S02
....................ugacagaagauacugCaagcu....................................................................  4     1   S02
....................uCacagaagauacugaaagcu....................................................................  1     1   S02
....................Ggacagaagauacugaaagcu....................................................................  4     1   S02
....................ugacagUagauacugaaagcu....................................................................  3     1   S02
....................ugacaCaagauacugaaagcu....................................................................  1     1   S02
....................ugacagaagauacugaaagUu....................................................................  43    1   S02
....................ugacagaaAauacugaaagcu....................................................................  3     1   S02
....................uUacagaagauacugaaagcu....................................................................  2     1   S02
....................Ngacagaagauacugaaagcu....................................................................  4     1   S02
....................ugacagaagauacugaaagAu....................................................................  4     1   S02
....................ugCcagaagauacugaaagcu....................................................................  3     1   S02
....................ugacagaagaCacugaaagcu....................................................................  5     1   S02
....................ugacagaagauacugaaaUcu....................................................................  1     1   S02
....................ugacagaUgauacugaaagcu....................................................................  4     1   S02
....................ugacaUaagauacugaaagcu....................................................................  1     1   S02
....................ugacagaagauacugUaagcu....................................................................  1     1   S02
....................ugacaAaagauacugaaagcu....................................................................  7     1   S02
....................ugacagaagauaGugaaagcu....................................................................  2     1   S02
....................ugacagaagauacuAaaagcu....................................................................  5     1   S02
....................ugacagaagauacugaaGgcu....................................................................  20    1   S02
....................ugacagaagauaAugaaagcu....................................................................  6     1   S02
....................ugacagaagUuacugaaagcu....................................................................  4     1   S02
....................ugaUagaagauacugaaagcu....................................................................  3     1   S02
....................ugacagaagauacugaaagcA....................................................................  134   1   S02
....................uAacagaagauacugaaagcu....................................................................  7     1   S02
....................ugacagaagauacugaaagcG....................................................................  481   1   S02
....................Cgacagaagauacugaaagcu....................................................................  7     1   S02
....................ugaAagaagauacugaaagcu....................................................................  3     1   S02
....................ugacagaagauGcugaaagcu....................................................................  11    1   S02
....................ugGcagaagauacugaaagcu....................................................................  18    1   S02
....................ugacagaagauacugGaagcu....................................................................  18    1   S02
....................ugacagaagauacGgaaagcu....................................................................  1     1   S02
....................ugacagaagGuacugaaagcu....................................................................  26    1   S02
....................ugacagaagauacugaaagcu....................................................................  3986  0   S02
....................ugUcagaagauacugaaagcu....................................................................  6     1   S02
....................ugacagaagauacugaaUgcu....................................................................  1     1   S02
....................ugacGgaagauacugaaagcu....................................................................  17    1   S02
....................ugacagaagauacugaaagUua...................................................................  2     1   S02
....................ugacagaagauacugaaagcuG...................................................................  6     1   S02
....................ugacagaagauacugaaagcua...................................................................  4     0   S02
....................ugacagaagauacugaaagcuU...................................................................  87    1   S02
```

Mature   Star

```
aaaugucuagauuuuguuaaugacagaagauacugaaagcuaauuauuuauugaucauaugcguucaguauucucugucauuaaugaauucuaagacaaauaucguuga
....................ugacagaagauacugaaagcuC..................................................................	9	1	S02
....................ugacagaagauacugaaagcuUa.................................................................	1	1	S02
....................ugacagaagauacugaaagcuaU.................................................................	1	1	S02
....................ugacagaagauacugaaagcuaaG................................................................	1	1	S02
....................ugacagaagauacugaaagcuaau................................................................	4	0	S02
....................ugacagaagauacugaaagcuaUuu...............................................................	1	1	S02
....................ugacagaagauacugaaagcuaauu...............................................................	1	0	S02
.........................................................augcAuucaguauucucuguc..............................	1	1	S02
.........................................................augcguucaguauucucugucau............................	1	0	S02
.........................................................augcguucaguauucucugucauu...........................	6	0	S02
.........................................................augcAuucaguauucucugucauu...........................	1	1	S02
............................................................cguucaguauucucuguc..............................	9	0	S02
............................................................cAuucaguauucucuguc..............................	2	1	S02
............................................................cguucaguauucucuguU..............................	2	1	S02
............................................................cguucaguauucuGugucauu...........................	1	1	S02
............................................................cguucGguauucucugucauu...........................	1	1	S02
............................................................cguucaguauucucugucauu...........................	70	0	S02
............................................................cguucaguauucucugucaGu...........................	1	1	S02
............................................................cguucaguauucucugucauC...........................	7	1	S02
............................................................cguGcaguauucucugucauu...........................	1	1	S02
............................................................cCuucaguauucucugucauu...........................	1	1	S02
............................................................cguucaguauucucugucauG...........................	2	1	S02
............................................................cguucagAauucucugucauu...........................	2	1	S02
............................................................cAuucaguauucucugucauu...........................	37	1	S02
.............................................................Auucaguauucucugucauu...........................	4	1	S02
.............................................................Auucaguauucucugucauua..........................	1	1	S02
..............................................................uucaguauucucugucauuaC.........................	1	1	S02
..............................................................uucaguauucucugucauuaa.........................	1	0	S02
..............................................................uucaguauucucugucauuaauU.......................	1	1	S02
...............................................................ucaguauucucugucauu...........................	1	0	S02
..................................................................................augaauucuaagacaaauauc.....	1	0	S02
```

Provisional ID	:	Maple_Unigene_BMK.33555_155616
Score total	:	155.1
Score for star read(s)	:	3.9
Score for read counts	:	150
Score for mfe	:	1.8
Score for randfold	:	0
Score for cons. seed	:	-0.6
Total read count	:	306
Mature read count	:	304
Loop read count	:	0
Star read count	:	2

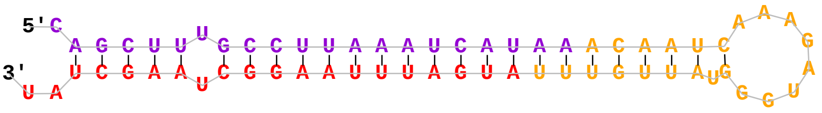

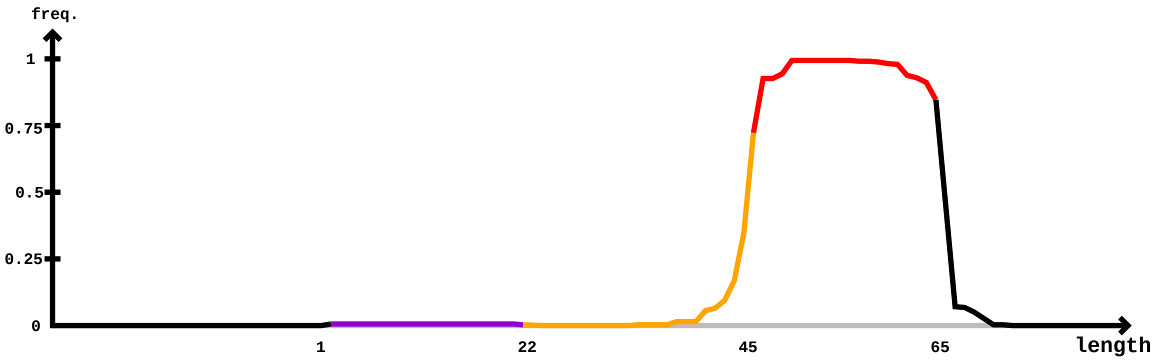

Star Mature

```
5'- uguuuaugguuucuagaguauuuacuacagcuuugccuuaaaucauaaacaaucaaagauggguauuguuuaugauuuaaggcuaagcuauaguaauuaaucaaagagaa  -3'   obs
    uguuuaugguuucuagaguauuuacuacagcuuugccuuaaaucauaaacaaucaaagauggguauuguuuaugauuuaaggcuaagcuauaguaauuaaucaaagagaa          exp
    .........(((((.((.((.((((((.(((((.((((((((((((((((((((........).))))))))))))))))))).))))).)))))).)).))..))))).    reads    mm    sample
    ...........................cagcuuAgccuuaaaucaua...............................................................    1        1     S01
    ...........................................................uggUuauuguuuaugauuuaagg............................    1        1     S01
    ...............................................................uauuguuuaugauuuaaggcuaag.......................    1        0     S01
    ..................................................................uguuuaugauuuaaggcua.........................    1        0     S01
    ..................................................................uguuuaugauuuaaggcuaag.......................    4        0     S01
    ..................................................................uguuuaugauuuaaggcuaaA.......................    1        1     S01
    ...................................................................guuuaugauuuaaggcuaagcuGu...................    1        1     S01
    ....................................................................Cuuaugauuuaaggcuaagcu.....................    1        1     S01
    ....................................................................uuuaugauuuaaggcuaagcu.....................    3        0     S01
    .....................................................................uuaugauuuaaggcuaagcuG....................    6        1     S01
    ......................................................................uaugauuuaaggcuaagcuGu...................    15       1     S01
    .......................................................................augauuuaaggcuaagcuGu...................    36       1     S01
    .......................................................................augauuuaaggcuaagcuGua..................    13       1     S01
    ........................................................................ugauuuaaggcuaagcuGu...................    26       1     S01
    ...........................................................................uuuaaggcuaagcuGuagua...............    2        1     S01
    ...........................................................................uuuaaggcuaagcuGuaguaa..............    3        1     S01

    ...........................cagcuuAgccuuaaaucauaa..............................................................    1        1     S02
    ...............................................................uauuguuuaugauuuaaggcua.........................    1        0     S02
    ...............................................................uauuguuuaugauuuaaggcuaag.......................    2        0     S02
    ..................................................................uguuuaugauuuaaggcu..........................    1        0     S02
    ..................................................................uguuuaugauuuaaggcuaa........................    1        0     S02
    ..................................................................uguuuaugauuuaaggcuaag.......................    5        0     S02
    ..................................................................uguuuaugUuuuaaggcuaag.......................    1        1     S02
    ...................................................................guuuaugauuuaaggcuaagcuGu...................    2        1     S02
    ....................................................................uuAaugauuuaaggcuaagc......................    1        1     S02
    ....................................................................uuAaugauuuaaggcuaagcu.....................    1        1     S02
    ....................................................................uuuaugauuuaaggcuaagcu.....................    1        0     S02
    ....................................................................uuuaugauuuaaggcuaagcuGu...................    2        1     S02
    ....................................................................uuuaugauuuaaggcuaagcuGua..................    1        1     S02
    .....................................................................uuaugauuuaaggcuaagc......................    2        0     S02
    .....................................................................uuaugauuuaaggcuaagcuC....................    1        1     S02
    .....................................................................uuaugauuuaaggcuaagcuU....................    1        1     S02
```

```
                           Star                                        Mature
uguuuaugguuucuagaguauuuacuacagcuuugccuuaaaucauaaacaaucaaagauggguauuguuuaugauuuaaggcuaagcuauaguaauuaaucaaagagaa
.....................................................................uuaugauuuaaggcuaagcuG....................    14    1    S02
.....................................................................uuaugauuuaaggcuaagcuUu...................    1     1    S02
......................................................................uaugauuuaaggcuaagcuCu...................    1     1    S02
......................................................................uaugauuuaaggcuaagcuGu...................    44    1    S02
......................................................................uaugauuuaaggcuaagcuau...................    1     0    S02
.......................................................................augauuuaaggcuaagcuGu...................    63    1    S02
.......................................................................augauuuaaggcuaagcuGua..................    15    1    S02
........................................................................ugauuuaaggcuaagcuGu...................    41    1    S02
........................................................................ugauuuaaggcuaagcuCua..................    1     1    S02
........................................................................ugauuuaaggcuaagcuGuag.................    1     1    S02
..........................................................................auuuaaggcuaagcuGuagu................    5     1    S02
..........................................................................auuuaaggcuaagcuGuagua...............    1     1    S02
...........................................................................uuuaaggcuaagcuGuagu................    1     1    S02
...........................................................................uuuaaggcuaagcuGuagua...............    5     1    S02
...........................................................................uuuaaggcuaagcuGuaguaa..............    5     1    S02
...........................................................................uuuaaggcuaagcuGuaguaauu............    1     1    S02
```

Provisional ID	:	Maple_Unigene_BMK.33719_157261
Score total	:	2.6
Score for star read(s)	:	3.9
Score for read counts	:	-4.9
Score for mfe	:	0.7
Score for randfold	:	0
Score for cons. seed	:	3
Total read count	:	2
Mature read count	:	1
Loop read count	:	0
Star read count	:	1

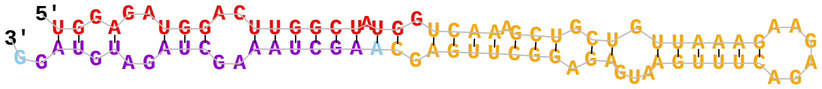

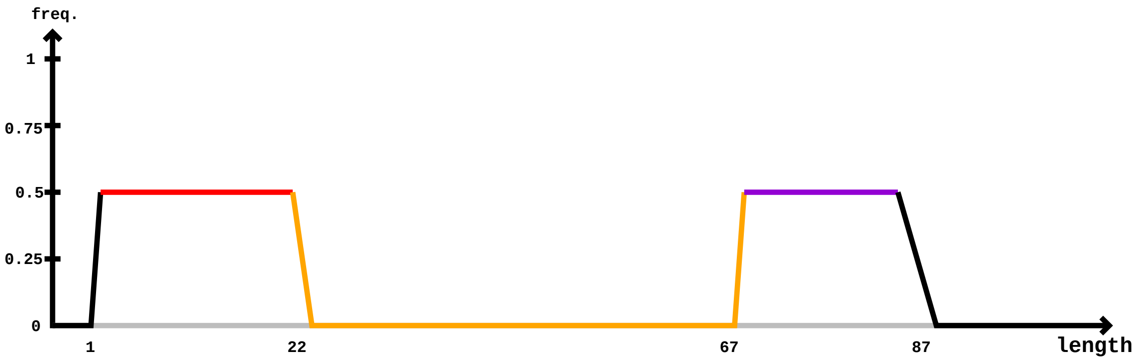

Mature   Star

```
5'- ugauggagauggacuuggcuauggucaaagcugcuguuaaagaagagacuuugaaugagaggcuugagcaagcuaaagcuagauguaggcaggcaauacuaguugcca   -3'   obs
    ugauggagauggacuuggcuauggucaaagcugcuguuaaagaagagacuuugaaugagaggcuugagcaagcuaaagcuagauguaggcaggcaauacuaguugcca         exp
    ...((.(..(((..((((((.((.((((.(((.((.((((((......))))))...)).))))))).))))))))..)))..).))....((((((....)))))).   reads   mm   sample
    ...uggagauggacuuggcuaugg....................................................................................   1       0    S02
    ......................................................................agcuaaagcuagauguag....................   1       0    S02
```

Provisional ID : Maple_Unigene_BMK.34013_160302
Score total : 5256.9
Score for star read(s) : 3.9
Score for read counts : 5247.8
Score for mfe : 2.3
Score for randfold : 0
Score for cons. seed : 3
Total read count : 10305
Mature read count : 5434
Loop read count : 1
Star read count : 4870

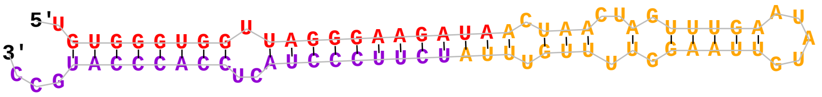

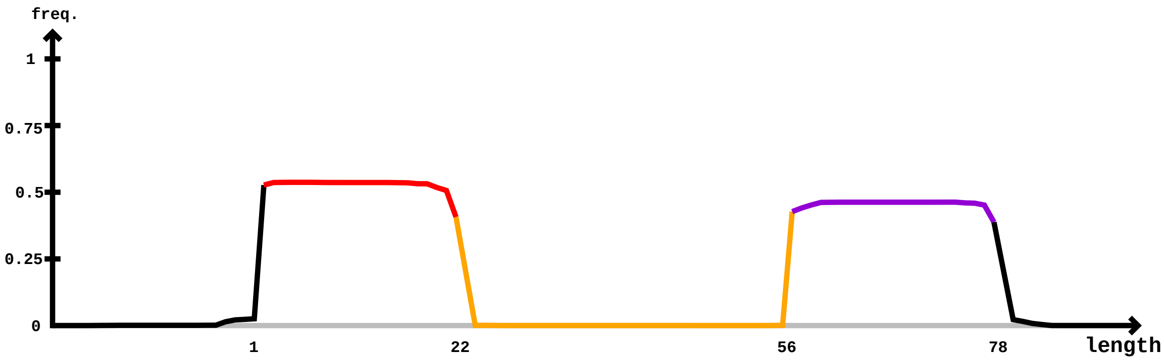

Mature Star

```
5'- ggauuuugggaagguuuuggugugggugguuagggaagauaacuaacuaguuugaauauguuaagguuuuguuuaucuucccuacuccacccaugccauaguuucccauc  -3'  obs
    ggauuuugggaagguuuuggugugggugguuagggaagauaacuaacuaguuugaauauguuaagguuuuguuuaucuucccuacuccacccaugccauaguuucccauc       exp
    ......((((((..((.((((((((((((.((((((((((((.(((..(.(((((.....))))).).))).))))))))))))..)))))))))))).))..))))))..  reads  mm  sample
    ..auuuugggaaAguuuuggugug......................................................................................  1  1  S01
    ...uuuugggaagguuuuggugugg.....................................................................................  1  0  S01
    ...uuuugggaagguuuuggugugggu...................................................................................  1  0  S01
    ....uuugggaagguuuugguguggA....................................................................................  1  1  S01
    ....uuugggaagguuuuggugugg.....................................................................................  1  0  S01
    .....uugggaagguuuuggugugg.....................................................................................  1  0  S01
    .....uugggaagguuuuggugugggu...................................................................................  1  0  S01
    ..............uuuuggugugggugguuagg............................................................................  1  0  S01
    ..............uuuuggugugggugguuagggaa.........................................................................  1  0  S01
    ..............uuuuggugugggugguuagggaag........................................................................  1  0  S01
    ................uuggugugggugguuagg............................................................................  3  0  S01
    ................uuggugugggugguuaggg...........................................................................  2  0  S01
    ................uuggugugggugguuaggga..........................................................................  5  0  S01
    ................uuggugugggCgguuaggga..........................................................................  1  1  S01
    ................uuggugugggugguuagggaag........................................................................  13  0  S01
    ................uuggGgugggugguuagggaag........................................................................  1  1  S01
    ................uuggugugAgugguuagggaaga.......................................................................  1  1  S01
    ................uuggugugggugguuagggaaga.......................................................................  34  0  S01
    ................uuggugugggugguuagggaagG.......................................................................  1  1  S01
    ................uuUgugugggugguuagggaaga.......................................................................  1  1  S01
    ................uuggugugggugguuaggCaaga.......................................................................  1  1  S01
    ................uuggugugggugguuagggaagU.......................................................................  1  1  S01
    ................uugguguggguggAuagggaaga.......................................................................  1  1  S01
    ................uuggugugggugguuagggaagaC......................................................................  3  1  S01
    ................uugguguggAugguuagggaagau......................................................................  1  1  S01
    ................uuggGgugggugguuagggaagau......................................................................  1  1  S01
    ................uuggugugggugguuagggaagau......................................................................  15  0  S01
    ................uCggugugggugguuagggaagau......................................................................  1  1  S01
    ................uuggugugggugguuagggaagauU.....................................................................  7  1  S01
    ................uuggugugggugguuagggaagaua.....................................................................  3  0  S01
    .................uggugugggugguuaggga..........................................................................  4  0  S01
    .................uggugugggugguuagggaaA........................................................................  1  1  S01
    .................uggugugggugguuagggaag........................................................................  13  0  S01
```

```
                    Mature                                          Star
ggauuuugggaagguuuuggugugggugguuagggaagauaacuaacuaguuugaauauguuaaagguuuuguuuaucuucccuacuccacccaugccauaguuuucccauc
.................uggugugggugguuagggaaga.........................................................................    4     0    S01
.................uggugugggugguuagggaagau........................................................................    1     0    S01
..................ggGgugggugguuaggga............................................................................    1     1    S01
..................ggugugggugguuaggga............................................................................    4     0    S01
..................ggugugggugguuagggaag..........................................................................    1     0    S01
..................ggugugggugguuagggaaga.........................................................................    1     0    S01
..................ggugugggugguuagggaagau........................................................................    1     0    S01
..................ggugugggugguuagggaagaua.......................................................................    2     0    S01
..................ggugugggugguuagggaagauU......................................................................    1     1    S01
...................gugugggugguuagAgaag..........................................................................    1     1    S01
...................gugugggugguuagggaaga.........................................................................    1     0    S01
...................gugugggugguuagggaagaC........................................................................    1     1    S01
...................gugugggugguuagggaagau........................................................................    6     0    S01
...................Cugugggugguuagggaagaua.......................................................................    1     1    S01
....................ugugggugguuagggaGg..........................................................................    1     1    S01
....................ugugggugguuaggUaag..........................................................................    1     1    S01
....................ugugggugguuagggaag..........................................................................    36    0    S01
....................ugugggugguuagggaaA..........................................................................    2     1    S01
....................ugugggugguuagggaaU..........................................................................    1     1    S01
....................uguUggugguuagggaag..........................................................................    1     1    S01
....................uguggguAguuagggaag..........................................................................    1     1    S01
....................ugugAgugguuagggaag..........................................................................    1     1    S01
....................ugugggugguuagggaagC.........................................................................    1     1    S01
....................ugugggugguuagggaaga.........................................................................    19    0    S01
....................ugugggGgguuagggaaga.........................................................................    1     1    S01
....................ugugggugguuagggaagU.........................................................................    1     1    S01
....................ugugggAgguuagggaaga.........................................................................    1     1    S01
....................uguAggugguuagggaaga.........................................................................    1     1    S01
....................ugugggugguuGgggaaga.........................................................................    1     1    S01
....................ugugggugguuCgggaagau........................................................................    1     1    S01
....................ugCgggugguuagggaagau........................................................................    1     1    S01
....................ugugggGgguuagggaagau........................................................................    2     1    S01
....................uguggguggucagggaagau........................................................................    2     1    S01
....................ugugggugguGagggaagau........................................................................    1     1    S01
....................ugugggugguuUgggaagau........................................................................    2     1    S01
....................ugugggugguuagggaaUau........................................................................    1     1    S01
....................ugugggugguuagggaagGu........................................................................    2     1    S01
....................uguggAugguuagggaagau........................................................................    1     1    S01
....................ugugggugguuagggaagaA........................................................................    2     1    S01
....................ugugggugg Cuagggaagau.......................................................................    3     1    S01
....................ugGgggugguuagggaagau........................................................................    2     1    S01
....................ugugggugg Auagggaagau.......................................................................    2     1    S01
....................ugugggugguuagggaagau........................................................................    480   0    S01
....................ugugggugguuagggaagaC........................................................................    42    1    S01
....................ugugggugguuagggaagaG........................................................................    5     1    S01
....................uguUggugguuagggaagau........................................................................    1     1    S01
....................ugugggAgguuagggaagau........................................................................    2     1    S01
....................ugugggCgguuagggaagau........................................................................    1     1    S01
....................ugugggugguuagggGagau........................................................................    2     1    S01
....................ugugggugg Guagggaagau.......................................................................    3     1    S01
....................ugugggugguuagggaagaua.......................................................................    1895  0    S01
....................ugugggugguuagggaaAaua.......................................................................    1     1    S01
....................ugugggugguuagggaCgaua.......................................................................    2     1    S01
....................ugugggCgguuagggaagaua.......................................................................    13    1    S01
....................ugugggugguuagggaagauG.......................................................................    15    1    S01
....................uguggAugguuagggaagaua.......................................................................    1     1    S01
....................ugugggugguCagggaagaua.......................................................................    2     1    S01
....................ugugggugguuagggaagaCa.......................................................................    2     1    S01
....................uguggguUguuagggaagaua.......................................................................    1     1    S01
....................ugugggGgguuagggaagaua.......................................................................    15    1    S01
....................ugugggugg Auagggaagaua......................................................................    4     1    S01
....................ugugggugguuaggAaagaua.......................................................................    3     1    S01
....................ugugggugguAagggaagaua.......................................................................    1     1    S01
....................ugugggAgguuagggaagaua.......................................................................    15    1    S01
....................ugugggugg Cuagggaagaua......................................................................    2     1    S01
....................ugugggugguuagggGagaua.......................................................................    7     1    S01
....................ugugggugAuuagggaagaua.......................................................................    2     1    S01
....................uguCggugguuagggaagaua.......................................................................    1     1    S01
....................Cgugggugguuagggaagaua.......................................................................    4     1    S01
....................ugCgggugguuagggaagaua.......................................................................    3     1    S01
```

Mature   Star

```
ggauuuugggaagguuuuggugugggugguuagggaagauaacuaacuaguuugaauauguuaagguuuuguuuaucuucccuacuccacccaugccauaguuucccauc
....................ugugggugguuaCggaagaua.....................................................................  1    1  S01
....................ugugggugguuagggaGgaua.....................................................................  9    1  S01
....................ugugggugguuaAggaagaua.....................................................................  2    1  S01
....................uguggguAguuagggaagaua.....................................................................  1    1  S01
....................ugugggugguuagggaagauC.....................................................................  73   1  S01
....................ugugggugguuUgggaagaua.....................................................................  1    1  S01
....................ugugggugUuuagggaagaua.....................................................................  1    1  S01
....................uUugggugguuagggaagaua.....................................................................  2    1  S01
....................ugAgggugguuagggaagaua.....................................................................  5    1  S01
....................ugugggugguuGgggaagaua.....................................................................  6    1  S01
....................ugugggugguuCgggaagaua.....................................................................  2    1  S01
....................ugugggugguuaggUaagaua.....................................................................  3    1  S01
....................Ggugggugguuagggaagaua.....................................................................  1    1  S01
....................ugugggugguuaggCaagaua.....................................................................  2    1  S01
....................ugugggugguuagggaagUua.....................................................................  2    1  S01
....................ugugggugguuagggaagaAa.....................................................................  1    1  S01
....................uguUggugguuagggaagaua.....................................................................  9    1  S01
....................ugugggugguuagggaagGua.....................................................................  10   1  S01
....................Agugggugguuagggaagaua.....................................................................  2    1  S01
....................uguAggugguuagggaagaua.....................................................................  4    1  S01
....................ugugggugguuagggaagauU.....................................................................  125  1  S01
....................uguggCugguuagggaagaua.....................................................................  1    1  S01
....................Ngugggugguuagggaagaua.....................................................................  2    1  S01
....................ugugAgugguuagggaagaua.....................................................................  2    1  S01
....................uAugggugguuagggaagaua.....................................................................  1    1  S01
....................uguggguggGuagggaagaua.....................................................................  8    1  S01
....................ugugggugguuagggCagaua.....................................................................  2    1  S01
....................ugugggugguuagggaagauaG....................................................................  2    1  S01
....................ugugggugguuagggaagauaa....................................................................  1    0  S01
....................ugugggugguuagggaagauaC....................................................................  2    1  S01
....................ugugggugguuagggaagauaU....................................................................  2    1  S01
....................ugugggugguuagggaagauaGcu..................................................................  1    1  S01
....................ugugggugguuagggaagauaacua.................................................................  1    0  S01
....................ugugggugguuagggaagauaCcua.................................................................  1    1  S01
.....................gugggugguuagggaCgau......................................................................  1    1  S01
.....................gugggugguuagggaagGu......................................................................  1    1  S01
.....................gugggugguuagggaagau......................................................................  8    0  S01
.....................gugggugguuaAggaagau......................................................................  1    1  S01
.....................Augggugguuagggaagau......................................................................  1    1  S01
.....................gugggugguuCgggaagau......................................................................  1    1  S01
.....................gugggugguuagggaagauC.....................................................................  1    1  S01
.....................gugggugAuuagggaagaua.....................................................................  1    1  S01
.....................gugggGgguuagggaagaua.....................................................................  1    1  S01
.....................gugggugguuagggaagCua.....................................................................  1    1  S01
.....................gugAgugguuagggaagaua.....................................................................  2    1  S01
.....................gugggugguuagggaagauU.....................................................................  7    1  S01
.....................gugggugguuagggaagaua.....................................................................  31   0  S01
......................ugggugguuagggaagau......................................................................  1    0  S01
......................ugggugguuGgggaagau......................................................................  1    1  S01
......................ugggugguuagggaagaua.....................................................................  1    0  S01
.......................gggugguuagggaagaua.....................................................................  3    0  S01
..........................................................................aucuucccuacuccacccaugc..............  2    0  S01
..........................................................................aucuucccuacuccacccaugA..............  1    1  S01
...........................................................................ucuucccuacuccacccU.................  1    1  S01
...........................................................................ucuucccuacuccaccca.................  13   0  S01
...........................................................................ucuucccuacuccacccC.................  1    1  S01
...........................................................................ucuucccuacuccacccau................  7    0  S01
...........................................................................ucuucccuacuccacccUu................  1    1  S01
...........................................................................ucuucccuacuccacccaug...............  33   0  S01
...........................................................................ucuGcccuacuccacccaug...............  1    1  S01
...........................................................................ucuucccuacuccacccaAg...............  1    1  S01
...........................................................................ucuucccuacuccacccauA...............  2    1  S01
...........................................................................ucuucccuacuccacGcaugc..............  1    1  S01
...........................................................................ucuucccuacuccacccaugU..............  54   1  S01
...........................................................................ucuucccAacuccacccaugc..............  1    1  S01
...........................................................................ucCucccuacuccacccaugc..............  1    1  S01
...........................................................................Acuucccuacuccacccaugc..............  1    1  S01
...........................................................................ucuucccuacuccacccaGgc..............  1    1  S01
...........................................................................ucuucccuacCccacccaugc..............  3    1  S01
...........................................................................Ccuucccuacuccacccaugc..............  1    1  S01
```

```
                    Mature                                                     Star
ggauuuugggaagguuuuggugugggugguuagggaagauaacuaacuaguuugaauauguuaagguuuuguuuaucuucccuacuccacccaugccauaguuuucccauc
...........................................................................ucuucUcuacuccacccaugc...............   1      1     S01
...........................................................................ucuucccuacuccacccaugA...............   19     1     S01
...........................................................................ucuucccuaAuccacccaugc...............   1      1     S01
...........................................................................ucuucccuacuccacccauAc...............   1      1     S01
...........................................................................ucuucccuacuccaccUaugc...............   1      1     S01
...........................................................................ucuucccuaGuccacccaugc...............   1      1     S01
...........................................................................ucuucccCacuccacccaugc...............   1      1     S01
...........................................................................ucuucccuacuccaccGaugc...............   1      1     S01
...........................................................................ucuucccuacuccaGccaugc...............   1      1     S01
...........................................................................ucuAcccuacuccacccaugc...............   1      1     S01
...........................................................................ucuucccuacuccacAcaugc...............   2      1     S01
...........................................................................ucuGcccuacuccacccaugc...............   1      1     S01
...........................................................................uGuucccuacuccacccaugc...............   1      1     S01
...........................................................................ucuucccuacuccacccaugc...............   323    0     S01
...........................................................................ucuucccuacuGcacccaugc...............   1      1     S01
...........................................................................ucuuccAuacuccacccaugc...............   1      1     S01
...........................................................................ucuuAccuacuccacccaugc...............   1      1     S01
...........................................................................ucuucccuacuccacccauUc...............   1      1     S01
...........................................................................ucuCcccuacuccacccaugc...............   1      1     S01
...........................................................................ucuucccuacuccGcccaugc...............   2      1     S01
...........................................................................ucuucccuacuUcacccaugc...............   1      1     S01
...........................................................................uUuucccuacuccacccaugc...............   1      1     S01
...........................................................................ucGucccuacuccacccaugc...............   1      1     S01
...........................................................................ucuucccuacuccCcccaugc...............   2      1     S01
...........................................................................Gcuucccuacuccacccaugcc..............   1      1     S01
...........................................................................Ncuucccuacuccacccaugcc..............   2      1     S01
...........................................................................ucuCcccuacuccacccaugcc..............   4      1     S01
...........................................................................ucuucccuacucUacccaugcc..............   2      1     S01
...........................................................................ucCucccuacuccacccaugcc..............   5      1     S01
...........................................................................ucuucccuacuGcacccaugcc..............   1      1     S01
...........................................................................ucuucccuaAuccacccaugcc..............   3      1     S01
...........................................................................ucuucccuacuccacccauUcc..............   2      1     S01
...........................................................................ucuucAcuacuccacccaugcc..............   2      1     S01
...........................................................................ucuucccuacuccacccauAcc..............   3      1     S01
...........................................................................ucuucccuacuccacccaugUc..............   5      1     S01
...........................................................................Acuucccuacuccacccaugcc..............   1      1     S01
...........................................................................ucuucccuacuAcacccaugcc..............   1      1     S01
...........................................................................ucuucccuacuccCcccaugcc..............   5      1     S01
...........................................................................ucuAcccuacuccacccaugcc..............   3      1     S01
...........................................................................ucuucccuacuccacccaugcU..............   446    1     S01
...........................................................................ucuucccuacuccUcccaugcc..............   1      1     S01
...........................................................................ucuuUccuacuccacccaugcc..............   1      1     S01
...........................................................................ucuucccuacuccacccaugcA..............   123    1     S01
...........................................................................ucuucccuacuUcacccaugcc..............   7      1     S01
...........................................................................uAuucccuacuccacccaugcc..............   1      1     S01
...........................................................................ucuucccuGcuccacccaugcc..............   4      1     S01
...........................................................................ucuucccuacuccaccAaugcc..............   3      1     S01
...........................................................................ucuucccuacuccaUccaugcc..............   3      1     S01
...........................................................................Ccuucccuacuccacccaugcc..............   3      1     S01
...........................................................................ucuucccuacuccacccUugcc..............   2      1     S01
...........................................................................ucuuccGuacuccacccaugcc..............   1      1     S01
...........................................................................ucuucccuaGuccacccaugcc..............   3      1     S01
...........................................................................ucuucccuacAccacccaugcc..............   1      1     S01
...........................................................................ucuucccuacuccacccaugcc..............   1417   0     S01
...........................................................................ucuucccuacuccacccGugcc..............   1      1     S01
...........................................................................ucuucccuacuccacccaugAc..............   6      1     S01
...........................................................................ucuucccuacuccGcccaugcc..............   2      1     S01
...........................................................................ucuucccuacuccacAcaugcc..............   2      1     S01
...........................................................................ucuucccuacuccacccaCgcc..............   2      1     S01
...........................................................................uUuucccuacuccacccaugcc..............   2      1     S01
...........................................................................ucuucccuacuccaccGaugcc..............   1      1     S01
...........................................................................ucuGcccuacuccacccaugcc..............   3      1     S01
...........................................................................ucuucccuacuccaAccaugcc..............   1      1     S01
...........................................................................ucuucccuacuccaccUaugcc..............   1      1     S01
...........................................................................ucAucccuacuccacccaugcc..............   1      1     S01
...........................................................................ucuucccuaUuccacccaugcc..............   4      1     S01
...........................................................................ucuuccUuacuccacccaugcc..............   1      1     S01
...........................................................................ucuucccGacuccacccaugcc..............   2      1     S01
...........................................................................ucuucGcuacuccacccaugcc..............   1      1     S01
...........................................................................ucuuAccuacuccacccaugcc..............   3      1     S01
```

```
                    Mature                                                 Star
ggauuuugggaagguuuuggugugggugguuagggaagauaacuaacuaguuugaauauguuaagguuuuguuuaucuucccuacuccacccaugccauaguuuucccauc
...........................................................................ucuucccuacuccacccaugcG..............  12  1  S01
...........................................................................ucuuccAuacuccacccaugcc..............  2   1  S01
...........................................................................ucuucccAacuccacccaugcc..............  2   1  S01
...........................................................................ucuucccuCcuccacccaugcc..............  1   1  S01
...........................................................................ucuucccuacuccacccaAgcc..............  5   1  S01
...........................................................................ucuucccuacuccacccaGgcc..............  2   1  S01
...........................................................................ucuucccuacucAacccaugcc..............  1   1  S01
...........................................................................ucuucccuacuccacUcaugcc..............  2   1  S01
...........................................................................ucuucccuacCccacccaugcc..............  6   1  S01
...........................................................................ucuucccuacuccacccaugGc..............  1   1  S01
...........................................................................ucuucccCacuccacccaugcc..............  2   1  S01
...........................................................................ucuucccuacuccacccaugcca.............  2   0  S01
...........................................................................ucuucccuacuccacAcaugcca.............  1   1  S01
...........................................................................ucuucccuacuccacccaugccU.............  5   1  S01
...........................................................................ucuucccuacuccacccaugccC.............  2   1  S01
...........................................................................ucuucccuacuccacccaugccG.............  1   1  S01
...........................................................................ucuucccuacuccacccaugccau............  2   0  S01
...........................................................................ucuucccuacuccacccaugccUu............  2   1  S01
...........................................................................ucuucccuacCccacccaugccau............  1   1  S01
...........................................................................ucuuccGuacuccacccaugccaua...........  1   1  S01
...........................................................................ucuucccuacuccacccaugccauC...........  1   1  S01
............................................................................cuucccuacuccacccau.................  1   0  S01
............................................................................cuucccuacuccacccaug................  2   0  S01
............................................................................cuucccuacuccacccauU................  1   1  S01
............................................................................cuucccuacuccacccauA................  1   1  S01
............................................................................cuucccuacuccacccaugc...............  4   0  S01
............................................................................cuucccuacuccacccaugU...............  1   1  S01
............................................................................cuucccuacuccacccaugG...............  1   1  S01
............................................................................cuucccuacuccacccaugcc..............  16  0  S01
............................................................................cuucccuacuccacccaugcA..............  2   1  S01
............................................................................cuucccuacuccacccaugcU..............  7   1  S01
............................................................................cuucccuacuccaUccaugcc..............  1   1  S01
............................................................................cuucccuacuccacccaugcG..............  1   1  S01
............................................................................cuuGccuacuccacccaugcca.............  1   1  S01
............................................................................cuucccuacuccacccaugcca.............  15  0  S01
............................................................................cuucccuacuccacccaugccG.............  1   1  S01
............................................................................cuucccuacuccacccaugccaC............  2   1  S01
............................................................................cuucccuacuccacccaugccau............  8   0  S01
............................................................................cuucccuacuccacccaugccaua...........  4   0  S01
............................................................................cuucccuacuccacccaugccauU...........  1   1  S01
............................................................................cuucccuacuccacccaugUcaua...........  1   1  S01
............................................................................cuucccuacuccacccaugccauaA..........  4   1  S01
............................................................................cuucccuacGccacccaugccauag..........  1   1  S01
............................................................................cuucccuacuccacccaugccauag..........  18  0  S01
............................................................................cGucccuacuccacccaugccauag..........  1   1  S01
............................................................................cuucccuacuccacccaugccauagu.........  6   0  S01
............................................................................cuucccuacuccacccaugccauagC.........  1   1  S01
.............................................................................uucccuacuccacccaug................  1   0  S01
.............................................................................uucccuacuccacccaugc...............  1   0  S01
.............................................................................uucccuacuccacccGugcc..............  1   1  S01
.............................................................................uucccuacuccacccaugcc..............  6   0  S01
.............................................................................uucccuacuccacccaugccG.............  1   1  S01
.............................................................................uucccuacuccacccaugccaC............  1   1  S01
.............................................................................uucccuacuccacccGugccau............  1   1  S01
.............................................................................uucccuacuccacccaugccau............  12  0  S01
.............................................................................uucccuacuccacccaugccaua...........  11  0  S01
.............................................................................uucccuacuccacccaugccauag..........  13  0  S01
.............................................................................uucccuacuccacccaugccauagC.........  1   1  S01
.............................................................................uucccuacuccacccaugccauagG.........  1   1  S01
.............................................................................uucccuacuccacccaugccauagu.........  8   0  S01
..............................................................................ucccuacuccacccaugc...............  1   0  S01
..............................................................................ucccuacuccacccaugcc..............  9   0  S01
..............................................................................ucccuacuccacccaugcG..............  1   1  S01
..............................................................................ucccuacuccacccaugcU..............  1   1  S01
..............................................................................ucccuacuccacccaugcca.............  2   0  S01
..............................................................................ucccuacuccacccaugccUu............  1   1  S01
..............................................................................ucccuacuccaUccaugccau............  1   1  S01
..............................................................................ucccuacuccacccaugccau............  3   0  S01
..............................................................................ucUcuacuccacccaugccaua...........  1   1  S01
..............................................................................uGccuacuccacccaugccaua...........  1   1  S01
```

Mature Star

ggauuuugggaagguuuuggugugggugguuagggaagauaacuaacuaguuugaauauguuaagguuuuguuuaucuucccuacuccacccaugccauaguuuucccauc			
....ucccuacuccacccaugccaua...........	18	0	S01
....ucccuacuccacccaugccauagA.........	1	1	S01
....ucccuacuccacccaugccauagu.........	8	0	S01
....ucccuacuccacccaugccauaguu........	1	0	S01
....cccuacuccacccaugcc..............	1	0	S01
....cccuacuccacccaugccau............	1	0	S01
....ccuacuccacccaugccau............	1	0	S01
....ccuacuccacccaugccauag..........	2	0	S01
...uuuugggaagguuuugguguggg...	1	0	S02
.....uugggaagguuuCgguguggg...	1	1	S02
........ggaagguuuuggugugggugg..	1	0	S02
...............uuuggugugggugguuaggga...	1	0	S02
................uuggugugggCgguuagg...	1	1	S02
................uuggugugggugguuagg...	1	0	S02
................uuggugugggugguuaggg..	1	0	S02
................uuggugugggugguuaggga...	11	0	S02
................uuggugugggugguuagggaa..	1	0	S02
................uuggugugggugguuagggaag...	9	0	S02
................uuggugugggugguuagggaaAa..	1	1	S02
................uugUugugggugguuagggaaga..	1	1	S02
................uuggugugggAgguuagggaaga..	1	1	S02
................uuggugugggugguuagggaaga..	8	0	S02
................uuggugugggugguuagggaagau...	4	0	S02
................uuggugugggugguuagggaagaC...	1	1	S02
.................uggugugggugguuaggg..	2	0	S02
.................Aggugugggugguuaggga...	1	1	S02
.................uggugugggugguuaggga...	5	0	S02
.................uggugugggugguuagggaag...	30	0	S02
.................uggugugggugguuagggaaA...	1	1	S02
.................uggugugggugguuagggaaC...	1	1	S02
.................uggugugggugguuagggaagG..	1	1	S02
.................uggugugggugguuagggaaga..	5	0	S02
.................uggugugggugguuagggaagC..	1	1	S02
.................uggugugCgugguuagggaaga..	1	1	S02
.................uggugugggugguuagggaagau...	1	0	S02
.................uggugugggCgguuagggaagaua..	1	1	S02
..................ggugugggugguuaggga...	3	0	S02
..................ggugugggugguuagggaag...	3	0	S02
..................ggugugggugguuagggaaga..	4	0	S02
...................gugggugguuagggaag...	1	0	S02
...................gugggugguuagggaagG..	1	1	S02
...................gugggGgguuagggaagau...	3	1	S02
...................gugggugguuagggaagau...	6	0	S02
...................gugCgugguuagggaagau...	1	1	S02
...................gugggugguuagggaagaG...	1	1	S02
...................gugggugguuagggaagaua..	1	0	S02
...................gugggugguuagggaagauU..	1	1	S02
....................ugggugguuagggaaC...	5	1	S02
....................ugggugguuagggaCg...	1	1	S02
....................ugggugguuagggaag...	20	0	S02
....................ugggugguuagggaaA...	1	1	S02
....................ugggugguuagggaaga..	18	0	S02
....................ugggugguuagggGaga..	1	1	S02
....................ugggugguuagggaagC..	1	1	S02
....................ugggugguuagggaagG..	1	1	S02
....................ugggugguuGgggaagau...	3	1	S02
....................ugggugguuagggaagau...	348	0	S02
....................ugggugguCuagggaagau..	2	1	S02
....................uguUgggugguuagggaagau..	2	1	S02
....................ugggugguuaggAaagau...	1	1	S02
....................ugggugguuagggaGgau...	4	1	S02
....................ugggugguuagggaagaC...	33	1	S02
....................ugggugguuagggaagaG...	10	1	S02
....................ugggugguuaUggaagau...	1	1	S02
....................ugggAgguuagggaagau...	1	1	S02
....................ugggugguGuagggaagau..	1	1	S02
....................ugggCgguuagggaagau...	1	1	S02
....................ugggugguuagggGagau...	1	1	S02
....................ugggugguAuagggaagau..	3	1	S02

Mature **Star**

```
ggauuuugggaagguuuuggugugggugguuagggaagauaacuaacuaguuugaauauguuaagguuuuguuuaucuucccuacuccacccaugccauaguuuucccauc
....................ugugggugguuaCggaagau.......................................................................   1      1      S02
....................uAugggugguuagggaagau.......................................................................   2      1      S02
....................uCugggugguuagggaagau.......................................................................   1      1      S02
....................uguggguggguAagggaagau......................................................................   1      1      S02
....................ugugggGgguuagggaagau.......................................................................   3      1      S02
....................ugugggugguuagggaagaA.......................................................................   2      1      S02
....................ugugggugguuagCgaagaua......................................................................   1      1      S02
....................ugugggugguuagggaagaua......................................................................   1548   0      S02
....................uguggAugguuagggaagaua......................................................................   3      1      S02
....................uguggguggguCagggaagaua.....................................................................   5      1      S02
....................uguggguggguAagggaagaua.....................................................................   2      1      S02
....................uguAggugguuagggaagaua......................................................................   2      1      S02
....................ugCgggugguuagggaagaua......................................................................   5      1      S02
....................ugugggugguuagggaagauC......................................................................   104    1      S02
....................Ngugggugguuagggaagaua......................................................................   1      1      S02
....................ugugggugguuaggAaagaua......................................................................   2      1      S02
....................ugugggugAuuagggaagaua......................................................................   1      1      S02
....................ugugggugguuagggaagGua......................................................................   5      1      S02
....................ugugggugguuaAggaagaua......................................................................   3      1      S02
....................ugugggugguuGgggaagaua......................................................................   4      1      S02
....................ugugggCgguuagggaagaua......................................................................   6      1      S02
....................ugugUgugguuagggaagaua......................................................................   1      1      S02
....................ugugggugguuagggaagauU......................................................................   91     1      S02
....................uguCggugguuagggaagaua......................................................................   1      1      S02
....................ugugggAgguuagggaagaua......................................................................   8      1      S02
....................uguUggugguuagggaagaua......................................................................   14     1      S02
....................Cgugggugguuagggaagaua......................................................................   4      1      S02
....................uguggguggCuagggaagaua......................................................................   5      1      S02
....................ugugggugguuagggaaAaua......................................................................   1      1      S02
....................ugugggugguuagggUagaua......................................................................   3      1      S02
....................ugugggugguuagggaaCaua......................................................................   1      1      S02
....................ugAgggugguuagggaagaua......................................................................   1      1      S02
....................uguggguggAuagggaagaua......................................................................   4      1      S02
....................uAugggugguuagggaagaua......................................................................   3      1      S02
....................Agugggugguuagggaagaua......................................................................   3      1      S02
....................uguggguAguuagggaagaua......................................................................   5      1      S02
....................ugugggugguuagggaagaGa......................................................................   1      1      S02
....................uguggguggGuagggaagaua......................................................................   6      1      S02
....................ugugggugguuagUgaagaua......................................................................   1      1      S02
....................ugugggugguuagggaGgaua......................................................................   3      1      S02
....................ugGgggugguuagggaagaua......................................................................   1      1      S02
....................ugugggugguuagggaagaCa......................................................................   6      1      S02
....................ugugggugguuagggaagauG......................................................................   25     1      S02
....................ugugggugguuagggCagaua......................................................................   1      1      S02
....................ugugggugguuaggUaagaua......................................................................   1      1      S02
....................ugugggugguuagggGagaua......................................................................   9      1      S02
....................ugugggGgguuagggaagaua......................................................................   8      1      S02
....................ugugggugguuagggaagauaC.....................................................................   3      1      S02
....................ugugggugguuagggaagauaa.....................................................................   1      0      S02
....................ugugggugguuagggaagauaacu...................................................................   1      0      S02
....................ugugggugguuagggaagauaacA...................................................................   1      1      S02
.....................gugggugguuagggaagau.......................................................................   7      0      S02
.....................Uugggugguuagggaagau.......................................................................   1      1      S02
.....................gugggugguuagggaagaua......................................................................   28     0      S02
.....................gugggugguuagggaagauG......................................................................   1      1      S02
.....................gugggugguuagggaagauU......................................................................   1      1      S02
.....................gugggugguuagggaagauC......................................................................   4      1      S02
.....................gugggugguuagggaagauaacu...................................................................   1      0      S02
......................uggguggGuagggaagau.......................................................................   1      1      S02
......................ugggugguuagggaagau.......................................................................   1      0      S02
......................ugggugguuagggaagaua......................................................................   2      0      S02
............................................uaacuaguuugaauauguuaag.............................................   1      0      S02
.........................................................................uaucuucccuacuccacccaugcc..............   2      0      S02
..........................................................................aucuucccuacuccacccaugc...............   1      0      S02
..........................................................................aucuucccuacuccacccaugcc..............   1      0      S02
...........................................................................ucuuccGuacuccaccca..................   1      1      S02
...........................................................................ucuucccuacuccaccca..................   11     0      S02
...........................................................................ucuucccuacucUaccca..................   1      1      S02
...........................................................................ucuucccuacuccacccau.................   3      0      S02
...........................................................................uUuucccuacuccacccau.................   1      1      S02
```

Mature Star

ggauuuugggaagguuuuggugugggugguuagggaagauaacuaacuaguuugaauauguuaagguuuuguuuaucuucccuacuccacccaugccauaguuuucccauc			
....ucuucccuacuccacccaCg................	1	1	S02
....ucuucccuacuccacccaug................	19	0	S02
....ucuucccuacuccacccauU................	2	1	S02
....ucuucccuacuccacccGug................	1	1	S02
....ucuucccuacuccacccauA................	1	1	S02
....Ccuucccuacuccacccaugc...............	1	1	S02
....ucuucccAacuccacccaugc...............	1	1	S02
....ucuucccuacuccacccaCgc...............	1	1	S02
....ucuCcccuacuccacccaugc...............	1	1	S02
....ucuucccGacuccacccaugc...............	1	1	S02
....ucuucccuacuccacccauUc...............	1	1	S02
....Acuucccuacuccacccaugc...............	1	1	S02
....ucuucccuacuccaAccaugc...............	1	1	S02
....Ncuucccuacuccacccaugc...............	1	1	S02
....ucuucccuacucUacccaugc...............	1	1	S02
....uUuucccuacuccacccaugc...............	1	1	S02
....ucuucccuacuccacccaugU...............	28	1	S02
....ucuucccuacuccacccaugc...............	181	0	S02
....ucuucccuacuccaUccaugc...............	1	1	S02
....ucuucccuacuccacccaugG...............	1	1	S02
....ucuucccuacuccacccaugA...............	6	1	S02
....ucCucccuacuccacccaugc...............	1	1	S02
....ucuucccuacuccacccaGgcc..............	2	1	S02
....ucuucccuacuccacccaCgcc..............	4	1	S02
....uUuucccuacuccacccaugcc..............	3	1	S02
....ucuucccuacuccacGcaugcc..............	1	1	S02
....ucuCcccuacuccacccaugcc..............	2	1	S02
....ucuucAcuacuccacccaugcc..............	5	1	S02
....ucuucccuacuccacccauUcc..............	1	1	S02
....ucuucccuacuccCcccaugcc..............	2	1	S02
....ucuucccuacuccGcccaugcc..............	6	1	S02
....ucuucccuacuccacUcaugcc..............	1	1	S02
....ucuucccGacuccacccaugcc..............	2	1	S02
....ucuucccAacuccacccaugcc..............	1	1	S02
....ucuucUcuacuccacccaugcc..............	2	1	S02
....ucCucccuacuccacccaugcc..............	3	1	S02
....ucuucccuacuccacccCugcc..............	2	1	S02
....ucuucccuacuccacccaugcG..............	32	1	S02
....ucuucccuacucAacccaugcc..............	1	1	S02
....ucuucccuacuccacccauAcc..............	2	1	S02
....Ccuucccuacuccacccaugcc..............	1	1	S02
....ucuucccuacuccacccaugUc..............	2	1	S02
....ucuucccuUcuccacccaugcc..............	1	1	S02
....ucuucGcuacuccacccaugcc..............	1	1	S02
....ucuucccuacuccacccGugcc..............	3	1	S02
....ucuucccuGcuccacccaugcc..............	2	1	S02
....ucuuccUuacuccacccaugcc..............	1	1	S02
....ucuucccuacuUcacccaugcc..............	2	1	S02
....ucuucccuaAuccacccaugcc..............	1	1	S02
....ucuucccuacuccacccaAgcc..............	1	1	S02
....ucuucccuacuccacccaugcU..............	283	1	S02
....Ncuucccuacuccacccaugcc..............	3	1	S02
....ucuucccuacuccacccaugcc..............	1090	0	S02
....ucuucccuacuccaccUaugcc..............	2	1	S02
....ucuucccCacuccacccaugcc..............	3	1	S02
....ucuucccuacCccacccaugcc..............	5	1	S02
....ucuucccuacuccaAccaugcc..............	2	1	S02
....ucuucccuacuccacAcaugcc..............	1	1	S02
....ucuucccuacuccacccauCcc..............	1	1	S02
....ucuucccuacGccacccaugcc..............	1	1	S02
....ucuGcccuacuccacccaugcc..............	2	1	S02
....ucuucccuacuccaUccaugcc..............	1	1	S02
....ucuucccuacuAcacccaugcc..............	1	1	S02
....ucuucccuacAccacccaugcc..............	2	1	S02
....ucuucccuacuccacccaugAc..............	3	1	S02
....ucuucccuacuccacccNugcc..............	1	1	S02
....ucuucccuacuccaGccaugcc..............	1	1	S02
....ucuucccuacuccaccGaugcc..............	1	1	S02
....ucuucccuaUuccacccaugcc..............	3	1	S02
....ucuucccuacuccUcccaugcc..............	2	1	S02

Mature Star

ggauuuugggaagguuuuggugugguggguuagggaagauaacuaacuaguuugaauauguuaagguuuuguuuaucuucccuacuccacccaugccauaguuuucccauc			
....ucuucccuacuccacccaugcA..............	76	1	S02
....ucuucccuaGuccacccaugcc..............	4	1	S02
....Acuucccuacuccacccaugcc..............	2	1	S02
....ucAucccuacuccacccaugcc..............	2	1	S02
....Gcuucccuacuccacccaugcc..............	1	1	S02
....ucuucccuacuccacccaugccG.............	1	1	S02
....ucuucccuacuccacccaugcca.............	1	0	S02
....ucuucccuacuccacccaugccU.............	6	1	S02
....ucuucccuacuccacccaugccUu............	1	1	S02
....ucuucccuacuccacccaugccaua...........	1	0	S02
....cuucccuacuccacccaug................	1	0	S02
....cuucccuacuccacccauC................	1	1	S02
....cuucccuacuccacccauU................	1	1	S02
....cuucccuacuccacccGugc...............	1	1	S02
....cuucccuacuccacccaugc...............	3	0	S02
....cuCcccuacuccacccaugcc..............	1	1	S02
....cuucccuacuccacccaugcc..............	14	0	S02
....cuucccuacuccacccaugcU..............	5	1	S02
....cuucccuacuccacccaugccG.............	1	1	S02
....cuucccuacuccacccaugcca.............	9	0	S02
....cuucccuacuccacccaugccU.............	1	1	S02
....cuucccuacuccacccaugccaC............	1	1	S02
....cuucccuacuccacccaugccau............	1	0	S02
....cuucccuacuccacccaugccaua...........	3	0	S02
....uucccuacuccacccaug................	2	0	S02
....uucccuacuGcacccaug................	1	1	S02
....uucccuacuccacccauUcc..............	1	1	S02
....uucccuacuccacccaugcc..............	8	0	S02
....uucccuacuccacccaugcU..............	2	1	S02
....uucccuacuccacccaugcG..............	1	1	S02
....uucccuacuccacccaugccG.............	1	1	S02
....uucccuacuccacccaugcca.............	2	0	S02
....uucccuacuccacccaugccaG............	1	1	S02
....uucccuacuccacccaugccaC............	1	1	S02
....uucccuacuccacccaCgccau............	1	1	S02
....uucccuacuccacccaugccau............	15	0	S02
....uucccuacuccacGcaugccau............	1	1	S02
....uucccuacuccacccaugGcaua...........	1	1	S02
....uucccuacuccacccaugccauC...........	1	1	S02
....uucccuacuccacccaugccaua...........	11	0	S02
....uucccuacuccacccaugccauU...........	1	1	S02
....uucccuacuccacccaugccauG...........	2	1	S02
....uucccuacuccacccaugccauag..........	2	0	S02
....uucUcuacuccacccaugccauag..........	1	1	S02
....uucccuacuccacccaugccauagu.........	3	0	S02
....uucccuacuccacccaugccauagA.........	1	1	S02
....ucccuacuccacccaugc...............	1	0	S02
....ucccuacuccacccaugcc..............	2	0	S02
....ucccuacuccacccaugcU..............	4	1	S02
....ucccuacuccacccaugcca.............	2	0	S02
....ucccuacuccacccaugccaC............	1	1	S02
....ucccuacuccacccaugccau............	10	0	S02
....ucccuacuccacccaugccaG............	1	1	S02
....Ncccuacuccacccaugccaua...........	1	1	S02
....ucccuacuccacccaugccaua...........	19	0	S02
....ucccuacuccacccaugccauag..........	1	0	S02
....ucccuacuccacccaugccauagu.........	7	0	S02
....ucccuacuccacccaugccauagG.........	2	1	S02
....cccuacuccacccaugcc..............	1	0	S02
....cccuacuccacccaugccaua...........	1	0	S02
....ccuacuccacccaugccau............	1	0	S02
....caugccauaguuuucccauU	1	1	S02

Provisional ID	:	Maple_Unigene_BMK.34062_160827
Score total	:	0.3
Score for star read(s)	:	-1.3
Score for read counts	:	0
Score for mfe	:	2.2
Score for randfold	:	0
Score for cons. seed	:	-0.6
Total read count	:	213
Mature read count	:	150
Loop read count	:	61
Star read count	:	2

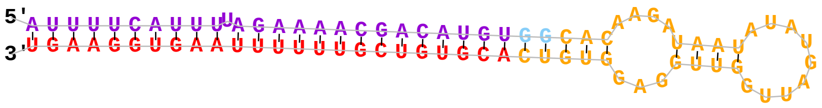

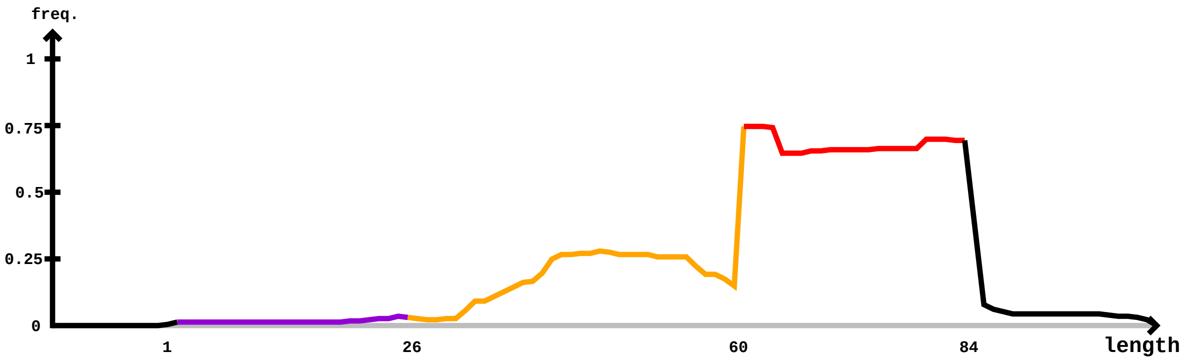

Star **Mature**

```
5'- cucaccaugaaauuuucauuuuagaaaacgacauguggcacaagauaauauaugauugguuggaggugucacgugucguuuuuuaaguggaagucucacggugagaacguccc   -3'   obs
    cucaccaugaaauuuucauuuuagaaaacgacauguggcacaagauaauauaugauugguuggaggugucacgugucguuuuuuaaguggaagucucacggugagaacguccc         exp
    ((((((.(((.((((((((((.(((((((((((((((((((....((((.........))))...))))))))))))))))))))))))))))).))).))))))........   reads   mm   sample
    ..........aauuuucauuuuagaaaacgacaug..............................................................................   1   0   S01
    ...........auuuucauuuuagaaaacgacaugu.............................................................................   1   0   S01
    ...........auuuucauuuuagaaaacgacaugC.............................................................................   1   1   S01
    .............................gacauguggcacaagauaauau..............................................................   1   0   S01
    ...............................caugugAcacaagauaaua...............................................................   1   1   S01
    ..................................gugAcacaagauaauauaugauu........................................................   1   1   S01
    .......................................acaagauaauauaugaAugguu....................................................   1   1   S01
    .........................................aagauaauauaugauugUuuggag................................................   2   1   S01
    .........................................aagauaauauaugauugAuuggag................................................   1   1   S01
    .........................................aagauaauauaugauuUguuggag................................................   1   1   S01
    ..........................................agauaauauaugauugUuuggag................................................   1   1   S01
    ..........................................agauaauauaugauugguuggagU...............................................   1   1   S01
    ............................................auaauauaugauugUuuggag................................................   1   1   S01
    ............................................auaauauaugauugguuggaUgug.............................................   3   1   S01
    ...............................................auauaugauugguuggaUgugu............................................   1   1   S01
    ...............................................auauaugauugguuggaUguguc...........................................   2   1   S01
    .................................................auaugauugguuggaUgug.............................................   1   1   S01
    .................................................auaugauugguuggaUgugucacg........................................   2   1   S01
    .................................................auaugauugguuggaUgugucacgu.......................................   1   1   S01
    ..................................................uaugauugguuggaUgugucacgu.......................................   8   1   S01
    ...................................................augauugguuggaUgugucacgu.......................................   4   1   S01
    .......................................................uugguuggaUgugucacgu.......................................   1   1   S01
    .....................................................................cacgugucguuuuuuaaguggaa.....................   1   0   S01
    ......................................................................acgugucguuuuuuaaguggaagC...................   4   1   S01
    ......................................................................acgugucguuuuuuaaguggaagA...................   2   1   S01
    ......................................................................acgugucguuuuuuaaguggaagG...................   2   1   S01
    ......................................................................acgugucguuuuuuUaguggaagu...................   1   1   S01
    ......................................................................acgugucguuuuuuaaguggaagu...................   65  0   S01
    ......................................................................acgugGcguuuuuuaaguggaagu...................   1   1   S01
    ......................................................................acgugucguuuuuuaaguggaaguUu.................   1   1   S01
    .........................................................................ugucguuuuuuaaguggaagucu.................   1   0   S01
    .........................................................................ugucguuuuuuaaguggaagucuU................   1   1   S01
    ................................................................................uuuuaaguggaagucucacggugagaacC....   1   1   S01
```

Star **Mature**

cucaccaugaaauuuucauuuuagaaaacgacauguggcacaagauaauauaugauugguuggaggugucacgugucguuuuuuaaguggaagucucacggugagaacguccc			
....gaagucucacggugagaacguc..	1	0	S01
....gaagucucacggugagaacgucc.	1	0	S01
....gaagucucacggugagaacguccc	3	0	S01
................................auguggcacaagauaauauaugau...	1	0	S02
..................................gugAcacaagauaauauaugauu..	1	1	S02
....aagauaauauaugauugUuuggag..	2	1	S02
....aagauaauauaugauugguuggaUg...	1	1	S02
....agauaaGauaugauugguu..	1	1	S02
....agauaauauaugauugguuggaUg...	3	1	S02
....agauaauauaugauugguugAagg...	1	1	S02
....agauaauauaugauugUuuggagg...	1	1	S02
....uaauauaugauugguuggaUgugu..	4	1	S02
....aauauaugauugguuggaUgugu..	1	1	S02
....aauauaugauugguuggaUguguc...	3	1	S02
....auauaugauugguuggaUgugucac...	1	1	S02
....uauaugauugguuggaUgugucac...	1	1	S02
....auaugauugguuggaUgugu..	1	1	S02
....auaugauugguuggaUgugucacg..	2	1	S02
....uaugauugguuggaUgugucacgu.......................................	5	1	S02
....augauugguuggaUgugucacgu.......................................	1	1	S02
....gauugguuggaUgugucacgu.......................................	1	1	S02
....uugguuggaUgugucacgu.......................................	1	1	S02
....acgCgucguuuuuuaaguggaagu...................	1	1	S02
....acgugucguuuuuuaaguggaagG...................	7	1	S02
....acgugucguuuuuuaaguggaagu...................	47	0	S02
....acgugCcguuuuuuaaguggaagu...................	1	1	S02
....acgugucguuuuuCaaguggaagu...................	1	1	S02
....acgugucguuuuuuaaguggaagC...................	4	1	S02
....acgAgucguuuuuuaaguggaagu...................	1	1	S02
....acgugucguuuAuuaaguggaagu...................	1	1	S02
....acgugucgGuuuuuaaguggaagu...................	2	1	S02
....acgugucguuuuuuaagugAaagu...................	1	1	S02
....gugucguuuuuuaaguggaagucu.................	2	0	S02
....ugucguuuuuuaaguggaagucuc................	1	0	S02
....guuuuuuaaguggaagucuca...............	2	0	S02
....aaguggaagucucacggugagaac.....	1	0	S02
....gaagucucacggugagaacgucc.	1	0	S02
....gaagucucacggugagaacguccU	1	1	S02
....gaagucucacggugagaacguccc	1	0	S02

Provisional ID	:	Maple_Unigene_BMK.34307_163173
Score total	:	66.2
Score for star read(s)	:	3.9
Score for read counts	:	60.3
Score for mfe	:	-0.9
Score for randfold	:	0
Score for cons. seed	:	3
Total read count	:	130
Mature read count	:	114
Loop read count	:	5
Star read count	:	11

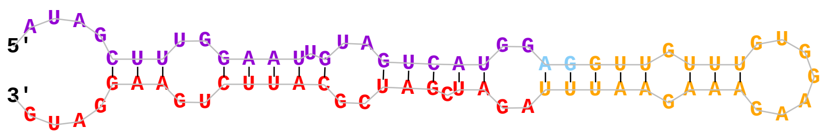

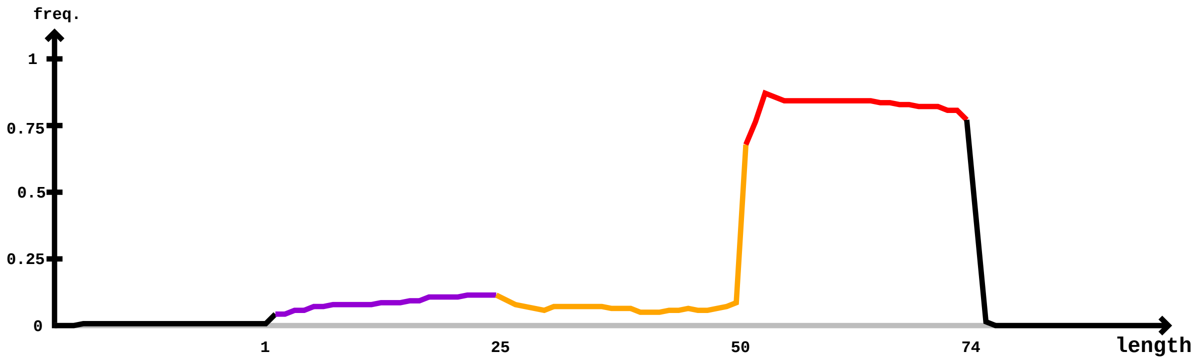

Star **Mature**

```
5'- uaacugaaaaggcagauguuuauagcuuuggaauuguagucauggagguuguuuguggaagaaagaauuuagaucgaucgcauucugaaggaugcauacauuugcucccuuuuu   -3'   obs
    uaacugaaaaggcagauguuuauagcuuuggaauuguagucauggagguuguuuguggaagaaagaauuuagaucgaucgcauucugaaggaugcauacauuugcucccuuuuu         exp
    .....((((((((((((((.(((..(((..((((.(..(((((..(((((.(((.......))).)))))..)).)))..)))))..))).)))...))))))))...))))))  reads  mm  sample
    .aacugaaaaggcGgauguuu.............................................................................................  1  1  S01
    .....................auagcuuuggaauuguagucaugg.....................................................................  4  0  S01
    .......................agcuuuggaauuguagucauggag...................................................................  1  0  S01
    .......................agcuuuggaauuguagucauggagg..................................................................  1  0  S01
    .........................cuuuggaauuguagucauggaggu.................................................................  1  0  S01
    .....................................agucauggagguuguCuguggaag.....................................................  1  1  S01
    .........................................auggagguuguCuguggaagaaag.................................................  1  1  S01
    .............................................agguuguCuguggaagaaagaauu.............................................  1  1  S01
    .................................................uguCuguggaagaaagaauuuagau........................................  1  1  S01
    ..................................................guuuguggaagaaagaauuuaga.........................................  1  0  S01
    ..................................................guuuguggaagaaagaGuuuaga.........................................  1  1  S01
    ..................................................guCuguggaagaaagaauuuagau........................................  1  1  S01
    ....................................................................uuagaucgaucgcauucugaagg.......................  1  0  S01
    .....................................................................uagaucgaucgcauucugaaggau.....................  3  0  S01
    ......................................................................agaucgaucgcauucugaaggau.....................  1  0  S01
    ......................................................................agaucgaucgcauCcugaaggaug....................  1  1  S01
    ......................................................................agaucgaucgcauucugaaggaug....................  39  0  S01
    ......................................................................agaucgaucgcauucugaaggauA....................  1  1  S01
    ......................................................................agaucgaucgcaGucugaaggaug....................  6  1  S01
    ......................................................................agauAgaucgcauucugaaggaug....................  1  1  S01
    ......................................................................agaucgaucgcauucugaaggaCg....................  1  1  S01
    ......................................................................Ggaucgaucgcauucugaaggaug....................  1  1  S01
    ......................................................................agCucgaucgcauucugaaggaug....................  1  1  S01
    ......................................................................agaucgauGgcauucugaaggaug....................  2  1  S01
    ......................................................................agaucgaucgcaGucugaaggaugc...................  1  1  S01
    .......................................................................gaucgaucgcaGucugaaggaugc...................  3  1  S01
    .......................................................................gaucgaucgcauucAgaaggaugc...................  1  1  S01
    .......................................................................gaucgaucgcauucugaaggaugA...................  1  1  S01
    .......................................................................gaucgaucgcauucugaaggaugU...................  1  1  S01
    .......................................................................gaucgaucgcauucugaaggaugc...................  4  0  S01
    ........................................................................aCcgaucgcauucugaaggau.....................  1  1  S01
    ........................................................................aucgaucgcauucugaaggaugA...................  2  1  S01
    ........................................................................aucgaucgcauucugaaggaugc...................  2  0  S01
```

```
                     Star                                                  Mature
uaacugaaaaggcagauguuuauagcuuuggaauuguagucauggagguuguuuguggaagaaagaauuuagaucgaucgcauucugaaggaugcauacauuugcucccuuuuu
........................................................................aucgaucgcaGucugaaggaugc...................   4   1   S01
........................................................................aucgaucCcauucugaaggaugc...................   1   1   S01
........................................................................aucgaucgcauucugaaggaugca..................   2   0   S01

.....................auagcuuuggaauuguagucaugg.....................................................................   2   0   S02
.........................cuuuggGauuguagucauggaggu.................................................................   1   1   S02
...........................uuggaauuguagucauggagguu................................................................   1   0   S02
................................auuguagucauggagguuguCugu..........................................................   1   1   S02
...................................guagucauggagguuguCugugga.......................................................   1   1   S02
.....................................agucauggaAguuguuugugga.......................................................   1   1   S02
.............................................................aaagaauuuagaucgaucAcauu..............................   1   1   S02
..............................................................aagaauuuagaucgaucgcaGucu............................   1   1   S02
................................................................gaauuuagaucgaucgcauucuAa..........................   1   1   S02
...................................................................uuuagaucgaucgcauucugaagg.......................   1   0   S02
......................................................................agaucgaucgcauucugaaggaCg....................   2   1   S02
......................................................................agaucgaucgcaGucugaaggaug....................   2   1   S02
......................................................................agaucgaucgcauucugaaggauA....................   1   1   S02
......................................................................agaucgaucgcauucugGaggaug....................   1   1   S02
......................................................................agaucgaucgcauucugaaggaug....................  22   0   S02
.......................................................................gaucgaucgcaGucugaaggaugc...................   1   1   S02
.......................................................................gaucgaucgcauucugaaggaugc...................   1   0   S02
........................................................................aucgaucgcaGucugaaggaugc...................   2   1   S02
........................................................................aucgaucgcauucugaUggaugc...................   1   1   S02
```

Provisional ID	:	Maple_Unigene_BMK.34474_164846
Score total	:	3.2
Score for star read(s)	:	-1.3
Score for read counts	:	0
Score for mfe	:	1.5
Score for randfold	:	0
Score for cons. seed	:	3
Total read count	:	12
Mature read count	:	8
Loop read count	:	1
Star read count	:	3

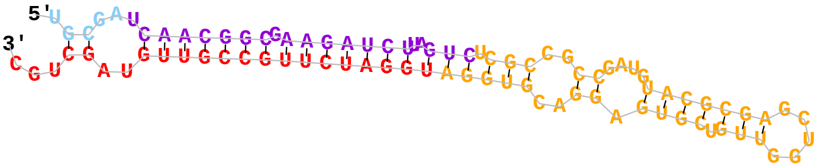

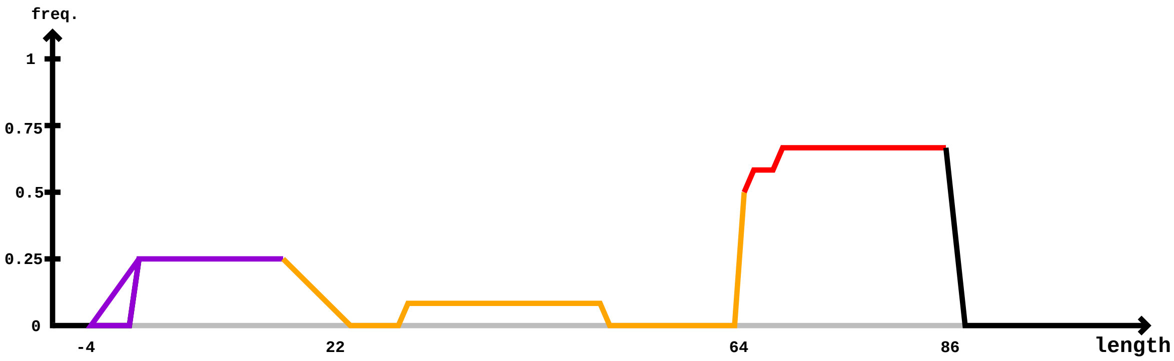

Star Mature

```
5'- guugcgaucaacggcgaagaucuuagucucgccgccgauguacgcgagcugguugucgugaggacguggauggaucuugccguuguagcugcacaugaauuugaccuuguag   -3'    obs
    guugcgaucaacggcgaagaucuuagucucgccgccgauguacgcgagcugguugucgugaggacguggauggaucuugccguuguagcugcacaugaauuugaccuuguag          exp
    ((.((...(((((((.(((((((..(((.(((..((....(((((((.....))).)))).))..))))))))))))))))))))..)).))....................    reads     mm     sample
    .......ucaacggcgaagaucuuaguc....................................................................................    2         0      S01
    ...................................cgauguacgcgagcugguugu........................................................    1         0      S01
    ......................................................................uggaucuugccguuguagcugc....................    3         0      S01
    .......................................................................ggaucuugccguuguagcugU....................    1         1      S01

    .......ucaacggcgaagaucuuaguc....................................................................................    1         0      S02
    ......................................................................uggaucuugccguuguagcugc....................    3         0      S02
    ..........................................................................ucuugccguuguagcugU....................    1         1      S02
```

Provisional ID	:	Maple_Unigene_BMK.34771_167954
Score total	:	821.6
Score for star read(s)	:	3.9
Score for read counts	:	812.3
Score for mfe	:	2.4
Score for randfold	:	0
Score for cons. seed	:	3
Total read count	:	1605
Mature read count	:	1295
Loop read count	:	12
Star read count	:	298

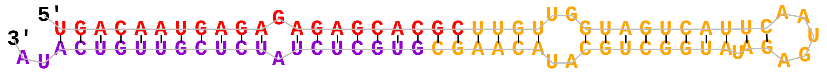

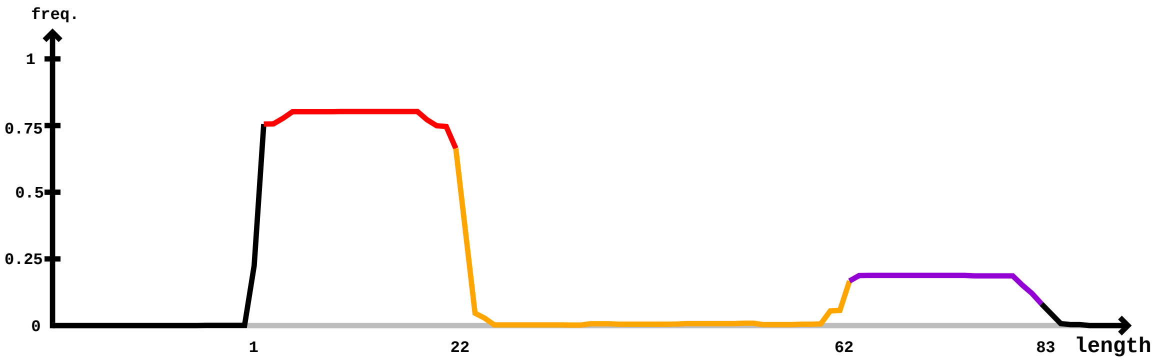

Mature Star

```
5'- agacuugaugcaucauuauuugacaaugagagagagcacgcuuguugguagucauucaaugagauauggcugcauacaagcgugcucuaucucguugucauaugauuugc -3'   obs
    agacuugaugcaucauuauuugacaaugagagagagcacgcuuguugguagucauucaaugagauauggcugcauacaagcgugcucuaucucguugucauaugauuugc        exp
    .........(((..(((((.(((((((((((.(((((((((((((..((((((((((.....)).))))))))..))))))))))))).))))))))))).))))).)))   reads   mm   sample
    ..............auuGuuugacaaugagagagagcacg......................................................................   1       1    S01
    ...................uugacaaugagagagagU.........................................................................   2       1    S01
    ...................uugacaaugagagagagc.........................................................................   28      0    S01
    ...................uugacaaugagagagagG.........................................................................   1       1    S01
    ...................uugaAaaugagagagagc.........................................................................   1       1    S01
    ...................uugacaaugagagCgagc.........................................................................   1       1    S01
    ...................uugacaaugagagagagA.........................................................................   1       1    S01
    ...................uugacaaugagagagagca........................................................................   2       0    S01
    ...................uugacaaCgagagagagca........................................................................   1       1    S01
    ...................uugacaaugagagagagcacA......................................................................   6       1    S01
    ...................uugacaaugagagagagAacg......................................................................   1       1    S01
    ...................uGgacaaugagagagagcacg......................................................................   1       1    S01
    ...................uugacaaugagagagagcacg......................................................................   54      0    S01
    ...................uugacaaugagagagagcacC......................................................................   1       1    S01
    ...................uuUacaaugagagagagcacg......................................................................   1       1    S01
    ...................uugacaaugagGgagagcacgc.....................................................................   1       1    S01
    ...................uugacaaugagagagCgcacgc.....................................................................   1       1    S01
    ...................uugacaaugagagagaUcacgc.....................................................................   1       1    S01
    ...................uugacaaugagagagagcacgc.....................................................................   78      0    S01
    ...................uCgacaaugagagagagcacgc.....................................................................   1       1    S01
    ...................uugacaaugagagagagcacgU.....................................................................   17      1    S01
    ...................uugacaaugagagagagcacgA.....................................................................   3       1    S01
    ...................uugacaaugagagGgagcacgc.....................................................................   1       1    S01
    ....................ugacaaugagagagagAa........................................................................   1       1    S01
    ....................ugacaaugagagagagca........................................................................   16      0    S01
    ....................ugacaaugagGgagagca........................................................................   1       1    S01
    ....................ugacaaugaAagagagca........................................................................   1       1    S01
    ....................ugacaaugagagagagcac.......................................................................   2       0    S01
    ....................ugacaaugagagagagcacg......................................................................   3       0    S01
    ....................ugacaaugagagagagcacU......................................................................   1       1    S01
    ....................uCacaaugagagagagcacgc.....................................................................   1       1    S01
    ....................ugacUaugagagagagcacgc.....................................................................   1       1    S01
    ....................Agacaaugagagagagcacgc.....................................................................   1       1    S01
```

Mature / Star			
agacuugaugcaucauuauuugacaaugagagagagcacgcuuguugguagucauucaaugagauauggcugcauacaagcgugcucuaucucguugucauaugauuugc			
....................ugacaaugagagagUgcacgc...	2	1	S01
....................Ngacaaugagagagagcacgc...	1	1	S01
....................ugacaaugagagagaAcacgc...	1	1	S01
....................ugacaaugagagagagcaAgc...	3	1	S01
....................ugacaauAagagagagcacgc...	2	1	S01
....................ugacaaugagagagagAacgc...	1	1	S01
....................ugacaaugagagagGgcacgc...	2	1	S01
....................ugacaaugagagagagcacgG...	2	1	S01
....................ugacaaugagagagagcacgA...	12	1	S01
....................ugacaaugagagagagcacgU...	66	1	S01
....................ugacCaugagagagagcacgc...	1	1	S01
....................ugacaaugagagagagcacCc...	2	1	S01
....................ugacaaugagagagagcacgc...	399	0	S01
....................ugacaCugagagagagcacgc...	1	1	S01
....................ugacaauUagagagagcacgc...	1	1	S01
....................ugacaaugGgagagagcacgc...	1	1	S01
....................ugacaaugUgagagagcacgc...	2	1	S01
....................ugacaaugagagagagcaUgc...	2	1	S01
....................ugacaaCgagagagagcacgc...	1	1	S01
....................ugacaaugagagagCgcacgc...	1	1	S01
....................ugacGaugagagagagcacgc...	2	1	S01
....................ugacaaugagagagagcGcgc...	1	1	S01
....................ugacaGugagagagagcacgc...	1	1	S01
....................ugacaaugagaCagagcacgc...	1	1	S01
....................ugacaaugagagagagUacgc...	1	1	S01
....................ugacaaugagagagagcacgcuuU..	2	1	S01
.....................gacaaugagagagagcacgU...	1	1	S01
......................acGaugagagagagcacg..	1	1	S01
......................acaaugagagagagcacg..	1	0	S01
......................acaaugagagagagcacgc...	1	0	S01
......................acaaugagagagagcacgcu..	2	0	S01
......................acaaugagagagagcacgcuu...	9	0	S01
......................acaGugagagagagcacgcuu...	1	1	S01
......................acaaugagagagagcacgcuC...	3	1	S01
......................acaaugagagagagcacgcuA...	1	1	S01
.......................caaugagagagagcacgcu..	1	0	S01
.......................caaugagagagagcacgcuu...	1	0	S01
.......................caaugagagagagcacgcuuA..	1	1	S01
.......................caaugagagagagcUcgcuug..	1	1	S01
.......................caaugagagagagcacgcuug..	19	0	S01
.......................caaugagGgagagcacgcuug..	1	1	S01
.......................caaugagagagagcacgcuuU..	1	1	S01
............................agagagagcacgcuCguugguagu..	1	1	S01
.....................................acgcuuguugguagucauuU...	2	1	S01
....uucaaugagauauggcug......................................	2	0	S01
....uauggcugcauacaagcgugc.........................	1	0	S01
....uauggcugcauacaagcgugU.........................	1	1	S01
....uauggcugcauacaGgcgugc.........................	1	1	S01
....ugcauacaagcgugcucuaucucg................	2	0	S01
....caagcgugcCcuaucucg................	1	1	S01
....caagcguAcucuaucucguuguc...........	1	1	S01
....agcgugcucuaucucguuguc...........	2	0	S01
....gcgugcucuaucucgCuguc...........	1	1	S01
....gcgugcucuaucucCuuguc...........	1	1	S01
....gcgugcucuaucucguuguc...........	27	0	S01
....gcgGgcucuaucucguuguc...........	1	1	S01
....gcgugcucuaucucguuguU...........	1	1	S01
....gcgugcucuaucucguuUuc...........	2	1	S01
....gcgugcucuaucucguuUuca..........	1	1	S01
....gcgugcucuUucucguuguca..........	1	1	S01
....gcgugcucuaucucguuguca..........	25	0	S01
....gcgugcucuaucucguugucU..........	2	1	S01
....gcgugcucuaCcucguuguca..........	2	1	S01
....cgugcucuaucucguuguc...........	1	0	S01
....cgugcAcuaucucguuguca..........	1	1	S01
....gugcucuaucucguuguU...........	1	1	S01
....gugcucuaucucguuguc...........	9	0	S01
....gugcucuaucucguuguca..........	4	0	S01
....gugcucuaucucguuUucau.........	1	1	S01
....gugcucuaucucguugucau.........	18	0	S01

```
                   Mature                                                          Star
agacuugaugcaucauuauuugacaaugagagagagcacgcuuguugguagucauucaaugagauauggcugcauacaagcgugcucuaucucguugucauaugauuugc
.................................................................................gugcucuaucucguugucaA.........  1    1   S01
.................................................................................gugcucuaucucguugucCu.........  1    1   S01
.................................................................................gugcuUuaucucguugucau.........  1    1   S01
.................................................................................gugcucuaucucguugucaC.........  1    1   S01
.................................................................................gugcucuaucucguugCcaua........  2    1   S01
.................................................................................gugcucuaucucguugucaua........  51   0   S01
.................................................................................gugcucuaucucguugucauC........  1    1   S01
.................................................................................gugcucuaucucguugucaAa........  1    1   S01
.................................................................................gugcucuGucucguugucaua........  1    1   S01
.................................................................................gugcucuaucucguugucauaU.......  2    1   S01
..................................................................................ugcucuaucAcguugucau.........  1    1   S01
..................................................................................ugcucuaucucguugucau.........  6    0   S01
..................................................................................uCcucuaucucguugucau.........  1    1   S01
..................................................................................ugcucuaucucguugucaua........  10   0   S01
..................................................................................ugcucuaucucguugucauau.......  3    0   S01
..................................................................................ugcucuaucucguugucauaC.......  1    1   S01
..................................................................................ugcucuaucucguugucauaug......  1    0   S01
..................................................................................ugcucuaucucguugucauauC......  1    1   S01
..................................................................................ugcucuaucucguugucauaugau....  1    0   S01
..................................................................................ugcucuaucucguugucauaugaC....  1    1   S01
...................................................................................gcucuaucucguugucauaug......  1    0   S01
.....................................................................................ucuaucucguugucauaugau....  2    0   S01

...................uugacaaugagagGgagc.........................................................................  1    1   S02
...................uugacaaugagagagagA.........................................................................  1    1   S02
...................uugacaaugagagagagU.........................................................................  5    1   S02
...................uugacaaugagagagagc.........................................................................  12   0   S02
...................uugacaaugagagagagcU........................................................................  1    1   S02
...................uugacaaugagagagagca........................................................................  2    0   S02
...................uugacaaugGgagagagcacg......................................................................  1    1   S02
...................uugacaaugagagagagcacg......................................................................  43   0   S02
...................uugacaaugagagagagUacg......................................................................  1    1   S02
...................uugGcaaugagagagagcacg......................................................................  1    1   S02
...................uugacaaCgagagagagcacg......................................................................  1    1   S02
...................uugacaaugagGgagagcacg......................................................................  1    1   S02
...................uugacaaugagagagagcacA......................................................................  5    1   S02
...................uCgacaaugagagagagcacg......................................................................  1    1   S02
...................uugacGaugagagagagcacgc.....................................................................  1    1   S02
...................uugacaauCagagagagcacgc.....................................................................  1    1   S02
...................uugacaaugagagagagcaUgc.....................................................................  1    1   S02
...................uugacUaugagagagagcacgc.....................................................................  1    1   S02
...................uugacaaugaCagagagcacgc.....................................................................  1    1   S02
...................uugacaaugagagagagcacgc.....................................................................  63   0   S02
...................uugGcaaugagagagagcacgc.....................................................................  1    1   S02
...................uugacaaugagagagagcacgA.....................................................................  1    1   S02
...................uugacaaugagagagagcacgU.....................................................................  10   1   S02
...................uugacaaugagagUgagcacgc.....................................................................  1    1   S02
...................uugacaaugagagagagcacgcCu...................................................................  1    1   S02
....................ugacaaugagagagagcG........................................................................  3    1   S02
....................ugacaaugagagagagca........................................................................  8    0   S02
....................ugacaaugagagagagcaA.......................................................................  1    1   S02
....................ugacaaugagagagagcaU.......................................................................  1    1   S02
....................ugacaaugagagagagcac.......................................................................  1    0   S02
....................ugacaaugagagagagcacg......................................................................  7    0   S02
....................ugacaaugagagagagcacA......................................................................  1    1   S02
....................uAacaaugagagagagcacgc.....................................................................  1    1   S02
....................ugacUaugagagagagcacgc.....................................................................  1    1   S02
....................ugacaaugagagagagcacgU.....................................................................  34   1   S02
....................ugacaaugagagagagcacgG.....................................................................  1    1   S02
....................ugacaaugagagagagcacgc.....................................................................  225  0   S02
....................ugGcaaugagagagagcacgc.....................................................................  1    1   S02
....................ugacGaugagagagagcacgc.....................................................................  1    1   S02
....................ugacaaugagagagagcaGgc.....................................................................  2    1   S02
....................ugacaaugagaAagagcacgc.....................................................................  2    1   S02
....................ugacaaugaAagagagcacgc.....................................................................  2    1   S02
....................ugacaaugagagagagcaUgc.....................................................................  1    1   S02
....................ugacaaugagagagaUcacgc.....................................................................  1    1   S02
....................ugacaaugagagagagcaAgc.....................................................................  2    1   S02
....................ugacaaugagagagagUacgc.....................................................................  1    1   S02
....................ugacaaugagagagagcacCc.....................................................................  1    1   S02
```

Mature **Star**

```
agacuugaugcaucauuauuugacaaugagagagagcacgcuuguugguagucauucaaugagauauggcugcauacaagcgugcucuaucucguugucauaugauuugc
....................ugacaaugagagagagcacgA.....................................................................  10  1  S02
....................ugacaaugagagagCgcacgc.....................................................................  1  1  S02
....................ugacaaugagagagGgcacgc.....................................................................  1  1  S02
....................ugacaaugagagCgagcacgc.....................................................................  1  1  S02
....................ugacaaugGgagagagcacgc.....................................................................  2  1  S02
....................ugacaaugagagagagcacAc.....................................................................  2  1  S02
....................ugacaaugagGgagagcacgc.....................................................................  2  1  S02
....................ugacaaugagagagagcacgcG....................................................................  1  1  S02
....................ugacaaugagagagagcacgcuAg..................................................................  1  1  S02
......................acaaugagagagagcacgc.....................................................................  1  0  S02
......................acaaugagagagagcacgcuu...................................................................  11  0  S02
......................acaaugagagagagcacgcuC...................................................................  3  1  S02
.......................caaugagagagagcacgcuug..................................................................  10  0  S02
.......................caaugaCagagagcacgcuug..................................................................  1  1  S02
.......................caaGgagagagagcacgcuug..................................................................  1  1  S02
.......................caaugagUgagagcacgcuug..................................................................  1  1  S02
.......................caaugagagagagcacgcuCg..................................................................  1  1  S02
.......................caaugagagUgagcacgcuug..................................................................  1  1  S02
.....................................acgcuuguugguagucauuU.....................................................  1  1  S02
......................................................uucaaugagauauggcug......................................  6  0  S02
...............................................................auauggcugcauacaagc.............................  1  0  S02
...............................................................................gcgugcucuaucucguuguc...........  3  0  S02
...............................................................................gcgugcucuauUucguuguc...........  1  1  S02
...............................................................................gcgugcCcuaucucguuguc...........  1  1  S02
...............................................................................gcgugcucuaucucguuguca..........  7  0  S02
...............................................................................gcgugcucuaucucguugucC..........  1  1  S02
...............................................................................gcgugcucuaucucguugucU..........  2  1  S02
................................................................................cgugcucuaucucguugucauauU......  1  1  S02
.................................................................................gugcucuaucucguuguc...........  3  0  S02
.................................................................................gugcucuaucucguuUuc...........  1  1  S02
.................................................................................Cugcucuaucucguuguca..........  1  1  S02
.................................................................................gugcucuaucucguuguca..........  2  0  S02
.................................................................................gugcucuaucucguugucG..........  1  1  S02
.................................................................................gugcucuGucucguugucau.........  1  1  S02
.................................................................................gugcucuauAucguugucau.........  1  1  S02
.................................................................................gugcucuaucucguugucau.........  28  0  S02
.................................................................................gugGucuaucucguugucaua........  1  1  S02
.................................................................................Uugcucuaucucguugucaua........  1  1  S02
.................................................................................gugcucuaCcucguugucaua........  1  1  S02
.................................................................................Augcucuaucucguugucaua........  1  1  S02
.................................................................................gugcucuaucucguugucaua........  38  0  S02
.................................................................................gugcucuaucucguugucauG........  1  1  S02
.................................................................................Cugcucuaucucguugucaua........  1  1  S02
.................................................................................gugcucGaucucguugucaua........  1  1  S02
.................................................................................gugcucuaucucguugucGua........  1  1  S02
..................................................................................Cgcucuaucucguugucau.........  1  1  S02
..................................................................................ugcucuaucucguugucau.........  2  0  S02
..................................................................................ugcucuaucucguugucaua........  2  0  S02
..................................................................................ugcucuaucucguugucauau.......  1  0  S02
..................................................................................ugcucuaucucguugucauaugau....  1  0  S02
.....................................................................................ucuaucucguugucauaugau....  1  0  S02
```

Provisional ID	:	Maple_Unigene_BMK.34899_169283
Score total	:	1.8
Score for star read(s)	:	3.9
Score for read counts	:	3.2
Score for mfe	:	-4.6
Score for randfold	:	0
Score for cons. seed	:	-0.6
Total read count	:	18
Mature read count	:	15
Loop read count	:	2
Star read count	:	1

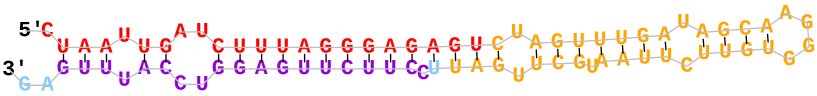

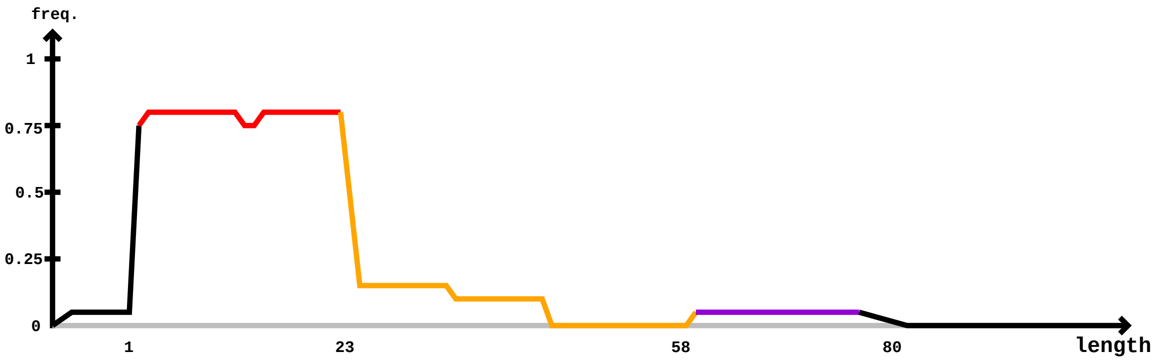

Mature **Star**

```
5'- agaaguccuaauugaucuuuagggagagucuaguuugauagcaagggguguucuuaaugcuugauuccuucuugagguccauuugaguaaaaaguuuaagcguuccuaaua   -3'    obs
    agaaguccuaauugaucuuuagggagagucuaguuugauagcaagggguguucuuaaugcuugauuccuucuugagguccauuugaguaaaaaguuuaagcguuccuaaua          exp
    ...(.((..(((.((((((.(((..(((((.(((((((.((((....)))).)))).))).)))))..))).)))))).))).)).).......................    reads       mm          sample
    .......cuaauugaucuuuaggAagagu.................................................................................    9           1           S01
    ....................agggagagucuaguuugaua......................................................................    1           0           S01
    .................................................................cUuucuugagguccauuug..........................    1           1           S01

    aCaaguccuaauugaucu............................................................................................    1           1           S02
    .......cuaauugaucuuuaggAagagu.................................................................................    5           1           S02
    ........uaauugaucuuuaggAagagu.................................................................................    1           1           S02
    .............................cuaguuugauagcaagggugu............................................................    2           0           S02
```

Provisional ID : Maple_Unigene_BMK.35372_174784
Score total : 408.4
Score for star read(s) : 3.9
Score for read counts : 404.9
Score for mfe : -3.3
Score for randfold : 0
Score for cons. seed : 3
Total read count : 806
Mature read count : 587
Loop read count : 218
Star read count : 1

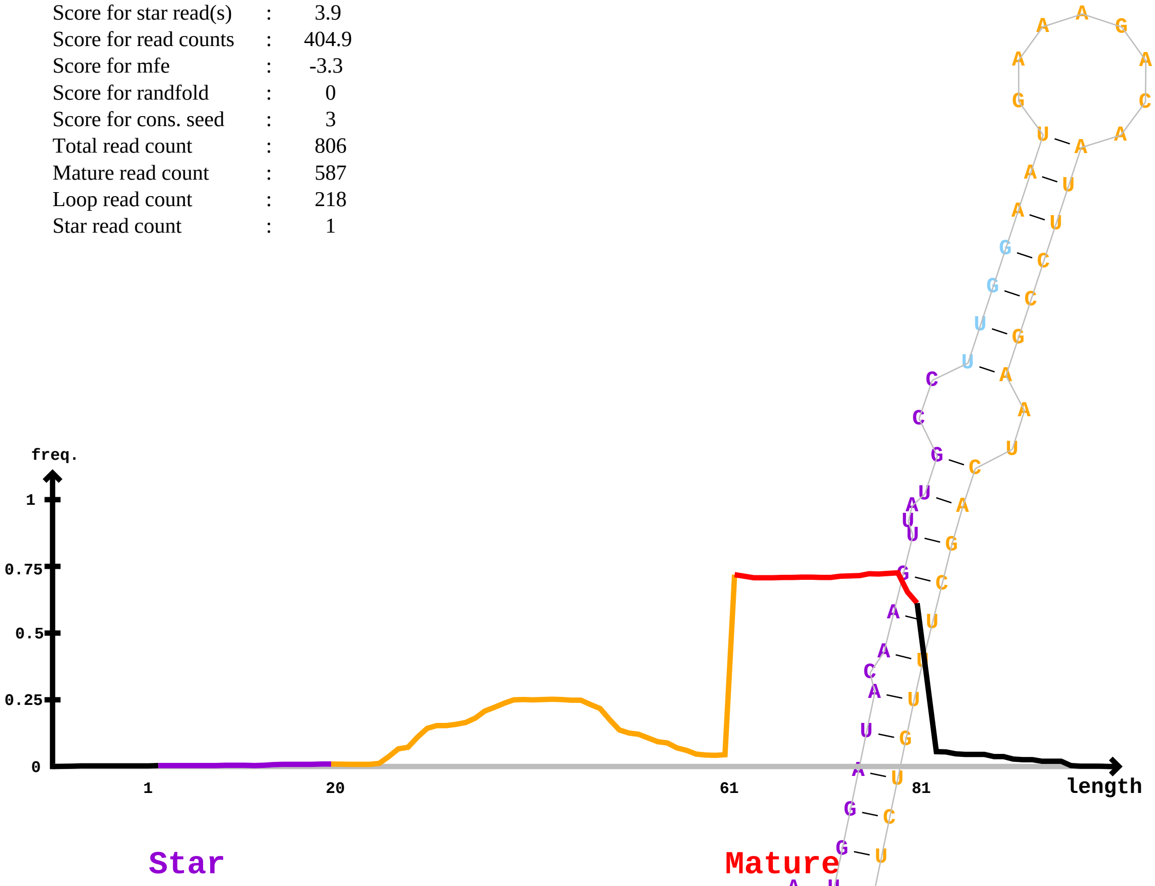


```
5'- uaugaagcgcauggauacaaguuaugccuuggaaugaaagacaauuccgaaucagcuuugucuacgaacaaggaagcuauaaguaaugcaacuaugaaucucauggaga -3'  obs
    uaugaagcgcauggauacaaguuaugccuuggaaugaaagacaauuccgaaucagcuuugucuacgaacaaggaagcuauaaguaaugcaacuaugaaucucauggaga      exp
    ........((((.......((((...(((((.......((((((...(......)..))))))....))))).))))).......))))..(((((.....)))))...      reads  mm  sample
    uaugaagcgcaCggauaca..........................................................................................      1      1   S01
    .augaagcgcauggauacG..........................................................................................      1      1   S01
    .........cauggauacaaguuaugcU.................................................................................      1      1   S01
    .....................uuaugccuuggaaugaaagaU...................................................................      1      1   S01
    .....................uuaugccuuggaaugaaagacaauuU..............................................................      1      1   S01
    ......................uauCccuuggaaugaaaga....................................................................      1      1   S01
    ..........................ccuuggaaugaaagacaauucU.............................................................      1      1   S01
    ................................aaugaaagacaauuccgaauca.......................................................      1      0   S01
    ................................aaugaaagacaauuccgaaucaCc.....................................................      1      1   S01
    ................................aaugaaagacaauuccgaaucagcC....................................................      1      1   S01
    .................................aCgaaagacaauuccgaauc........................................................      1      1   S01
    .................................augaaagacaauuccgaauca.......................................................      1      0   S01
    .................................augaaagacaauuccgaaucag......................................................      2      0   S01
    .................................augaaagacaauuccgaaucagc.....................................................      2      0   S01
    .................................augaaagacaauuccgaaucagcu....................................................      1      0   S01
    .................................augaaagacaauuccgaaucagcC....................................................      1      1   S01
    .................................augaaagacaauuccgaaucagcuu...................................................      1      0   S01
    .................................augaaagacaauuccgaaucagcuuug.................................................      1      0   S01
    ..................................ugaaagacaauuccgaaucag......................................................      1      0   S01
    ..................................ugaaagacaauuccgaaucaA......................................................      1      1   S01
    ..................................ugaaagacaauuccgaaucagA.....................................................      3      1   S01
    ..................................ugaaagacaauuccgaaucagc.....................................................      2      0   S01
    ..................................ugaaagacaauuccgaaucaCc.....................................................      1      1   S01
    ..................................ugaaagacaauuccgaaucagcu....................................................      1      0   S01
    ..................................ugaaagacaauuccgaaucagcG....................................................      1      1   S01
    ..................................ugaaagacaauuccgaaucagcC....................................................      1      1   S01
    ..................................ugaaagacaauuccgaaucagcuu...................................................      2      0   S01
    ...................................gaaagacaauuccgaauca.......................................................      1      0   S01
    ...................................gaaagacaauuccgaaucaCc.....................................................      1      1   S01
    ...................................gaaagacaauuccgaaucagc.....................................................      1      0   S01
    ....................................aaagacaauuccgaauca.......................................................      4      0   S01
    ....................................aaagacaauuccgaaucagc.....................................................      5      0   S01
    ....................................aaagacaauuccgaaucGgc.....................................................      1      1   S01
```

Star **Mature**

```
uaugaagcgcauggauacaaguuaugccuuggaaugaaagacaauuccgaaucagcuuugucuacgaacaaggaagcuauaaguaaugcaacuaugaaucucauggaga
....................................aaagacaauuccgaaucagU.....................................................   1   1   S01
....................................aaagacaauuccgaaucagcu....................................................   1   0   S01
....................................Caagacaauuccgaaucagcu....................................................   1   1   S01
....................................aaagacaauuccgaaucagcC....................................................   3   1   S01
....................................aaagacaauuccgaaucagcuuug.................................................   1   0   S01
.....................................aagacaauuccgaaucag......................................................   3   0   S01
.....................................aagacaauuccgaaucagc.....................................................   1   0   S01
.....................................aagacaauuccgaaucagU.....................................................   2   1   S01
.....................................aagacaauuccgaaucagcA....................................................   1   1   S01
.....................................aagacaauuccgaaucagcu....................................................   4   0   S01
.....................................aagacaauuccgaaucagcC....................................................   1   1   S01
.....................................aagacaauuccgaaucagcuA...................................................   1   1   S01
.....................................aagacaauuccgaaucagcuC...................................................   1   1   S01
.....................................aagacaauuccgaaucagcuuu..................................................   1   0   S01
.....................................aagacaauuccgaaucagcuuug.................................................   1   0   S01
.....................................aagacaauuccgaaucagcuuugu................................................   2   0   S01
......................................agacaauuccgaaucagc.....................................................   2   0   S01
......................................agacaauuccgaaucagcu....................................................   1   0   S01
......................................agacaauuccgaaucagcuu...................................................   1   0   S01
........................................acaauuccgaaucagcuuugA................................................   1   1   S01
.........................................caauuccgaaucagcuuu..................................................   1   0   S01
.........................................caauuccgaaucagcuuugucu..............................................   1   0   S01
..........................................aauuccgaaucagcuuug.................................................   1   0   S01
..........................................aauuccgaaucagcuuugu................................................   1   0   S01
..........................................aauuccgaaucagcuuuguc...............................................   1   0   S01
..........................................aauuccgaaucagcuuugucuac............................................   1   0   S01
..........................................aauuccgaaucagcuuugucuaU............................................   1   1   S01
..........................................aauuccgaaucagcuuugucuacU...........................................   1   1   S01
..........................................aauuccgaaucagcuuugucuacga..........................................   1   0   S01
..........................................aauuccgaaucagcuuugucuacgaac........................................   1   0   S01
...........................................auuccgaaucagcuuugu................................................   1   0   S01
...........................................auuccgaaucagcuuuguc...............................................   1   0   S01
...........................................auuccgaaucagcuuugucC..............................................   1   1   S01
...........................................auuccgaaucagcuuugucu..............................................   3   0   S01
...........................................auuccgaaucagcuuugucua.............................................   1   0   S01
...........................................auuccgaaucagcuuugucuaU............................................   1   1   S01
...........................................auuccgaaucagcuuugucuacC...........................................   1   1   S01
...........................................Guuccgaaucagcuuugucuacgaac........................................   1   1   S01
...........................................auuccgaaucagcuuugucuacgaac........................................   1   0   S01
............................................uuccgaaucagcuuuguc...............................................   1   0   S01
............................................uuccgaaucagcuuugucC..............................................   1   1   S01
............................................uuccgaaucagcuuugucua.............................................   2   0   S01
............................................uuccgaaucagcuuugucuac............................................   1   0   S01
............................................uuccgaaucagcuuugucuacgaac........................................   1   0   S01
.............................................uccgaaucagcuuugucu..............................................   4   0   S01
.............................................uccgaaucagcuuugucuac............................................   2   0   S01
.............................................uccgaaucagcuuugucuacg...........................................   1   0   S01
.............................................uccgaaucagcuuugucuacgaac........................................   1   0   S01
.............................................uccgaaucagcuuugucuacgaaca.......................................   2   0   S01
..............................................ccgaaucagcuuugucua.............................................   2   0   S01
..............................................ccgaaucagcuuugucuacgaaca.......................................   1   0   S01
..............................................ccgaaucagcuuugucuacgaacaa......................................   2   0   S01
...............................................cgaaucagcuuugucuacgaacaa......................................   1   0   S01
.....................................................agcuuugucuacgaacaa......................................   1   0   S01
..............................................................uacgaacaaggaagcuauaagC.........................   1   1   S01
................................................................cgaacaaggaagcuauaaguaaC......................   1   1   S01
...................................................................acaagAaagcuauaagua........................   1   1   S01
....................................................................caaggaagcuauaaguaaugA....................   1   1   S01
.....................................................................aaUgaagcuauaaguaau......................   1   1   S01
.....................................................................Gaggaagcuauaaguaau......................   1   1   S01
.....................................................................aaggaagcuauaaAuaau......................   1   1   S01
.....................................................................aaggaagcuauaaguaaA......................   2   1   S01
.....................................................................aaggaagcuauaaguaUu......................   2   1   S01
.....................................................................aaggaagcuauaGguaau......................   1   1   S01
.....................................................................aaggaagcuauaaguaaC......................   24  1   S01
.....................................................................aaggaagAuauaaguaau......................   1   1   S01
.....................................................................aaggaagcuauaagCaau......................   1   1   S01
.....................................................................aaggaagcuauaagGaau......................   2   1   S01
.....................................................................aaggaagcCauaaguaau......................   1   1   S01
.....................................................................aUggaagcuauaaguaau......................   1   1   S01
```

Star **Mature**

```
uaugaagcgcauggauacaaguuaugccuuggaaugaaagacaauuccgaaucagcuuugucuacgaacaaggaagcuauaaguaaugcaacuaugaaucucauggaga
.....................................................................aaggaaAcuauaaguaau......................   1     1    S01
.....................................................................aagUaagcuauaaguaau......................   1     1    S01
.....................................................................Uaggaagcuauaaguaau......................   1     1    S01
.....................................................................aagAaagcuauaaguaau......................   1     1    S01
.....................................................................aaggaagcuUuaaguaau......................   1     1    S01
.....................................................................aGggaagcuauaaguaau......................   2     1    S01
.....................................................................aaggaCgcuauaaguaaug.....................   1     1    S01
.....................................................................Gaggaagcuauaaguaaug.....................   1     1    S01
.....................................................................aaggaagcuauaaguaauA.....................   12    1    S01
.....................................................................aaggaagcuauGaguaaug.....................   1     1    S01
.....................................................................aaggaagcuauaaguGaug.....................   2     1    S01
.....................................................................aaggaagcuGuaaguaaug.....................   1     1    S01
.....................................................................aaggaagcuauaaguaaCg.....................   2     1    S01
.....................................................................aaggaagGuauaaguaaug.....................   1     1    S01
.....................................................................aaggaaAcuauaaguaaug.....................   1     1    S01
.....................................................................aaggaagcuauaaguaCug.....................   1     1    S01
.....................................................................aaggaGgcuauaaguaaugc....................   1     1    S01
.....................................................................aaggaagcGauaaguaaugc....................   1     1    S01
.....................................................................aaggCagcuauaaguaaugc....................   1     1    S01
.....................................................................aaggaagcCauaaguaaugc....................   1     1    S01
.....................................................................aaggaagcuCuaaguaaugc....................   1     1    S01
.....................................................................aaggaagcuauaaguaaugU....................   79    1    S01
.....................................................................aaggaaAcuauaaguaaugc....................   1     1    S01
.....................................................................aGggaagcuauaaguaaugc....................   1     1    S01
.....................................................................aaggaagAuauaaguaaugc....................   1     1    S01
.....................................................................Gaggaagcuauaaguaaugc....................   2     1    S01
.....................................................................aaggaagGuauaaguaaugc....................   1     1    S01
.....................................................................aaggaagcuauaaguaaCgc....................   2     1    S01
.....................................................................aaggaagcuaCaaguaaugc....................   1     1    S01
.....................................................................aaggaagcuauaaguaaugA....................   285   1    S01
.....................................................................aaggaagcuGuaaguaaugc....................   1     1    S01
.....................................................................aaggaagcuauaaguGaugc....................   2     1    S01
.....................................................................aaUgaagcuauaaguaaugc....................   1     1    S01
.....................................................................aaggaagcuauaGguaaugc....................   1     1    S01
.....................................................................Naggaagcuauaaguaaugc....................   3     1    S01
.....................................................................aaggaagcuauaaguaaugG....................   5     1    S01
.....................................................................aagUaagcuauaaguaaugc....................   5     1    S01
.....................................................................aaggaagcuauaCguaaugc....................   2     1    S01
.....................................................................aaggaagcuauaaguaaAgc....................   1     1    S01
.....................................................................aaggaagcuauaaguaGugca...................   1     1    S01
.....................................................................aaggaagcuauaaguaaugcG...................   1     1    S01
.....................................................................aaggaagcuauaaguaaugAa...................   16    1    S01
.....................................................................aaggaagcuauaaguaaugAaa..................   1     1    S01
.....................................................................aaggaagcuauaaguGaugcaac.................   1     1    S01
.....................................................................aaggaagcuauaGguaaugcaac.................   1     1    S01
.....................................................................aaggaagcuauaaguaGugcaac.................   1     1    S01
.....................................................................aGggaagcuauaaguaaugcaacuaug.............   1     1    S01
.....................................................................aaggaagcuauaaguaaugcaGcuaug.............   1     1    S01
.....................................................................aaggaagcuauaaguaaugcaacuaCg.............   2     1    S01
.....................................................................aaggaagcuauaaguaaugcaacuauA.............   1     1    S01
.....................................................................aaggaagcuauaaguaUugcaacuaugaa...........   1     1    S01
........................................................................gaagcuauaaguaaugcaacuaugaaC..........   1     1    S01
............................................................................cuauaaguaaugcaacuaugaaC..........   1     1    S01
..............................................................................auaaguaauAcaacuaugaaucucau.....   1     1    S01
................................................................................aaguaaugcaacuaugaGucu........   1     1    S01
................................................................................aaguaaugcGacuaugaaucu........   1     1    S01
.................................................................................agGaaugcaacuaugaaucu........   1     1    S01
..................................................................................guaaugcaacuaugaaucucaC.....   1     1    S01
...................................................................................uaaugcaacuaugaaucC........   1     1    S01
...................................................................................uaaugcaacuaugaaucucaG.....   1     1    S01
...................................................................................uaaugcaacuaugaaucucaC.....   2     1    S01
.....................................................................................augcaacuaugaaucucaC.....   1     1    S01
......................................................................................ugcaacuaugaaucucaC.....   1     1    S01
.......................................................................................gcaacuaugaaucucauU....   1     1    S01
.......................................................................................gcaacuaugaaucucauggaA.   1     1    S01

................acaaguuaugccuuggaaugGaagac...................................................................   1     1    S02
...................aguuaugccuuggaaugaGagac...................................................................   1     1    S02
....................guuaugccuugCaaugaaa......................................................................   1     1    S02
.................................augaaagacaGuuccgaa..........................................................   1     1    S02
```

Star Mature

```
uaugaagcgcauggauacaaguuaugccuuggaaugaaagacaauuccgaaucagcuuugucuacgaacaaggaagcuauaaguaaugcaacuaugaaucucauggaga
.................................augaaagacaauuccgaaA.........................................................   1   1   S02
.................................augaaagacaauuccgaaC.........................................................   1   1   S02
.................................augaaagacaauuccgaauca.......................................................   2   0   S02
.................................augaaagacaauuccgaaucag......................................................   2   0   S02
.................................augaaagacaauuccgaaucagc.....................................................   1   0   S02
.................................augaaagacaauuccgaaucagcu....................................................   1   0   S02
.................................augaaagacaauuccgaaucagcuu...................................................   1   0   S02
.................................augaaagacaauuccgaaucagcuuug.................................................   2   0   S02
..................................ugaaagacaauuccgaaC.........................................................   1   1   S02
..................................ugaaagacaauuccgaauca.......................................................   2   0   S02
..................................ugaaagacaauuccgaaucag......................................................   1   0   S02
..................................ugaaagacaauuccgaaucagc.....................................................   2   0   S02
..................................ugaaagacaauuccgaauUagcu....................................................   1   1   S02
..................................ugaaagacaauuccgaaucagcG....................................................   2   1   S02
..................................ugaaagacaauuccgaaucagcu....................................................   2   0   S02
..................................ugaaagacaauuccgaaucagcuuug.................................................   1   0   S02
...................................gaaagacaauuccgaauca.......................................................   1   0   S02
...................................gaaagacaauuccgaaucagcC....................................................   1   1   S02
....................................aaagacaauuccgaauca.......................................................   2   0   S02
....................................aaagacaauuccgaaucag......................................................   2   0   S02
....................................aaagacaauuccgaaucagU.....................................................   1   1   S02
....................................aaagacaauuccgaaucagc.....................................................   2   0   S02
....................................aaagacaauuccgaaucagcu....................................................   4   0   S02
....................................aaagacaauuccgaaucagcuG...................................................   1   1   S02
....................................Gaagacaauuccgaaucagcuu...................................................   1   1   S02
....................................aaagacaauuccgaaucagcuu...................................................   2   0   S02
....................................aaagacaauuccgaaucagcuuug.................................................   1   0   S02
.....................................aGgacaauuccgaaucag......................................................   1   1   S02
.....................................aagacaauuccgaaucagc.....................................................   4   0   S02
.....................................aagacaauuccgaaucagcu....................................................   1   0   S02
.....................................aagacaauuccgaauUagcu....................................................   1   1   S02
.....................................aagacaauuccgaaucagcuuu..................................................   2   0   S02
.....................................aagacaauuccgaaucagcuuug.................................................   1   0   S02
......................................agacaauuccgaaucagc.....................................................   2   0   S02
......................................agacaCuuccgaaucagc.....................................................   1   1   S02
......................................agacaauuccgaaucagcu....................................................   1   0   S02
......................................agacaauuccgaaucagcuuuU.................................................   1   1   S02
.......................................gacaauuccgaaucagcu....................................................   1   0   S02
........................................acaauuccgaaucagcuuug.................................................   2   0   S02
........................................acaauuccgaauUagcuuugucuac............................................   1   1   S02
.........................................caauuccgaaucagcuuugucC..............................................   1   1   S02
.........................................caauuccgaaucagcuuugucu..............................................   1   0   S02
.........................................caauuccgaaucagcuuugucua.............................................   2   0   S02
.........................................caauuccgaaucagcuuugucuacgaac........................................   1   0   S02
..........................................aauuccgaaucagcuuug.................................................   1   0   S02
..........................................aauuccgaauUagcuuugu................................................   1   1   S02
..........................................aauuccgaaucagcuuugu................................................   1   0   S02
..........................................aauuccgaaucagcuuugG................................................   1   1   S02
..........................................aauuccgaaucagcuuuguc...............................................   1   0   S02
..........................................aauuccgaaucagcuuugucu..............................................   2   0   S02
..........................................aauuccgaaucagcuuugucua.............................................   1   0   S02
..........................................aauuccgaaucagcuuugucuac............................................   1   0   S02
...........................................auuccgaaucagcuuugu................................................   3   0   S02
...........................................auuccgaaucagcuuugC................................................   1   1   S02
...........................................auuccgaaucagcuuugucG..............................................   1   1   S02
...........................................auuccgaaucagcuuugucuaU............................................   1   1   S02
...........................................auuccgaaucagcuuugucuac............................................   2   0   S02
...........................................auuccgaaucagcuuugucuacga..........................................   1   0   S02
...........................................auuccgaaucagcuuugucuacgaac........................................   3   0   S02
............................................uuccgaaucagcuuuguc...............................................   1   0   S02
............................................uuccUaaucagcuuugucu..............................................   1   1   S02
............................................uuccgaaucagcuuugucuacgaac........................................   2   0   S02
............................................uuccgaaucagcuuugucuacgaaU........................................   1   1   S02
............................................uuccgaaucagcuuugucuacgaaca.......................................   1   0   S02
.............................................uccgaaucagcuuugucu..............................................   1   0   S02
.............................................uccgaaucagcuuugucCa.............................................   1   1   S02
.............................................uccgaaucagcuuugucua.............................................   1   0   S02
..............................................ccgaaucagcuuugucuac............................................   1   0   S02
..............................................ccgaaucagcuuugucuacgaac........................................   4   0   S02
..............................................ccgaaucagGuuugucuacgaaca.......................................   1   1   S02
```

Star   Mature

```
uaugaagcgcauggauacaaguuaugccuuggaaugaaagacaauuccgaaucagcuuugucuacgaacaaggaagcuauaaguaaugcaacuaugaaucucauggaga
...............................................cgaaucagcuuugucuacgaaU........................................   1   1   S02
.................................................aaucagcuuugucuacgaacaag.....................................   1   0   S02
..................................................auUagcuuugucuacgaacaaggaagcu...............................   1   1   S02
....................................................cagcuuuguUuacgaacaa......................................   1   1   S02
...........................................................ugucuacgaacaaggaagcC..............................   1   1   S02
.............................................................ucuacgaacaaggaagcuauaagC........................   1   1   S02
...............................................................acgaacaaggaUgcuauaaguaau......................   1   1   S02
................................................................cgaacaaggaagcuauaagC.........................   1   1   S02
....................................................................cGaggaagcuauaaguaaug.....................   1   1   S02
.....................................................................aaggGagcuauaaguaau......................   1   1   S02
.....................................................................aaCgaagcuauaaguaau......................   1   1   S02
.....................................................................aaggaagcuGuaaguaau......................   1   1   S02
.....................................................................aaggaagcuauGaguaau......................   1   1   S02
.....................................................................aaggaagcuauaaguaaC......................   3   1   S02
.....................................................................aagUaagcuauaaguaau......................   1   1   S02
.....................................................................aagCaagcuauaaguaau......................   1   1   S02
.....................................................................aGggaagcuauaaguaau......................   1   1   S02
.....................................................................aaggaagcuauaaguaaA......................   1   1   S02
.....................................................................aaggaagcuauaaguaGu......................   2   1   S02
.....................................................................aaggaGgcuauaaguaau......................   1   1   S02
.....................................................................aaggaagcGauaaguaau......................   1   1   S02
.....................................................................aaggaagcuauaaguaaG......................   1   1   S02
.....................................................................aaggaagGuauaaguaau......................   1   1   S02
.....................................................................aagCaagcuauaaguaaug.....................   1   1   S02
.....................................................................aaggaagcuauaCguaaug.....................   1   1   S02
.....................................................................Naggaagcuauaaguaaug.....................   1   1   S02
.....................................................................aaggaagcuauCaguaaug.....................   1   1   S02
.....................................................................aagUaagcuauaaguaaug.....................   1   1   S02
.....................................................................aaggaGgcuauaaguaaug.....................   1   1   S02
.....................................................................aaggaagcuauaaguaaCg.....................   2   1   S02
.....................................................................aaggaagcuauaaguaaGg.....................   1   1   S02
.....................................................................aaggaagcuauaaguaauA.....................   3   1   S02
.....................................................................aaggaagAuauaaguaaugc....................   1   1   S02
.....................................................................Naggaagcuauaaguaaugc....................   1   1   S02
.....................................................................aaggaagcuauaaguaaAgc....................   1   1   S02
.....................................................................aaggaagcuauaaguaaugA....................   38  1   S02
.....................................................................aaggaagcuauaagAaaugc....................   1   1   S02
.....................................................................aaggaagcuauaaguaaugU....................   7   1   S02
.....................................................................aaggaagcuauaaguaaugG....................   4   1   S02
.....................................................................aaggaagcuauaaguaaugcG...................   2   1   S02
.....................................................................aaggaagcuGuaaguaaugca...................   1   1   S02
.....................................................................aaggaagcuauaaguaaugcC...................   1   1   S02
.....................................................................aaggaagcuauaaguaaugAa...................   4   1   S02
.....................................................................aagAaagcuauaaguaaugcaac.................   1   1   S02
.....................................................................aaggaagcuauaaguaaugcaaU.................   2   1   S02
.....................................................................aaggaagcuauaaguaaugcaacG................   1   1   S02
.....................................................................aaggaagcuaAaaguaaugcaacu................   1   1   S02
.....................................................................aaggaagcuauaagCaaugcaacuaug.............   1   1   S02
.....................................................................aaggaagcuauaaguaaugcaacuauA.............   1   1   S02
.....................................................................aagUaagcuauaaguaaugcaacuaugaa...........   1   1   S02
.....................................................................aaggaagcuauaaguaaugcaacuaugaG...........   2   1   S02
.....................................................................aaggaagcuauaaguaaugcaacuaugGa...........   1   1   S02
.....................................................................aaggaagcuUuaaguaaugcaacuaugaa...........   1   1   S02
.....................................................................aGggaagcuauaaguaaugcaacuaugaa...........   1   1   S02
..........................................................................agcuauaaguaaugcGacuaugaa...........   1   1   S02
................................................................................aaguaaugcaacuaugaaucG........   1   1   S02
................................................................................aaguaaugcaacuauAaaucucau.....   1   1   S02
...................................................................................uaaugcaacuaugaaucucGu.....   1   1   S02
...................................................................................uaaugcaacuaugaaucucaC.....   1   1   S02
....................................................................................aaugcaacuaugaaucucaC.....   1   1   S02
....................................................................................aaugcGacuaugaaucucaug....   1   1   S02
.....................................................................................augcGacuaugaaucucau.....   1   1   S02
.....................................................................................augcaacuaugaaucucaG.....   1   1   S02
......................................................................................ugcaacuaugaaucucaC.....   1   1   S02
```

Provisional ID	:	Maple_Unigene_BMK.35383_174954
Score total	:	0
Score for star read(s)	:	3.9
Score for read counts	:	-4.9
Score for mfe	:	-1.8
Score for randfold	:	0
Score for cons. seed	:	3
Total read count	:	2
Mature read count	:	1
Loop read count	:	0
Star read count	:	1

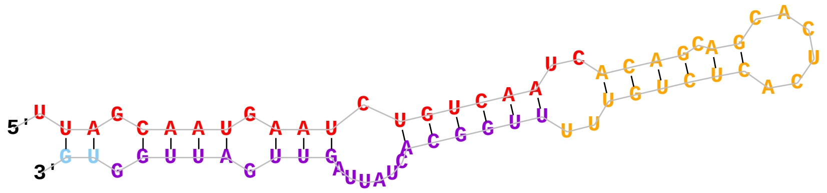

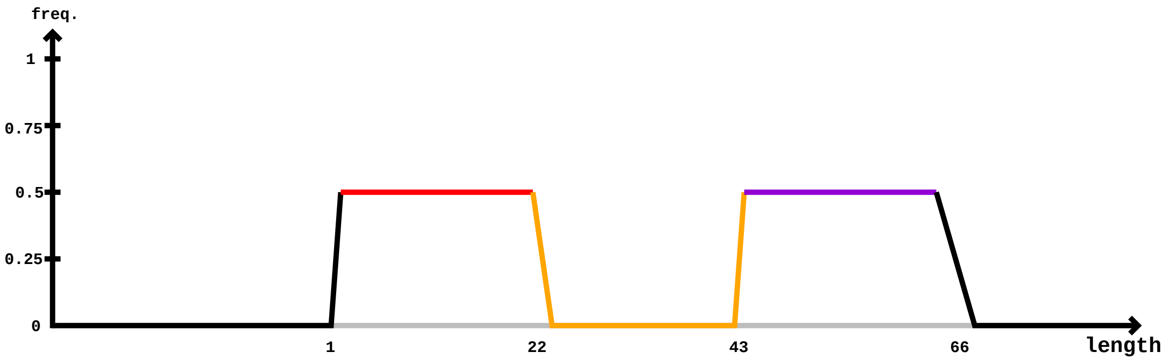


```
                                Mature                                      Star
5'- uuguggucaugguugccuuggcuucauguuagcaaugaaucugucaaucacagcagcacucacucuguuuuuggcacuauuaguugauugguguauuuaguaaagguggau   -3'    obs
    uuguggucaugguugccuuggcuucauguuagcaaugaaucugucaaucacagcagcacucacucuguuuuuggcacuauuaguugauugguguauuuaguaaagguggau          exp
    ...........(..((((.(((..(..((((((((.(((.((((((..((((.((......))))))..))))))..))).)))).)))).)..)....))).)))).)..   reads   mm   sample
    ...........................uuagcaaugaaucugucaauc.................................................................   1       0    S02
    ...............................................................uuggcacuauuaguugauugg...................   1       0    S02
```

Provisional ID	:	Maple_Unigene_BMK.35734_178434
Score total	:	2.8
Score for star read(s)	:	3.9
Score for read counts	:	-4.9
Score for mfe	:	0.9
Score for randfold	:	0
Score for cons. seed	:	3
Total read count	:	2
Mature read count	:	1
Loop read count	:	0
Star read count	:	1

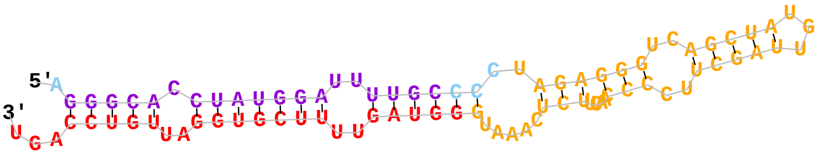

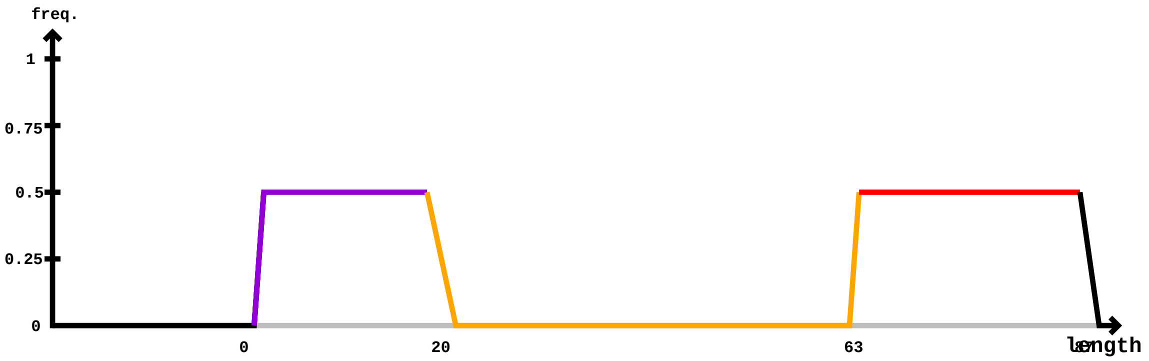


```
                         Star                                                              Mature
5'- caugaauucuucuuccagaagggcaccuauggauuuugccccuagagggucagcuauguuagcuuccccaacuucucaaaugggguaguuuucguggauuguccagucag  -3'   obs
    caugaauucuucuuccagaagggcaccuauggauuuugccccuagagggucagcuauguuagcuuccccaacuucucaaauggguaguuuucguggauuguccagucag        exp
    ..(((..(((......))).(((((.(((((((..((((((..((((((..(((((...)))))..)))....)))....))))))..)))))))..)))))..))).   reads   mm   sample
    ....................gggcaccuauggauuuugc......................................................................   1       0    S02
    ..................................................................................gguaguuuucguggauuguccagu...   1       0    S02
```

Provisional ID	:	Maple_Unigene_BMK.36373_187147
Score total	:	58.7
Score for star read(s)	:	3.9
Score for read counts	:	62.8
Score for mfe	:	-10.9
Score for randfold	:	0
Score for cons. seed	:	3
Total read count	:	135
Mature read count	:	123
Loop read count	:	0
Star read count	:	12

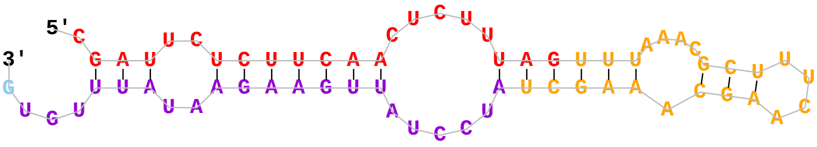

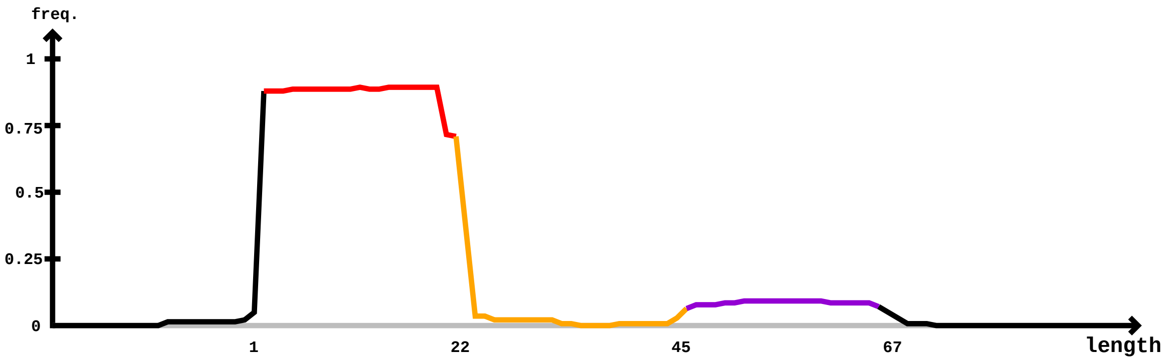

Mature **Star**

```
5'- cuuaaagaauuaaauuaucacgauucucuucaacucuuuaguuuaaacgcuuucaagcaaagcuauccuauugaagaauauuugugccuuucaaaucagauucucucuuau  -3'   obs
    cuuaaagaauuaaauuaucacgauucucuucaacucuuuaguuuaaacgcuuucaagcaaagcuauccuauugaagaauauuugugccuuucaaaucagauucucucuuau         exp
    .....((((((..(((..(((((...(((((((.....((((((....(((....))).)))))).....)))))))....))))).......)))..)))))).......   reads   mm   sample
    ...................acgauucucuucaacucuuuag......................................................................   1       0    S01
    ...................acgauGcucuucaacucuuuagu.....................................................................   1       1    S01
    ....................cgauGcucuucaacucuuu........................................................................   1       1    S01
    ....................cgauucucuucaacucuuu........................................................................   7       0    S01
    ....................cgauucucuucaacucuuuag......................................................................   24      0    S01
    ....................cgauGcucuucaacucuuuag......................................................................   6       1    S01
    ....................cgauucucCucaacucuuuag......................................................................   1       1    S01
    ....................cgauGcucuucaacucuuuagu.....................................................................   1       1    S01
    ....................cgauucucuucaacucuuuaguuu...................................................................   1       0    S01
    ..............................caacucuuuaguuuaaacgcu............................................................   1       0    S01
    ................................acucuuuaguuuaaacgcuuC..........................................................   1       1    S01
    .................................cucuuuaguuuaaacgcu............................................................   1       0    S01
    .........................................................caaagcuauccuauugaagaau................................   1       0    S01
    ................................................................auccuauugaagaauauuugu..........................   1       0    S01
    .................................................................uccuauugaagaauauuugug.........................   2       0    S01
    ....................................................................uauugaagaauauuugugc........................   1       0    S01

    ..........uaaauuaucacgauucucuuc................................................................................   1       0    S02
    ..........uaaauuaucacgauucucuuca...............................................................................   1       0    S02
    ..................cacgauGcucuucaacucuuu........................................................................   1       1    S02
    ...................acgauucucuucaacucuuua.......................................................................   1       0    S02
    ...................acgauucucuucaacucuuuag......................................................................   1       0    S02
    ....................cgauucucuucaacucuuu........................................................................   10      0    S02
    ....................cgGuucucuucaacucuuu........................................................................   1       1    S02
    ....................cgauGcucuucaacucuuu........................................................................   4       1    S02
    ....................cCauucucuucaacucuuu........................................................................   1       1    S02
    ....................cgauucucuucaacCcuuuag......................................................................   1       1    S02
    ....................Ugauucucuucaacucuuuag......................................................................   1       1    S02
    ....................cgauucucuucaacucuuuaA......................................................................   1       1    S02
    ....................cgauGcucuucaacucuuuag......................................................................   6       1    S02
    ....................cgauucucuucaacucuuuag......................................................................   50      0    S02
    ....................cgauucucuucaacucuuuagu.....................................................................   1       0    S02
    .......................uucucuucaacucuuuaguuu...................................................................   1       0    S02
```

Mature Star

```
cuuaaagaauuaaauuaucacgauucucuucaacucuuuaguuuaaacgcuuucaagcaaagcuauccuauugaagaauauuugugccuuucaaaucagauucucucuuau
..............................................................uauccuauugaagaauauuug...........................  2  0  S02
..............................................................uauccuauugaagaauauuugu..........................  1  0  S02
...............................................................auccuauugaagaauauuugu..........................  3  0  S02
...............................................................auccuauugaagaauauuugugc........................  1  0  S02
.....................................................................uugaagaauauuugugccuu.....................  1  0  S02
```

Provisional ID	: Maple_Unigene_BMK.37738_193407
Score total	: 0.2
Score for star read(s)	: -1.3
Score for read counts	: 0
Score for mfe	: 2.1
Score for randfold	: 0
Score for cons. seed	: -0.6
Total read count	: 12
Mature read count	: 12
Loop read count	: 0
Star read count	: 0

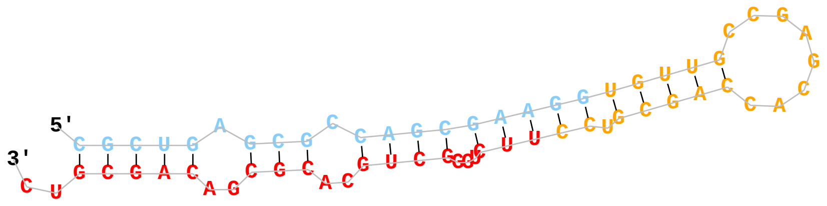

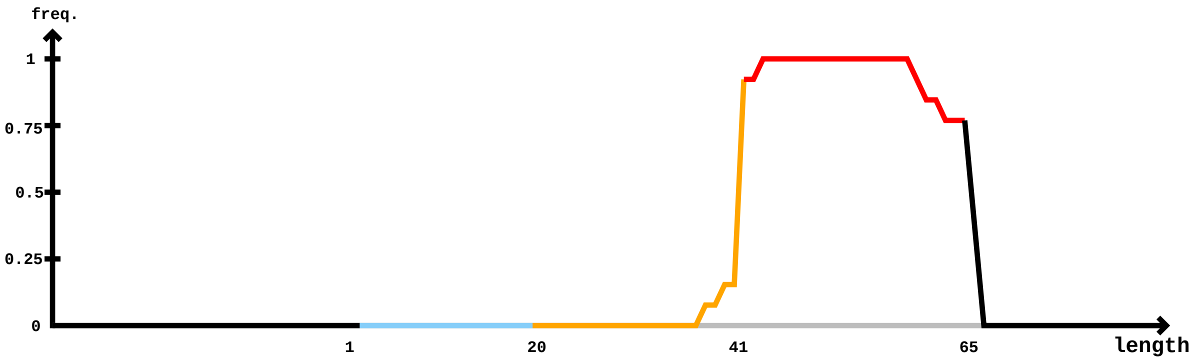

Star Mature

```
5'- caaagugaucgccgacggcuuuuaaggugucgcugagcgccagcgaagguguugccgagcaccagcguccuucugggcugcacgcgacagcgucgccgucguggccuugcgguu   -3'    exp
    ..........((((..((((.....((((.(((((.(((.((((((((((((((........))))).)))))...))))..)))..))))).)))).....))))...)))).    reads    mm    sample
    ......................................................................uucugggcugcacgcgacagcguc....................    2        0     S01

    ..................................................................Auccuucugggcugcacgcgac..........................    1        1     S02
    ....................................................................ccuucugggcugcacgcgaca.........................    1        0     S02
    ......................................................................uucugggcugcacgcgacagcguc....................    5        0     S02
    ......................................................................uucugggcugcacgcgaUagcguc....................    3        1     S02
    ........................................................................cugggcugcacgcgacagc.......................    1        0     S02
```

Provisional ID	:	Maple_Unigene_BMK.37952_193645
Score total	:	18
Score for star read(s)	:	3.9
Score for read counts	:	12.4
Score for mfe	:	2.3
Score for randfold	:	0
Score for cons. seed	:	-0.6
Total read count	:	36
Mature read count	:	31
Loop read count	:	1
Star read count	:	4

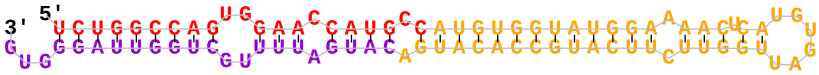

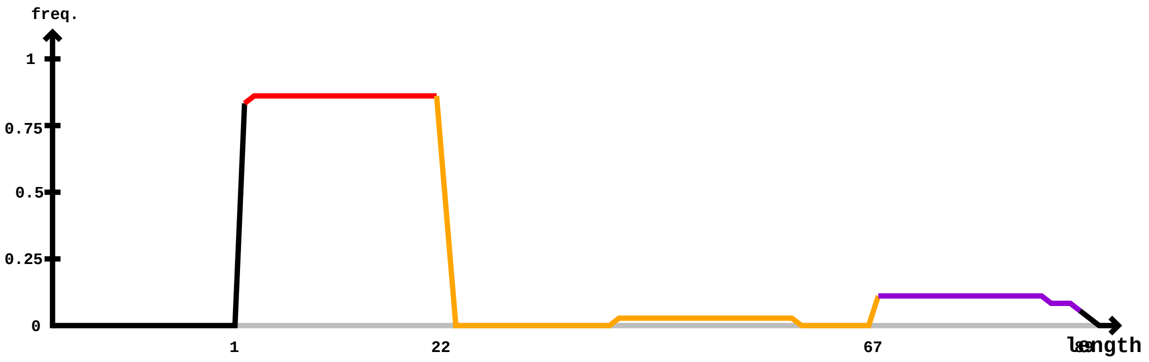

Mature Star

```
5'- gugcacuugcugaaugcaucuggccaguggaaccaugccaugugguauggaaaacucaugugauugguucuucaugccacaugacaugauuuugcugguuagggugcaa  -3'   obs
    gugcacuugcugaaugcaucuggccaguggaaccaugccaugugguauggaaaacucaugugauugguucuucaugccacaugacaugauuuugcugguuagggugcaa         exp
    .(((((.(((.....))).((((((((..(((.((((.((((((((((((.(((.((......))))).)))))))))))))).)))).))).))))))))).))))).   reads   mm   sample
    ..................ucuggccaguggaaccaugcc......................................................................   10      0    S01
    ..................ucuggccaguggaaccaugcU.....................................................................    3       1    S01
    ..................ucuggccaguggaaccaugcA.....................................................................    1       1    S01
    ....................................................................................caugauuuugcugguuag.......   1       0    S01
    ....................................................................................caugauuuugcugguuagggug...   2       0    S01

    ..................ucuggccaguggaaccaugcA.....................................................................    1       1    S02
    ..................ucuggccaguggaaccaugcc......................................................................   13      0    S02
    ..................ucuggccGguggaaccaugcc......................................................................   1       1    S02
    ..................ucuggccaguggaaccaugcU.....................................................................    1       1    S02
    ...................cuggccaguggaaccaugcU......................................................................   1       1    S02
    .........................................................augugauugguuAuucaug.................................   1       1    S02
    ....................................................................................caugauuuugcugguuagggu....   1       0    S02
```

Provisional ID	:	Maple_Unigene_BMK.39316_195217
Score total	:	59.7
Score for star read(s)	:	3.9
Score for read counts	:	57.7
Score for mfe	:	-1.2
Score for randfold	:	0
Score for cons. seed	:	-0.6
Total read count	:	125
Mature read count	:	123
Loop read count	:	1
Star read count	:	1

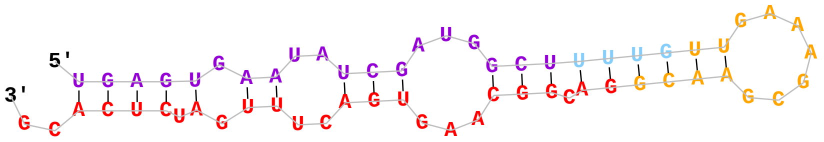

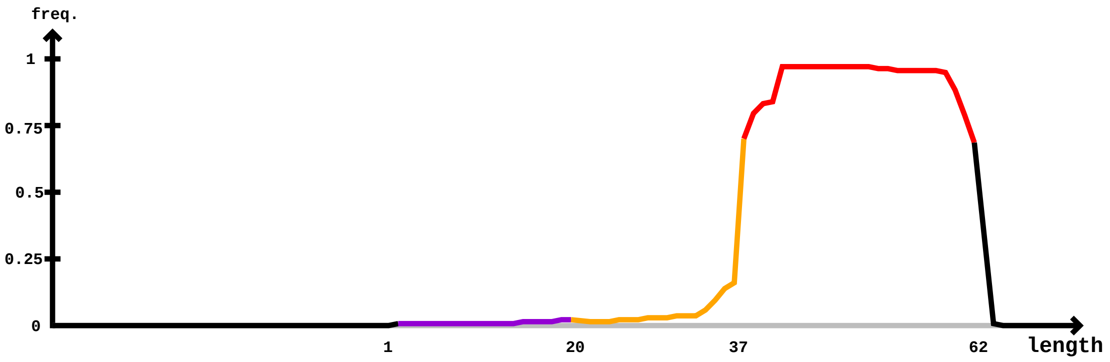

Star Mature

```
5'- gguugggcuugggcagaauauuuuucuaaagaagugagugaauaucgauggcuuuuguugaaagcgaacggacggcaagugacuuugaucucacgcgggcuuaua   -3'   obs
    gguugggcuugggcagaauauuuuucuaaagaagugagugaauaucgauggcuuuuguugaaagcgaacggacggcaagugacuuugaucucacgcgggcuuaua         exp
    ...((((((((...((((.....))))......((((((.((..(((...(((((((((.......)))))).)))...)))..)).).))))).))))))))..   reads   mm   sample
    ..................................ugagugaauaucgauggcu....................................................   1   0   S01
    ...............................................auggcuuuuguugaaagcgaacggacgg..............................   1   0   S01
    ...................................................cuuuuguugaaagcgaacggacgg..............................   1   0   S01
    .........................................................uugaaagcgaacggacgg..............................   1   0   S01
    ............................................................aaagcgaacggacggcaagCgacu.....................   1   1   S01
    ...............................................................gcgaacggacggcaagugacuuC...................   1   1   S01
    ..................................................................aacggacggcaagugacuuugaucucacA..........   1   1   S01
    ..................................................................aacggacggcaagugacuuugaucucacU..........   1   1   S01
    ..................................................................aacggacggcaagugacuuugaucucacg..........   1   0   S01
    ...................................................................acggacggcaagugacuuugaucuca............   1   0   S01
    ...................................................................acggacggcaagugacuuugaucucac...........   2   0   S01
    ...................................................................acggacggcaagugacuuugaucucacA..........   1   1   S01
    ...................................................................acggacggcaagugacuuugaucucacU..........   1   1   S01
    ....................................................................cggacggcaagugacuuugaucuAa............   1   1   S01
    ....................................................................cggacggcaagugacuuugaucucacC..........   3   1   S01
    ....................................................................cggacggcaagugacuuugaucucacA..........   1   1   S01
    .....................................................................Cgacggcaagugacuuugaucucac...........   1   1   S01
    .....................................................................ggacggcaagugacuuugaucucacA..........   1   1   S01
    .....................................................................ggacggcaagugacuuugaucucacU..........   1   1   S01
    ......................................................................gacggcaagugacuuugaucuU.............   1   1   S01
    ......................................................................gacggcaagugacuuugaucuc.............   4   0   S01
    ......................................................................gacggcaagugacuuugaCcuca............   1   1   S01
    ......................................................................gacggcaagugacuuugaucuca............   1   0   S01
    ......................................................................gaUggcaagugacuuugaucucac...........   1   1   S01
    ......................................................................gacggcaagugacuuugaucucac...........   1   0   S01
    ......................................................................gacggcaagugacuuugaucucCc...........   1   1   S01
    ......................................................................gacggcaagugacuuugaucucacC..........   16  1   S01
    ......................................................................gacggcaagugacuuugaucucacU..........   3   1   S01
    ......................................................................gacggcaagugacuuugaucucacA..........   9   1   S01
    ......................................................................gacggcaagugacuuugaucucacg..........   3   0   S01
    ......................................................................gacggcaagugacuuugaucucacgc.........   1   0   S01
    .......................................................................acggcaagugacuuugaucu..............   1   0   S01
    .......................................................................acggcaagugacuuugaucucCc...........   1   1   S01
```

```
                                   Star                                  Mature
gguugggcuugggcagaauauuuuucuaaagaagugagugaauaucgauggcuuuuguugaaagcgaacggacggcaagugacuuugaucucacgcgggcuuaua
.......................................................................acggcaagugacuuugaucucacU..........   1   1   S01
.......................................................................acggcaagugacuuugaucucacC..........   5   1   S01
.......................................................................acggcaagugacuuugaucucacA..........   2   1   S01
........................................................................cggcaagugacuuugaucucC............   1   1   S01
........................................................................cggcaagugacuuugaucucacg..........   2   0   S01
.........................................................................ggcaagugacuuugaucucCc...........   1   1   S01
..........................................................................gcaagugacuuugaucucC............   1   1   S01
..........................................................................gcaagugacuuugaucucacg..........   1   0   S01
..........................................................................gcaagugacuuugaucucacA..........   1   1   S01
..........................................................................gcaagugacuuugaucucacU..........   2   1   S01
..........................................................................gcaagugacuuugaucucacC..........   3   1   S01
..........................................................................gcaagugacuuugaucucacgU.........   1   1   S01
..........................................................................gcaagugacuuugaucucacgc.........   1   0   S01
...........................................................................caagugacuuugaucucacA..........   1   1   S01

....................................................................cggacggcaagugacuuugaucucacC..........   1   1   S02
......................................................................gacggcaagugacuuugaucuc.............   1   0   S02
......................................................................gacggcaagAgacuuugaucuc.............   1   1   S02
......................................................................gacggcaagugacuuugaucuA.............   1   1   S02
......................................................................gacggcaagugacuuugaucucC............   3   1   S02
......................................................................gacggcaagugacuuugaucuca............   2   0   S02
......................................................................gacggcaagugacuuugaucucaA...........   1   1   S02
......................................................................gacggcaagugCcuuugaucucac...........   1   1   S02
......................................................................gacggAaagugacuuugaucucac...........   1   1   S02
......................................................................gacggcaagugacuuugaucucac...........   3   0   S02
......................................................................gacggcaagugacuuugaucucacC..........   7   1   S02
......................................................................gacggcaagugacuuugaucucacA..........   5   1   S02
......................................................................gacggcaagugacuuugaucucacg..........   4   0   S02
......................................................................gacggUaagugacuuugaucucacg..........   1   1   S02
......................................................................gGcggcaagugacuuugaucucacgcg........   1   1   S02
.......................................................................acggcaagugacuuugaucucacC..........   2   1   S02
.......................................................................acggcaagugacuuugaucucacA..........   1   1   S02
........................................................................cggcaagugacuuugaucucacg..........   1   0   S02
........................................................................cggcaagugacuuugaucucacgc.........   1   0   S02
..........................................................................gcaagugacuuugaucuc.............   1   0   S02
..........................................................................gcaagugacuuugaucucacC..........   6   1   S02
..........................................................................gcaagugacuuugaucucacU..........   1   1   S02
...........................................................................caagugacuuugaucuca............   2   0   S02
```

Provisional ID	:	Maple_Unigene_BMK.39829_195726
Score total	:	0.8
Score for star read(s)	:	-1.3
Score for read counts	:	-0.8
Score for mfe	:	0
Score for randfold	:	0
Score for cons. seed	:	3
Total read count	:	10
Mature read count	:	10
Loop read count	:	0
Star read count	:	0

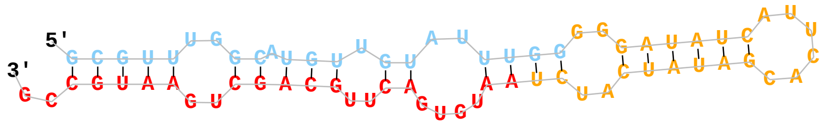

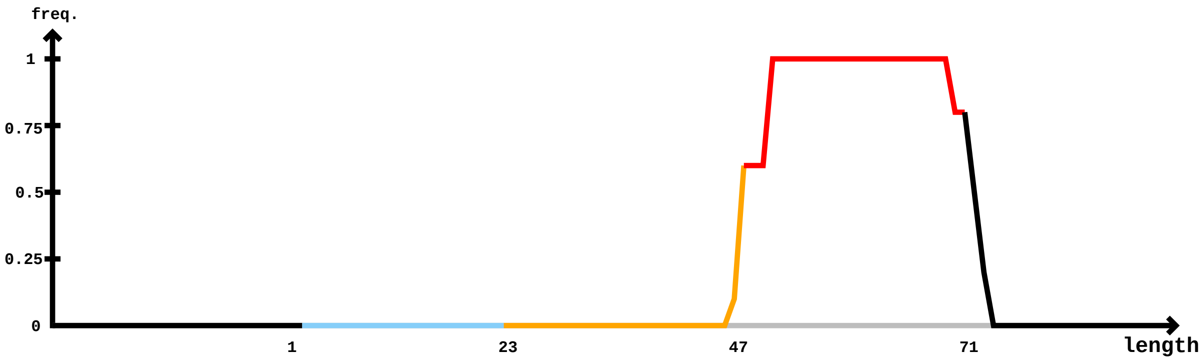


```
                                   Star                                           Mature
5'- uguauuuuaaaauacauugacgaugcguuuggcauguuguauuuggggauaucauucacgauaucaucuaaugugacuugcagcugaaugccguaaauagucuacgcccuuuuu   -3'    exp
    ((((((....))))))...(((..(((((..((.(((.((..((((..((((((......))))))..))))....))..)))))..))))))))....................    reads      mm       sample
    ......................................................................aaugugacuugcagcugaaugccg.....................    2          0        S01
    ......................................................................aaugugacuugcagcugaaugccA.....................    1          1        S01
    ......................................................................aaugugacuugcagcugaaugccgu....................    1          0        S01
    .........................................................................gugacuugcagcugaaugccg.....................    1          0        S01
    .........................................................................gugacuugcagcugaaugccgua...................    1          0        S01
    .........................................................................gugacuugcagcugaaugccguU...................    1          1        S01

    .....................................................................uaaugugacuugcagcugaaugU.......................    1          1        S02
    ......................................................................aaugugacuugcagcugaaugccgu....................    1          0        S02
    .........................................................................gugacuugcagcugaaugU.......................    1          1        S02
```

Provisional ID	:	Maple_Unigene_BMK.41468_197791
Score total	:	2.1
Score for star read(s)	:	-1.3
Score for read counts	:	0
Score for mfe	:	0.4
Score for randfold	:	0
Score for cons. seed	:	3
Total read count	:	19
Mature read count	:	18
Loop read count	:	0
Star read count	:	1

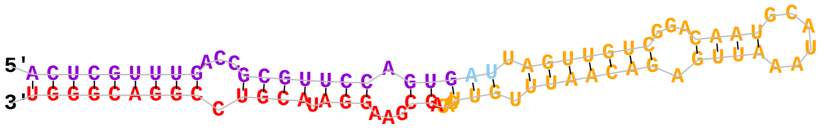

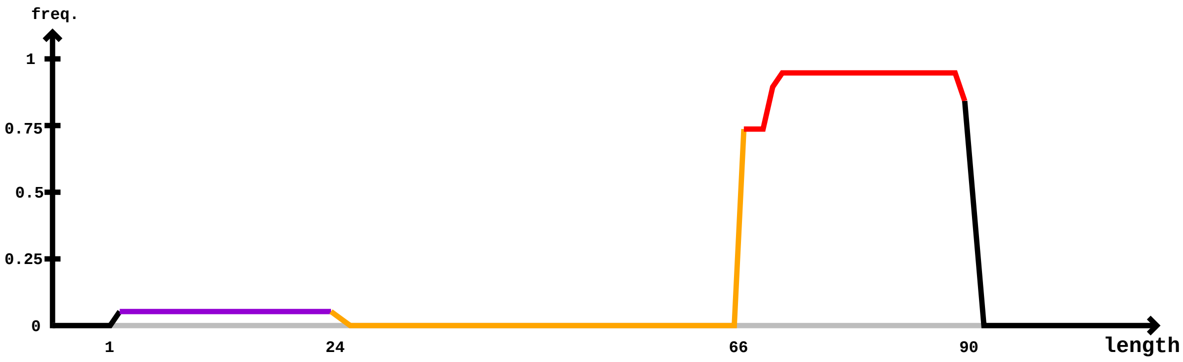

Star **Mature**

```
5'- gaaggacucguuugaccgcguuccagugauuaguugucggacaaugcauaaaauugagacaauuuguugacagcgaaggauacguccggacggguucagagauagauauugaaga   -3'    obs
    gaaggacucguuugaccgcguuccagugauuaguugucggacaaugcauaaaauugagacaauuuguugacagcgaaggauacguccggacgggucagagauagauauugaaga          exp
    ....(((((((((...((((((.(((((.(((((((...((((......)))).))))))).)))....))...))).)))).)))))))))..................   reads     mm     sample
    .....acucguuugaccgcguuccagug......................................................................................   1         0      S01
    .......................................................................agcgaaggauacguccggacgggu...................   6         0      S01
    .......................................................................agcgaaggauacguccggacgggG...................   1         1      S01
    ..........................................................................gaaggauacguccggacgggu...................   1         0      S01
    ...........................................................................aaggauaUguccggacggg....................   1         1      S01

    .......................................................................agcgaaggauacguccggacgggu...................   4         0      S02
    .......................................................................agcgaaggauacguccggacgggG...................   1         1      S02
    .......................................................................agcgaaggauacguccggacgggA...................   1         1      S02
    .......................................................................agcgaagAauacguccggacgggu...................   1         1      S02
    ..........................................................................gaaggauacguccggacggg....................   1         0      S02
    ..........................................................................gaaggauacguccggacgggu...................   1         0      S02
```

Provisional ID	:	Maple_Unigene_BMK.43582_200519
Score total	:	4.9
Score for star read(s)	:	3.9
Score for read counts	:	-3.3
Score for mfe	:	1.4
Score for randfold	:	0
Score for cons. seed	:	3
Total read count	:	5
Mature read count	:	1
Loop read count	:	2
Star read count	:	2

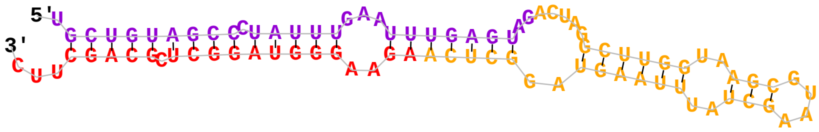

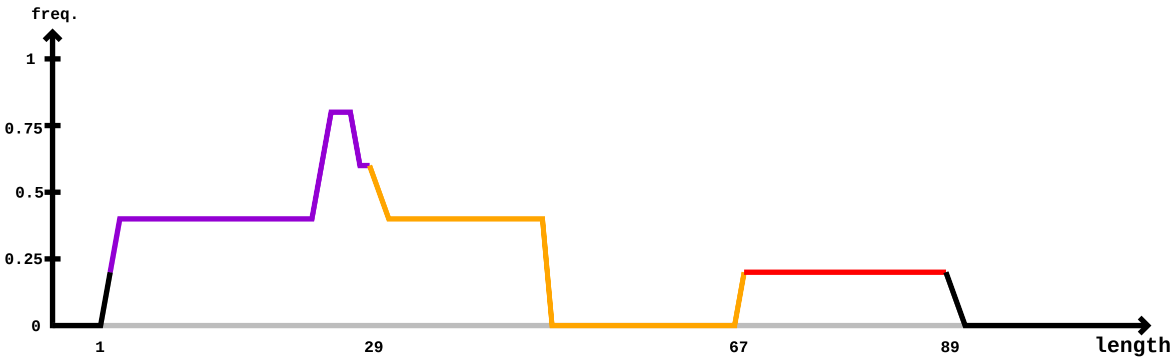

Star **Mature**

```
5'- ugcuugcuguagcccuauuugaauuugaguagacuaggcuugguaagcguaagcuauuuaaguaggcucaagaaggguaggcucgcagcuucgcucagcagaagagccuuac   -3'   obs
    ugcuugcuguagcccuauuugaauuugaguagacuaggcuugguaagcguaagcuauuuaaguaggcucaagaaggguaggcucgcagcuucgcucagcagaagagccuuac         exp
    .....(((((((((.(((((...(((((((.......((((((..(((....)))..))))))..)))))))..))))))))).)))))...((((.......)))).....   reads   mm   sample
    .....gcuguagcccuauuugaauuugaAu..................................................................................   1       1    S01
    ..........................gaguagacuaggcuugguaagcgu..............................................................   1       0    S01
    ...........................aguagacuaggcuugguaagcgu..............................................................   1       0    S01
    ......................................................................agaaggguaggcucgcagcuuc....................   1       0    S01

    ....ugcuguagcccuauuugaauuugaguaU................................................................................   1       1    S02
```

Provisional ID	:	Maple_Unigene_BMK.43875_201097
Score total	:	1426.6
Score for star read(s)	:	3.9
Score for read counts	:	1417.4
Score for mfe	:	2.3
Score for randfold	:	0
Score for cons. seed	:	3
Total read count	:	2792
Mature read count	:	1615
Loop read count	:	0
Star read count	:	1177

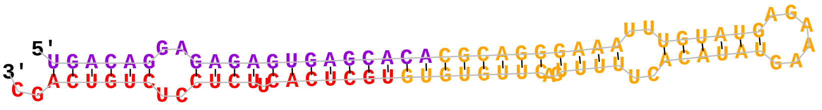

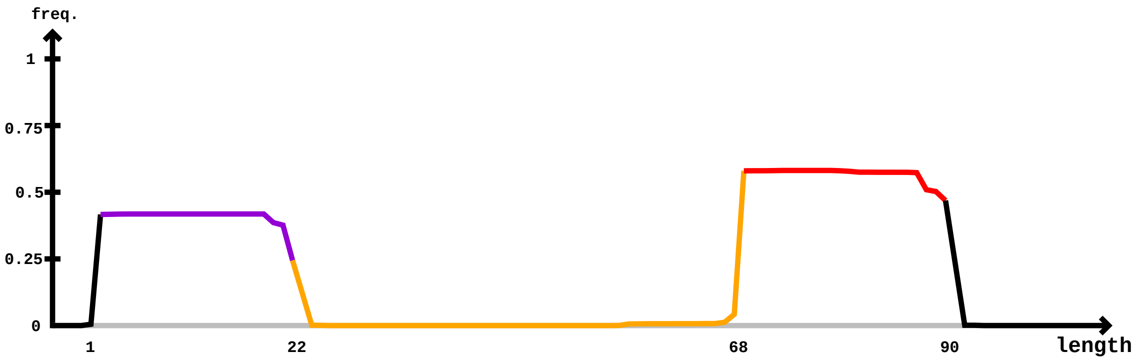

Star **Mature**

```
5'- gacugacaggagagagugagcacacgcagggaaauuuguaugagaaaguauacacuuuuugacuuguguguugcucacuucuccucugucagcucagcucaacccaacu  -3'   obs
    gacugacaggagagagugagcacacgcagggaaauuuguaugagaaaguauacacuuuuugacuuguguguugcucacuucuccucugucagcucagcucaacccaacu         exp
    ((((((((..((((((((((((((((((((((..((((((......))))))..))))..))))))))))))))).))))..))))))).)).............   reads   mm   sample
    ..Uugacaggagagagugagca......................................................................................   1       1    S01
    ..Uugacaggagagagugagcac.....................................................................................   5       1    S01
    ...ugacaggagGgagugagc.......................................................................................   1       1    S01
    ...ugacaggagaUagugagc.......................................................................................   1       1    S01
    ...ugacaggagagagugagc.......................................................................................   32      0    S01
    ...ugacaggagagagugagU.......................................................................................   6       1    S01
    ...ugacaggagagagugagca......................................................................................   10      0    S01
    ...ugacGggagagagugagcac.....................................................................................   1       1    S01
    ...ugacaggagagagugagcaU.....................................................................................   16      1    S01
    ...ugaAaggagagagugagcac.....................................................................................   1       1    S01
    ...ugacaggagagagCgagcac.....................................................................................   1       1    S01
    ...ugacagCagagagugagcac.....................................................................................   2       1    S01
    ...ugacaggagagagugagcGc.....................................................................................   1       1    S01
    ...ugacaggagagagugagcac.....................................................................................   166     0    S01
    ...ugacaggagagCgugagcac.....................................................................................   1       1    S01
    ...ugacaggagagagAgagcac.....................................................................................   4       1    S01
    ...ugacaggCgagagugagcac.....................................................................................   1       1    S01
    ...ugacaggagagagugaCcac.....................................................................................   1       1    S01
    ...ugacaggagagagGgagcac.....................................................................................   1       1    S01
    ...ugacaggaAagagugagcac.....................................................................................   1       1    S01
    ...ugacaggagagagugagcaA.....................................................................................   1       1    S01
    ...ugacagUagagagugagcac.....................................................................................   3       1    S01
    ...ugacaggagagUgugagcac.....................................................................................   1       1    S01
    ...ugacaggagGgagugagcac.....................................................................................   2       1    S01
    ...ugacaggagagagugagAac.....................................................................................   1       1    S01
    ...ugacaggagagagugagcacG....................................................................................   8       1    S01
    ...ugacagCagagagugagcaca....................................................................................   1       1    S01
    ...ugacaggagagagugagcacC....................................................................................   2       1    S01
    ...ugacaCgagagagugagcaca....................................................................................   1       1    S01
    ...ugacaggagGgagugagcaca....................................................................................   1       1    S01
    ...ugacaggagagagugagcGca....................................................................................   1       1    S01
    ...ugacaggagagagugUgcaca....................................................................................   1       1    S01
    ...ugacaggUgagagugagcaca....................................................................................   1       1    S01
```

Star **Mature**

```
gacugacaggagagagugagcacacgcagggaaauuuguaugagaaaguauacacuuuuugacuugugugugcucacuucuccucugucagcucagcucaacccaacu
...ugGcaggagagagugagcaca....................................................................................  2    1  S01
...ugacaggGgagagugagcaca....................................................................................  1    1  S01
...ugaUaggagagagugagcaca....................................................................................  2    1  S01
...ugacaggaUagagugagcaca....................................................................................  1    1  S01
...ugacaggagagagAgagcaca....................................................................................  1    1  S01
...ugacaggagagagugagUaca....................................................................................  1    1  S01
...ugacaggagagagugagcaca....................................................................................  296  0  S01
...ugacaggagagagugaAcaca....................................................................................  1    1  S01
...ugaGgaggagagugagcaca....................................................................................  1    1  S01
...ugacaAgagagagugagcaca....................................................................................  1    1  S01
...ugacaggagagagGgagcaca....................................................................................  1    1  S01
...ugacaggagagagugagcacU....................................................................................  6    1  S01
...Ggacaggagagagugagcaca....................................................................................  1    1  S01
...Cgacaggagagagugagcaca....................................................................................  2    1  S01
...ugacaggagagagugGgcaca....................................................................................  1    1  S01
...ugacaggagagagCgagcaca....................................................................................  1    1  S01
...ugacaggagagagugagcacaA...................................................................................  1    1  S01
...ugacaggagagagugagcacaU...................................................................................  1    1  S01
...ugacaggagagagugagcacac...................................................................................  1    0  S01
...ugacaggagagagugagcacaG...................................................................................  1    1  S01
...ugacaggagagagugagcacacgU.................................................................................  1    1  S01
...ugacaggagagagugagcacaGgc.................................................................................  1    1  S01
....gacaggagagagugagca......................................................................................  1    0  S01
.....acaggagagagugGgcaca....................................................................................  1    1  S01
.........................................................uuugacuugugugugcucacuucu...........................  1    0  S01
..........................................................uugacuugugugugcucacuuc............................  1    0  S01
..........................................................uugacuugugugugcucacuucu...........................  3    0  S01
..........................................................uugacuugugugugUucacuucuc..........................  1    1  S01
..........................................................uugacuugugugugcucacuucuU..........................  3    1  S01
..........................................................uugacuugugugugcucacuucuc..........................  2    0  S01
............................................................gacuugugugugcucacuucuccu........................  1    0  S01
...............................................................uugugugugcucacuucuccucugucagc................  1    0  S01
..................................................................Agugugcucacuucuccucugucagc................  1    1  S01
....................................................................ugugcucacuucuccucuguc...................  2    0  S01
....................................................................ugugcucacuucuccucuguU...................  1    1  S01
....................................................................ugugcucacuucuccucugCc...................  1    1  S01
....................................................................ugugcucacuucuccucugucG..................  1    1  S01
....................................................................ugugcucacuucuccucuguca..................  1    0  S01
.....................................................................gugcucacuucucUucuguc...................  1    1  S01
.....................................................................gugcucacuucuccucuguc...................  13   0  S01
.....................................................................gugcucacuucuccucCguc...................  1    1  S01
.....................................................................gugcucacuucuccucuguca..................  2    0  S01
.....................................................................gugcucacuucuccucCguca..................  1    1  S01
.....................................................................gugcucacuucuccucugucag.................  37   0  S01
.....................................................................Augcucacuucuccucugucag.................  1    1  S01
.....................................................................gugcucacuucuccucugucaA.................  2    1  S01
.....................................................................guAcucacuucuccucugucag.................  1    1  S01
.....................................................................gugcucGcuucuccucugucag.................  1    1  S01
.....................................................................gugcucacuucuccuGugucag.................  1    1  S01
......................................................................ugcucacuucuccucugu....................  4    0  S01
......................................................................uUcucacuucuccucuguc...................  1    1  S01
......................................................................ugcucacuucuccucuguc...................  97   0  S01
......................................................................ugcucacuucuccucuguU...................  13   1  S01
......................................................................ugcGcacuucuccucuguc...................  1    1  S01
......................................................................ugcucacuucuccucuguA...................  1    1  S01
......................................................................ugcucacuCcuccucuguc...................  1    1  S01
......................................................................ugcucacuucuGcucuguc...................  1    1  S01
......................................................................ugcucacuucGccucuguc...................  1    1  S01
......................................................................ugcucacuucuccucugucG..................  1    1  S01
......................................................................ugcucacuucuccucuguca..................  4    0  S01
......................................................................ugcucacuucuccucugucaA.................  3    1  S01
......................................................................ugcucacuucuccucugucag.................  23   0  S01
......................................................................ugcucacuuUuccucugucag.................  1    1  S01
......................................................................ugcGcacuucuccucugucagc................  1    1  S01
......................................................................ugcucacuucuccucugAcagc................  1    1  S01
......................................................................ugcucacuucuccucuguAagc................  1    1  S01
......................................................................ugcucacuucucAucugucagc................  2    1  S01
......................................................................Ggcucacuucuccucugucagc................  2    1  S01
......................................................................ugcucacuucuccucGgucagc................  3    1  S01
......................................................................ugUucacuucuccucugucagc................  1    1  S01
```

```
 Star                                                                     Mature
gacugacaggagagagugagcacacgcagggaaauuuguaugagaaaguauacacuuuuugacuugugugugcucacuucucccugucagcucagcucaacccaacu
......................................................................ugcuUacuucucccugucagc................    2     1     S01
......................................................................uUcucacuucucccugucagc................    1     1     S01
......................................................................uCcucacuucucccugucagc................    2     1     S01
......................................................................ugcucacuucucccugucGgc................    3     1     S01
......................................................................ugcucacuucucccugucagG................    3     1     S01
......................................................................ugcucacuucucccuCgucagc................    4     1     S01
......................................................................ugcucacuucucccugucUgc................    1     1     S01
......................................................................ugcucacCucucccugucagc................    2     1     S01
......................................................................ugcucacuGcucccugucagc................    1     1     S01
......................................................................ugcucacuucAcccugucagc................    1     1     S01
......................................................................ugcucacuCcucccugucagc................    3     1     S01
......................................................................ugcAcacuucucccugucagc................    1     1     S01
......................................................................ugcucGcuucucccugucagc................    1     1     S01
......................................................................ugcucacuucucccugucagc................    758   0     S01
......................................................................ugcucacuucuccAcugucagc................    1     1     S01
......................................................................ugcucacuucCcccugucagc................    2     1     S01
......................................................................Ngcucacuucucccugucagc................    1     1     S01
......................................................................ugcucacuucucccugGcagc................    1     1     S01
......................................................................ugcucacuucucccuguGagc................    1     1     S01
......................................................................ugcucacuuUucccugucagc................    1     1     S01
......................................................................uAcucacuucucccugucagc................    4     1     S01
......................................................................ugcuGacuucucccugucagc................    1     1     S01
......................................................................ugcucacuucucccugucagA................    27    1     S01
......................................................................ugGucacuucucccugucagc................    3     1     S01
......................................................................ugcucaAuucucccugucagc................    1     1     S01
......................................................................ugcucacuuAucccugucagc................    3     1     S01
......................................................................ugcucacuucuccuAugucagc................    1     1     S01
......................................................................ugcucacuucuccuGugucagc................    3     1     S01
......................................................................ugcucacuucuGcucugucagc................    1     1     S01
......................................................................ugcucacuAcucccugucagc................    1     1     S01
......................................................................ugcucacuucuccCcugucagc................    4     1     S01
......................................................................ugcucacAucucccugucagc................    1     1     S01
......................................................................ugcucacuuGucccugucagc................    2     1     S01
......................................................................Cgcucacuucucccugucagc................    1     1     S01
......................................................................ugcucacuucucccugucagU................    141   1     S01
......................................................................ugcCcacuucucccugucagc................    1     1     S01
......................................................................ugcucacuucucccugucagcu...............    2     0     S01
......................................................................ugcucacuucucccugucagcA...............    1     1     S01
......................................................................ugcucacuucucccugucagcAca.............    1     1     S01
.......................................................................gcucacuucucccuguca..................    1     0     S01
.........................................................................ucacuucucccugucagc................    1     0     S01
..........................................................................cacuucucccugucagc................    1     0     S01

..Uugacaggagagagugagca.....................................................................................    1     1     S02
..Uugacaggagagagugagcac....................................................................................    4     1     S02
..cugacagAagagagugagcac....................................................................................    3     1     S02
...ugacaggagagagugagc......................................................................................    41    0     S02
...ugacaggCgagagugagc......................................................................................    1     1     S02
...ugacaggagGgagugagc......................................................................................    1     1     S02
...ugacaggagagagugagU......................................................................................    5     1     S02
...ugacaggagagagGgagc......................................................................................    2     1     S02
...ugacaggagagagCgagc......................................................................................    1     1     S02
...ugacaggagagagugagca.....................................................................................    10    0     S02
...ugacaggagagagugagcG.....................................................................................    3     1     S02
...ugacaggagagagGgagcac....................................................................................    2     1     S02
...ugacaggagagagugagcUc....................................................................................    1     1     S02
...ugacaggagagUgugagcac....................................................................................    1     1     S02
...ugacaggagagagugagcGc....................................................................................    1     1     S02
...ugacagCagagagugagcac....................................................................................    3     1     S02
...ugacaggagagagugCgcac....................................................................................    2     1     S02
...ugacaggagagagugagcac....................................................................................    120   0     S02
...ugacaggagagagugagcaU....................................................................................    16    1     S02
...ugacaggaCagagugagcac....................................................................................    1     1     S02
...ugacaggagGgagugagcac....................................................................................    2     1     S02
...ugaAaggagagagugagcac....................................................................................    1     1     S02
...ugacaggagagagAgagcac....................................................................................    1     1     S02
...ugGcaggagagagugagcac....................................................................................    1     1     S02
...ugacaggagagagCgagcac....................................................................................    2     1     S02
...ugacaggagagaCugagcac....................................................................................    1     1     S02
...ugacaCgagagagugagcaca...................................................................................    1     1     S02
```

Star **Mature**

```
gacugacaggagagagugagcacacgcagggaaauuuguaugagaaaguauacacuuuuugacuugugugugcucacuucuccucugucagcucagcucaacccaacu
...ugacaggagagagCgagcaca....................................................................................   3   1   S02
...ugacaggagGgagugagcaca....................................................................................   2   1   S02
...ugacaggagagagAgagcaca....................................................................................   4   1   S02
...ugacaggGgagagugagcaca....................................................................................   2   1   S02
...ugacaggagagagugagcacC....................................................................................   7   1   S02
...ugacaggagagagGgagcaca....................................................................................   3   1   S02
...Cgacaggagagagugagcaca....................................................................................   1   1   S02
...ugacaggagagagugagcacG....................................................................................   33   1   S02
...uAacaggagagagugagcaca....................................................................................   1   1   S02
...ugacaggagagagugagcaca....................................................................................   277   0   S02
...Ggacaggagagagugagcaca....................................................................................   1   1   S02
...ugacaggagagUgugagcaca....................................................................................   1   1   S02
...ugacaggagagagugagcacU....................................................................................   2   1   S02
...ugacaggUgagagugagcaca....................................................................................   1   1   S02
...ugacaggagagagugaAcaca....................................................................................   1   1   S02
...ugacaggagagagugGgcaca....................................................................................   3   1   S02
....gacaggagagagugagca......................................................................................   1   0   S02
.....acagAagagagugagcacacg..................................................................................   1   1   S02
......caggagagagugagcacacC.................................................................................   1   1   S02
..........................................................uugacuugugugugcucacuuc............................   1   0   S02
..........................................................uugacuugugGgugcucacuucu...........................   1   1   S02
..........................................................uugacuugugugugcucacuucu...........................   1   0   S02
..........................................................uugacuugugugugcucacuucuU..........................   2   1   S02
..........................................................uugacuugugCgugcucacuucuc..........................   1   1   S02
...........................................................ugacuugugugugcucacuuc............................   1   0   S02
....................................................................ugugcucacuucuccucuguc...................   4   0   S02
....................................................................ugugcucacuucuccucuguca..................   3   0   S02
....................................................................ugugcucacuucuccucugucG..................   1   1   S02
.....................................................................gugcucacuucuccucuguc...................   3   0   S02
.....................................................................gugcucacuucuccuUuguc...................   1   1   S02
.....................................................................gugcucacuucuccucuUucag.................   1   1   S02
.....................................................................gugcucacuucuccucugucaA.................   2   1   S02
.....................................................................gugcucacuucuccucugucag.................   15   0   S02
.....................................................................gugcucacuucuccucugucGg.................   1   1   S02
......................................................................ugcucacuucuccucuguc...................   34   0   S02
......................................................................ugcucacuucuccucuguU...................   3   1   S02
......................................................................ugcucacuucuccucugucG..................   3   1   S02
......................................................................ugcucacuucuccucuguca..................   1   0   S02
......................................................................ugcucacuucuccucugucag.................   6   0   S02
......................................................................ugcAcacuucuccucugucagc................   1   1   S02
......................................................................ugcucacuucuccucuguGagc................   1   1   S02
......................................................................ugcucacuucucUucugucagc................   1   1   S02
......................................................................uAcucacuucuccucugucagc................   1   1   S02
......................................................................ugcucacuCcuccucugucagc................   2   1   S02
......................................................................ugcucacuucuccucugucagU................   40   1   S02
......................................................................ugcuUacuucuccucugucagc................   1   1   S02
......................................................................Cgcucacuucuccucugucagc................   1   1   S02
......................................................................ugcucacuucuccuGugucagc................   1   1   S02
......................................................................ugcCcacuucuccucugucagc................   2   1   S02
......................................................................ugcucacuucCccucugucagc................   1   1   S02
......................................................................ugcuAacuucuccucugucagc................   2   1   S02
......................................................................ugcucacuucuccucugucagA................   10   1   S02
......................................................................ugcucacuucuccucCgucagc................   2   1   S02
......................................................................ugcuUcuuucuccucugucagc................   1   1   S02
......................................................................ugcucacuucuccucugucGgc................   2   1   S02
......................................................................ugcucacuucuccucugucagc................   230   0   S02
......................................................................ugcGcacuucuccucugucagc................   1   1   S02
......................................................................ugcucacuucuccucuguUagc................   1   1   S02
......................................................................ugcucGcuucuccucugucagc................   1   1   S02
......................................................................ugcucaUuucuccucugucagc................   2   1   S02
......................................................................ugcucacuAcuccucugucagc................   1   1   S02
......................................................................ugUucacuucuccucugucagc................   1   1   S02
......................................................................ugcucacuucuccucuguAagc................   1   1   S02
......................................................................ugcucacuucuccucugucUgc................   1   1   S02
......................................................................ugcucacuucuccucugucagG................   1   1   S02
......................................................................ugcucacuucuccucugCcagc................   1   1   S02
......................................................................ugcucacuucuccucugucagcC...............   2   1   S02
......................................................................ugcucacuucuccucugucagcuGa.............   2   1   S02
..........................................................................cacuucuccucugucagc................   1   0   S02
```

Provisional ID	:	Maple_Unigene_BMK.44261_202659
Score total	:	1.4
Score for star read(s)	:	3.9
Score for read counts	:	-3.3
Score for mfe	:	1.6
Score for randfold	:	0
Score for cons. seed	:	-0.6
Total read count	:	5
Mature read count	:	2
Loop read count	:	2
Star read count	:	1

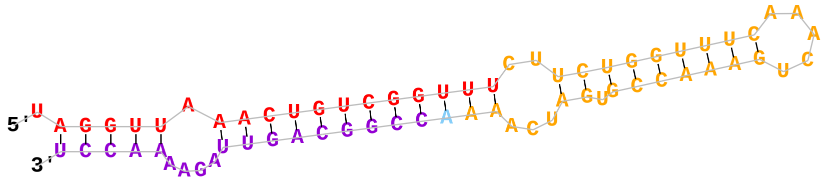

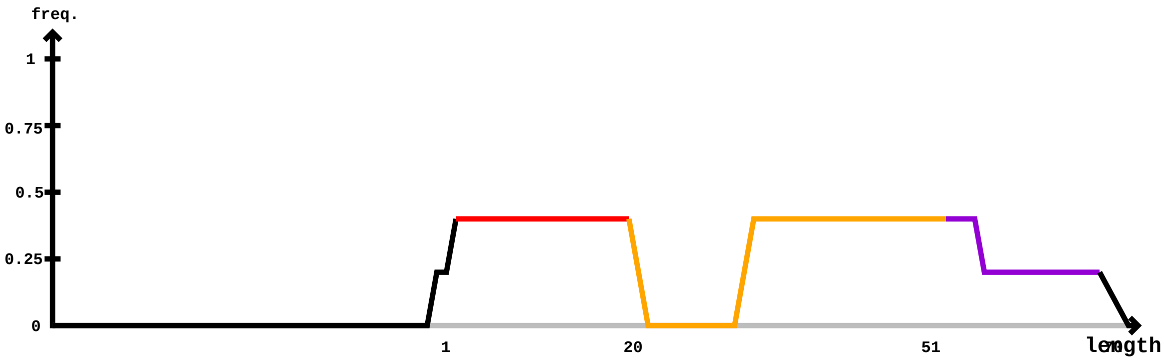

Mature **Star**

```
5'- uggggugggugggggugggcgggaguaagguggccaagguuuagguuaaacugucgguuucuucugguuucaaacugaaaccgugaucaaaaccggcaguuagaaaaccuau   -3'    obs
    uggggugggugggggugggcgggaguaagguggccaagguuuagguuaaacugucgguuucuucugguuucaaacugaaaccgugaucaaaaccggcaguuagaaaaccuau          exp
    ....((((((..(((.(.(........).).)))............((((((((((((..(((((((((.....))))))).))...))))))))))))).....))))))   reads     mm       sample
    ........................................uCgguuaaacugucgguuu....................................................   1         1        S01
    ......................................................................aaacugaaaccgugaucaaaa....................   1         0        S01
    ...........................................................................................ccggcaguuagaaaaccG..   1         1        S01

    .....................................uuuCgguuaaacugucgguuu.....................................................   1         1        S02
    .......................................................................aacugaaaccgugaucaaaaccgA................   1         1        S02
```

Provisional ID	:	Maple_Unigene_BMK.44468_203061
Score total	:	2.1
Score for star read(s)	:	-1.3
Score for read counts	:	0
Score for mfe	:	0.4
Score for randfold	:	0
Score for cons. seed	:	3
Total read count	:	37
Mature read count	:	37
Loop read count	:	0
Star read count	:	0

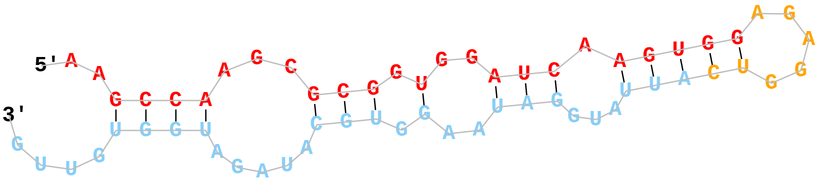

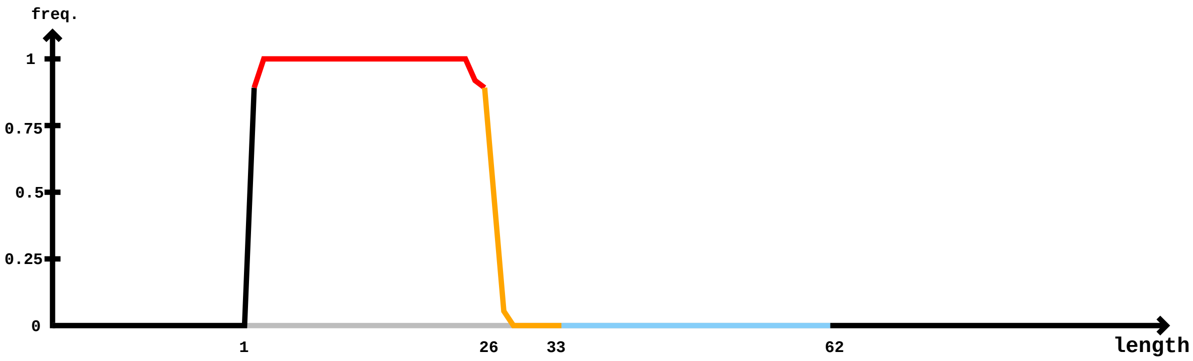

Mature **Star**

```
5'- auacauggugggcgacgacgaagccaagcgcgguggaucaaguggagaggucauuauggauaaggugcauagaugguguugcauaugugguguaaguggagaggucuuuccacag  -3'   exp
    .((((((...(((((......((((...(((.(..(((.(((((.....)))))...)))..).))).....))))))))).))))))......(((((((....)))))))..    reads     mm       sample
    ...................aagccaagcgcggGggaucaagugg......................................................................    2         1        S01
    ...................aagccaagcgcgguggaucaaguAg......................................................................    1         1        S01
    ...................aagccaagcgcgguggaucaagugg......................................................................    11        0        S01
    ....................agccaagcgcgguggaucaagugg......................................................................    1         0        S01

    ...................aagccaagcgcgguggaucaagu........................................................................    2         0        S02
    ...................aagccaagcgcgguggaucaagug.......................................................................    1         0        S02
    ...................aagccaagcgcgguggaucaagugg......................................................................    14        0        S02
    ...................aagccaagcgcgguggaucaaguggaA....................................................................    1         1        S02
    ...................aagccaagcgcgguggaucaaguggaU....................................................................    1         1        S02
    ....................agccaagcgcgguggaucaagu........................................................................    1         0        S02
    ....................agccaagcgcgguggaucaagugg......................................................................    1         0        S02
    ....................agccaCgcgcgguggaucaagugga.....................................................................    1         1        S02
```

Provisional ID	:	Maple_Unigene_BMK.44684_203569
Score total	:	2.8
Score for star read(s)	:	-1.3
Score for read counts	:	0
Score for mfe	:	1.1
Score for randfold	:	0
Score for cons. seed	:	3
Total read count	:	32
Mature read count	:	31
Loop read count	:	0
Star read count	:	1

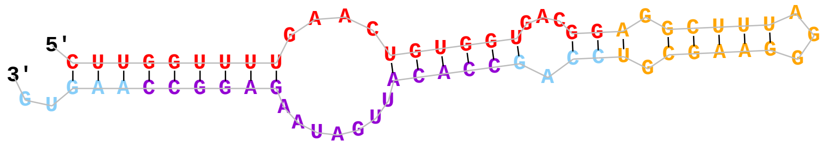

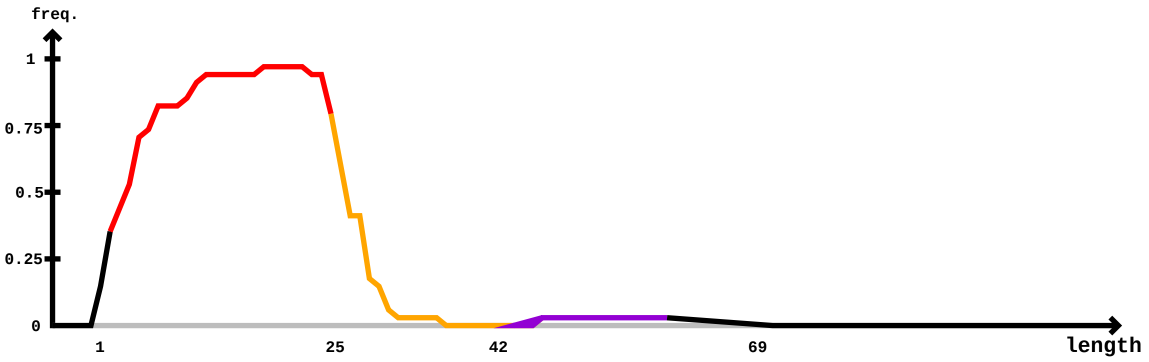

Mature **Star**

```
5'- cacacuugguuuugaacuguggugacggaggcuuuagggaagcguccagccacauugauaagaggccaaguguuacaaaagaaaagcuuuaauucaaccaaaaacacaa   -3'   obs
    cacacuugguuuugaacuguggugacggaggcuuuagggaagcguccagccacauugauaagaggccaaguguuacaaaagaaaagcuuuaauucaaccaaaaacacaa         exp
    .((((((((((((....((((((...(((.(((((...))))).))).)))))).......))))))))))))....((((.....))))..................   reads   mm   sample
    ...acuuggCuuugaacuguggugacg..................................................................................   2   1   S01
    .....uuggCuuugaacuguggugacgga................................................................................   1   1   S01
    ......uggCuuugaacugugguga....................................................................................   1   1   S01
    .......ggCuuugaacuguggugacggagg..............................................................................   3   1   S01
    .........Cuuugaacuguggugacgg.................................................................................   1   1   S01
    .........uuuugaacCguggugacggaggcu............................................................................   1   1   S01
    ...........ugaacCguggugacggaggcuu............................................................................   1   1   S01
    ............gaacuguggugacggagC...............................................................................   1   1   S01
    .............aacuguggugacggagUcu.............................................................................   1   1   S01
    ...................ggugacggaggcuuuaggg.......................................................................   1   0   S01
    .................................................ccacauugauaagaggcU..........................................   1   1   S01

    ...acuuggCuuugaacuguggugacg..................................................................................   3   1   S02
    ....cuuggCuuugaacuguggugacgg.................................................................................   7   1   S02
    .....uuggCuuugaacuguggugacgga................................................................................   2   1   S02
    ......uggCuuugaacuguggugacgg.................................................................................   2   1   S02
    .......ggCuuugaacuguggugacggagg..............................................................................   3   1   S02
    ........gCuuugaacuguggugacggaggc.............................................................................   1   1   S02
    .........Cuuugaacuguggugacggagg..............................................................................   1   1   S02
    ............gaacuguggugacggaggcu.............................................................................   1   0   S02
```

Provisional ID	:	Maple_Unigene_BMK.45392_204566
Score total	:	1.5
Score for star read(s)	:	-1.3
Score for read counts	:	0
Score for mfe	:	-0.1
Score for randfold	:	0
Score for cons. seed	:	3
Total read count	:	16
Mature read count	:	16
Loop read count	:	0
Star read count	:	0

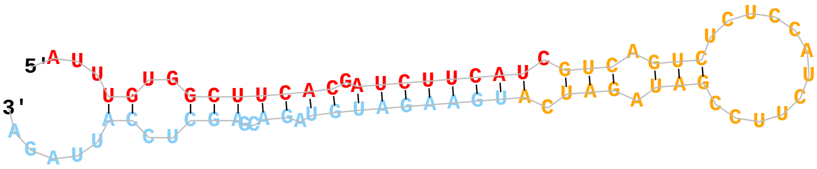

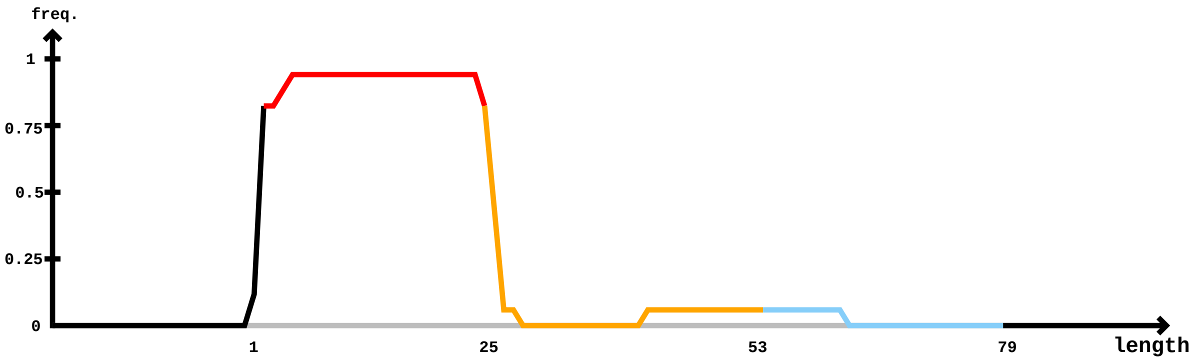

Mature Star

```
5'- ucaauggccaucauugacucauuuguggcuucacgaucuucaucgucagucucuccaucuuccgauagaucaugaagauguagacgagcuccauuagagaugggaucuccucca   -3'    exp
    ((((((.....))))))(((...((..(((((((.((((((((.(((.(((...........))).))).))))))))))).))..))).))...)))...(((.....))).   reads   mm   sample
    ...................cauuuguggcuucacgaucuucau.......................................................................   1       0    S01
    ....................auuuguggcuucacgaucuucauc......................................................................   4       0    S01
    ....................auuuguggcuucacgaucuuAauc......................................................................   1       1    S01
    .......................uguggcuucacgaucuucaucguc...................................................................   1       0    S01

    ...................cauuuguggcuucacgaucuucau.......................................................................   1       0    S02
    ....................auuuguggcuucGcgaucuucauc......................................................................   1       1    S02
    ....................auuuguggcuucacgaucuucauc......................................................................   6       0    S02
    ......................uuguggcuucacgaucuucaucA.....................................................................   1       1    S02
    ............................................................uccgauagaucaugaagaugu.................................   1       0    S02
```

Provisional ID	:	Maple_Unigene_BMK.45417_204614
Score total	:	0.4
Score for star read(s)	:	-1.3
Score for read counts	:	-0.3
Score for mfe	:	-0.8
Score for randfold	:	0
Score for cons. seed	:	3
Total read count	:	11
Mature read count	:	11
Loop read count	:	0
Star read count	:	0

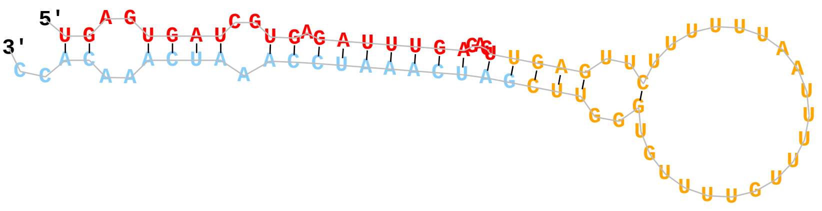

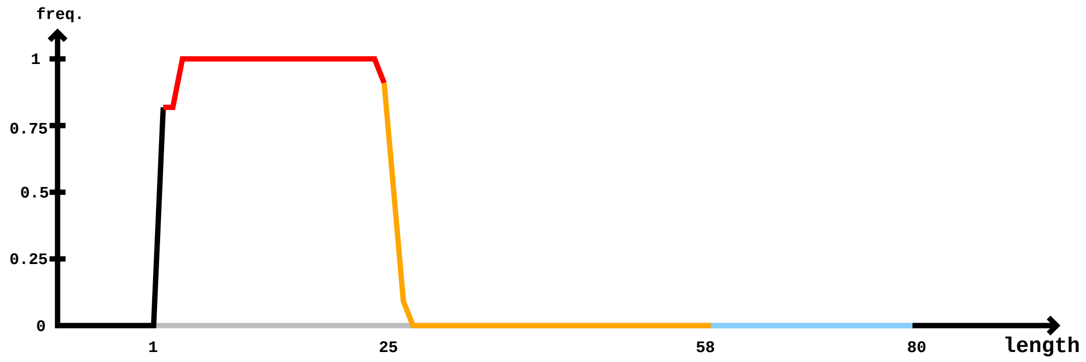

Mature **Star**

```
5'- ugagagaggugagugaucgugagauuugagaguugaguucuuuuuuaauuuuuguuuuguggguucgaucaaauccaaaucaaacaccacuccaagagauca  -3'    exp
    ((.(((.((((..((((..((.(((((((...(((((..(....................)..)))))))))))))).))))..)))).)))))........   reads    mm    sample
    .........ugagugaucgugagauuugagagu.....................................................................   5        0     S01
    .........ugagugaucgugagauuugagagC.....................................................................   1        1     S01
    ...........agugaGcgugagauuugagagu.....................................................................   1        1     S01
    ...........agugaucgugagauuugagaguug...................................................................   1        0     S01

    .........ugagugaucgugagauuugagag......................................................................   1        0     S02
    .........ugagugaucgugagauuugagagu.....................................................................   2        0     S02
```

Provisional ID	:	Maple_Unigene_BMK.46572_206168
Score total	:	0.7
Score for star read(s)	:	-1.3
Score for read counts	:	0
Score for mfe	:	2.6
Score for randfold	:	0
Score for cons. seed	:	-0.6
Total read count	:	41
Mature read count	:	41
Loop read count	:	0
Star read count	:	0

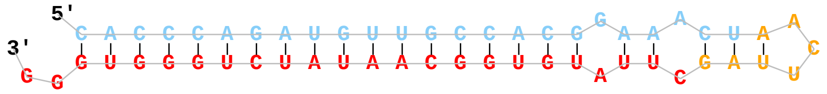

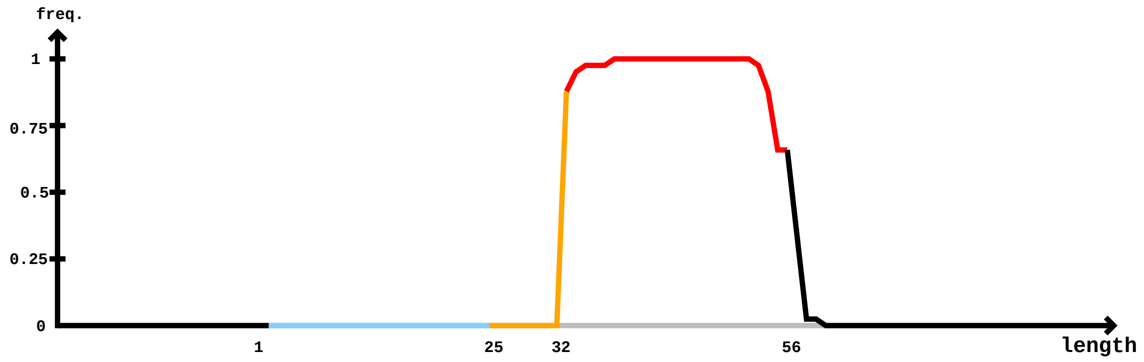

Star Mature

```
5'- cgggauguuucauguaucccacccagauguugccacggaaacuaacuuagcuuauguggcaauaucuggguggggaugcauggugcaucagauguacccaauaacuu -3'    exp
    ...(((((.((((((((((((((((((((((((((((.((.(((...))).)).)))))))))))))))))))))))))))).))))).................. reads  mm  sample
    ...................................................cuGauguggcaauaucuggg.................................... 1      1   S01
    ...................................................cuGauguggcaauaucugggu................................... 2      1   S01
    ...................................................cuGauguggcaauaucugggug.................................. 7      1   S01
    ...................................................cuGauguggcaauaucuggguggg................................ 12     1   S01
    .....................................................Gauguggcaauaucuggguggg................................ 1      1   S01
    ........................................................guggcaauaucuAggugggau............................... 1      1   S01

    ...................................................cuGauguggcaauaucugggu................................... 2      1   S02
    ...................................................cuGauguggcaauaucugggug.................................. 1      1   S02
    ...................................................cuGauguggcaauaucuggguggg................................ 11     1   S02
    ....................................................uGauguggcaauaucugggug.................................. 1      1   S02
    ....................................................uGauguggcaauaucuggguggg................................ 2      1   S02
```

Provisional ID	:	Maple_Unigene_BMK.46572_206170
Score total	:	0.3
Score for star read(s)	:	-1.3
Score for read counts	:	0
Score for mfe	:	2.2
Score for randfold	:	0
Score for cons. seed	:	-0.6
Total read count	:	17
Mature read count	:	17
Loop read count	:	0
Star read count	:	0

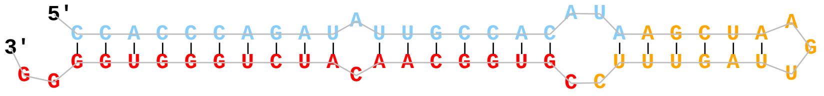

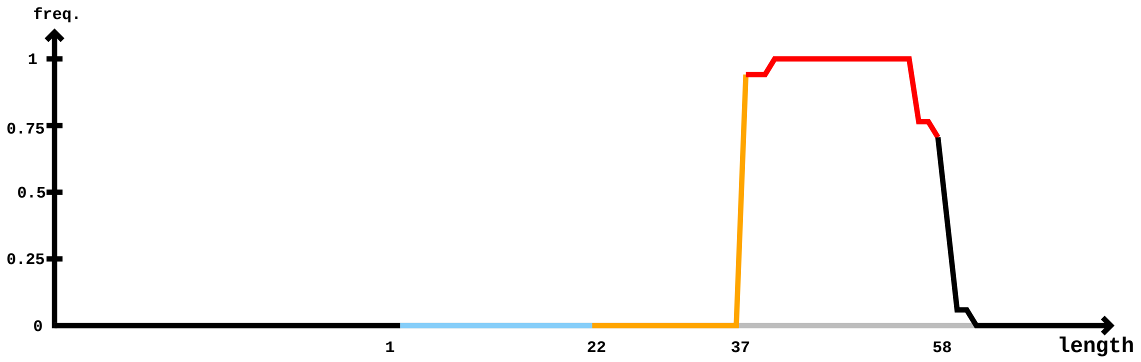

Star Mature

```
5'- aaguuauuggguacaucugaugcaccaugcauccccacccagauauugccacauaagcuaaguuaguuuccguggcaacaucugggugggggauacaugaaacaucccg   -3'    exp
    ..................((((...((((.((((((((((((((.(((((((..((((((...))))))..))))))).))))))))))))))).))))...))))...   reads   mm   sample
    ......................................................................cguggcaacaucugggug....................   3       0    S01
    ......................................................................cguAgcaacaucuggguggg.................   1       1    S01
    ......................................................................cguggcaacaucugggugggg.................   1       0    S01
    ......................................................................cguggcaacaucugggugAgg.................   3       1    S01
    ......................................................................cguggcaacaucugAgugggg.................   1       1    S01

    ......................................................................cguggcaacaucugggug....................   1       0    S02
    ......................................................................cguggcaacaucugggugggg.................   1       0    S02
    ......................................................................cguggcaacaucugggugAgg.................   4       1    S02
    ......................................................................cguggcaacaucuggguggggauG.............   1       1    S02
    .........................................................................ggcaacaucugggugggga................   1       0    S02
```
