# Supplementary figures and images for: Gene Expressing and sRNA Sequencing Show That Gene Differentiation Associates with a Yellow Acer palmatum Mutant Leaf in Different Light Conditions
Source: Biomed Res Int. 2015 Dec 15;2015:843470. doi: 10.1155/2015/843470 (PMC4692996; doi:10.1155/2015/843470)

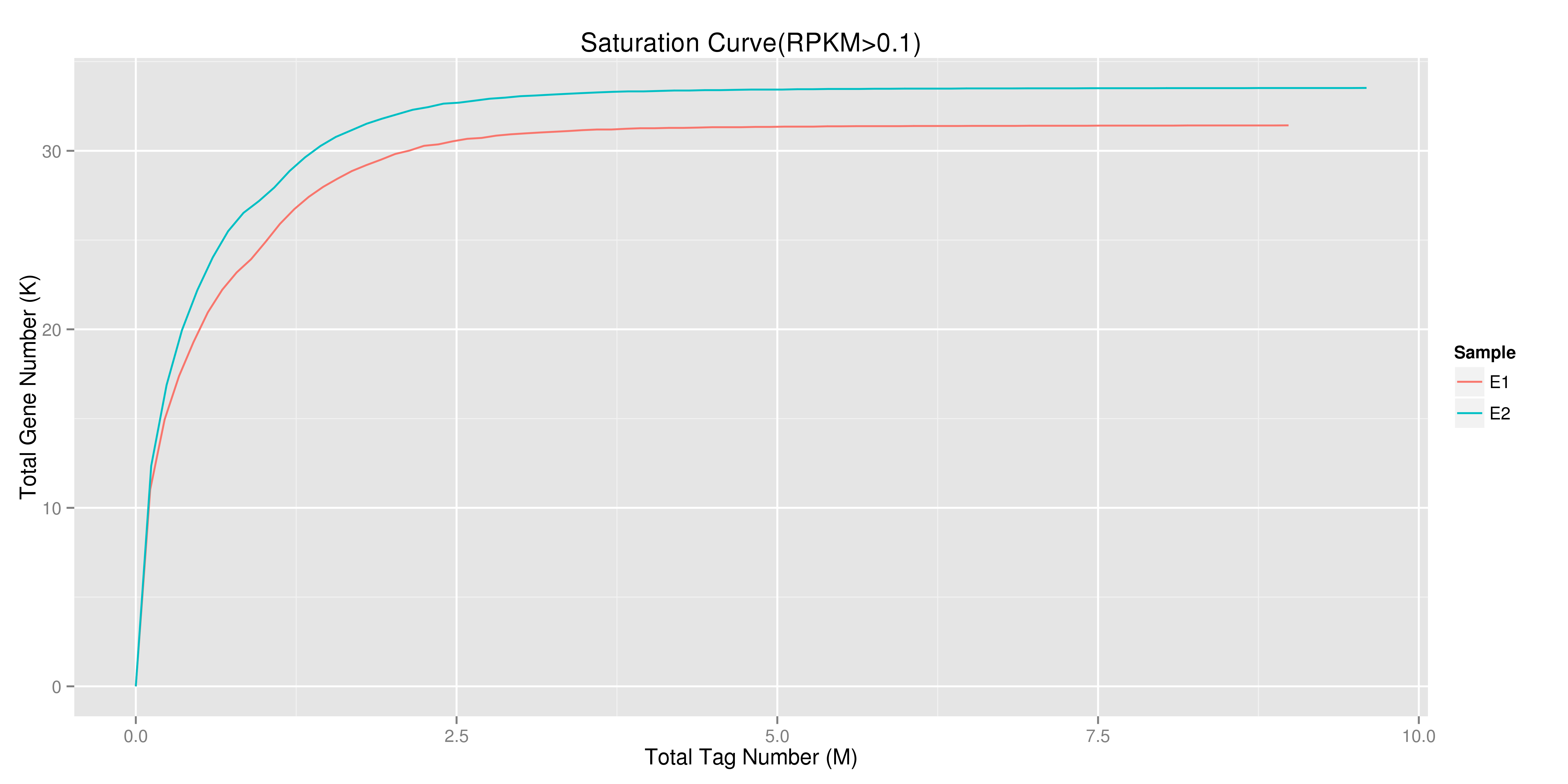

Supplement: Supplementary file 1 — Fig S1. Saturation Curve of high-throughput sequencing. Fig S2. KEGG annotation of DEGs in photosynthesis pathway. Fig S3. KEGG annotation of DEGs related to antenna proteins. [file 843470.f1.zip › Fig-S1 (3).png]

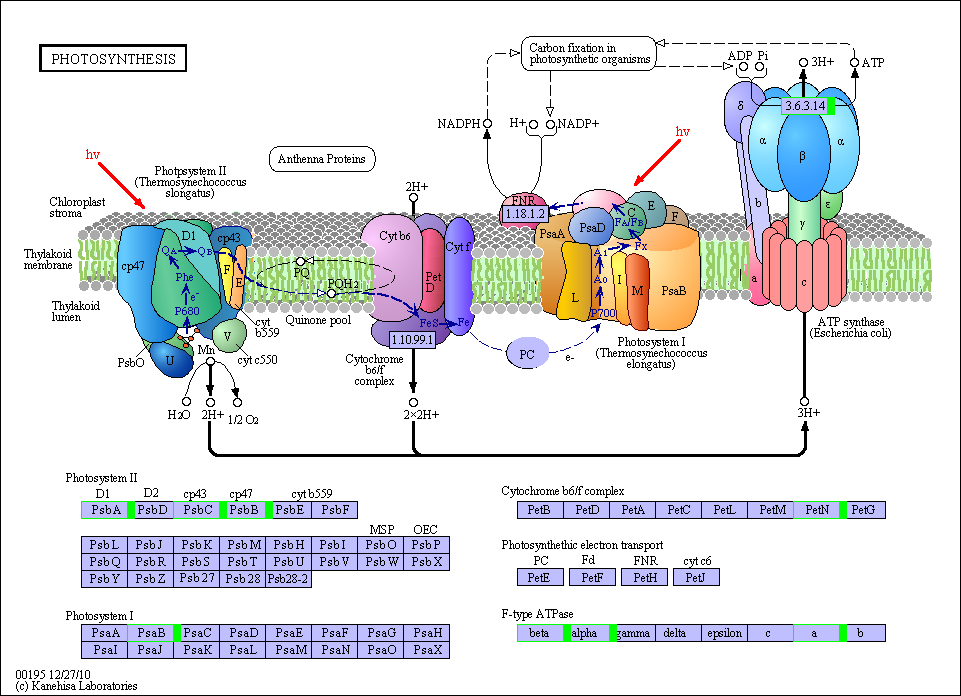

Supplement: Supplementary file 1 — Fig S1. Saturation Curve of high-throughput sequencing. Fig S2. KEGG annotation of DEGs in photosynthesis pathway. Fig S3. KEGG annotation of DEGs related to antenna proteins. [file 843470.f1.zip › Fig-S2-ko00195 (1).png]

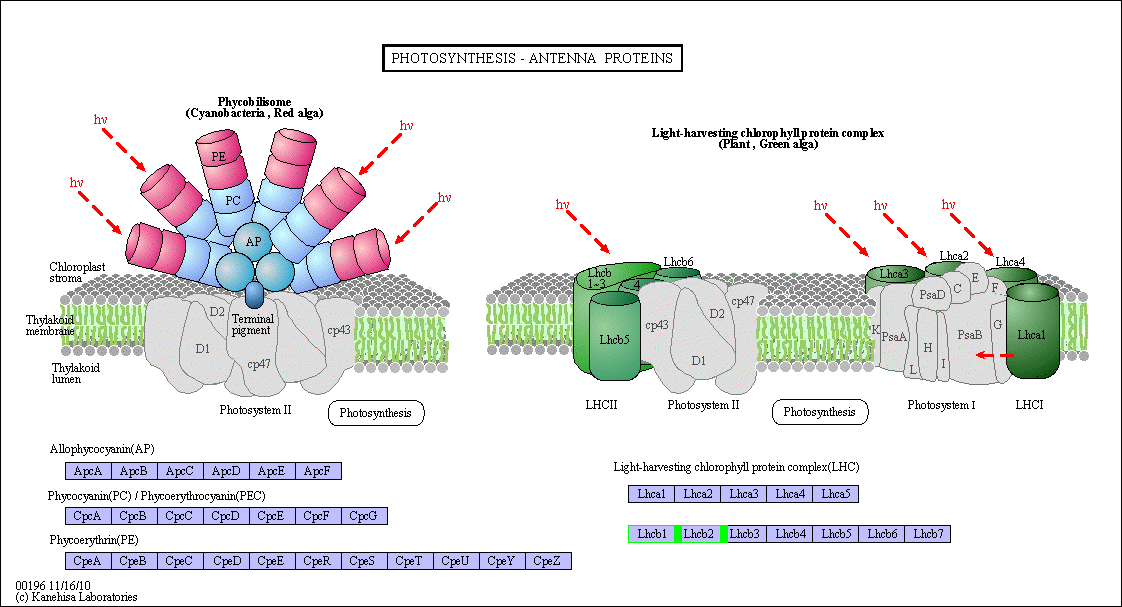

Supplement: Supplementary file 1 — Fig S1. Saturation Curve of high-throughput sequencing. Fig S2. KEGG annotation of DEGs in photosynthesis pathway. Fig S3. KEGG annotation of DEGs related to antenna proteins. [file 843470.f1.zip › Fig-S3-ko00196.png]
